# Predictive and mechanistic insights of *GLTP* on survival in patients with head and neck squamous cell carcinoma

**DOI:** 10.7717/peerj.21611

**Published:** 2026-08-05

**Authors:** Lu Zeng, Yihong Hu, Ziwei Ma, Xinxin Wen, Xianqiong Zou

**Affiliations:** Guilin Medical University, Guilin, Guangxi Zhuang Autonomous Region, China

**Keywords:** GLTP, HNSCC, Prognostic model, Clinical treatment, Bioinformatics analysis

## Abstract

**Background:**

Glycolipid transfer protein (GLTP) is a key regulator of glycosphingolipid distribution between intracellular membranes. Although aberrant GLTP expression has been implicated in various cancers, its role in head and neck squamous cell carcinoma (HNSCC) remains unclear.

**Methods:**

HNSCC samples from The Cancer Genome Atlas (TCGA) were stratified based on *GLTP* expression levels. A comprehensive bioinformatic analysis was conducted to investigate *GLTP’*s expression, functional networks, and impact on the tumor immune microenvironment. To construct a prognostic model, a signature based on genes associated with *GLTP* expression was developed using univariate Cox and LASSO regression analyses, and its robustness was validated in the independent GSE41613 cohort. The role of *GLTP* in cell migration was further verified using wound-healing assays in HSC-3 and SCC-9 cell lines with *GLTP* overexpression or knockdown.

**Results:**

*GLTP* was significantly downregulated in HNSCC tissues. Functional analysis linked *GLTP* to epidermal differentiation, muscle contractile processes, and immunomodulatory mechanisms, with involvement in key pathways such as the chemokine, Ras, and PI3K-Akt signaling pathways. Immune infiltration analysis revealed that, compared to the low GLTP expression group, the high GLTP expression group exhibited increased plasma cell infiltration, decreased levels of resting CD4^+^ memory T cells and M1 macrophages, and generally lower expression of immune checkpoint genes. Based on these findings, a *GLTP*-related risk score model was constructed, which stratified patients into distinct prognostic groups and was validated in an independent cohort. Functional experiments demonstrated that GLTP overexpression inhibited cell migration, whereas its knockdown promoted migration, suggesting that GLTP regulates HNSCC cell motility.

**Conclusions:**

This study developed and validated a *GLTP*-based prognostic model that stratifies HNSCC patients into distinct risk groups. Functional experiments demonstrated that GLTP overexpression inhibited HNSCC cell migration, while its knockdown enhanced migration. These findings indicate that GLTP is involved in HNSCC progression and provide a rationale for further investigation.

## Introduction

Head and neck cancer is the sixth most common cancer worldwide, with head and neck squamous cell carcinoma (HNSCC) accounting for 90% of head and neck malignancies and representing the most common histological subtype. HNSCC is highly invasive and heterogeneous, originating mainly from the mucosal epithelium of the oral cavity, nasopharynx and larynx. In 2022, there were an estimated 890,000 new cases of HNSCC and over 450,000 deaths globally (Bray et al., 2024). Currently, HNSCC is treated with surgery, radiation, and chemotherapy, while immunotherapies are still under active investigation for broader clinical application (Bhatia & Burtness, 2023). However, the significant cytotoxicity associated with radiotherapy and chemotherapy can result in severe local or systemic adverse effects (Dos Santos et al., 2021; Kangboonruang et al., 2020). Statistics reveal that HNSCC patients have a 5-year survival rate of less than 50% (Dos Santos et al., 2021; Hu et al., 2023). These challenges underscore the urgent need for novel prognostic biomarkers and therapeutic targets.

Human glycolipid transfer protein (GLTP) is a small, amphipathic, soluble protein of approximately 24 kDa encoded at locus 12q24.11 on human chromosome 12. Structurally, GLTP adopts an all-α-helical fold consisting of two tightly packed layers that form a hydrophobic cavity for glycolipid binding, a unique architecture designated the ‘GLTP fold’ (Malinina, Patel & Brown, 2017; Mishra et al., 2020). This binding pocket enables GLTP to recognize glycosphingolipids (GSLs) with high specificity and affinity: it engages the glycan head group through a hydrogen-bonding network while accommodating the acyl chains *via* hydrophobic interactions (Backman et al., 2018; Mishra et al., 2020). Based on this unique structural motif, GLTP is recognized as the founding member of a novel protein superfamily (Brown & Mattjus, 2007; Mishra et al., 2020).

Functionally, GLTP mediates the non-vesicular transfer of various GSLs between membranes, thereby regulating GSL homeostasis (Brown & Mattjus, 2007). The transfer process is thought to involve the formation and dissociation of a GLTP-GSL carrier complex at membrane surfaces, with kinetic studies identifying this as the rate-limiting step (Rao et al., 2004). Indeed, GSLs play critical roles in diverse physiological and pathological processes, including cell adhesion, neurogenesis, immune modulation, apoptosis, and drug resistance (Chiricozzi et al., 2021; Dong et al., 2025; Garcia-Ruiz, Morales & Fernández-Checa, 2015). Alterations in GSL metabolism have been closely linked to key malignant features such as immune evasion, chemoresistance, and cancer stem cell maintenance (Clark, Dickinson & Lima, 2023; Longo et al., 2025; Mannini et al., 2025). Given their essential role in organizing membrane signaling platforms like lipid rafts, even minor shifts in GSL composition can influence cell behavior by modulating receptor-mediated pathways, including those activated by growth factors and immune checkpoints (Park et al., 2012; Yokoyama et al., 2021; Zhuo & Guan, 2019). By controlling GSL distribution, GLTP thus serves as a key modulator of these processes, with potential implications for both normal physiology and tumor progression.

For instance, GLTP overexpression in HeLa or HEK-293 cells induces marked cell rounding, an effect that requires an intact glycolipid-binding site and is enhanced by co-expression of δ-catenin, suggesting a role for GLTP in modulating cell morphology through protein-protein interactions (Gao et al., 2011). GLTP also participates in a feedback loop with its GSL cargo: its expression correlates with levels of newly synthesized glucosylceramide (GlcCer) in the endoplasmic reticulum and Golgi, and GlcCer accumulation in turn upregulates GLTP expression (Kjellberg & Mattjus, 2013). More recent work has shown that *GLTP* knockout disrupts vesicle trafficking and alters cellular motility and three-dimensional growth through its interaction with VAP-A, pointing to broader roles in cell physiology (Nurmi et al., 2023). Emerging evidence has implicated GLTP dysregulation in several malignancies, where it suppresses tumor progression in colorectal cancer by regulating cell cycle and necroptosis, modulates immune microenvironment and prognosis in cervical carcinoma, and influences therapeutic resistance in lung cancer (Liu et al., 2022; Mishra et al., 2019; Shi et al., 2022).

However, despite these insights into GLTP’s role in other malignancies, its specific expression pattern, functional significance, and clinical relevance in HNSCC remain largely unexplored.

In this study, we utilized public databases, Cytoscape, and various R (version 4.4.2) software packages to investigate *GLTP* expression and its interaction networks in HNSCC, aiming to gain an initial understanding of the role of *GLTP* in HNSCC. Subsequently, samples from The Cancer Genome Atlas (TCGA) HNSCC cohort were divided into low and high *GLTP* expression groups based on the expression level of *GLTP* (Table S1). We then analyzed the immune landscape and effects of *GLTP* by examining the immune microenvironment and performing enrichment analysis of differentially expressed genes (DEGs). A prognostic signature based on *GLTP*-related genes was subsequently constructed and validated, offering a potential tool for risk stratification in HNSCC. To investigate the functional role of GLTP in HNSCC cell migration, wound-healing assays were conducted on cells following GLTP overexpression and knockdown. Collectively, this study establishes a *GLTP*-based prognostic model and provides preliminary insights into the potential role of GLTP in HNSCC cell migration.

## Materials and Methods

### Dataset

The RNA-seq FPKM data and corresponding clinical information for HNSCC samples were acquired from the Sangerbox database (http://sangerbox.com/), a platform that provides access to TCGA datasets along with integrated clinical annotations, facilitating the download of both expression and clinical data from a single source. Initially, 546 HNSCC samples with available expression data were obtained. Samples were excluded based on the following criteria: (i) missing survival data (overall survival time or status), and (ii) incomplete clinical annotation required for subsequent analyses. After applying these filtering steps, 379 samples remained for further analysis. For gene filtering, Ensemble IDs were converted to Gene Symbols, and genes with zero expression across all samples were removed prior to downstream analysis. The resulting expression matrix was then log_2_(x + 1) transformed to approximate a normal distribution. The GSE41613 cohort (*n* = 97) was downloaded from the Gene Expression Omnibus (GEO) database. Overlapping genes common to both the TCGA-HNSCC and GSE41613 datasets were identified to ensure consistency across platforms. To correct for non-biological technical variations between the two cohorts, batch effect adjustment was performed using the ComBat function from the ‘sva’ package (version 3.42.0) in R. The adjustment model included study cohort as the batch variable, while preserving biological variation associated with clinical covariates of interest.

### GLTP expression in HNSCC

In the TCGA module of the UALCAN database (https://ualcan.path.uab.edu/index.html), a comparative table of *GLTP* expression levels in cancer tissues and adjacent normal tissues of HNSCC patients was obtained, along with *GLTP* expression figures stratified by tumor grade, patient age, gender, cancer stage and sample type (Chandrashekar et al., 2017).

### Protein interactions

To identify genes co-expressed with GLTP in HNSCC, the TCGA (Firehose Legacy) dataset was accessed *via* the cBioPortal database (https://www.cbioportal.org/). Using the ‘Co-expression’ module in cBioPortal, Spearman’s correlation coefficients were calculated between GLTP and all other genes in the dataset based on RNA-seq expression data (FPKM values). Genes with |Spearman’s ρ| > 0.5 and a Benjamini-Hochberg adjusted *P*-value (FDR) < 0.05 were considered significantly co-expressed with *GLTP*. Both positively and negatively correlated genes were included in the analysis, and directionality was noted based on the sign of Spearman’s ρ but was not used as a separating criterion in subsequent protein-protein interaction network construction. The overlapping genes identified from this analysis were then subjected to protein-protein interaction (PPI) network analysis using the STRING database (https://string-db.org/) with a high confidence score threshold (minimum interaction score > 0.7) and *Homo sapiens* as the selected organism (Szklarczyk et al., 2021). Finally, the top 10 hub proteins with the highest degree of connectivity were identified using the ‘cytoHubba’ plugin (Chin et al., 2014) in Cytoscape, based on the Matthews correlation coefficient.

### Consistency clustering and identification of DEGs

To identify intrinsic subgroups of HNSCC patients based on GLTP expression, K-means clustering (k = 2, nstart = 25) was applied. This method partitions samples by maximizing inter-group differences while minimizing intra-group variability. Based on the mean expression levels of the resulting clusters, the two groups were designated as *GLTP* low-expression (*n* = 207) and *GLTP* high-expression (*n* = 172). A fixed random seed (set.seed = 123,456) was used to ensure reproducibility. The DEGs between the two GLTP expression groups were evaluated using R’s t-test function, and each gene’s statistical significance was determined using the Benjamini-Hochberg method to control the false discovery rate (FDR). Subsequently, volcano plots were drawn using R packages such as ‘grid’ (version 4.4.2), ‘ggrepel’ (version 0.9.6), and ‘ggfun’ (version 0.1.8), and DEGs that met the FDR < 0.05 standard and |fold change| > 1.2 were chosen for further analysis.

### Enrichment analysis

R packages such as ‘clusterProfiler’ (version 4.14.4) and ‘enrichplot’ (version 1.26.5) were used to perform Gene Ontology (GO), Kyoto Encyclopedia of Genes and Genomes (KEGG), and Gene Set Enrichment Analysis (GSEA) on the entire set of DEGs. The GO analysis covered three ontologies: biological process, molecular function, and cellular component. The source of the annotation data is ‘org.Hs.eg.db’ (version 3.20.0). For GSEA, 1,000 resampling permutations were performed, with a minimum gene set size of 15, and *P* < 0.05 was considered statistically significant.

### Characterisation of the immune landscape

The CIBERSORT method was used to evaluate immune cell infiltration for 22 immune cell types (Newman et al., 2015). The proportions of different tumor-infiltrating immune cells (TICs) was analyzed for correlation with GLTP expression. In addition, based on the GLTP high- and low-expression groups, the Wilcoxon rank-sum test was used to evaluate the relationship between *GLTP* expression and immune checkpoint genes and human leukocyte antigen (HLA) genes in HNSCC samples; a difference was deemed statistically significant if *P* < 0.05. ‘e1071’ (version 1.7.16), ‘parallel’ (version 4.4.2), ‘tidyverse’ (version 2.0.0), ‘ggpubr’ (version 0.6.0), ‘ggstatsplot’ (version 0.13.0), ‘ggplot2’ (version 3.5.1), and others were used for the aforementioned analyses and figure generation.

### Prognostic model construction and validation

Survival time and status data of patients in the TCGA HNSCC cohort were integrated using R to construct survival objects for survival analysis. R packages including ‘pbapply’ (version 1.7.2), ‘glmnet’ (version 4.1.8), and ‘tidyverse’ (version 2.0.0) were used for univariate Cox and LASSO-Cox analysis to screen for genes significantly correlated with survival status (*P* < 0.05) and to construct a proportional hazards model. HNSCC patients were then divided into high-risk and low-risk groups, and R software packages such as ‘ggplot2’ (version 3.5.1), ‘survival’ (version 3.8.3), and ‘survminer’ (version 0.4.9) were used to assess prognostic differences. Principal component analysis (PCA) and receiver operating characteristic (ROC) curve plotting were also performed using R packages such as ‘scatterplot3d’ (version 0.3.44) and ‘timeROC’ (version 0.4).

For external validation, the batch-corrected GSE41613 dataset was used. Patients were stratified into high- and low-risk groups using the same cutoff as the TCGA cohort to validate the prognostic model.

### Construction of independent prognostic markers and nomogram based on risk score

Lastly, univariate and multivariate independent prognostic analyses were performed on the available patient clinical data from the TCGA HNSCC cohort and the GSE41613 validation set. Sex (male as reference) and HPV status (negative as reference, retrieved from cBioPortal at https://www.cbioportal.org) were modeled as categorical variables. For T stage, N stage, and grade, these variables were treated as ordinal variables using numeric codes (1, 2, 3, 4), with hazard ratios interpreted as the risk change per unit increase in category code. This approach is commonly used when sample sizes in higher categories are limited (*e.g*., T4, N3, stage IV, and G4 in this cohort), as it preserves degrees of freedom, avoids unstable estimates from sparse data, and reduces the risk of overfitting. A nomogram integrating independent prognostic markers was constructed to predict 1-, 3-, and 5-year overall survival probabilities for HNSCC patients. Calibration curves were generated to evaluate the agreement between nomogram-predicted survival and observed survival outcomes.

### *In vitro* functional validation

The HSC-3 and SCC-9 stable cell lines (GLTP-overexpressing and knockdown) were used in this study. Briefly, lentiviral particles were generated in 293T cells using a three-plasmid packaging system (*pSPAX2* and *pMD2.G*) together with the *GV492* or *GV493* constructs obtained from GeneChem (Shanghai, China). The *GV492* vector is based on the *pLVX-Puro* backbone and contains a Ubi-MCS-3FLAG-CBh-gcGFP-IRES-puromycin cassette, with human *GLTP* (NM_016433.4) cloned into the *BamHI* and *AgeI* sites. The vector also carries an enhanced green fluorescent protein (EGFP) reporter and a puromycin resistance gene for selection. The *GV493* vector is based on the *pLKO.1-puro* backbone and contains an hU6-MCS-CBh-gcGFP-IRES-puromycin cassette, with the shRNA targeting *GLTP* cloned into the *AgeI* and *EcoRI* sites. The shRNA target sequence for *GLTP* was 5′-GCACTGTACGCAGCACCCTATAAGT-3′. A scrambled shRNA sequence (5′-TTCTCCGAACGTGTCACGT-3′) was used as a negative control. HSC-3 and SCC-9 cells were transduced at a multiplicity of infection (MOI) of 10 and 20, respectively. Stable transductants were selected with 1 μg/mL puromycin for 7 days, and single-cell clones were expanded. The GLTP-modified HSC-3 and SCC-9 cell lines were verified by reverse transcription quantitative real-time PCR (RT-qPCR). HSC-3 cells in RPMI-1640 and SCC-9 in DMEM at 37 °C/5% CO_2_. Three biological replicates, each measured in triplicate. RNA extracted using RNAiso Plus (TaKaRa); concentration measured by microvolume spectrophotometer. RT using FastKing cDNA Kit (TIANGEN) with 1 μg RNA in 20 μL (42 °C 30 min, 95 °C 3 min). qPCR using SYBR Green Premix (TIANGEN) on CFX96 in 10 μL (5 μL Premix, 0.3 μL each 10 μM primer, 1 μL cDNA, 3.4 μL H_2_O). Cycling: 95 °C 5 min; 40 cycles of 95 °C 10 s, 60 °C 30 s; acquisition at 65 °C 5 s. Melting curve and NTC (Cq > 40) confirmed specificity. The primer sequences used were: *GLTP* forward 5′-TGGGTCCCCAGTGTTTACTC-3′, reverse 5′-TCTCCACCTCCAGGATGTTC-3′; *ACTB* forward 5′-CATGTACGTTGCTATCCAGGC-3′, reverse 5′-CTCCTTAATGTCACGCACGAT-3′. The relative expression of *GLTP* mRNA was calculated using the $2^{\rm {(-\Delta \Delta Ct)}}$ method with *ACTB* as an internal control. All reactions were performed in triplicate. Data are presented as mean ± SD from three independent experiments. Statistical comparisons between groups were performed using two-tailed unpaired Student’s *t*-test.

For wound healing assay, HSC-3 and SCC-9 cells were cultured in 10 cm dishes and passaged three times prior to the experiment. Cells were then seeded into 6-well plates and grown to >90% confluence in complete medium containing 10% fetal bovine serum (FBS). A sterile pipette tip was used to create straight scratches in the cell monolayer. After gently washing with PBS to remove detached cells, the medium was replaced with fresh serum-free medium. Wound closure was monitored using a Nikon inverted microscope equipped with a 10× objective lens. For HSC-3 cells, images were captured at 0, 6, and 12 h post-scratch; for SCC-9 cells, images were captured at 0, 24, and 48 h post-scratch using a Nikon digital camera. Each experiment was performed in triplicate. The migration distance at each time point was quantified using ImageJ software, and the wound healing rate was calculated as (migration distance at each time point/initial wound width at 0 h) × 100% and expressed as the mean ± SD. Two-way ANOVA was used for intergroup comparisons with GraphPad Prism 10. A *P*-value of less than 0.05 was considered statistically significant.

### Data access and ethical considerations

This study constitutes a retrospective analysis of fully anonymized public data obtained from the TCGA-HNSCC and GEO (GSE41613) repositories. The data were accessed for research purposes on 15 March 2025. The original data collection for these public resources was conducted in accordance with the ethical standards of their respective institutional review boards, and all participants provided informed consent. As this study did not involve direct interaction with human subjects or access to identifiable private information, it was deemed exempt from requiring additional ethics approval or IRB review, in compliance with the National Institutes of Health (NIH) guidelines for the use of public datasets.

## Results

### Expression analysis of GLTP in HNSCC

**Figure 1:**
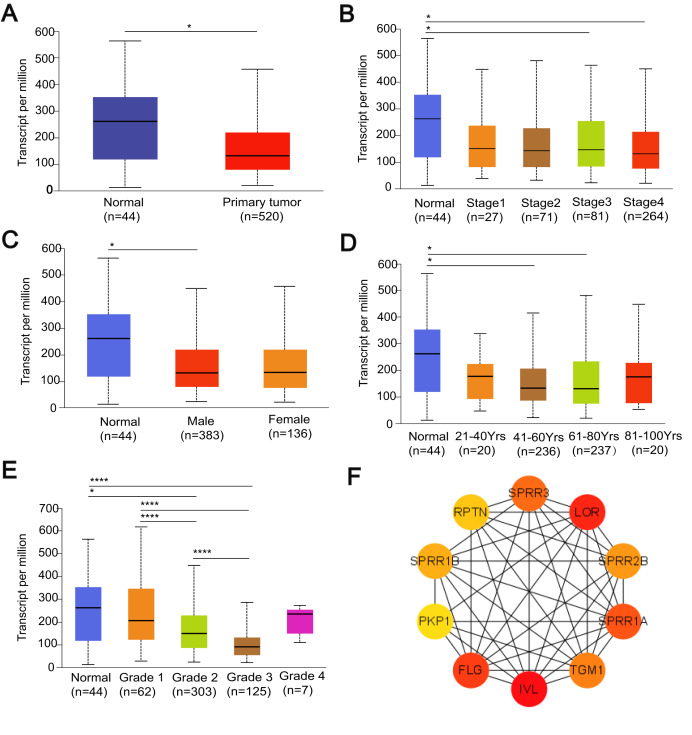
Expression of *GLTP* and related hub genes in HNSCC. (A) Expression of *GLTP* in HNSCC and normal tissues. (B) Expression of *GLTP* in HNSC based on individual cancer stages. (C) Expression of *GLTP* in HNSC based on patient’s gender. (D) Expression of *GLTP* in HNSC based on patient’s age. (E) Expression of *GLTP* in HNSC based on tumor grade. (F) Top 10 hub genes most related to *GLTP*. *P < 0.05, ****P < 0.0001. HNSCC, head and neck squamous cell carcinoma; SPRR, small proline-rich protein; LVL, involucrin; LOR, loricrin; FLG, filaggrin; TGM1, transglutaminase 1; RPTN, repetin; PKP1, plakophilin 1.

Compared with adjacent normal tissues, the expression of *GLTP* mRNA in HNSCC showed significant downregulation (Fig. 1A). In addition, patients with stage III and IV disease had significantly lower *GLTP* expression levels than normal tissues (Fig. 1B); both male and female tumor groups had lower levels of *GLTP* than normal controls, with the male group showing a more marked decrease (Fig. 1C); patients aged 41–60 and 61–80 years had significantly lower levels of *GLTP* expression (Fig. 1D); and tumor grading analysis revealed a significant gradient of *GLTP* expression from grade 1 to grade 3 (Fig. 1E). In HNSCC, 241 genes showed significant co-expression with *GLTP* (|Spearman’s ρ| > 0.5, *P* < 0.05). The PPI network consisted of 276 nodes and 210 edges (interaction score ≥ 0.7). The top 10 hub proteins identified by the PPI were small proline-rich protein 1A (SPRR1A), SPRR1B, SPRR2B, SPRR3, involucrin (IVL), loricrin (LOR), filaggrin (FLG), transglutaminase 1 (TGM1), repetin (RPTN), and plakophilin 1 (PKP1) (Fig. 1F).

### Identification of DEGs

**Figure 2:**
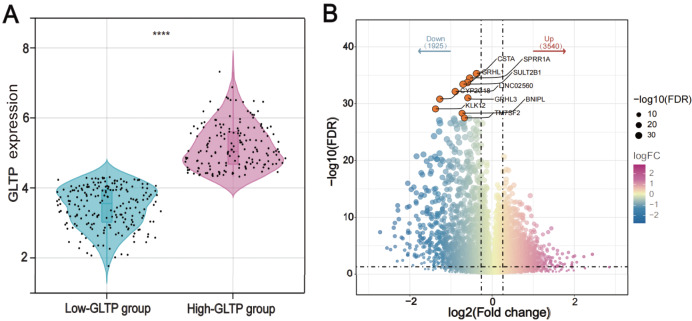
Divide HNSCC samples into *GLTP* high/low expression groups and identify DEGs between them. (A) Comparison of *GLTP* expression levels between high/low expression groups on violin plot. (B) Distribution of DEGs between *GLTP* high expression group and low expression group (FDR < 0.05 and |fold change| > 1.2). ****P < 0.0001. HNSCC, Head and neck squamous cell carcinoma; DEG, differentially expressed genes.

We categorized the HNSCC samples into high (*n* = 207) and low (*n* = 172) *GLTP* expression groups (Fig. 2A). Compared with the low *GLTP* expression group, the high *GLTP* expression group had a total of 5,464 DEGs, of which 3,540 were upregulated and 1,924 were downregulated (Fig. 2B) (Table S2).

### Functional analysis of DEGs

**Figure 3:**
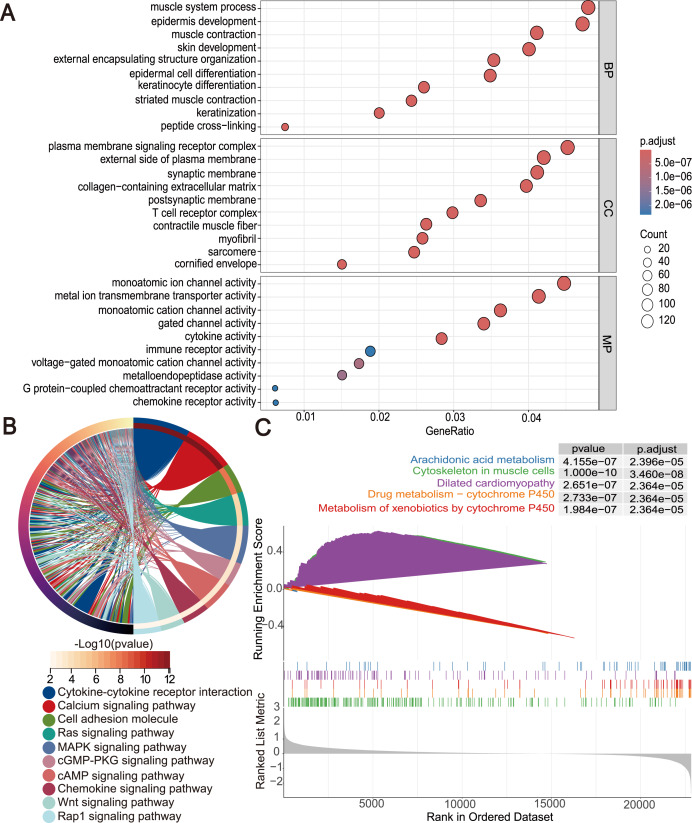
Enrichment analysis. (A) GO analysis of DEGs. (B) KEGG pathway analysis of DEGs. (C) Results of GSEA of the DEGs. DEGs, Differentially expressed genes; GO, Gene Ontology; KEGG, Kyoto Encyclopedia of Genes and Genomes; GSEA, Gene Set Enrichment Analysis.

According to the GO analysis, these DEGs were found to be associated with various biological processes, including ‘muscle system process’, ‘skin development’, ‘plasma membrane signaling receptor complex’, ‘monatomic ion channel activity’, and ‘immune receptor activity’ (Fig. 3A). KEGG pathway analysis revealed that these DEGs were linked to ‘Cytokine-cytokine receptor interaction’, ‘Calcium signaling pathway’, ‘Ras signaling pathway’, ‘MAPK signaling pathway’, and others (Fig. 3B). Furthermore, GSEA revealed that DEGs were significantly enriched in processes such as ‘Cytoskeleton in muscle cells’, ‘Arachidonic acid metabolism’, and ‘Metabolism of xenobiotics by cytochrome P450’ (Fig. 3C), as well as in pathways such as the ‘Chemokine signaling pathway’ and ‘PI3K-Akt signaling pathway’ (Table S3). These findings suggest that *GLTP* may be involved in skin development, muscle contraction, signal transduction, immune regulation, and other related processes.

### Tumor microenvironment (TME) landscape

**Figure 4:**
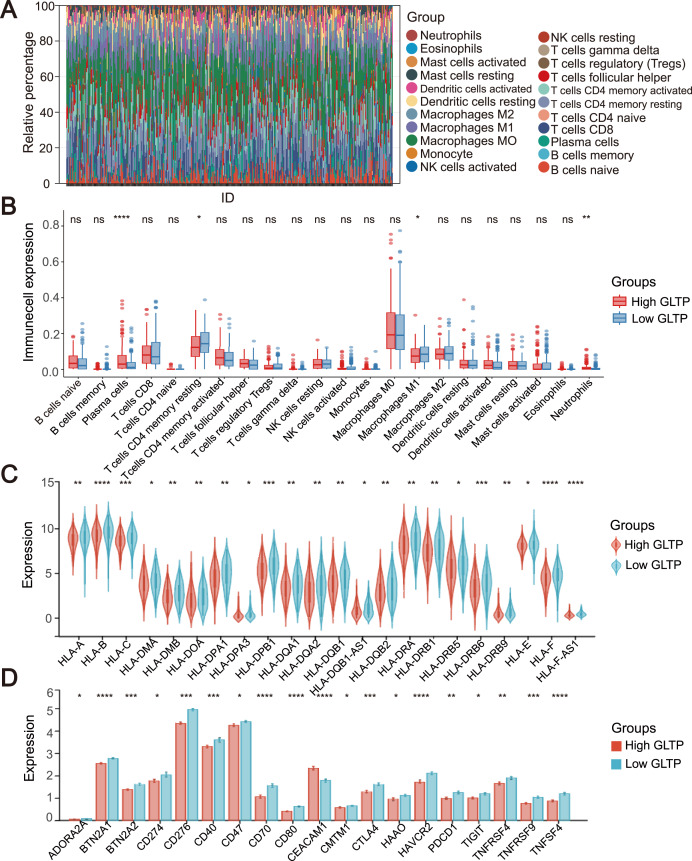
Immune characteristics of different *GLTP* expression groups. (A) Correlation of *GLTP* immunological score and stromal score in HNSCC. (B) Infiltration of 22 immune cells of HNSCC samples in low and high *GLTP* expression groups. (C) Differential expression of HLA genes in low and high *GLTP* expression groups in HNSCC. (D) Differential expression of immune checkpoint related genes in low and high *GLTP* expression groups in HNSCC. Ns, Not significant, *P < 0.05, **P < 0.01, ***P < 0.001, ****P < 0.0001. HNSCC, head and neck squamous cell carcinoma; TICs, tumor-infiltrating cells; HLA, human leukocyte antigen.

As studies on GLTP and tumors have progressed, it has been shown that GLTP is essential for the activation of particular anti-tumor immune responses (Shi et al., 2022; Xing, Tian & Jin, 2022). Thus, in the two *GLTP* expression groups, the current study investigated the immune-infiltrating TME composition of 22 different immune cell types (Fig. 4A). Comparison between the high and low GLTP expression groups revealed that the high-expression group exhibited increased infiltration levels of plasma cells, alongside decreased levels of resting CD4^+^ memory T cells and M1 macrophages (Fig. 4B). A statistically significant difference in neutrophil infiltration was also observed between groups (*P* = 0.01); however, the absolute levels were extremely low in both, limiting the biological significance of this finding (Fig. 4B). The high GLTP expression group showed slightly higher expression levels of HLA-related genes, but the limited separation between groups suggests uncertain biological relevance (Fig. 4C). In contrast, immune checkpoint genes were generally lower in the high-expression group (Fig. 4D).

### Prognostic model construction

**Figure 5:**
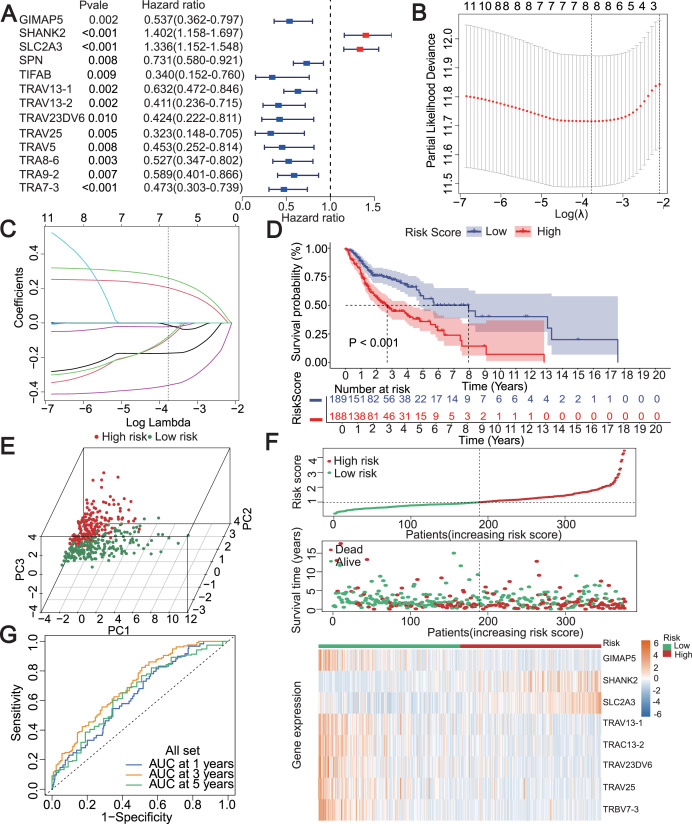
Construction of prognostic models. (A) Univariate Cox analysis assesses the prognostic value of DEGs in terms of OS. (B, C) LASSO-Cox analysis identified the five prognostic genes. (D) Kaplan-Meier survival analysis. (E) PCA of TCGA HNSCC cohort. (F) Risk score distribution, survival status, and heatmap of 8-genes prognostic signature in the TCGA HNSCC cohort. (G) ROC curve analysis of risk score on 1-, 3-, and 5-year survival status of HNSCC patients. DEGs, differentially expressed genes; TCGA, the Cancer Genome Atlas; HNSCC, head and neck squamous cell carcinoma; AUC, area under the curve; PCA, principal component analysis; ROC, receiver operating characteristic.

To identify prognostic genes in the TCGA HNSCC cohort, univariate Cox regression analysis was performed with DEG expression levels modeled as continuous variables. Genes with nominal *P* < 0.05 were retained as candidates, yielding 13 DEGs (Fig. 5A). To refine this gene set and reduce the risk of overfitting, LASSO-Cox regression with 10-fold cross-validation was subsequently applied. This process identified a final set of eight core genes that were used to construct the risk score model (Figs. 5B, 5C). The calculation formula was: risk score = −0.185 × GIMAP5 + 0.259 × SHANK2 + 0.317 × SLC2A3 − 0.024 × TRAV13-1 + 0.046 × TRAV13-2 − 0.266 × TRAV23DV6 − 0.267 × TRAV25 − 0.412 × TRBV7-3. The negative coefficients of *GIMAP5*, *TRAV13-1*, *TRAV23DV6*, *TRAV25*, and *TRBV7-3* indicate their roles as protective prognostic markers, whereas the positive coefficients of *SHANK2*, *SLC2A3*, and *TRAV13-2* identify them as risk-associated genes. Patients in the TCGA HNSCC cohort were successfully stratified as high-risk or low-risk based on their risk scores, with the median risk score serving as the cutoff. There were 189 patients in the low-risk category and 188 in the high-risk group. Kaplan-Meier survival analysis showed that patients in the low-risk group had a better prognosis than those in the high-risk group (Fig. 5D). The sample distributions of the two groups differed significantly, as further shown by PCA (Fig. 5E). Furthermore, a trend was observed wherein OS worsened considerably with increasing risk scores (Fig. 5F). Age, distant metastasis, and risk score were identified as independent prognostic factors with high significance for HNSCC patients (Table S4). Using ROC curve analysis, the model achieved AUC values of 0.622, 0.649, and 0.614 for 1-, 3-, and 5-year survival predictions, respectively, reflecting modest but relatively stable predictive performance over time (Fig. 5G). Compared with individual clinical variables, the risk score showed improved predictive capacity (Fig. S1).

### Validation of the prognostic model

**Figure 6:**
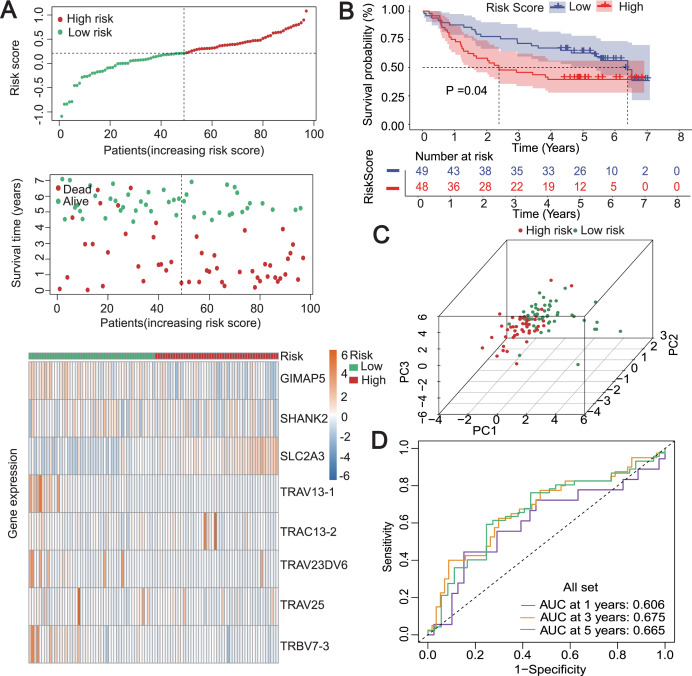
Validation of prognostic models (GSE41613). (A) Risk map of patients based on risk score heatmap of survival status and risk gene expression profiles of individual HNSCC patients. (B) Kaplan-Meier survival analysis. (C) PCA of GSE41613 cohort. (D) ROC curve analysis of risk score on 1-, 3-, and 5-year survival status of HNSCC patients. ROC, receiver operating characteristic; HNSCC, head and neck squamous cell carcinoma.

Based on the aforementioned risk scores, patients in the GSE41613 cohort were stratified into low-risk (*n* = 49) and high-risk (*n* = 48) groups (Fig. 6A). Kaplan-Meier survival analysis in this GEO validation set showed survival trends consistent with those observed in the TCGA cohort (Fig. 6B). PCA demonstrated distinct separation between the high-risk and low-risk groups in the GSE41613 cohort (Fig. 6C). Time-dependent ROC analysis yielded AUC values of 0.606, 0.675, and 0.665 for 1-, 3-, and 5-year survival predictions, further evaluating the predictive performance of the risk model (Figs. 6D, 6E) (Fig. S2).

### Independent prognosis, predictive value of risk scores and construction of nomogram based on the TCGA training set

**Figure 7:**
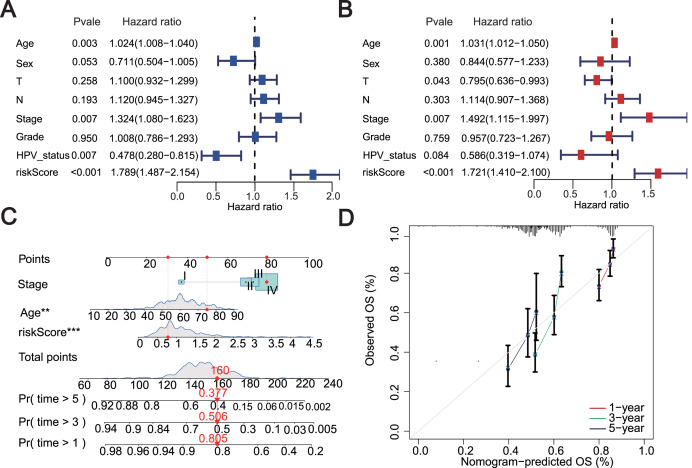
Prognostic values of the 8-gene signature model in the TCGA training set. (A) Univariate regression analysis obtains the prognostic value of clinical information and risk score in the TCGA training set. (B) Multivariate regression analysis obtains the prognostic value of clinical information and risk score in the TCGA training set. (C) Nomogram for prediction of 1-, 3-, and 5-year survival probabilities of HNSCC patients in the TCGA training set. (D) Calibration curve for assessing the agreement between nomogram predicted and actual survival outcomes in the TCGA training set. TCGA, the Cancer Genome Atlas; HNSCC, head and neck squamous cell carcinoma.

In this study, risk score, age, and clinical stage were identified as independent predictive markers for HNSCC patients and used to create the subsequent nomogram (Figs. 7A, 7B). Column line plots were used to estimate 1-, 3-, and 5-year survival rates for HNSCC patients (Fig. 7C). Calibration plots showed acceptable agreement between nomogram predictions and actual observations, with mean absolute errors of 0.073, 0.073, and 0.075 for 1-, 3-, and 5-year predictions, respectively, indicating that the nomogram was reasonably calibrated.

### Independent prognosis, predictive value of risk scores and construction of nomogram based on the GEO validation set

**Figure 8:**
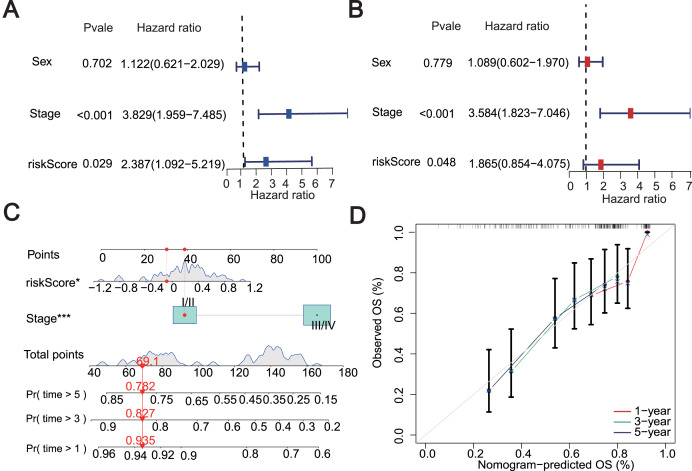
Prognostic values of the 8-gene signature model in the GSE41613 cohort. (A) Univariate regression analysis obtains the prognostic value of clinical information and risk score in the GSE41613 cohort. (B) Multivariate regression analysis obtains the prognostic value of clinical information and risk score in the GSE41613 cohort. (C) Nomogram for prediction of 1-, 3-, and 5-year survival probabilities of HNSCC patients in the GSE41613 cohort. (D) Calibration curve for assessing the agreement between nomogram predicted and actual survival outcomes in the GSE41613 cohort. HNSCC, head and neck squamous cell carcinoma.

To verify the model’s robustness, the aforementioned analysis was also carried out on the GEO validation set. In contrast to the TCGA training set, univariate and multivariate Cox regression analysis in the GEO validation set revealed that only risk score and clinical stage were independent prognostic markers for HNSCC patients (Figs. 8A, 8B). Therefore, a nomogram combining risk score and clinical stage was constructed to estimate 1-, 3-, and 5-year survival probabilities for HNSCC patients (Fig. 8C). The calibration curves demonstrated good agreement between predicted and observed survival outcomes, consistent with findings from the TCGA training set (Fig. 8D).

### *In vitro* functional validation of *GLTP* overexpression

**Figure 9:**
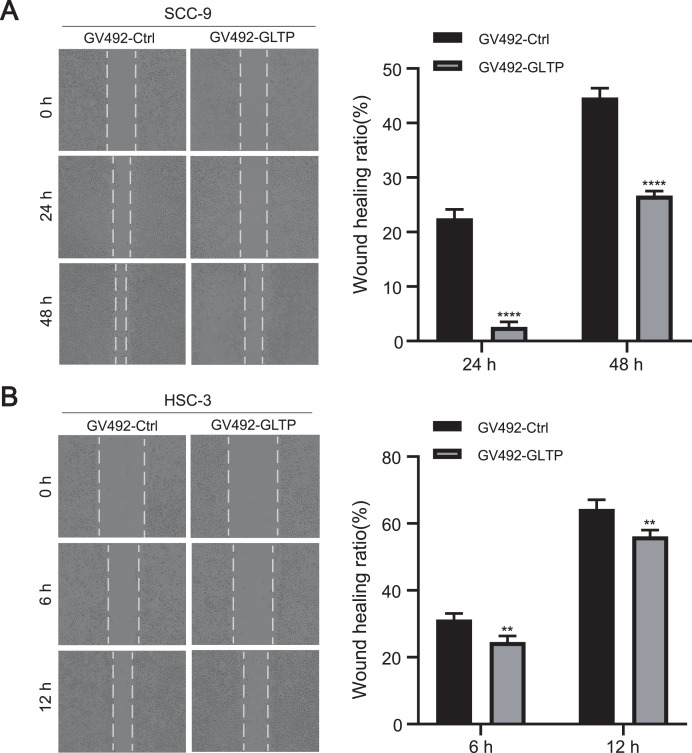
*GLTP* overexpression-mediated regulation of migration in HNSCC cells. (A) Wound-healing assay showing that overexpression of *GLTP* (GV492-GLTP) inhibited the migration of SCC-9 and (B) HSC-3 cells compared with the control group (GV492-Ctrl). **P < 0.01, ****P < 0.0001; *GLTP*, glycolipid transfer protein.

RT-qPCR confirmed the successful establishment of GLTP-overexpressing HSC-3 and SCC-9 cell lines (Fig. S3A). Wound healing assays demonstrated that overexpression of *GLTP* significantly suppressed the migratory capacity of both HSC-3 and SCC-9 cell lines compared to controls (Figs. 9A, 9B). This inhibitory effect on cell migration suggests that *GLTP* may play a role in regulating HNSCC cell motility.

### *In vitro* functional validation of *GLTP* knockdown

**Figure 10:**
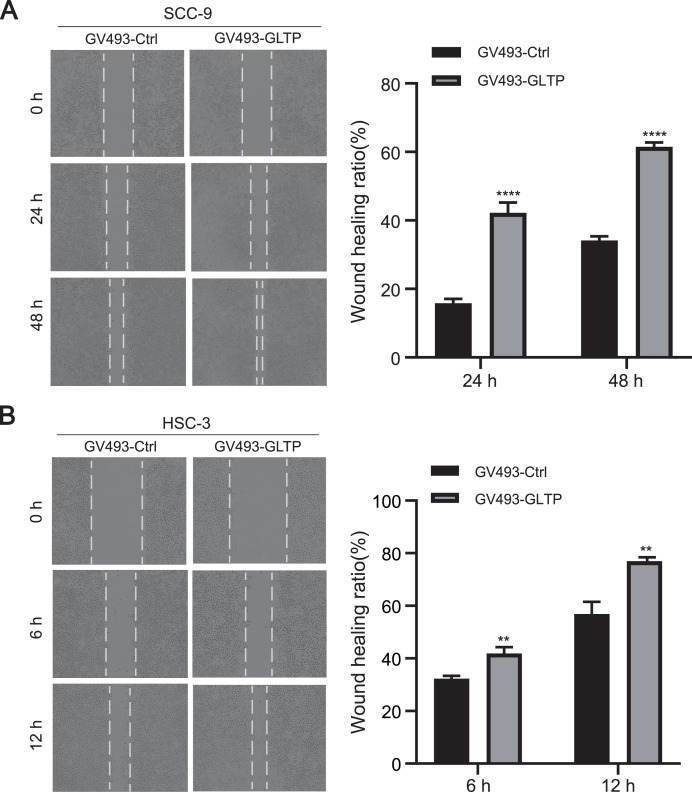
*GLTP* knockdown-mediated regulation of migration in HNSCC cells. (A) Knockdown of *GLTP* (GV493-GLTP) promoted the migration of SCC-9 and (B) HSC-3 cells compared with the control group (GV493-Ctrl). **P < 0.01, ****P < 0.0001; *GLTP*, glycolipid transfer protein.

RT-qPCR confirmed the successful establishment of GLTP-knockdown HSC-3 and SCC-9 cell lines (Fig. S3B). In contrast, knockdown of *GLTP* markedly enhanced the migratory ability of HSC-3 and SCC-9 cells relative to their respective controls (Figs. 10A, 10B). Together with the overexpression results, these findings indicate that *GLTP* modulates HNSCC cell migration, which may have implications for tumor progression.

## Discussions

Transcriptome analysis revealed significant downregulation of *GLTP* mRNA expression in HNSCC samples compared to normal tissues across multiple clinical subgroups (Fig. 1A). This downregulation was more pronounced in patients with advanced stage (III/IV) disease (Fig. 1B). Sex-stratified analysis showed that *GLTP* mRNA levels were lower in male patients than in females (Fig. 1C). Age-related analysis indicated significantly lower *GLTP* mRNA expression in patients aged 41–80 years compared to younger individuals (Fig. 1D). *GLTP* mRNA expression was also decreased in grade 3 tumors relative to lower-grade tumors (Fig. 1E). Spearman correlation analysis (|ρ| > 0.5) identified 241 genes significantly co-expressed with *GLTP*. Protein-protein interaction network analysis defined the top ten hub proteins, which included several members of the SPRR family, as well as IVL, LOR, FLG, RPTN, TGM1, and PKP1 (Fig. 1F). PPI revealed that the proteins encoded by these epidermal differentiation complex (EDC) genes interacted closely with one another and were all engaged in the process of keratinocyte development. These findings align with previous reports implicating *GLTP* in epidermal homeostasis. Earlier studies have shown that GLTP plays a crucial role in epidermal healing of genetic skin disorders and regulates human keratinocyte differentiation (Zhang et al., 2021). Furthermore, *GLTP* has emerged as a potential therapeutic target for keloid treatment (Song et al., 2023), suggesting its broader significance in cutaneous pathophysiology. It is noteworthy that several of the hub genes identified in this study have critical regulatory functions in the onset and development of various malignant tumors. Members of the SPRR family frequently show pro-oncogenic characteristics in tumors. For instance, SPRR1B has been identified as a novel potential biomarker for liver metastasis in pancreatic ductal adenocarcinoma (Zhang et al., 2020) and stimulates tumor growth by activating the MAPK pathway in head and neck squamous cell carcinoma (Sasahira et al., 2021); SPRR1A, SPRR1B, and SPRR2B may speed up melanoma growth *via* the MEK/ATF2/EGFR signaling axis (Barbero et al., 2022). Additionally, abnormal EDC expression has demonstrated important clinical implications: decreased FLG expression is linked to a higher risk of cutaneous squamous cell carcinoma (cSCC)/basal cell carcinoma (BCC) and the advancement of esophageal squamous cell carcinoma (Vanderbeck et al., 2021; Zhong et al., 2019), while downregulated IVL expression is linked to a poor prognosis of oral tongue squamous cell carcinoma (OTSCC) (Pandey et al., 2021). Although the precise molecular mechanisms remain to be elucidated, our findings suggest that *GLTP* and its co-expressed EDC-related genes may play important roles in tumor development and progression.

Utilizing transcriptome data from the TCGA HNSCC cohort, patients were divided into high and low-expression groups based on *GLTP* expression levels, resulting in the identification of 5,464 DEGs (Figs. 2A, 2B). GO functional enrichment analysis demonstrated that DEGs are primarily involved in biological processes such as ‘muscle system process’ and ‘skin development’ (Fig 3A). KEGG pathway analysis revealed that these DEGs were associated with ‘cytokine-cytokine receptor interaction’, ‘Ras signaling pathway’ and other pathways (Fig. 3B). Additionally, GSEA showed that DEGs were considerably enriched in pathways like ‘Chemokine signaling route’ and ‘PI3K-Akt signaling pathway’ (Table S3), as well as pathways like ‘Arachidonic acid metabolism’ (Fig. 3C). All of these findings point to GLTP’s potential involvement in a variety of bodily functions, including immune regulation, muscle contraction, signal transduction, skin development, and more. In general, HNSCC tumors exhibit a high degree of immune cell infiltration. Immune infiltration analysis revealed that GLTP expression correlates with distinct immune characteristics in the HNSCC microenvironment. High GLTP expression was associated with increased plasma cell infiltration, whereas low GLTP expression corresponded with higher levels of resting CD4^+^ memory T cells and M1 macrophages (Fig. 4B). Although neutrophil infiltration reached statistical significance between groups, the low absolute levels across all samples limit the biological relevance of this observation (Fig. 4B). HLA family genes showed marginally higher expression in the high GLTP group, yet the limited separation between groups leaves the functional implications uncertain (Fig. 4C). More notably, immune checkpoint genes trended lower in patients with high GLTP expression (Fig. 4D). Together, these findings suggest a potential role for GLTP in modulating immune activity in HNSCC.

In this study, a risk score model was constructed based on eight key genes (*GIMAP5*, *SHANK2*, *SLC2A3*, *TRAV13-1*, *TRAV13-2*, *TRAV23DV6*, *TRAV25*, and *TRBV7-3*) (Figs. 5A–5C). Of particular note is the inclusion of several T-cell receptor-related genes (*e.g*., *TRAV13-1*, *TRAV23DV6*, and *TRBV7-3*) within the model. The negative coefficients of these genes identify them as protective prognostic markers, which aligns with previous findings that higher T-cell receptor diversity is associated with enhanced anti-tumor immunity (Tanaka et al., 2025). However, it is important to acknowledge that the expression levels of these TCR-related genes, as detected in bulk tumor samples, may reflect the abundance of tumor-infiltrating lymphocytes rather than tumor-intrinsic gene expression. Thus, their protective prognostic value could be partially attributed to the extent of T-cell infiltration within the tumor microenvironment, highlighting the role of adaptive immunity in HNSCC progression. The negative coefficient of *GIMAP5* in the model identifies it as a protective prognostic marker, consistent with its known role in T-cell homeostasis (Park et al., 2024), whereas the positive coefficient of *SLC2A3* defines it as a risk-associated gene, supporting previous evidence that its upregulation promotes glycolysis and suppresses antitumor immunity (Jiang et al., 2024; Yao et al., 2020). These results offer fresh perspectives on the immunological escape mechanism of HNSCC. As shown by PCA visualization, samples from the high-risk and low-risk groups had significant spatial distribution differences in gene expression profiles (Fig. 5E), confirming the reliability of risk stratification at the molecular level. The high-risk group had a significantly worse clinical prognosis when split by the median risk score (Figs. 5D, 5F). Time-dependent ROC analysis demonstrated that the model achieved sustained predictive performance at 1 year (AUC = 0.622), 3 years (AUC = 0.649), and 5 years (AUC = 0.614) (Fig. 5G). This risk score also exceeded conventional clinical indicators in terms of prediction efficacy (Fig. S1). The robustness and reproducibility of the model were further validated in an independent validation cohort (GSE41613), which demonstrated consistent prognostic discriminatory ability with the training set (Figs. 6A–6D and S2). In the TCGA training cohort, univariable and multivariable Cox regression analyses incorporating clinicopathological factors and HPV status were performed to evaluate independent prognostic factors (Figs. 7A, 7B). In univariable analysis, age, stage, HPV status, and risk score were significantly associated with overall survival (Fig. 7A). In multivariable analysis, age, T stage, stage, and risk score remained independent prognostic factors, while HPV status did not reach statistical significance (*P* = 0.084) (Fig. 7B). The loss of significance for HPV status in multivariable analysis suggests that its prognostic effect may be partially captured by the risk score. Notably, the independent validation cohort GSE41613 consists exclusively of HPV-negative patients, and in this cohort our risk score also demonstrated significant prognostic value (Figs. 8A, 8B). Collectively, these analyses indicate that the predictive performance of our risk model is independent of HPV status. Nomograms integrating independent prognostic markers in both the training and validation sets demonstrated good calibration in predicting 1-, 3-, and 5-year survival rates (Figs. 7C, 7D and 8C, 8D), suggesting their potential utility. Wound healing assays conducted in HSC-3 and SCC-9 cell lines demonstrated that *GLTP* overexpression significantly suppressed cellular migration, whereas *GLTP* knockdown markedly enhanced this malignant phenotype (Figs. 9A, 9B and 10A, 10B). These results suggest that *GLTP* may modulate cell motility, offering a possible biological basis for its role in shaping the gene expression profile that underlies the prognostic stratification of our risk model.

However, this study has several limitations. First, as the analysis was based on retrospective data from public platforms, it may be subject to update delays or inherent sample bias. Second, the functional role of GLTP was investigated only in the context of migration, and the molecular mechanisms responsible for this phenotype have yet to be elucidated. Although serum-free medium was used during the wound healing assay, a minor effect on cell growth cannot be completely excluded without the use of mitomycin C. Third, incomplete survival and clinicopathological information in the TCGA cohort, as well as a lack of detailed staging in the GSE41613 set, somewhat restricted the model’s comprehensive validation. Future prospective studies with expanded experimental validation are needed to reinforce the applicability of our findings.

## Conclusions

This study systematically investigated *GLTP* expression in HNSCC, its associated protein interaction network, potential molecular pathways, and immune infiltration profile. A *GLTP*-related prognostic model for HNSCC was developed and validated. The risk score was evaluated as an independent prognostic factor, and a nomogram incorporating clinical variables was constructed. Functional experiments using wound healing assays demonstrated that GLTP overexpression inhibited HNSCC cell migration, while GLTP knockdown enhanced migration. These findings indicate that *GLTP* may serve as a potential biomarker for HNSCC prognosis and provide preliminary insights into its biological function.

## Supplemental Information

10.7717/peerj.21611/supp-1 Supplemental Information 1 RT-qPCR raw data.

10.7717/peerj.21611/supp-2 Supplemental Information 2 The AUC of the clinical information and risk score in TCGA HNSCC cohort.

10.7717/peerj.21611/supp-3 Supplemental Information 3 The AUC of the clinical information and risk score in GSE41613 cohort.

10.7717/peerj.21611/supp-4 Supplemental Information 4 Validation of GLTP overexpression and knockdown in HSC-3 and SCC-9 cell lines by RT-qPCR.

10.7717/peerj.21611/supp-5 Supplemental Information 5 Classification of TCGA HNSCC samples based on the expression levels of *GLTP*. High, high *GLTP* expression group; Low, low *GLTP* expression group.

10.7717/peerj.21611/supp-6 Supplemental Information 6 DEGs between low and high *GLTP* expression groups.

10.7717/peerj.21611/supp-7 Supplemental Information 7 The pathways of DEGs identified through GSEA in the TCGA-HNSCC cohort. DEG, differentially expressed genes; TCGA, the Cancer Genome Atlas; HNSCC, head and neck squamous cell carcinoma; NES, normalized enrichment score; FDR, false discovery rate.

10.7717/peerj.21611/supp-8 Supplemental Information 8 Clinicopathological characteristics of the patients in the TCGA HNSCC cohort and the GSE41613 cohort.

10.7717/peerj.21611/supp-9 Supplemental Information 9 MIQE checklist.

## Additional Information and Declarations

### Data Availability

The following information was supplied regarding data availability:

The data is available in the Supplemental Files.

The code is available at: luu919. (2025). luu919/GLTP-HNSCC-Prognostic-Mode: Code Release for PeerJ Manuscript—GLTP in HNSCC (Manuscript_Submission). Zenodo. https://doi.org/10.5281/zenodo.17973402

The raw data from the cell wound healing (scratch) assays are available at: Zeng, Lu (2025). Predictive and mechanistic insights of GLTP on survival in patients with head and neck squamous cell carcinomaem. figshare. Dataset. https://doi.org/10.6084/m9.figshare.30931355.v1.
