# Supplementary material for: Predictive and mechanistic insights of GLTP on survival in patients with head and neck squamous cell carcinoma
Source: PeerJ. 2026 Aug 5;14:e21611. doi: 10.7717/peerj.21611 (PMC13452426; doi:10.7717/peerj.21611)
Supplement: Supplementary file 1 [file peerj-14-21611-s005.docx]

**Table S1. Classification of TCGA HNSCC samples based on the expression levels of *GLTP*. High, high *GLTP* expression group; Low, low *GLTP* expression group.**

| **Samples** | **Groups** | ***GLTP*** Expression |
| --- | --- | --- |
| TCGA-BB-4228-01 | Low | 5.170 |
| TCGA-UF-A7JH-01 | Low | 5.996 |
| TCGA-IQ-A6SH-01 | Low | 5.143 |
| TCGA-CV-5430-01 | Low | 5.028 |
| TCGA-CR-6481-01 | Low | 6.032 |
| TCGA-CV-6942-01 | Low | 4.352 |
| TCGA-CN-A498-01 | Low | 6.039 |
| TCGA-F7-A50I-01 | Low | 5.197 |
| TCGA-CN-6989-01 | Low | 5.485 |
| TCGA-F7-A624-01 | Low | 4.891 |
| TCGA-UF-A718-01 | Low | 6.242 |
| TCGA-CV-5978-01 | Low | 6.232 |
| TCGA-BA-6868-01 | Low | 4.977 |
| TCGA-TN-A7HL-01 | Low | 6.151 |
| TCGA-CV-7410-01 | Low | 5.229 |
| TCGA-D6-6826-01 | Low | 5.536 |
| TCGA-CV-7429-01 | Low | 4.871 |
| TCGA-CQ-6224-01 | Low | 4.104 |
| TCGA-CR-6484-01 | Low | 6.264 |
| TCGA-CV-A6JZ-01 | Low | 5.298 |
| TCGA-CR-6488-01 | Low | 6.094 |
| TCGA-CV-6937-01 | Low | 5.332 |
| TCGA-P3-A6T0-01 | Low | 5.189 |
| TCGA-T2-A6WX-01 | Low | 4.139 |
| TCGA-CR-6478-01 | Low | 4.787 |
| TCGA-CV-7421-01 | Low | 5.642 |
| TCGA-CQ-A4C7-01 | Low | 4.823 |
| TCGA-CR-7404-01 | Low | 6.056 |
| TCGA-CR-7370-01 | Low | 5.883 |
| TCGA-BA-A6DG-01 | Low | 5.864 |
| TCGA-CR-7382-01 | Low | 4.605 |
| TCGA-CV-6950-01 | Low | 4.699 |
| TCGA-CV-6943-01 | Low | 6.057 |
| TCGA-BA-4078-01 | Low | 6.287 |
| TCGA-BB-8596-01 | Low | 4.862 |
| TCGA-BA-A6D8-01 | Low | 5.778 |
| TCGA-CV-5976-01 | Low | 5.479 |
| TCGA-CV-5440-01 | Low | 5.805 |
| TCGA-BB-A5HY-01 | Low | 5.206 |
| TCGA-UF-A7JS-01 | Low | 5.127 |
| TCGA-CV-A6K0-01 | Low | 6.034 |
| TCGA-BB-4225-01 | Low | 5.784 |
| TCGA-CQ-A4CG-01 | Low | 4.711 |
| TCGA-CV-6948-01 | Low | 6.213 |
| TCGA-CV-7103-01 | Low | 6.223 |
| TCGA-CV-7100-01 | Low | 5.630 |
| TCGA-CV-7416-01 | Low | 5.033 |
| TCGA-HD-7832-01 | Low | 5.237 |
| TCGA-CV-5443-01 | Low | 6.044 |
| TCGA-T3-A92M-01 | Low | 5.342 |
| TCGA-CV-7568-01 | Low | 5.521 |
| TCGA-D6-6517-01 | Low | 5.506 |
| TCGA-P3-A5QE-01 | Low | 5.669 |
| TCGA-CN-5361-01 | Low | 5.156 |
| TCGA-CN-6022-01 | Low | 4.456 |
| TCGA-CR-6467-01 | Low | 6.026 |
| TCGA-CN-6024-01 | Low | 5.980 |
| TCGA-CV-7415-01 | Low | 5.809 |
| TCGA-CV-5444-01 | Low | 4.934 |
| TCGA-MZ-A7D7-01 | Low | 6.185 |
| TCGA-CN-4735-01 | Low | 5.224 |
| TCGA-CN-A642-01 | Low | 4.636 |
| TCGA-CV-A45Y-01 | Low | 6.257 |
| TCGA-IQ-A61G-01 | Low | 6.078 |
| TCGA-BB-A5HU-01 | Low | 5.186 |
| TCGA-DQ-7592-01 | Low | 6.295 |
| TCGA-HD-7753-01 | Low | 5.295 |
| TCGA-CV-7102-01 | Low | 4.922 |
| TCGA-CV-7406-01 | Low | 6.309 |
| TCGA-CQ-A4CH-01 | Low | 5.637 |
| TCGA-HL-7533-01 | Low | 4.456 |
| TCGA-IQ-A61E-01 | Low | 5.769 |
| TCGA-HD-8635-01 | Low | 5.016 |
| TCGA-CR-6477-01 | Low | 5.912 |
| TCGA-CV-5436-01 | Low | 4.801 |
| TCGA-CV-7091-01 | Low | 5.622 |
| TCGA-CV-6934-01 | Low | 5.660 |
| TCGA-DQ-5624-01 | Low | 5.913 |
| TCGA-CV-7433-01 | Low | 6.270 |
| TCGA-P3-A5QA-01 | Low | 4.278 |
| TCGA-CV-7418-01 | Low | 5.693 |
| TCGA-CN-A49A-01 | Low | 5.898 |
| TCGA-UF-A7JT-01 | Low | 6.033 |
| TCGA-DQ-7591-01 | Low | 6.159 |
| TCGA-WA-A7H4-01 | Low | 4.813 |
| TCGA-CR-7399-01 | Low | 5.948 |
| TCGA-IQ-7630-01 | Low | 5.313 |
| TCGA-CR-6473-01 | Low | 5.917 |
| TCGA-CN-4723-01 | Low | 5.342 |
| TCGA-CQ-A4C6-01 | Low | 5.554 |
| TCGA-QK-A6VB-01 | Low | 6.253 |
| TCGA-CN-6013-01 | Low | 6.007 |
| TCGA-BA-5153-01 | Low | 6.134 |
| TCGA-CR-7377-01 | Low | 5.813 |
| TCGA-CR-7398-01 | Low | 5.735 |
| TCGA-CV-7437-01 | Low | 5.945 |
| TCGA-KU-A66S-01 | Low | 5.326 |
| TCGA-CN-6997-01 | Low | 5.038 |
| TCGA-CV-5434-01 | Low | 6.031 |
| TCGA-CR-7376-01 | Low | 4.951 |
| TCGA-KU-A66T-01 | Low | 5.540 |
| TCGA-CV-7178-01 | Low | 4.455 |
| TCGA-CN-4738-01 | Low | 5.795 |
| TCGA-F7-A620-01 | Low | 6.003 |
| TCGA-IQ-A61O-01 | Low | 4.069 |
| TCGA-QK-A8Z9-01 | Low | 4.357 |
| TCGA-CV-6959-01 | Low | 5.596 |
| TCGA-CV-6935-01 | Low | 5.036 |
| TCGA-P3-A5Q5-01 | Low | 5.863 |
| TCGA-UF-A7JV-01 | Low | 4.613 |
| TCGA-CV-6003-01 | Low | 4.846 |
| TCGA-CV-5431-01 | Low | 5.238 |
| TCGA-P3-A6T3-01 | Low | 5.945 |
| TCGA-P3-A6SX-01 | Low | 5.754 |
| TCGA-BA-4074-01 | Low | 5.668 |
| TCGA-CN-A6V3-01 | Low | 6.112 |
| TCGA-D6-6515-01 | Low | 5.758 |
| TCGA-CN-4733-01 | Low | 4.016 |
| TCGA-CN-5365-01 | Low | 6.230 |
| TCGA-KU-A6H8-01 | Low | 5.955 |
| TCGA-QK-A64Z-01 | Low | 4.863 |
| TCGA-DQ-5630-01 | Low | 5.787 |
| TCGA-CQ-A4CD-01 | Low | 4.835 |
| TCGA-CR-6482-01 | Low | 5.421 |
| TCGA-CV-6961-01 | Low | 4.670 |
| TCGA-DQ-5629-01 | Low | 5.514 |
| TCGA-CV-7097-01 | Low | 5.622 |
| TCGA-D6-6827-01 | Low | 5.236 |
| TCGA-F7-8298-01 | Low | 4.712 |
| TCGA-CV-7255-01 | Low | 4.972 |
| TCGA-CV-7236-01 | Low | 5.219 |
| TCGA-CQ-A4CA-01 | Low | 5.390 |
| TCGA-HD-8314-01 | Low | 6.274 |
| TCGA-CN-6995-01 | Low | 5.990 |
| TCGA-CN-6996-01 | Low | 6.233 |
| TCGA-CQ-A4CB-01 | Low | 4.810 |
| TCGA-CR-7383-01 | Low | 5.164 |
| TCGA-HD-7229-01 | Low | 5.438 |
| TCGA-BB-4223-01 | Low | 6.186 |
| TCGA-CV-5979-01 | Low | 5.107 |
| TCGA-MT-A7BN-01 | Low | 4.677 |
| TCGA-CQ-7068-01 | Low | 5.212 |
| TCGA-CN-5370-01 | Low | 5.595 |
| TCGA-CV-7238-01 | Low | 5.743 |
| TCGA-CN-A6UY-01 | Low | 5.897 |
| TCGA-BB-4227-01 | Low | 5.952 |
| TCGA-MZ-A5BI-01 | Low | 5.872 |
| TCGA-BA-6871-01 | Low | 4.957 |
| TCGA-CR-5243-01 | Low | 6.115 |
| TCGA-CV-7247-01 | Low | 6.087 |
| TCGA-CX-7219-01 | Low | 6.061 |
| TCGA-CN-6023-01 | Low | 5.288 |
| TCGA-H7-A76A-01 | Low | 5.943 |
| TCGA-CV-A464-01 | Low | 4.946 |
| TCGA-HD-8224-01 | Low | 4.688 |
| TCGA-CV-5435-01 | Low | 5.924 |
| TCGA-BA-A8YP-01 | Low | 5.087 |
| TCGA-QK-AA3K-01 | Low | 3.767 |
| TCGA-RS-A6TP-01 | Low | 6.158 |
| TCGA-HD-A634-01 | Low | 4.089 |
| TCGA-CN-A63V-01 | Low | 5.606 |
| TCGA-UF-A71B-01 | Low | 5.765 |
| TCGA-QK-A6IF-01 | Low | 5.318 |
| TCGA-UF-A7JA-01 | Low | 5.414 |
| TCGA-CV-A460-01 | Low | 5.734 |
| TCGA-CQ-7072-01 | Low | 4.566 |
| TCGA-CN-5367-01 | Low | 4.932 |
| TCGA-P3-A6T8-01 | Low | 4.955 |
| TCGA-BA-6870-01 | Low | 5.201 |
| TCGA-CN-5366-01 | Low | 5.359 |
| TCGA-IQ-A61J-01 | Low | 6.160 |
| TCGA-CN-4736-01 | Low | 5.778 |
| TCGA-CN-5356-01 | Low | 6.049 |
| TCGA-UF-A7JD-01 | Low | 4.449 |
| TCGA-CV-7438-01 | Low | 4.862 |
| TCGA-KU-A6H7-01 | Low | 5.934 |
| TCGA-CN-A497-01 | Low | 5.931 |
| TCGA-P3-A6T5-01 | Low | 4.997 |
| TCGA-CR-7374-01 | Low | 6.281 |
| TCGA-CR-7392-01 | Low | 5.981 |
| TCGA-BB-4217-01 | Low | 6.131 |
| TCGA-CV-6936-01 | Low | 4.963 |
| TCGA-IQ-7631-01 | Low | 5.178 |
| TCGA-CX-A4AQ-01 | Low | 5.226 |
| TCGA-BA-A4IH-01 | Low | 6.096 |
| TCGA-CV-5971-01 | Low | 4.579 |
| TCGA-CN-5359-01 | Low | 5.989 |
| TCGA-CV-7254-01 | Low | 5.578 |
| TCGA-CV-5442-01 | Low | 5.479 |
| TCGA-4P-AA8J-01 | Low | 5.684 |
| TCGA-D6-6824-01 | Low | 5.437 |
| TCGA-CV-7414-01 | Low | 5.942 |
| TCGA-CR-6470-01 | Low | 5.760 |
| TCGA-P3-A6T2-01 | Low | 4.994 |
| TCGA-CN-4728-01 | Low | 5.144 |
| TCGA-CV-6939-01 | Low | 5.937 |
| TCGA-CN-A499-01 | Low | 6.202 |
| TCGA-CR-7373-01 | Low | 5.266 |
| TCGA-CR-6487-01 | Low | 5.390 |
| TCGA-WA-A7GZ-01 | Low | 5.647 |
| TCGA-D6-8568-01 | Low | 5.635 |
| TCGA-D6-A6EP-01 | Low | 4.261 |
| TCGA-CR-5249-01 | Low | 6.200 |
| TCGA-CR-6474-01 | Low | 4.337 |
| TCGA-DQ-5631-01 | Low | 4.635 |
| TCGA-CN-5374-01 | Low | 6.036 |
| TCGA-CV-6433-01 | Low | 5.674 |
| TCGA-CV-6945-01 | High | 6.704 |
| TCGA-CN-5358-01 | High | 7.561 |
| TCGA-CR-7397-01 | High | 6.460 |
| TCGA-TN-A7HI-01 | High | 6.587 |
| TCGA-CV-7428-01 | High | 6.982 |
| TCGA-CV-A461-01 | High | 7.274 |
| TCGA-CQ-A4CI-01 | High | 6.572 |
| TCGA-BA-A4IG-01 | High | 6.548 |
| TCGA-D6-A4Z9-01 | High | 6.620 |
| TCGA-CV-A468-01 | High | 7.020 |
| TCGA-CV-6933-01 | High | 6.805 |
| TCGA-F7-A623-01 | High | 7.773 |
| TCGA-QK-A8Z8-01 | High | 8.558 |
| TCGA-T2-A6X0-01 | High | 6.520 |
| TCGA-UF-A719-01 | High | 7.732 |
| TCGA-CQ-5331-01 | High | 6.910 |
| TCGA-CR-7368-01 | High | 6.728 |
| TCGA-CN-6992-01 | High | 6.830 |
| TCGA-CR-7364-01 | High | 8.051 |
| TCGA-UF-A7JK-01 | High | 6.369 |
| TCGA-CR-6492-01 | High | 7.340 |
| TCGA-BA-A4IF-01 | High | 7.025 |
| TCGA-CV-5441-01 | High | 8.195 |
| TCGA-C9-A47Z-01 | High | 7.945 |
| TCGA-CV-7101-01 | High | 6.371 |
| TCGA-CR-7388-01 | High | 6.748 |
| TCGA-QK-A6VC-01 | High | 6.372 |
| TCGA-CQ-6222-01 | High | 6.340 |
| TCGA-D6-6825-01 | High | 6.899 |
| TCGA-CQ-5326-01 | High | 6.868 |
| TCGA-CR-6491-01 | High | 6.714 |
| TCGA-CV-7413-01 | High | 6.793 |
| TCGA-HD-7754-01 | High | 6.530 |
| TCGA-MT-A67D-01 | High | 7.298 |
| TCGA-P3-A6T7-01 | High | 7.316 |
| TCGA-HD-A4C1-01 | High | 7.577 |
| TCGA-CV-7427-01 | High | 7.848 |
| TCGA-CN-A6V6-01 | High | 7.056 |
| TCGA-UF-A7JO-01 | High | 6.687 |
| TCGA-CQ-6228-01 | High | 6.775 |
| TCGA-CV-7099-01 | High | 6.679 |
| TCGA-CN-6994-01 | High | 6.710 |
| TCGA-CN-5369-01 | High | 6.853 |
| TCGA-CV-7263-01 | High | 7.687 |
| TCGA-BA-A6DL-01 | High | 8.508 |
| TCGA-F7-7848-01 | High | 6.717 |
| TCGA-CV-6954-01 | High | 7.016 |
| TCGA-CV-7248-01 | High | 7.105 |
| TCGA-CN-A641-01 | High | 8.877 |
| TCGA-UP-A6WW-01 | High | 6.571 |
| TCGA-CV-7424-01 | High | 6.673 |
| TCGA-CN-4737-01 | High | 6.734 |
| TCGA-QK-A8ZA-01 | High | 7.030 |
| TCGA-CR-7365-01 | High | 7.154 |
| TCGA-CQ-A4CE-01 | High | 8.461 |
| TCGA-CR-7389-01 | High | 6.366 |
| TCGA-QK-A8ZB-01 | High | 7.535 |
| TCGA-CQ-A4C9-01 | High | 7.730 |
| TCGA-CV-7440-01 | High | 7.046 |
| TCGA-D6-6823-01 | High | 6.641 |
| TCGA-CR-6471-01 | High | 6.409 |
| TCGA-C9-A480-01 | High | 6.664 |
| TCGA-CR-7379-01 | High | 6.930 |
| TCGA-UF-A7J9-01 | High | 6.589 |
| TCGA-BA-5151-01 | High | 7.562 |
| TCGA-MZ-A6I9-01 | High | 6.689 |
| TCGA-HD-A6HZ-01 | High | 6.698 |
| TCGA-CX-7082-01 | High | 6.913 |
| TCGA-CV-7435-01 | High | 7.427 |
| TCGA-CN-4739-01 | High | 7.881 |
| TCGA-CX-7086-01 | High | 7.819 |
| TCGA-CN-6010-01 | High | 6.343 |
| TCGA-CV-A45X-01 | High | 7.968 |
| TCGA-BB-A5HZ-01 | High | 7.940 |
| TCGA-CN-A63T-01 | High | 8.094 |
| TCGA-CN-A63W-01 | High | 6.971 |
| TCGA-CN-6998-01 | High | 6.510 |
| TCGA-D6-A6EO-01 | High | 8.454 |
| TCGA-CN-5355-01 | High | 6.581 |
| TCGA-CV-6962-01 | High | 7.485 |
| TCGA-CV-6941-01 | High | 7.722 |
| TCGA-CR-7390-01 | High | 6.431 |
| TCGA-CN-4729-01 | High | 7.335 |
| TCGA-CV-A6JY-01 | High | 8.234 |
| TCGA-CV-5970-01 | High | 7.510 |
| TCGA-CV-A45R-01 | High | 7.302 |
| TCGA-CN-4731-01 | High | 8.087 |
| TCGA-UF-A7JJ-01 | High | 6.422 |
| TCGA-CN-6019-01 | High | 6.769 |
| TCGA-D6-A74Q-01 | High | 7.267 |
| TCGA-CN-6012-01 | High | 6.999 |
| TCGA-IQ-A6SG-01 | High | 7.164 |
| TCGA-CN-A63U-01 | High | 6.613 |
| TCGA-CV-A465-01 | High | 8.192 |
| TCGA-CV-A45W-01 | High | 7.107 |
| TCGA-IQ-A61H-01 | High | 7.131 |
| TCGA-CR-7402-01 | High | 7.032 |
| TCGA-CR-6480-01 | High | 6.399 |
| TCGA-CV-A45P-01 | High | 7.811 |
| TCGA-CV-7242-01 | High | 8.469 |
| TCGA-CQ-5327-01 | High | 7.723 |
| TCGA-BA-A6DE-01 | High | 6.856 |
| TCGA-CR-6472-01 | High | 6.419 |
| TCGA-CV-A45V-01 | High | 8.591 |
| TCGA-CR-7372-01 | High | 8.512 |
| TCGA-CQ-6227-01 | High | 6.713 |
| TCGA-CV-7261-01 | High | 6.477 |
| TCGA-CR-5250-01 | High | 6.387 |
| TCGA-BA-5555-01 | High | 6.396 |
| TCGA-CV-A45Z-01 | High | 8.188 |
| TCGA-BB-A6UM-01 | High | 7.031 |
| TCGA-CN-5360-01 | High | 7.538 |
| TCGA-CN-4722-01 | High | 7.611 |
| TCGA-RS-A6TO-01 | High | 6.698 |
| TCGA-CV-7422-01 | High | 7.191 |
| TCGA-CN-6020-01 | High | 6.324 |
| TCGA-TN-A7HJ-01 | High | 7.371 |
| TCGA-CV-A45U-01 | High | 6.673 |
| TCGA-CV-A45T-01 | High | 6.454 |
| TCGA-P3-A6SW-01 | High | 7.561 |
| TCGA-CV-6956-01 | High | 7.058 |
| TCGA-HD-A633-01 | High | 7.244 |
| TCGA-CX-7085-01 | High | 7.354 |
| TCGA-CR-7369-01 | High | 6.556 |
| TCGA-BA-A4II-01 | High | 7.780 |
| TCGA-CV-6960-01 | High | 7.056 |
| TCGA-BA-A6DI-01 | High | 6.432 |
| TCGA-BA-5152-01 | High | 7.605 |
| TCGA-CR-7391-01 | High | 7.005 |
| TCGA-IQ-A61I-01 | High | 7.224 |
| TCGA-CN-6988-01 | High | 8.484 |
| TCGA-CV-A6JT-01 | High | 6.439 |
| TCGA-CV-A6JU-01 | High | 7.023 |
| TCGA-CN-A6V7-01 | High | 6.581 |
| TCGA-BA-5559-01 | High | 6.560 |
| TCGA-UF-A7JF-01 | High | 7.362 |
| TCGA-UF-A71E-01 | High | 7.476 |
| TCGA-CV-7250-01 | High | 9.320 |
| TCGA-P3-A6T4-01 | High | 7.637 |
| TCGA-CV-A6K2-01 | High | 6.948 |
| TCGA-QK-A652-01 | High | 6.490 |
| TCGA-H7-A6C4-01 | High | 7.753 |
| TCGA-F7-A50G-01 | High | 7.492 |
| TCGA-QK-A6V9-01 | High | 6.736 |
| TCGA-CQ-7071-01 | High | 7.355 |
| TCGA-D6-A4ZB-01 | High | 7.695 |
| TCGA-CN-4725-01 | High | 6.558 |
| TCGA-QK-AA3J-01 | High | 6.812 |
| TCGA-BB-4224-01 | High | 7.761 |
| TCGA-QK-A8Z7-01 | High | 7.718 |
| TCGA-BB-8601-01 | High | 7.097 |
| TCGA-UF-A7JC-01 | High | 7.498 |
| TCGA-F7-A61V-01 | High | 7.403 |
| TCGA-CV-5439-01 | High | 6.781 |
| TCGA-CR-5248-01 | High | 6.404 |
| TCGA-CQ-6223-01 | High | 8.147 |
| TCGA-CR-7367-01 | High | 6.459 |
| TCGA-UF-A71A-01 | High | 7.181 |
| TCGA-CR-7371-01 | High | 7.094 |
| TCGA-CQ-6225-01 | High | 7.879 |
| TCGA-CQ-6229-01 | High | 8.007 |
| TCGA-CR-7385-01 | High | 7.484 |
| TCGA-CV-7446-01 | High | 6.847 |
| TCGA-MT-A51X-01 | High | 7.048 |
| TCGA-CN-4741-01 | High | 6.465 |
| TCGA-CN-5364-01 | High | 6.764 |
| TCGA-CV-7245-01 | High | 7.702 |
| TCGA-CV-7423-01 | High | 6.426 |
| TCGA-BA-6869-01 | High | 7.825 |
| TCGA-CN-4740-01 | High | 7.095 |
| TCGA-P3-A5QF-01 | High | 7.714 |
| TCGA-UF-A71A-06 | High | 7.581 |
