# Supplementary material for: Predictive and mechanistic insights of GLTP on survival in patients with head and neck squamous cell carcinoma
Source: PeerJ. 2026 Aug 5;14:e21611. doi: 10.7717/peerj.21611 (PMC13452426; doi:10.7717/peerj.21611)
Supplement: Supplementary file 2 [file peerj-14-21611-s006.docx]

**Table S2. DEGs between low and high *GLTP* expression groups.**

| **Gene** | **pvalue** | **Log_2_FC** | **FDR** |
| --- | --- | --- | --- |
| A2M-AS1 | <0.001 | 0.394 | 0.001 |
| A2ML1 | <0.001 | -0.677 | <0.001 |
| A2MP1 | 0.002 | 0.500 | 0.009 |
| AADAC | <0.001 | -0.765 | 0.003 |
| AADACL2 | <0.001 | -2.039 | <0.001 |
| AADACP1 | 0.006 | -0.473 | 0.023 |
| AATBC | <0.001 | -0.369 | 0.001 |
| ABAT | 0.015 | -0.282 | 0.048 |
| ABCA12 | <0.001 | -0.703 | <0.001 |
| ABCA13 | 0.001 | -0.426 | 0.007 |
| ABCA3 | 0.003 | 0.458 | 0.013 |
| ABCA6 | <0.001 | 0.440 | 0.005 |
| ABCB4 | <0.001 | 0.556 | <0.001 |
| ABCC9 | <0.001 | 0.497 | <0.001 |
| ABCG4 | <0.001 | -1.566 | <0.001 |
| ABI3 | <0.001 | 0.270 | <0.001 |
| ABI3BP | <0.001 | 0.508 | 0.001 |
| ABLIM1 | <0.001 | -0.321 | <0.001 |
| ABO | 0.005 | -0.344 | 0.021 |
| AC000067.1 | <0.001 | 0.722 | <0.001 |
| AC000403.1 | <0.001 | 0.649 | <0.001 |
| AC002070.1 | <0.001 | 0.468 | <0.001 |
| AC002350.2 | <0.001 | -0.307 | <0.001 |
| AC002401.4 | <0.001 | 0.564 | <0.001 |
| AC002451.1 | <0.001 | 0.823 | 0.004 |
| AC002456.1 | <0.001 | 0.872 | <0.001 |
| AC002480.5 | <0.001 | 0.666 | 0.003 |
| AC002511.1 | <0.001 | -1.056 | <0.001 |
| AC002511.2 | <0.001 | -1.043 | <0.001 |
| AC003070.1 | <0.001 | -0.456 | <0.001 |
| AC003102.1 | <0.001 | -0.356 | <0.001 |
| AC003965.2 | 0.004 | -0.404 | 0.018 |
| AC003986.2 | <0.001 | 0.656 | 0.002 |
| AC004039.1 | <0.001 | 0.503 | <0.001 |
| AC004129.2 | 0.009 | 0.528 | 0.033 |
| AC004156.1 | 0.001 | 0.503 | 0.007 |
| AC004158.1 | <0.001 | -0.580 | <0.001 |
| AC004160.1 | 0.005 | 0.493 | 0.021 |
| AC004221.1 | 0.007 | -0.645 | 0.028 |
| AC004231.1 | 0.006 | -0.517 | 0.025 |
| AC004264.1 | 0.001 | 0.592 | 0.008 |
| AC004386.1 | 0.014 | 0.527 | 0.046 |
| AC004461.2 | 0.014 | -0.418 | 0.046 |
| AC004461.3 | 0.008 | -0.326 | 0.030 |
| AC004466.1 | 0.009 | -0.276 | 0.033 |
| AC004471.1 | <0.001 | -0.375 | 0.001 |
| AC004471.2 | <0.001 | -0.549 | <0.001 |
| AC004490.1 | 0.007 | -0.732 | 0.028 |
| AC004540.1 | <0.001 | 0.733 | <0.001 |
| AC004585.1 | <0.001 | 0.455 | <0.001 |
| AC004692.1 | 0.005 | 0.543 | 0.021 |
| AC004706.1 | <0.001 | -0.476 | <0.001 |
| AC004803.1 | <0.001 | 0.299 | 0.004 |
| AC004808.2 | <0.001 | -1.241 | <0.001 |
| AC004816.1 | <0.001 | -0.622 | <0.001 |
| AC004817.3 | <0.001 | 1.099 | 0.005 |
| AC004884.2 | <0.001 | -0.659 | <0.001 |
| AC004917.1 | 0.012 | 0.419 | 0.040 |
| AC004947.2 | 0.001 | 0.789 | 0.007 |
| AC004951.2 | <0.001 | -0.827 | <0.001 |
| AC004951.3 | <0.001 | -0.668 | <0.001 |
| AC004951.4 | <0.001 | -0.664 | <0.001 |
| AC004975.2 | <0.001 | -0.914 | <0.001 |
| AC004982.2 | 0.003 | -0.295 | 0.013 |
| AC004990.1 | <0.001 | -1.328 | <0.001 |
| AC005034.3 | 0.012 | 0.413 | 0.040 |
| AC005034.5 | 0.003 | 0.588 | 0.014 |
| AC005050.1 | 0.010 | 0.578 | 0.036 |
| AC005052.1 | 0.005 | 0.421 | 0.021 |
| AC005062.1 | <0.001 | -0.704 | 0.002 |
| AC005064.1 | <0.001 | -1.519 | 0.001 |
| AC005077.2 | <0.001 | -0.937 | 0.001 |
| AC005077.4 | <0.001 | -0.985 | <0.001 |
| AC005083.1 | <0.001 | -0.546 | <0.001 |
| AC005162.1 | <0.001 | -1.288 | <0.001 |
| AC005165.1 | 0.006 | 0.798 | 0.023 |
| AC005180.1 | 0.007 | 0.648 | 0.027 |
| AC005180.2 | 0.003 | 0.604 | 0.013 |
| AC005256.1 | <0.001 | -0.600 | 0.003 |
| AC005330.1 | <0.001 | 0.687 | <0.001 |
| AC005336.1 | <0.001 | -0.632 | 0.005 |
| AC005355.1 | 0.006 | 0.543 | 0.024 |
| AC005383.1 | 0.002 | 0.468 | 0.012 |
| AC005392.2 | <0.001 | -1.112 | <0.001 |
| AC005477.1 | 0.014 | -0.605 | 0.047 |
| AC005479.1 | 0.002 | 0.357 | 0.010 |
| AC005586.1 | <0.001 | -0.415 | <0.001 |
| AC005696.4 | 0.007 | -0.518 | 0.026 |
| AC005703.6 | <0.001 | 0.613 | <0.001 |
| AC005722.4 | <0.001 | -1.164 | <0.001 |
| AC005821.1 | <0.001 | 1.019 | <0.001 |
| AC005842.1 | 0.006 | 0.728 | 0.022 |
| AC005845.1 | 0.006 | -1.049 | 0.024 |
| AC005911.1 | 0.009 | -0.273 | 0.031 |
| AC005920.4 | <0.001 | -1.337 | <0.001 |
| AC006026.3 | 0.003 | -0.548 | 0.014 |
| AC006030.1 | 0.009 | 0.333 | 0.034 |
| AC006033.2 | 0.010 | 0.335 | 0.034 |
| AC006057.1 | 0.005 | -0.325 | 0.019 |
| AC006059.1 | <0.001 | 0.514 | <0.001 |
| AC006213.4 | 0.005 | 0.288 | 0.020 |
| AC006237.1 | <0.001 | 1.094 | <0.001 |
| AC006238.1 | 0.004 | -0.429 | 0.016 |
| AC006254.1 | <0.001 | 0.519 | 0.002 |
| AC006272.1 | 0.012 | 0.349 | 0.041 |
| AC006333.1 | <0.001 | -0.308 | <0.001 |
| AC006357.1 | 0.009 | -0.677 | 0.031 |
| AC006372.1 | 0.015 | -0.642 | 0.049 |
| AC006372.3 | <0.001 | -0.922 | 0.004 |
| AC006378.1 | 0.012 | 0.389 | 0.041 |
| AC006538.1 | <0.001 | 0.405 | 0.004 |
| AC006947.1 | 0.005 | -0.591 | 0.021 |
| AC006970.1 | <0.001 | 0.515 | 0.003 |
| AC007014.2 | 0.004 | -0.300 | 0.016 |
| AC007041.1 | <0.001 | -0.624 | <0.001 |
| AC007098.1 | <0.001 | -0.679 | <0.001 |
| AC007182.1 | <0.001 | -1.786 | <0.001 |
| AC007220.1 | 0.003 | 0.587 | 0.015 |
| AC007249.3 | <0.001 | -0.772 | 0.003 |
| AC007292.2 | <0.001 | -0.263 | 0.004 |
| AC007318.3 | 0.008 | 0.357 | 0.029 |
| AC007336.3 | <0.001 | 0.728 | 0.003 |
| AC007342.3 | <0.001 | -0.784 | <0.001 |
| AC007342.4 | <0.001 | -0.519 | <0.001 |
| AC007342.5 | <0.001 | -0.513 | <0.001 |
| AC007342.8 | <0.001 | -0.524 | <0.001 |
| AC007342.9 | <0.001 | -0.506 | <0.001 |
| AC007387.1 | 0.002 | -0.274 | 0.010 |
| AC007389.1 | <0.001 | 0.817 | <0.001 |
| AC007405.2 | <0.001 | 0.770 | 0.001 |
| AC007405.4 | <0.001 | 0.539 | <0.001 |
| AC007406.3 | <0.001 | 0.387 | 0.004 |
| AC007448.4 | <0.001 | 0.474 | 0.005 |
| AC007493.2 | <0.001 | -1.100 | <0.001 |
| AC007541.1 | <0.001 | 0.400 | 0.003 |
| AC007546.1 | 0.006 | -0.314 | 0.023 |
| AC007560.1 | 0.003 | 0.529 | 0.014 |
| AC007570.1 | 0.015 | -0.284 | 0.050 |
| AC007608.3 | <0.001 | -0.795 | <0.001 |
| AC007613.1 | 0.002 | -0.303 | 0.008 |
| AC007681.1 | 0.009 | 0.354 | 0.034 |
| AC007728.2 | 0.002 | 0.333 | 0.008 |
| AC007728.3 | <0.001 | -0.613 | <0.001 |
| AC007750.1 | <0.001 | 0.924 | <0.001 |
| AC007834.1 | <0.001 | -1.583 | <0.001 |
| AC007834.2 | <0.001 | -1.405 | <0.001 |
| AC007848.1 | 0.002 | 0.547 | 0.008 |
| AC007879.2 | <0.001 | 0.691 | <0.001 |
| AC007906.1 | 0.007 | -0.453 | 0.026 |
| AC007906.2 | <0.001 | -0.512 | <0.001 |
| AC007919.1 | 0.003 | 0.583 | 0.012 |
| AC007998.3 | 0.006 | 0.284 | 0.024 |
| AC008013.2 | <0.001 | 0.748 | 0.002 |
| AC008063.1 | 0.007 | -0.710 | 0.027 |
| AC008074.1 | 0.007 | -0.345 | 0.027 |
| AC008074.2 | <0.001 | -0.452 | <0.001 |
| AC008079.1 | 0.005 | 0.373 | 0.021 |
| AC008083.3 | <0.001 | 0.623 | <0.001 |
| AC008105.3 | 0.004 | 0.351 | 0.015 |
| AC008114.1 | 0.006 | -0.437 | 0.024 |
| AC008115.4 | <0.001 | 0.551 | <0.001 |
| AC008147.2 | 0.005 | 0.358 | 0.019 |
| AC008391.1 | 0.005 | -0.418 | 0.022 |
| AC008406.3 | <0.001 | -0.860 | <0.001 |
| AC008443.4 | <0.001 | -0.652 | <0.001 |
| AC008443.5 | 0.014 | 0.275 | 0.045 |
| AC008494.2 | 0.009 | 0.446 | 0.032 |
| AC008555.1 | 0.004 | 0.446 | 0.018 |
| AC008556.1 | <0.001 | -0.550 | <0.001 |
| AC008569.2 | 0.010 | 0.427 | 0.036 |
| AC008592.1 | 0.002 | -0.637 | 0.009 |
| AC008649.1 | <0.001 | -0.896 | <0.001 |
| AC008663.1 | <0.001 | -1.024 | <0.001 |
| AC008663.2 | 0.004 | 0.362 | 0.015 |
| AC008676.1 | 0.009 | 0.381 | 0.032 |
| AC008687.2 | <0.001 | 0.511 | 0.005 |
| AC008687.4 | 0.001 | 0.421 | 0.007 |
| AC008742.1 | <0.001 | 0.761 | <0.001 |
| AC008750.8 | 0.004 | 0.327 | 0.016 |
| AC008759.1 | <0.001 | -1.016 | <0.001 |
| AC008840.1 | <0.001 | 0.466 | 0.004 |
| AC008870.1 | 0.014 | -0.627 | 0.047 |
| AC008897.2 | 0.001 | -0.678 | 0.006 |
| AC008957.1 | 0.005 | 0.404 | 0.019 |
| AC008957.3 | 0.009 | 0.473 | 0.034 |
| AC009078.3 | 0.002 | 0.363 | 0.008 |
| AC009093.1 | <0.001 | 0.728 | <0.001 |
| AC009093.2 | <0.001 | 0.647 | <0.001 |
| AC009118.2 | 0.002 | -0.397 | 0.011 |
| AC009119.1 | <0.001 | -0.631 | <0.001 |
| AC009123.1 | <0.001 | -0.465 | 0.002 |
| AC009159.1 | 0.011 | 0.723 | 0.038 |
| AC009159.4 | <0.001 | 0.717 | 0.003 |
| AC009171.2 | 0.001 | -0.288 | 0.006 |
| AC009268.2 | 0.013 | -0.315 | 0.044 |
| AC009413.2 | 0.003 | 0.573 | 0.013 |
| AC009509.5 | <0.001 | 0.354 | <0.001 |
| AC009549.1 | <0.001 | 0.716 | 0.004 |
| AC009570.1 | <0.001 | -0.577 | <0.001 |
| AC009779.4 | <0.001 | 0.384 | <0.001 |
| AC009806.1 | 0.014 | 0.354 | 0.045 |
| AC009812.1 | <0.001 | 0.464 | 0.002 |
| AC009812.3 | <0.001 | 0.756 | <0.001 |
| AC009812.4 | <0.001 | 0.704 | <0.001 |
| AC009951.4 | <0.001 | 0.679 | <0.001 |
| AC009955.2 | 0.001 | 0.671 | 0.006 |
| AC009955.4 | <0.001 | 0.840 | 0.003 |
| AC009974.1 | <0.001 | 0.849 | 0.006 |
| AC009977.1 | 0.014 | 0.649 | 0.045 |
| AC010149.1 | 0.013 | 0.331 | 0.042 |
| AC010168.1 | 0.005 | -0.322 | 0.019 |
| AC010168.2 | 0.002 | -0.335 | 0.010 |
| AC010173.1 | <0.001 | 0.440 | <0.001 |
| AC010175.1 | <0.001 | 0.729 | <0.001 |
| AC010200.1 | 0.014 | 0.542 | 0.047 |
| AC010201.1 | 0.005 | 0.523 | 0.021 |
| AC010245.2 | <0.001 | 0.331 | <0.001 |
| AC010247.1 | <0.001 | 0.768 | <0.001 |
| AC010247.2 | <0.001 | 0.601 | <0.001 |
| AC010260.1 | <0.001 | -0.442 | <0.001 |
| AC010266.2 | 0.003 | -0.920 | 0.015 |
| AC010280.1 | 0.002 | -0.714 | 0.010 |
| AC010307.2 | 0.004 | -0.652 | 0.016 |
| AC010336.1 | <0.001 | -0.434 | 0.004 |
| AC010336.2 | <0.001 | -0.531 | 0.005 |
| AC010424.1 | 0.012 | 0.511 | 0.042 |
| AC010503.5 | <0.001 | -0.275 | <0.001 |
| AC010531.1 | 0.013 | 0.317 | 0.045 |
| AC010533.1 | <0.001 | 0.639 | 0.003 |
| AC010618.3 | <0.001 | 0.281 | 0.005 |
| AC010654.1 | <0.001 | -0.320 | 0.003 |
| AC010754.1 | <0.001 | 0.749 | <0.001 |
| AC010789.1 | <0.001 | -0.626 | 0.005 |
| AC010864.1 | 0.001 | 0.342 | 0.007 |
| AC010878.1 | 0.002 | 0.542 | 0.009 |
| AC010904.2 | 0.007 | 0.429 | 0.025 |
| AC010969.1 | <0.001 | -2.168 | 0.004 |
| AC010980.2 | 0.010 | -0.393 | 0.035 |
| AC010999.1 | 0.007 | 0.491 | 0.028 |
| AC011008.2 | <0.001 | 0.589 | 0.004 |
| AC011363.1 | <0.001 | -1.125 | <0.001 |
| AC011374.1 | <0.001 | -0.751 | <0.001 |
| AC011379.2 | 0.002 | 0.515 | 0.012 |
| AC011383.1 | 0.014 | 0.487 | 0.046 |
| AC011416.2 | 0.003 | 0.490 | 0.013 |
| AC011450.1 | 0.003 | 0.379 | 0.014 |
| AC011451.2 | 0.009 | 0.374 | 0.034 |
| AC011462.4 | 0.004 | 0.383 | 0.016 |
| AC011466.1 | 0.014 | 0.292 | 0.046 |
| AC011466.4 | 0.009 | 0.394 | 0.032 |
| AC011472.2 | <0.001 | -0.399 | <0.001 |
| AC011473.3 | <0.001 | -2.008 | <0.001 |
| AC011473.4 | <0.001 | -0.846 | <0.001 |
| AC011476.3 | <0.001 | -0.338 | <0.001 |
| AC011477.3 | 0.005 | 0.512 | 0.019 |
| AC011479.2 | 0.010 | 0.329 | 0.036 |
| AC011481.2 | 0.014 | 0.304 | 0.046 |
| AC011483.1 | <0.001 | -1.334 | <0.001 |
| AC011483.2 | <0.001 | -1.679 | <0.001 |
| AC011499.1 | 0.013 | -0.575 | 0.045 |
| AC011503.1 | <0.001 | -1.321 | <0.001 |
| AC011510.1 | <0.001 | 0.635 | 0.002 |
| AC011511.2 | <0.001 | 1.373 | 0.002 |
| AC011511.5 | <0.001 | 0.446 | 0.004 |
| AC011530.1 | 0.003 | 0.640 | 0.013 |
| AC011595.2 | <0.001 | 1.065 | <0.001 |
| AC011601.1 | 0.003 | -0.490 | 0.014 |
| AC011742.3 | 0.005 | -0.558 | 0.019 |
| AC011815.3 | 0.011 | 0.312 | 0.037 |
| AC011899.2 | 0.008 | 0.378 | 0.029 |
| AC011933.1 | 0.007 | 0.354 | 0.027 |
| AC011944.1 | <0.001 | 0.663 | <0.001 |
| AC011979.1 | 0.002 | 0.304 | 0.010 |
| AC012065.2 | 0.008 | 0.391 | 0.030 |
| AC012065.3 | 0.013 | 0.354 | 0.044 |
| AC012085.2 | 0.003 | 0.694 | 0.012 |
| AC012146.1 | 0.007 | 0.289 | 0.026 |
| AC012158.1 | <0.001 | -0.497 | 0.005 |
| AC012173.1 | 0.001 | -0.383 | 0.007 |
| AC012213.1 | 0.007 | 0.441 | 0.026 |
| AC012213.3 | 0.003 | 0.348 | 0.014 |
| AC012358.4 | <0.001 | -0.805 | <0.001 |
| AC012360.2 | <0.001 | 0.410 | 0.001 |
| AC012363.2 | 0.013 | -0.389 | 0.043 |
| AC012409.3 | 0.002 | 0.711 | 0.009 |
| AC012417.1 | 0.001 | -1.489 | 0.006 |
| AC012442.3 | <0.001 | 0.343 | 0.003 |
| AC012501.2 | 0.005 | -0.974 | 0.020 |
| AC012501.3 | 0.003 | -1.004 | 0.015 |
| AC012511.1 | <0.001 | 0.598 | <0.001 |
| AC012513.3 | 0.002 | 0.274 | 0.011 |
| AC012531.1 | 0.003 | 0.636 | 0.013 |
| AC012636.1 | 0.006 | 0.551 | 0.024 |
| AC012640.5 | 0.003 | 0.636 | 0.012 |
| AC012640.7 | <0.001 | 0.561 | 0.005 |
| AC012676.3 | <0.001 | -0.427 | 0.005 |
| AC013451.1 | <0.001 | 0.843 | 0.001 |
| AC013489.1 | 0.002 | 0.440 | 0.010 |
| AC013553.3 | 0.002 | 0.476 | 0.008 |
| AC015660.1 | <0.001 | -0.709 | <0.001 |
| AC015660.2 | <0.001 | -0.970 | <0.001 |
| AC015712.1 | 0.010 | 0.345 | 0.036 |
| AC015818.3 | <0.001 | -1.383 | <0.001 |
| AC015819.2 | <0.001 | -0.594 | <0.001 |
| AC015849.1 | 0.008 | 0.534 | 0.031 |
| AC015853.2 | 0.015 | 0.895 | 0.048 |
| AC015909.1 | 0.003 | 0.813 | 0.012 |
| AC015909.2 | 0.005 | 0.657 | 0.021 |
| AC015911.3 | 0.001 | 0.455 | 0.008 |
| AC015911.4 | 0.005 | 0.411 | 0.022 |
| AC015911.7 | 0.005 | 0.335 | 0.022 |
| AC015911.8 | 0.003 | 0.303 | 0.014 |
| AC015922.2 | <0.001 | 0.325 | 0.002 |
| AC015922.3 | <0.001 | 0.377 | <0.001 |
| AC015961.2 | <0.001 | 0.751 | 0.002 |
| AC016355.1 | 0.006 | -0.273 | 0.024 |
| AC016396.2 | <0.001 | 1.120 | 0.001 |
| AC016397.2 | <0.001 | 0.940 | <0.001 |
| AC016571.1 | <0.001 | 0.788 | <0.001 |
| AC016575.1 | 0.003 | 0.707 | 0.012 |
| AC016582.1 | <0.001 | -0.911 | 0.002 |
| AC016705.2 | 0.002 | 1.098 | 0.010 |
| AC016747.1 | <0.001 | 0.432 | <0.001 |
| AC016888.1 | <0.001 | -0.374 | <0.001 |
| AC016924.1 | <0.001 | 0.664 | 0.001 |
| AC016999.1 | 0.010 | 0.371 | 0.036 |
| AC017002.2 | <0.001 | 0.567 | <0.001 |
| AC017048.3 | <0.001 | 0.894 | <0.001 |
| AC017074.1 | <0.001 | -0.310 | 0.002 |
| AC017100.1 | <0.001 | 0.437 | 0.003 |
| AC017101.1 | 0.004 | 0.556 | 0.017 |
| AC017104.1 | <0.001 | 0.697 | <0.001 |
| AC018410.1 | 0.003 | 0.381 | 0.012 |
| AC018529.2 | 0.015 | 0.286 | 0.047 |
| AC018552.3 | 0.012 | -0.500 | 0.040 |
| AC018628.2 | <0.001 | -0.292 | <0.001 |
| AC018630.2 | <0.001 | 0.582 | <0.001 |
| AC018648.1 | 0.006 | 0.459 | 0.024 |
| AC018755.4 | 0.009 | 0.377 | 0.033 |
| AC018781.1 | <0.001 | -0.904 | <0.001 |
| AC019080.1 | <0.001 | -0.857 | <0.001 |
| AC019080.3 | 0.011 | -0.471 | 0.037 |
| AC019080.5 | <0.001 | 0.318 | 0.005 |
| AC019118.1 | <0.001 | -0.811 | <0.001 |
| AC019131.1 | <0.001 | -1.836 | <0.001 |
| AC019181.1 | 0.002 | -0.361 | 0.009 |
| AC019181.2 | 0.004 | -0.640 | 0.016 |
| AC019197.1 | <0.001 | -0.913 | 0.003 |
| AC019206.1 | 0.012 | 0.313 | 0.040 |
| AC019211.1 | <0.001 | -0.614 | 0.004 |
| AC019294.2 | <0.001 | 0.458 | 0.004 |
| AC020594.1 | <0.001 | 0.520 | <0.001 |
| AC020633.1 | 0.006 | -0.625 | 0.023 |
| AC020634.2 | 0.010 | 0.414 | 0.036 |
| AC020658.7 | 0.009 | -0.569 | 0.034 |
| AC020661.1 | <0.001 | -0.315 | 0.002 |
| AC020907.4 | 0.004 | 0.359 | 0.017 |
| AC020978.4 | <0.001 | -0.611 | <0.001 |
| AC020978.9 | <0.001 | 0.330 | 0.005 |
| AC021028.1 | <0.001 | 0.338 | 0.004 |
| AC021074.1 | 0.007 | 0.289 | 0.025 |
| AC021087.3 | 0.004 | 0.292 | 0.016 |
| AC021087.4 | <0.001 | 0.795 | 0.002 |
| AC021106.2 | 0.003 | 0.697 | 0.013 |
| AC021188.1 | 0.011 | 0.316 | 0.038 |
| AC021205.3 | 0.001 | 0.543 | 0.006 |
| AC021491.4 | 0.002 | 0.494 | 0.010 |
| AC021683.3 | 0.005 | -1.210 | 0.019 |
| AC022001.3 | 0.011 | 1.168 | 0.039 |
| AC022018.1 | 0.007 | 0.315 | 0.026 |
| AC022034.1 | <0.001 | 0.593 | <0.001 |
| AC022034.4 | <0.001 | 0.721 | <0.001 |
| AC022092.1 | 0.002 | 0.739 | 0.009 |
| AC022098.4 | 0.005 | -0.460 | 0.020 |
| AC022126.1 | <0.001 | 0.814 | 0.001 |
| AC022149.1 | <0.001 | 0.473 | <0.001 |
| AC022167.4 | 0.004 | -0.520 | 0.016 |
| AC022182.2 | <0.001 | 0.684 | 0.005 |
| AC022417.1 | <0.001 | 0.283 | 0.003 |
| AC022467.1 | <0.001 | 0.967 | <0.001 |
| AC022509.1 | 0.001 | 1.173 | 0.007 |
| AC022509.2 | 0.001 | 0.507 | 0.007 |
| AC022509.3 | <0.001 | 0.531 | <0.001 |
| AC022613.1 | <0.001 | 1.095 | <0.001 |
| AC022613.2 | <0.001 | -0.332 | <0.001 |
| AC022893.2 | <0.001 | 1.972 | <0.001 |
| AC022905.1 | 0.005 | 0.579 | 0.020 |
| AC022915.3 | <0.001 | 1.133 | 0.004 |
| AC023024.1 | 0.003 | -0.305 | 0.012 |
| AC023024.2 | 0.009 | -0.297 | 0.032 |
| AC023043.1 | <0.001 | 0.332 | 0.001 |
| AC023157.2 | <0.001 | -1.172 | <0.001 |
| AC023157.3 | <0.001 | -0.620 | <0.001 |
| AC023282.1 | 0.007 | 0.632 | 0.027 |
| AC023302.1 | 0.005 | -0.395 | 0.022 |
| AC023421.1 | 0.003 | 0.688 | 0.014 |
| AC023632.1 | 0.002 | 0.628 | 0.009 |
| AC023632.2 | <0.001 | 0.453 | <0.001 |
| AC023794.2 | <0.001 | 0.693 | 0.001 |
| AC023794.3 | <0.001 | 0.556 | 0.005 |
| AC023794.4 | 0.002 | 0.723 | 0.009 |
| AC023794.5 | 0.009 | 0.498 | 0.032 |
| AC023794.7 | <0.001 | 0.753 | <0.001 |
| AC023906.1 | 0.002 | 0.573 | 0.011 |
| AC023906.2 | <0.001 | -0.584 | <0.001 |
| AC023906.4 | <0.001 | -0.626 | 0.005 |
| AC023906.5 | <0.001 | -0.463 | 0.001 |
| AC024337.2 | 0.001 | 0.533 | 0.007 |
| AC024560.1 | <0.001 | -0.976 | 0.002 |
| AC024592.2 | <0.001 | -1.122 | <0.001 |
| AC024909.1 | 0.006 | 0.365 | 0.023 |
| AC024940.2 | <0.001 | 0.834 | 0.002 |
| AC025030.1 | 0.003 | -0.491 | 0.013 |
| AC025048.2 | 0.010 | 0.279 | 0.036 |
| AC025048.4 | <0.001 | -0.491 | <0.001 |
| AC025165.3 | <0.001 | 0.410 | <0.001 |
| AC025166.1 | 0.008 | -0.352 | 0.031 |
| AC025171.4 | 0.012 | 0.284 | 0.041 |
| AC025175.1 | 0.010 | 0.290 | 0.036 |
| AC025181.1 | 0.002 | -1.304 | 0.009 |
| AC025188.1 | <0.001 | 0.703 | 0.005 |
| AC025244.1 | 0.010 | -0.532 | 0.035 |
| AC025263.1 | 0.004 | -0.543 | 0.016 |
| AC025419.1 | <0.001 | 0.659 | 0.003 |
| AC025754.2 | <0.001 | 0.702 | <0.001 |
| AC025809.1 | 0.001 | 0.572 | 0.007 |
| AC025809.2 | 0.002 | 0.567 | 0.010 |
| AC025884.1 | <0.001 | -0.583 | <0.001 |
| AC026355.2 | <0.001 | -0.853 | <0.001 |
| AC026355.4 | <0.001 | -0.950 | 0.005 |
| AC026356.2 | <0.001 | 0.324 | 0.003 |
| AC026367.1 | 0.002 | -0.429 | 0.009 |
| AC026369.2 | <0.001 | 0.395 | <0.001 |
| AC026436.1 | <0.001 | 0.324 | 0.004 |
| AC026470.2 | <0.001 | -0.355 | 0.003 |
| AC026471.3 | 0.002 | -0.861 | 0.009 |
| AC026471.6 | 0.011 | 0.313 | 0.037 |
| AC026785.3 | 0.009 | 0.817 | 0.033 |
| AC027020.2 | 0.006 | -0.271 | 0.022 |
| AC027097.1 | <0.001 | 0.368 | <0.001 |
| AC027097.2 | <0.001 | 0.587 | <0.001 |
| AC027228.3 | 0.011 | 0.422 | 0.037 |
| AC027237.5 | <0.001 | 0.530 | <0.001 |
| AC027237.6 | 0.004 | 0.527 | 0.017 |
| AC027288.3 | 0.001 | 0.575 | 0.007 |
| AC027290.3 | 0.001 | -0.305 | 0.007 |
| AC027307.1 | 0.009 | -0.354 | 0.033 |
| AC027338.1 | 0.004 | 0.609 | 0.018 |
| AC027514.1 | <0.001 | -0.705 | <0.001 |
| AC027559.1 | <0.001 | 0.485 | 0.004 |
| AC027575.3 | <0.001 | 0.390 | <0.001 |
| AC027612.1 | <0.001 | -1.208 | <0.001 |
| AC027612.3 | 0.006 | -0.353 | 0.023 |
| AC027682.6 | <0.001 | 0.584 | 0.003 |
| AC027702.2 | <0.001 | 0.393 | 0.002 |
| AC027801.2 | <0.001 | -1.138 | <0.001 |
| AC032011.1 | 0.003 | 0.535 | 0.014 |
| AC034102.6 | <0.001 | 0.363 | 0.004 |
| AC034199.1 | <0.001 | 0.521 | 0.002 |
| AC034206.1 | <0.001 | -0.563 | <0.001 |
| AC034213.1 | 0.007 | 0.600 | 0.027 |
| AC034238.2 | 0.002 | 0.581 | 0.011 |
| AC035140.1 | 0.009 | 0.540 | 0.033 |
| AC036176.1 | 0.002 | 0.266 | 0.010 |
| AC036176.2 | <0.001 | -0.705 | 0.002 |
| AC037198.1 | <0.001 | 0.735 | <0.001 |
| AC037198.2 | <0.001 | 0.617 | <0.001 |
| AC039056.1 | <0.001 | -1.817 | <0.001 |
| AC040168.1 | 0.006 | 0.679 | 0.023 |
| AC040169.3 | <0.001 | -0.379 | <0.001 |
| AC040174.1 | 0.008 | -0.752 | 0.029 |
| AC040174.2 | 0.011 | -0.740 | 0.037 |
| AC040970.1 | <0.001 | 0.427 | <0.001 |
| AC041040.1 | <0.001 | 0.869 | 0.005 |
| AC046130.3 | 0.001 | 0.452 | 0.007 |
| AC046185.3 | 0.006 | 0.282 | 0.023 |
| AC048351.2 | 0.011 | 0.418 | 0.037 |
| AC048380.2 | 0.012 | 0.361 | 0.040 |
| AC048382.5 | <0.001 | 0.390 | 0.003 |
| AC051619.4 | 0.002 | 0.570 | 0.009 |
| AC053503.1 | <0.001 | 0.747 | <0.001 |
| AC053503.5 | 0.005 | 0.851 | 0.019 |
| AC055822.1 | <0.001 | 0.464 | <0.001 |
| AC060234.2 | <0.001 | 0.657 | 0.005 |
| AC060766.4 | <0.001 | 0.295 | 0.001 |
| AC060766.5 | 0.001 | 0.289 | 0.007 |
| AC060766.6 | 0.009 | 0.380 | 0.032 |
| AC062028.1 | 0.015 | -0.773 | 0.047 |
| AC063944.1 | 0.004 | 0.593 | 0.017 |
| AC063944.2 | <0.001 | 1.024 | <0.001 |
| AC063965.2 | 0.012 | 0.284 | 0.041 |
| AC063976.2 | <0.001 | 0.894 | <0.001 |
| AC064807.2 | <0.001 | 0.586 | 0.005 |
| AC064807.4 | <0.001 | 0.443 | <0.001 |
| AC064875.1 | 0.009 | 0.607 | 0.033 |
| AC067930.2 | 0.004 | 0.437 | 0.016 |
| AC067945.3 | 0.002 | 0.400 | 0.009 |
| AC068189.1 | <0.001 | -0.623 | 0.003 |
| AC068228.2 | 0.009 | -0.341 | 0.033 |
| AC068234.2 | <0.001 | 0.883 | <0.001 |
| AC068338.2 | <0.001 | -0.267 | <0.001 |
| AC068491.3 | <0.001 | 0.745 | 0.001 |
| AC068594.1 | <0.001 | -1.135 | <0.001 |
| AC068633.1 | <0.001 | -1.279 | 0.001 |
| AC068647.1 | <0.001 | -1.656 | 0.001 |
| AC068700.1 | <0.001 | 0.841 | 0.005 |
| AC068831.7 | <0.001 | -0.345 | <0.001 |
| AC068860.1 | <0.001 | 0.991 | <0.001 |
| AC069218.1 | 0.014 | 0.286 | 0.046 |
| AC069236.1 | 0.008 | -0.501 | 0.029 |
| AC069360.1 | <0.001 | 0.684 | <0.001 |
| AC069431.1 | <0.001 | -1.188 | 0.001 |
| AC072062.1 | <0.001 | -0.932 | <0.001 |
| AC073052.1 | 0.003 | -0.403 | 0.012 |
| AC073115.1 | 0.003 | 1.043 | 0.012 |
| AC073349.1 | 0.003 | -0.646 | 0.013 |
| AC073365.1 | <0.001 | -0.866 | 0.001 |
| AC073370.1 | 0.005 | -0.695 | 0.021 |
| AC073475.1 | 0.004 | 0.361 | 0.019 |
| AC073517.1 | 0.008 | 0.361 | 0.029 |
| AC073592.2 | 0.012 | -0.698 | 0.041 |
| AC073610.1 | <0.001 | 0.515 | <0.001 |
| AC073610.2 | 0.003 | 0.544 | 0.013 |
| AC073648.3 | <0.001 | -1.352 | <0.001 |
| AC073655.2 | 0.007 | 0.311 | 0.026 |
| AC073842.1 | <0.001 | -0.790 | 0.001 |
| AC073909.1 | <0.001 | 0.639 | 0.005 |
| AC073912.2 | 0.015 | 0.446 | 0.048 |
| AC073957.1 | <0.001 | -0.710 | <0.001 |
| AC074011.1 | <0.001 | -0.684 | <0.001 |
| AC078785.1 | <0.001 | 0.513 | 0.005 |
| AC078820.1 | 0.013 | -0.378 | 0.043 |
| AC078845.1 | 0.012 | -0.562 | 0.040 |
| AC078860.2 | 0.002 | 0.839 | 0.008 |
| AC078880.3 | 0.005 | 0.614 | 0.021 |
| AC078881.1 | <0.001 | -0.957 | 0.002 |
| AC078906.1 | <0.001 | 0.654 | 0.005 |
| AC078923.1 | <0.001 | -0.506 | 0.005 |
| AC079015.1 | 0.002 | 0.699 | 0.008 |
| AC079145.1 | <0.001 | 0.661 | 0.002 |
| AC079174.1 | <0.001 | -0.432 | 0.003 |
| AC079209.1 | 0.003 | 0.379 | 0.014 |
| AC079228.1 | 0.006 | 0.404 | 0.024 |
| AC079298.3 | <0.001 | 0.784 | <0.001 |
| AC079305.2 | <0.001 | -0.808 | <0.001 |
| AC079305.4 | 0.009 | -0.635 | 0.034 |
| AC079313.1 | <0.001 | 0.785 | <0.001 |
| AC079313.2 | <0.001 | 0.684 | 0.004 |
| AC079325.6 | <0.001 | 0.418 | 0.005 |
| AC079336.1 | <0.001 | -0.691 | <0.001 |
| AC079336.2 | <0.001 | -0.536 | <0.001 |
| AC079336.5 | <0.001 | -1.192 | <0.001 |
| AC079336.6 | <0.001 | -0.624 | <0.001 |
| AC079385.3 | 0.002 | 0.505 | 0.010 |
| AC079610.1 | 0.008 | -0.330 | 0.031 |
| AC079753.1 | 0.013 | -1.373 | 0.042 |
| AC079921.1 | <0.001 | 0.393 | 0.003 |
| AC079921.2 | <0.001 | 0.416 | <0.001 |
| AC079922.2 | <0.001 | 0.281 | 0.003 |
| AC080023.1 | <0.001 | -0.349 | 0.002 |
| AC080038.2 | <0.001 | 0.586 | <0.001 |
| AC080038.4 | 0.012 | 0.307 | 0.040 |
| AC080100.1 | 0.013 | 0.696 | 0.044 |
| AC080188.2 | 0.012 | -0.269 | 0.041 |
| AC082651.3 | <0.001 | -0.536 | <0.001 |
| AC083801.2 | <0.001 | -1.838 | <0.001 |
| AC083841.1 | <0.001 | -1.737 | <0.001 |
| AC083862.2 | <0.001 | 0.438 | <0.001 |
| AC083880.1 | 0.002 | -0.301 | 0.009 |
| AC083967.1 | <0.001 | 2.233 | <0.001 |
| AC083973.1 | <0.001 | -0.430 | <0.001 |
| AC084026.1 | <0.001 | 0.975 | <0.001 |
| AC084026.2 | 0.007 | 0.650 | 0.026 |
| AC084030.1 | 0.001 | -0.689 | 0.006 |
| AC084032.1 | 0.004 | 0.520 | 0.017 |
| AC084357.3 | <0.001 | -1.832 | <0.001 |
| AC084757.3 | <0.001 | 0.796 | <0.001 |
| AC084782.3 | 0.002 | 0.549 | 0.009 |
| AC084809.1 | <0.001 | -0.393 | <0.001 |
| AC084809.2 | 0.014 | -0.312 | 0.047 |
| AC084816.1 | 0.009 | -1.028 | 0.031 |
| AC084819.1 | <0.001 | -1.085 | 0.001 |
| AC084871.1 | <0.001 | -0.895 | <0.001 |
| AC084871.2 | <0.001 | -0.616 | <0.001 |
| AC084876.2 | <0.001 | -0.462 | <0.001 |
| AC084880.3 | 0.004 | -0.608 | 0.017 |
| AC087239.1 | 0.005 | 0.399 | 0.021 |
| AC087477.2 | <0.001 | 0.703 | <0.001 |
| AC087491.1 | 0.008 | -0.702 | 0.029 |
| AC087500.1 | <0.001 | 0.368 | <0.001 |
| AC087521.1 | <0.001 | 0.597 | 0.002 |
| AC087623.2 | <0.001 | -0.392 | 0.003 |
| AC087783.1 | <0.001 | -0.894 | 0.004 |
| AC087793.1 | 0.012 | 0.271 | 0.041 |
| AC087878.1 | 0.006 | 0.570 | 0.023 |
| AC089987.2 | <0.001 | -0.802 | 0.002 |
| AC090115.1 | 0.002 | 0.545 | 0.009 |
| AC090125.1 | <0.001 | 0.860 | 0.005 |
| AC090152.1 | <0.001 | 0.762 | <0.001 |
| AC090164.2 | <0.001 | -1.726 | <0.001 |
| AC090181.2 | <0.001 | -0.382 | 0.004 |
| AC090197.1 | <0.001 | 0.969 | <0.001 |
| AC090204.1 | <0.001 | -0.476 | 0.003 |
| AC090246.1 | 0.015 | -0.320 | 0.048 |
| AC090337.1 | 0.006 | 0.624 | 0.023 |
| AC090360.1 | <0.001 | 0.678 | <0.001 |
| AC090409.2 | 0.006 | 0.544 | 0.023 |
| AC090515.5 | 0.001 | -0.919 | 0.006 |
| AC090559.1 | <0.001 | 0.439 | <0.001 |
| AC090587.2 | <0.001 | -0.340 | <0.001 |
| AC090617.4 | 0.008 | 0.473 | 0.030 |
| AC090772.1 | 0.012 | 0.385 | 0.040 |
| AC090825.1 | <0.001 | 0.827 | <0.001 |
| AC090877.2 | 0.009 | 0.524 | 0.033 |
| AC090907.1 | 0.003 | 0.461 | 0.013 |
| AC090948.2 | <0.001 | 0.388 | 0.002 |
| AC090970.2 | <0.001 | -0.509 | <0.001 |
| AC091057.3 | 0.001 | 0.383 | 0.007 |
| AC091132.2 | <0.001 | -0.592 | <0.001 |
| AC091212.1 | <0.001 | 0.780 | 0.003 |
| AC091488.1 | 0.007 | 0.462 | 0.026 |
| AC091551.1 | <0.001 | 0.742 | <0.001 |
| AC091564.6 | 0.002 | 0.706 | 0.010 |
| AC091588.3 | <0.001 | 0.807 | <0.001 |
| AC091729.1 | <0.001 | -0.499 | 0.004 |
| AC091825.3 | <0.001 | 0.330 | <0.001 |
| AC091868.2 | 0.003 | -0.439 | 0.015 |
| AC091965.1 | <0.001 | 0.789 | <0.001 |
| AC091965.4 | <0.001 | 0.700 | <0.001 |
| AC091979.1 | <0.001 | 1.139 | <0.001 |
| AC092032.1 | 0.010 | 0.294 | 0.036 |
| AC092115.4 | 0.004 | -0.362 | 0.018 |
| AC092117.1 | <0.001 | -0.752 | <0.001 |
| AC092118.1 | <0.001 | -0.500 | 0.002 |
| AC092135.1 | <0.001 | -0.857 | <0.001 |
| AC092135.2 | 0.004 | -0.791 | 0.016 |
| AC092140.2 | 0.014 | 0.343 | 0.047 |
| AC092162.2 | 0.015 | 0.451 | 0.049 |
| AC092162.3 | <0.001 | 0.648 | 0.002 |
| AC092171.3 | <0.001 | -0.358 | <0.001 |
| AC092171.5 | <0.001 | -0.353 | <0.001 |
| AC092338.1 | 0.009 | 0.389 | 0.032 |
| AC092354.2 | <0.001 | 0.389 | <0.001 |
| AC092376.2 | <0.001 | 0.716 | <0.001 |
| AC092384.2 | <0.001 | -0.641 | <0.001 |
| AC092437.1 | 0.005 | 0.440 | 0.021 |
| AC092490.1 | <0.001 | -1.565 | <0.001 |
| AC092597.1 | 0.003 | 0.276 | 0.015 |
| AC092645.1 | <0.001 | 0.751 | <0.001 |
| AC092647.5 | 0.003 | 0.767 | 0.014 |
| AC092687.3 | <0.001 | 0.290 | 0.002 |
| AC092798.1 | 0.003 | 0.438 | 0.013 |
| AC092802.3 | 0.011 | 0.324 | 0.039 |
| AC092807.3 | 0.006 | 0.528 | 0.024 |
| AC092809.4 | 0.006 | -0.267 | 0.023 |
| AC092851.1 | 0.008 | -0.645 | 0.031 |
| AC092919.3 | 0.015 | 0.382 | 0.048 |
| AC093083.1 | <0.001 | -1.024 | <0.001 |
| AC093157.1 | <0.001 | 0.335 | <0.001 |
| AC093159.1 | <0.001 | -0.807 | 0.001 |
| AC093367.1 | 0.002 | -0.533 | 0.011 |
| AC093382.1 | <0.001 | -0.423 | 0.004 |
| AC093423.2 | 0.009 | 0.327 | 0.033 |
| AC093424.1 | <0.001 | 0.685 | 0.003 |
| AC093425.1 | <0.001 | 1.161 | <0.001 |
| AC093458.2 | 0.014 | 0.602 | 0.046 |
| AC093525.2 | <0.001 | 0.510 | 0.005 |
| AC093535.2 | <0.001 | 0.498 | <0.001 |
| AC093536.1 | <0.001 | -0.279 | 0.002 |
| AC093567.1 | 0.005 | 0.417 | 0.019 |
| AC093583.1 | 0.014 | 0.487 | 0.046 |
| AC093616.1 | <0.001 | 0.743 | <0.001 |
| AC093627.1 | 0.008 | 0.927 | 0.030 |
| AC093627.7 | 0.004 | 0.627 | 0.019 |
| AC093702.1 | 0.005 | 0.749 | 0.019 |
| AC093726.2 | 0.013 | 0.267 | 0.043 |
| AC093772.1 | 0.004 | -0.680 | 0.017 |
| AC093865.1 | 0.006 | -0.672 | 0.024 |
| AC093866.1 | 0.008 | 0.602 | 0.029 |
| AC093890.1 | 0.001 | 0.532 | 0.007 |
| AC093904.2 | <0.001 | -1.183 | <0.001 |
| AC093904.3 | <0.001 | -2.058 | <0.001 |
| AC093904.4 | <0.001 | -1.376 | <0.001 |
| AC093908.1 | <0.001 | 0.517 | <0.001 |
| AC095031.1 | 0.011 | 0.428 | 0.038 |
| AC096533.1 | 0.007 | 0.266 | 0.026 |
| AC096536.1 | <0.001 | -0.703 | <0.001 |
| AC096564.2 | 0.014 | -0.564 | 0.046 |
| AC096582.7 | 0.013 | 0.304 | 0.045 |
| AC096921.2 | 0.002 | 0.304 | 0.011 |
| AC097634.1 | <0.001 | 0.825 | <0.001 |
| AC097634.3 | <0.001 | 0.970 | 0.001 |
| AC097639.1 | <0.001 | -0.682 | <0.001 |
| AC098487.1 | <0.001 | 0.435 | <0.001 |
| AC098820.2 | <0.001 | 0.505 | <0.001 |
| AC098820.4 | 0.002 | 0.582 | 0.010 |
| AC098824.1 | 0.005 | -0.490 | 0.020 |
| AC098828.2 | <0.001 | -0.790 | <0.001 |
| AC098934.1 | <0.001 | -0.695 | <0.001 |
| AC098934.2 | <0.001 | -0.674 | 0.002 |
| AC099066.2 | 0.009 | 0.314 | 0.031 |
| AC099329.2 | 0.001 | -0.626 | 0.007 |
| AC099336.1 | 0.007 | 0.473 | 0.027 |
| AC099518.1 | 0.002 | 0.450 | 0.010 |
| AC099521.3 | <0.001 | -1.245 | <0.001 |
| AC099522.1 | 0.004 | 0.421 | 0.016 |
| AC099560.1 | 0.009 | 0.588 | 0.032 |
| AC099568.1 | <0.001 | 0.803 | <0.001 |
| AC099568.2 | <0.001 | -0.750 | <0.001 |
| AC099778.2 | 0.004 | -0.386 | 0.017 |
| AC099786.1 | 0.011 | 0.483 | 0.037 |
| AC100756.2 | <0.001 | -1.780 | <0.001 |
| AC100778.2 | 0.009 | 0.340 | 0.032 |
| AC100797.1 | 0.011 | -0.449 | 0.037 |
| AC100812.1 | <0.001 | 0.643 | <0.001 |
| AC100854.1 | <0.001 | 0.411 | 0.004 |
| AC100872.2 | 0.008 | 0.643 | 0.030 |
| AC102953.1 | <0.001 | 0.674 | <0.001 |
| AC102953.2 | <0.001 | 0.348 | 0.001 |
| AC103563.2 | 0.006 | -0.898 | 0.025 |
| AC103563.7 | 0.003 | -0.837 | 0.013 |
| AC103739.2 | 0.014 | 0.384 | 0.047 |
| AC103746.1 | 0.006 | 0.394 | 0.024 |
| AC103957.2 | <0.001 | -1.122 | <0.001 |
| AC103974.1 | <0.001 | -0.585 | 0.003 |
| AC104046.1 | 0.001 | -0.288 | 0.007 |
| AC104078.1 | <0.001 | -1.997 | <0.001 |
| AC104083.1 | <0.001 | 0.306 | <0.001 |
| AC104117.3 | 0.001 | 0.621 | 0.007 |
| AC104212.3 | 0.010 | 0.308 | 0.036 |
| AC104316.1 | <0.001 | 0.704 | 0.002 |
| AC104333.3 | <0.001 | 0.558 | 0.003 |
| AC104411.2 | 0.003 | -0.349 | 0.015 |
| AC104461.1 | <0.001 | 1.031 | 0.002 |
| AC104532.1 | 0.003 | 0.483 | 0.014 |
| AC104561.1 | 0.001 | 0.466 | 0.007 |
| AC104561.2 | <0.001 | 0.987 | <0.001 |
| AC104561.3 | <0.001 | 0.808 | <0.001 |
| AC104590.1 | 0.011 | 0.540 | 0.039 |
| AC104596.1 | <0.001 | 0.426 | <0.001 |
| AC104650.2 | 0.001 | 0.482 | 0.006 |
| AC104667.1 | 0.002 | 0.838 | 0.008 |
| AC104687.2 | <0.001 | -1.275 | <0.001 |
| AC104695.1 | <0.001 | -0.804 | <0.001 |
| AC104763.1 | 0.015 | -0.368 | 0.048 |
| AC104819.3 | 0.002 | -0.303 | 0.011 |
| AC104958.2 | <0.001 | 0.613 | <0.001 |
| AC104964.1 | <0.001 | 1.199 | 0.002 |
| AC104964.3 | 0.007 | 0.547 | 0.027 |
| AC104964.4 | 0.002 | 0.999 | 0.008 |
| AC104971.1 | 0.010 | 0.308 | 0.036 |
| AC104986.1 | 0.012 | 0.342 | 0.041 |
| AC105105.1 | <0.001 | 0.916 | 0.003 |
| AC105206.2 | <0.001 | 0.307 | 0.001 |
| AC105219.4 | <0.001 | -0.336 | 0.006 |
| AC105235.1 | 0.007 | 0.462 | 0.027 |
| AC105389.2 | 0.015 | 0.642 | 0.048 |
| AC105460.1 | <0.001 | -1.030 | <0.001 |
| AC105460.2 | <0.001 | -1.158 | <0.001 |
| AC105942.1 | <0.001 | 0.354 | <0.001 |
| AC106738.1 | 0.012 | 0.474 | 0.042 |
| AC106786.2 | 0.003 | 0.389 | 0.015 |
| AC106801.1 | 0.010 | 0.486 | 0.036 |
| AC106865.1 | <0.001 | -1.285 | <0.001 |
| AC106872.2 | 0.015 | 0.329 | 0.050 |
| AC106881.1 | <0.001 | 1.190 | <0.001 |
| AC106886.2 | <0.001 | -0.390 | <0.001 |
| AC106897.2 | <0.001 | 0.521 | <0.001 |
| AC107021.1 | 0.001 | 0.556 | 0.006 |
| AC107021.2 | <0.001 | 0.637 | <0.001 |
| AC107027.1 | 0.004 | 0.517 | 0.018 |
| AC107032.1 | 0.005 | 0.265 | 0.019 |
| AC107072.2 | 0.013 | -0.617 | 0.043 |
| AC107204.1 | <0.001 | 0.839 | <0.001 |
| AC107294.2 | <0.001 | 0.498 | 0.002 |
| AC107294.3 | <0.001 | 0.581 | 0.005 |
| AC107308.1 | 0.015 | 0.478 | 0.048 |
| AC107959.1 | <0.001 | 0.355 | <0.001 |
| AC108058.1 | <0.001 | 0.405 | 0.002 |
| AC108134.1 | <0.001 | -0.932 | <0.001 |
| AC108174.1 | 0.004 | -0.893 | 0.016 |
| AC108215.1 | <0.001 | -0.955 | <0.001 |
| AC108463.1 | <0.001 | 0.471 | <0.001 |
| AC108463.2 | <0.001 | 0.403 | <0.001 |
| AC108463.3 | <0.001 | 0.700 | <0.001 |
| AC108681.1 | 0.003 | -0.619 | 0.013 |
| AC108734.2 | <0.001 | 0.494 | 0.004 |
| AC108751.4 | <0.001 | -0.846 | 0.003 |
| AC108860.2 | 0.001 | -0.313 | 0.007 |
| AC108925.1 | <0.001 | -0.437 | 0.005 |
| AC109347.1 | <0.001 | -0.333 | <0.001 |
| AC109449.1 | 0.007 | 0.364 | 0.027 |
| AC109454.1 | 0.008 | 0.367 | 0.029 |
| AC109454.4 | 0.008 | 0.359 | 0.028 |
| AC109479.1 | 0.002 | 0.423 | 0.009 |
| AC109587.1 | <0.001 | 0.294 | <0.001 |
| AC110373.1 | 0.006 | -0.386 | 0.024 |
| AC110597.1 | <0.001 | 0.688 | <0.001 |
| AC110619.1 | <0.001 | -0.869 | <0.001 |
| AC110771.1 | 0.004 | 0.461 | 0.017 |
| AC112184.1 | 0.012 | 0.440 | 0.041 |
| AC112236.1 | <0.001 | -1.294 | <0.001 |
| AC112236.2 | <0.001 | -0.408 | 0.002 |
| AC112484.4 | <0.001 | -0.776 | <0.001 |
| AC112715.1 | <0.001 | 0.945 | <0.001 |
| AC112721.1 | <0.001 | 0.828 | <0.001 |
| AC112721.2 | <0.001 | 0.833 | <0.001 |
| AC112722.1 | 0.011 | 0.351 | 0.038 |
| AC113346.1 | 0.008 | 0.374 | 0.030 |
| AC113398.1 | 0.005 | 0.274 | 0.019 |
| AC114267.1 | <0.001 | -1.126 | 0.002 |
| AC114284.1 | <0.001 | 0.722 | <0.001 |
| AC114488.1 | 0.006 | 0.455 | 0.022 |
| AC114489.2 | <0.001 | -0.446 | <0.001 |
| AC114490.1 | <0.001 | 0.302 | <0.001 |
| AC114760.2 | 0.002 | 0.423 | 0.008 |
| AC114947.1 | 0.005 | -0.738 | 0.019 |
| AC115284.2 | <0.001 | -0.650 | <0.001 |
| AC115285.1 | 0.006 | 0.474 | 0.024 |
| AC115522.1 | <0.001 | -0.551 | <0.001 |
| AC116021.1 | 0.002 | 0.598 | 0.009 |
| AC116049.1 | <0.001 | 0.354 | 0.005 |
| AC116312.1 | 0.001 | 0.563 | 0.007 |
| AC116366.1 | 0.011 | 0.358 | 0.037 |
| AC116366.2 | <0.001 | 0.466 | <0.001 |
| AC116447.1 | 0.013 | 0.283 | 0.044 |
| AC116535.2 | 0.003 | 0.345 | 0.012 |
| AC117395.1 | 0.006 | 0.461 | 0.024 |
| AC118754.1 | <0.001 | -1.408 | <0.001 |
| AC118754.2 | <0.001 | -0.900 | 0.003 |
| AC119044.1 | <0.001 | 0.455 | <0.001 |
| AC119396.1 | 0.013 | 0.491 | 0.043 |
| AC119674.1 | <0.001 | 0.721 | 0.002 |
| AC120036.1 | 0.004 | -0.728 | 0.016 |
| AC120036.3 | 0.008 | -0.611 | 0.031 |
| AC120036.4 | 0.009 | -0.640 | 0.034 |
| AC120057.3 | 0.003 | -0.329 | 0.013 |
| AC120057.4 | <0.001 | 0.657 | 0.002 |
| AC120193.1 | <0.001 | 1.645 | 0.002 |
| AC120498.4 | 0.003 | -0.733 | 0.015 |
| AC121247.2 | <0.001 | 0.571 | 0.005 |
| AC121493.1 | 0.002 | -0.389 | 0.009 |
| AC122685.1 | 0.009 | -0.528 | 0.033 |
| AC124067.2 | 0.002 | -0.383 | 0.011 |
| AC124068.2 | 0.001 | -0.289 | 0.008 |
| AC124248.1 | <0.001 | -0.632 | <0.001 |
| AC124312.2 | 0.007 | 0.553 | 0.026 |
| AC124947.1 | 0.005 | 0.586 | 0.020 |
| AC125494.2 | 0.010 | 0.329 | 0.035 |
| AC125807.1 | 0.001 | 0.335 | 0.007 |
| AC126544.1 | 0.002 | -0.570 | 0.009 |
| AC126773.3 | <0.001 | -0.990 | <0.001 |
| AC127496.5 | <0.001 | -0.928 | <0.001 |
| AC128709.1 | <0.001 | -1.263 | <0.001 |
| AC128709.2 | <0.001 | -1.217 | <0.001 |
| AC128709.3 | <0.001 | -1.110 | <0.001 |
| AC129492.1 | <0.001 | -1.016 | <0.001 |
| AC129492.6 | <0.001 | -2.044 | <0.001 |
| AC129507.1 | <0.001 | 0.819 | 0.002 |
| AC129926.1 | <0.001 | -0.928 | 0.004 |
| AC130371.2 | 0.002 | -0.273 | 0.010 |
| AC130686.1 | <0.001 | -0.824 | <0.001 |
| AC131097.3 | 0.010 | 0.367 | 0.035 |
| AC131254.1 | 0.001 | 0.784 | 0.006 |
| AC131392.1 | 0.004 | 0.447 | 0.017 |
| AC131888.1 | <0.001 | -1.019 | <0.001 |
| AC131953.2 | 0.010 | 0.473 | 0.036 |
| AC132219.2 | <0.001 | 0.306 | 0.004 |
| AC133065.3 | 0.015 | 0.294 | 0.049 |
| AC133134.1 | 0.009 | 0.275 | 0.033 |
| AC133550.3 | 0.011 | -0.271 | 0.038 |
| AC133785.1 | <0.001 | -0.978 | <0.001 |
| AC133919.2 | 0.004 | -0.449 | 0.016 |
| AC134043.2 | <0.001 | -0.808 | <0.001 |
| AC134043.3 | <0.001 | 0.515 | <0.001 |
| AC134312.5 | <0.001 | 0.587 | <0.001 |
| AC134669.1 | 0.006 | 0.455 | 0.023 |
| AC135050.2 | 0.003 | 0.311 | 0.014 |
| AC135178.3 | 0.009 | 0.323 | 0.033 |
| AC135178.6 | 0.001 | 0.341 | 0.007 |
| AC135507.1 | 0.003 | 0.313 | 0.014 |
| AC136475.1 | 0.010 | 0.340 | 0.037 |
| AC136628.3 | 0.001 | 0.287 | 0.008 |
| AC137932.2 | <0.001 | 0.584 | <0.001 |
| AC138028.6 | 0.010 | 0.285 | 0.034 |
| AC138207.1 | 0.002 | -0.504 | 0.010 |
| AC138207.2 | <0.001 | 0.427 | 0.003 |
| AC138207.4 | <0.001 | -0.437 | <0.001 |
| AC138207.7 | <0.001 | -0.602 | <0.001 |
| AC138305.1 | 0.001 | -0.715 | 0.006 |
| AC138392.1 | <0.001 | 0.273 | 0.001 |
| AC138409.2 | 0.009 | 0.275 | 0.033 |
| AC138696.1 | 0.003 | 0.510 | 0.012 |
| AC139099.1 | <0.001 | -0.624 | 0.003 |
| AC139495.2 | 0.003 | 0.454 | 0.015 |
| AC139720.1 | 0.003 | 0.610 | 0.013 |
| AC139769.1 | 0.002 | -0.633 | 0.008 |
| AC139769.2 | <0.001 | -2.463 | <0.001 |
| AC139795.1 | 0.001 | 0.496 | 0.007 |
| AC139795.2 | <0.001 | 0.269 | 0.003 |
| AC140479.3 | 0.013 | -0.525 | 0.044 |
| AC140479.4 | 0.006 | -0.931 | 0.024 |
| AC144536.1 | <0.001 | -0.681 | <0.001 |
| AC144831.1 | <0.001 | -0.516 | <0.001 |
| AC145098.2 | <0.001 | 0.575 | <0.001 |
| AC145124.1 | 0.009 | 0.404 | 0.034 |
| AC145676.1 | <0.001 | 0.887 | <0.001 |
| AC147067.1 | <0.001 | 0.350 | <0.001 |
| AC147651.1 | 0.006 | 0.512 | 0.024 |
| AC147651.4 | <0.001 | 0.396 | <0.001 |
| AC231657.1 | <0.001 | -0.448 | <0.001 |
| AC233280.2 | 0.006 | -0.456 | 0.024 |
| AC233699.1 | 0.002 | -0.422 | 0.011 |
| AC233723.2 | <0.001 | -0.410 | <0.001 |
| AC241377.4 | <0.001 | 0.624 | 0.002 |
| AC241585.1 | <0.001 | 0.314 | <0.001 |
| AC241952.2 | <0.001 | 0.668 | <0.001 |
| AC243562.2 | 0.003 | 0.527 | 0.013 |
| AC243772.2 | 0.014 | -0.370 | 0.046 |
| AC243829.2 | 0.006 | -0.814 | 0.024 |
| AC243829.4 | 0.004 | 0.463 | 0.017 |
| AC243919.2 | 0.010 | -0.325 | 0.034 |
| AC243965.2 | 0.008 | -0.466 | 0.031 |
| AC243967.2 | <0.001 | -1.229 | <0.001 |
| AC244034.1 | 0.006 | 0.270 | 0.024 |
| AC244153.1 | <0.001 | -0.439 | <0.001 |
| AC244205.1 | <0.001 | -0.932 | <0.001 |
| AC244230.1 | <0.001 | -0.806 | <0.001 |
| AC244669.1 | <0.001 | 0.431 | <0.001 |
| AC244669.2 | 0.002 | 0.781 | 0.008 |
| AC245100.2 | 0.002 | 0.393 | 0.008 |
| AC245100.4 | <0.001 | 0.463 | <0.001 |
| AC245297.1 | <0.001 | 0.447 | 0.003 |
| AC245297.4 | <0.001 | 0.501 | <0.001 |
| AC253536.4 | <0.001 | -0.993 | 0.003 |
| ACAN | <0.001 | 0.548 | 0.003 |
| ACBD3-AS1 | <0.001 | -0.720 | <0.001 |
| ACE | <0.001 | 0.296 | <0.001 |
| ACE2 | <0.001 | -0.527 | <0.001 |
| ACER1 | <0.001 | -1.896 | <0.001 |
| ACHE | <0.001 | 0.512 | <0.001 |
| ACKR2 | <0.001 | -0.814 | <0.001 |
| ACP7 | <0.001 | -1.122 | <0.001 |
| ACPP | <0.001 | -0.608 | <0.001 |
| ACSBG1 | <0.001 | -1.042 | 0.003 |
| ACSF2 | <0.001 | 0.339 | <0.001 |
| ACSM5 | <0.001 | 0.696 | 0.002 |
| ACSS3 | 0.001 | 0.641 | 0.007 |
| ACTA2-AS1 | <0.001 | 0.599 | <0.001 |
| ACTBL2 | 0.003 | 0.647 | 0.012 |
| ACTBP12 | 0.013 | 1.218 | 0.044 |
| ACTBP7 | <0.001 | 0.383 | <0.001 |
| ACTBP9 | <0.001 | 0.448 | 0.002 |
| ACTC1 | 0.012 | 0.460 | 0.042 |
| ACTG1P1 | 0.011 | 0.266 | 0.037 |
| ACTG1P12 | 0.006 | 0.461 | 0.023 |
| ACTG1P20 | 0.003 | 0.331 | 0.013 |
| ACTG1P24 | <0.001 | 0.595 | <0.001 |
| ACTG2 | <0.001 | 0.393 | 0.003 |
| ACTN1-AS1 | <0.001 | 0.820 | <0.001 |
| ACTN2 | 0.015 | 0.513 | 0.048 |
| ACTN3 | 0.014 | 0.903 | 0.046 |
| ACY3 | <0.001 | 0.386 | 0.001 |
| AD000864.1 | <0.001 | 0.381 | 0.005 |
| ADAD2 | <0.001 | -0.621 | <0.001 |
| ADAM12 | <0.001 | 0.366 | <0.001 |
| ADAM19 | <0.001 | 0.533 | <0.001 |
| ADAM20 | <0.001 | -0.689 | <0.001 |
| ADAM22 | <0.001 | 0.678 | 0.002 |
| ADAMDEC1 | 0.008 | 0.283 | 0.031 |
| ADAMTS10 | <0.001 | 0.410 | <0.001 |
| ADAMTS12 | <0.001 | 0.368 | <0.001 |
| ADAMTS13 | <0.001 | 0.425 | 0.004 |
| ADAMTS14 | <0.001 | 0.405 | <0.001 |
| ADAMTS15 | <0.001 | 0.466 | <0.001 |
| ADAMTS16 | <0.001 | 1.009 | <0.001 |
| ADAMTS17 | 0.001 | -0.331 | 0.007 |
| ADAMTS20 | 0.005 | -0.514 | 0.020 |
| ADAMTS3 | 0.003 | 0.384 | 0.014 |
| ADAMTS4 | <0.001 | 0.274 | <0.001 |
| ADAMTS5 | <0.001 | 0.458 | 0.002 |
| ADAMTS6 | <0.001 | 0.782 | <0.001 |
| ADAMTS7 | <0.001 | 0.381 | <0.001 |
| ADAMTS7P1 | 0.009 | 0.431 | 0.034 |
| ADAMTS7P3 | 0.001 | -0.651 | 0.008 |
| ADAMTS9 | 0.002 | 0.294 | 0.011 |
| ADAMTS9-AS2 | <0.001 | 0.542 | 0.002 |
| ADAP2 | <0.001 | -0.263 | <0.001 |
| ADARB2 | 0.010 | 0.726 | 0.037 |
| ADCY1 | 0.002 | 0.416 | 0.010 |
| ADCY2 | 0.005 | 0.614 | 0.020 |
| ADGRA2 | <0.001 | 0.281 | <0.001 |
| ADGRB1 | 0.014 | 0.523 | 0.047 |
| ADGRB2 | <0.001 | 0.432 | <0.001 |
| ADGRE4P | 0.015 | 0.317 | 0.048 |
| ADGRF1 | <0.001 | -0.797 | <0.001 |
| ADGRF2 | 0.004 | -0.437 | 0.016 |
| ADGRF5P2 | 0.006 | 0.486 | 0.024 |
| ADH1B | 0.014 | 0.782 | 0.046 |
| ADH7 | <0.001 | -0.718 | <0.001 |
| ADIRF-AS1 | <0.001 | -0.294 | <0.001 |
| ADORA1 | <0.001 | 0.722 | <0.001 |
| ADORA2A | 0.003 | 0.405 | 0.014 |
| ADPGK-AS1 | 0.004 | 0.351 | 0.016 |
| ADPRH | <0.001 | 0.268 | <0.001 |
| ADPRHL1 | <0.001 | 0.364 | <0.001 |
| ADRA1A | 0.009 | 0.980 | 0.034 |
| ADRA1B | <0.001 | 1.367 | <0.001 |
| ADRA1D | 0.005 | 0.476 | 0.022 |
| AF015262.1 | 0.001 | 0.924 | 0.006 |
| AF117829.1 | <0.001 | 0.369 | <0.001 |
| AF124730.2 | 0.009 | -0.541 | 0.033 |
| AF127577.4 | <0.001 | 0.409 | <0.001 |
| AF127936.5 | <0.001 | 0.595 | <0.001 |
| AF129075.3 | 0.001 | -0.382 | 0.006 |
| AF131215.7 | <0.001 | -0.613 | 0.002 |
| AF131216.1 | 0.003 | 0.347 | 0.013 |
| AF131216.3 | 0.005 | -0.629 | 0.022 |
| AF165147.1 | <0.001 | -1.089 | <0.001 |
| AF287957.1 | 0.003 | 0.605 | 0.012 |
| AFAP1 | <0.001 | 0.263 | <0.001 |
| AFAP1-AS1 | <0.001 | -0.674 | 0.001 |
| AFDN-DT | 0.005 | -0.293 | 0.020 |
| AFF3 | 0.002 | 0.707 | 0.011 |
| AGAP11 | 0.003 | -0.647 | 0.012 |
| AGBL1-AS1 | <0.001 | 0.686 | 0.005 |
| AGBL3 | 0.002 | 0.285 | 0.009 |
| AGR2 | 0.001 | -0.338 | 0.007 |
| AGTR1 | 0.001 | 0.643 | 0.006 |
| AHSG | 0.010 | -0.748 | 0.036 |
| AIF1L | <0.001 | -0.506 | <0.001 |
| AIRE | 0.002 | 2.061 | 0.008 |
| AJ011932.1 | <0.001 | 1.193 | <0.001 |
| AK2P2 | 0.010 | 0.465 | 0.036 |
| AK3P3 | <0.001 | 0.359 | 0.003 |
| AK7 | 0.004 | -0.435 | 0.016 |
| AKAP12 | <0.001 | 0.395 | <0.001 |
| AKAP2 | <0.001 | 0.660 | <0.001 |
| AKAP5 | <0.001 | 0.389 | 0.002 |
| AKAP6 | 0.006 | 0.456 | 0.024 |
| AKR1B10 | <0.001 | -0.410 | <0.001 |
| AKR1B10P1 | <0.001 | -0.786 | <0.001 |
| AKR1B15 | <0.001 | -0.302 | 0.004 |
| AKR1C1 | <0.001 | -0.391 | 0.003 |
| AKR1C2 | <0.001 | -0.449 | <0.001 |
| AKR1C3 | <0.001 | -0.373 | <0.001 |
| AKR1D1 | 0.001 | -0.822 | 0.008 |
| AKR7A3 | 0.013 | -0.354 | 0.044 |
| AL008628.1 | 0.008 | 0.534 | 0.029 |
| AL008718.3 | 0.002 | -0.403 | 0.008 |
| AL008729.1 | 0.012 | 0.278 | 0.040 |
| AL009178.2 | <0.001 | 0.501 | <0.001 |
| AL021328.1 | 0.005 | -0.670 | 0.021 |
| AL021408.1 | <0.001 | 1.312 | 0.002 |
| AL022067.1 | 0.004 | -0.320 | 0.018 |
| AL022315.1 | 0.001 | -0.872 | 0.006 |
| AL022323.1 | 0.012 | -0.585 | 0.040 |
| AL022323.3 | 0.007 | -0.370 | 0.027 |
| AL022334.1 | 0.010 | 0.727 | 0.036 |
| AL022342.1 | 0.006 | 0.470 | 0.024 |
| AL022476.1 | 0.007 | 0.298 | 0.027 |
| AL023653.1 | 0.013 | 0.339 | 0.043 |
| AL023653.2 | <0.001 | 0.401 | 0.004 |
| AL023807.1 | <0.001 | -0.345 | <0.001 |
| AL024508.1 | 0.002 | -0.271 | 0.010 |
| AL031008.1 | 0.009 | 0.768 | 0.033 |
| AL031055.1 | <0.001 | -0.403 | <0.001 |
| AL031058.1 | <0.001 | -0.330 | <0.001 |
| AL031123.1 | <0.001 | -0.474 | <0.001 |
| AL031283.1 | <0.001 | 0.623 | <0.001 |
| AL031429.2 | 0.014 | -0.852 | 0.045 |
| AL031577.1 | 0.015 | 0.314 | 0.048 |
| AL031599.1 | 0.002 | -0.917 | 0.011 |
| AL031651.2 | <0.001 | 0.971 | <0.001 |
| AL031667.3 | <0.001 | -0.383 | 0.001 |
| AL031719.1 | 0.012 | -0.463 | 0.040 |
| AL031777.1 | <0.001 | -0.481 | 0.003 |
| AL031846.1 | <0.001 | 0.781 | <0.001 |
| AL033384.1 | <0.001 | -0.800 | <0.001 |
| AL033384.2 | 0.011 | -0.287 | 0.038 |
| AL033397.1 | 0.010 | -0.744 | 0.037 |
| AL033397.2 | 0.015 | -0.363 | 0.049 |
| AL033523.1 | <0.001 | -1.436 | <0.001 |
| AL034374.1 | 0.007 | 0.364 | 0.027 |
| AL034376.1 | 0.011 | -0.524 | 0.039 |
| AL034376.2 | <0.001 | -1.734 | <0.001 |
| AL034397.2 | <0.001 | -0.901 | 0.002 |
| AL034417.3 | <0.001 | 0.556 | <0.001 |
| AL034550.1 | <0.001 | 0.766 | <0.001 |
| AL034550.2 | 0.002 | 0.825 | 0.011 |
| AL035258.1 | <0.001 | -0.756 | 0.003 |
| AL035417.2 | 0.001 | 0.976 | 0.008 |
| AL035448.1 | <0.001 | 0.472 | 0.004 |
| AL035458.2 | 0.004 | 0.456 | 0.016 |
| AL035530.2 | <0.001 | 0.414 | <0.001 |
| AL035661.1 | <0.001 | -0.341 | <0.001 |
| AL035701.1 | 0.008 | 0.959 | 0.029 |
| AL049612.1 | 0.002 | 1.099 | 0.008 |
| AL049629.1 | <0.001 | -0.534 | 0.004 |
| AL049634.1 | 0.002 | 0.673 | 0.012 |
| AL049747.1 | 0.011 | -0.783 | 0.038 |
| AL049775.1 | 0.004 | 0.453 | 0.018 |
| AL049834.1 | <0.001 | 0.332 | <0.001 |
| AL049838.1 | 0.005 | 0.446 | 0.019 |
| AL049839.2 | <0.001 | 0.994 | 0.003 |
| AL049870.3 | 0.003 | -0.698 | 0.013 |
| AL049871.1 | 0.005 | 0.489 | 0.021 |
| AL049874.2 | <0.001 | 0.811 | <0.001 |
| AL050309.1 | <0.001 | -0.265 | <0.001 |
| AL050321.1 | 0.015 | -0.523 | 0.048 |
| AL050344.1 | 0.015 | 0.610 | 0.048 |
| AL078621.3 | 0.005 | 0.297 | 0.020 |
| AL079307.1 | 0.005 | -0.821 | 0.021 |
| AL080243.2 | 0.004 | 0.357 | 0.016 |
| AL096711.2 | <0.001 | 1.040 | <0.001 |
| AL096865.1 | <0.001 | 0.569 | <0.001 |
| AL109741.1 | 0.014 | 0.266 | 0.046 |
| AL109804.1 | 0.003 | 0.432 | 0.012 |
| AL109809.1 | <0.001 | 0.840 | 0.001 |
| AL109811.1 | 0.006 | 0.303 | 0.024 |
| AL109924.1 | <0.001 | 1.212 | <0.001 |
| AL109976.1 | <0.001 | -0.540 | 0.002 |
| AL117209.1 | 0.001 | -0.306 | 0.006 |
| AL117327.1 | 0.002 | 0.523 | 0.008 |
| AL118508.3 | 0.002 | 0.439 | 0.011 |
| AL121672.3 | 0.002 | -0.343 | 0.008 |
| AL121748.1 | <0.001 | 0.759 | 0.002 |
| AL121761.1 | <0.001 | -0.847 | <0.001 |
| AL121787.1 | 0.002 | 0.643 | 0.012 |
| AL121820.1 | <0.001 | 0.407 | 0.004 |
| AL121820.2 | 0.001 | -0.650 | 0.008 |
| AL121832.1 | 0.002 | -0.722 | 0.008 |
| AL121890.4 | <0.001 | 0.568 | 0.002 |
| AL121890.5 | 0.009 | 0.474 | 0.034 |
| AL121894.2 | 0.006 | 0.265 | 0.024 |
| AL121895.1 | <0.001 | -1.051 | <0.001 |
| AL121895.2 | <0.001 | -0.651 | <0.001 |
| AL122023.3 | <0.001 | -0.332 | <0.001 |
| AL122034.1 | 0.012 | -0.630 | 0.041 |
| AL132639.2 | <0.001 | -0.319 | <0.001 |
| AL133153.2 | 0.002 | -0.410 | 0.010 |
| AL133163.1 | <0.001 | -0.849 | <0.001 |
| AL133338.2 | 0.013 | 0.299 | 0.044 |
| AL133346.1 | <0.001 | 0.977 | <0.001 |
| AL133352.1 | 0.009 | 0.396 | 0.034 |
| AL133371.2 | <0.001 | 0.388 | 0.002 |
| AL133410.1 | 0.003 | 0.802 | 0.014 |
| AL133415.1 | <0.001 | 0.791 | <0.001 |
| AL133517.1 | <0.001 | 0.366 | <0.001 |
| AL135745.1 | 0.005 | -0.347 | 0.020 |
| AL135786.1 | <0.001 | 0.601 | 0.003 |
| AL135791.1 | 0.004 | -0.372 | 0.016 |
| AL135910.1 | <0.001 | 0.551 | <0.001 |
| AL136115.2 | 0.009 | 0.373 | 0.032 |
| AL136164.3 | 0.002 | 0.685 | 0.008 |
| AL136418.3 | <0.001 | -1.030 | 0.003 |
| AL136531.2 | <0.001 | -0.511 | <0.001 |
| AL136531.3 | <0.001 | -0.540 | 0.003 |
| AL136968.1 | <0.001 | 0.415 | 0.004 |
| AL136982.1 | 0.006 | -0.505 | 0.023 |
| AL136982.5 | <0.001 | -1.508 | <0.001 |
| AL136982.6 | <0.001 | -1.687 | <0.001 |
| AL136988.2 | <0.001 | 0.512 | <0.001 |
| AL137026.1 | 0.001 | 0.946 | 0.007 |
| AL137058.2 | <0.001 | 0.295 | <0.001 |
| AL137186.1 | <0.001 | 0.727 | <0.001 |
| AL137191.1 | 0.013 | -0.572 | 0.044 |
| AL137247.1 | 0.002 | 0.607 | 0.011 |
| AL137779.2 | <0.001 | 0.457 | <0.001 |
| AL137784.1 | <0.001 | 0.427 | <0.001 |
| AL138720.1 | <0.001 | -0.810 | 0.004 |
| AL138760.1 | <0.001 | -1.349 | <0.001 |
| AL138767.3 | <0.001 | 1.004 | 0.003 |
| AL138789.1 | <0.001 | -0.754 | <0.001 |
| AL138820.1 | 0.008 | 0.284 | 0.030 |
| AL138878.2 | 0.002 | -1.389 | 0.010 |
| AL138899.1 | 0.004 | 0.741 | 0.016 |
| AL138899.2 | <0.001 | 0.809 | 0.001 |
| AL138995.1 | <0.001 | 0.398 | 0.005 |
| AL138999.1 | <0.001 | 0.896 | <0.001 |
| AL139005.1 | 0.004 | 0.352 | 0.016 |
| AL139039.3 | 0.006 | -0.723 | 0.024 |
| AL139095.1 | <0.001 | -0.528 | <0.001 |
| AL139125.1 | <0.001 | 0.674 | <0.001 |
| AL139147.1 | <0.001 | 1.046 | <0.001 |
| AL139220.2 | <0.001 | 0.574 | <0.001 |
| AL139246.5 | <0.001 | -0.359 | <0.001 |
| AL139351.1 | <0.001 | 1.084 | <0.001 |
| AL139383.1 | 0.004 | 0.603 | 0.016 |
| AL139385.1 | <0.001 | -0.867 | <0.001 |
| AL139393.2 | <0.001 | -0.594 | 0.003 |
| AL139393.3 | <0.001 | 0.319 | <0.001 |
| AL139423.1 | <0.001 | -0.338 | 0.003 |
| AL157373.2 | <0.001 | -0.624 | 0.002 |
| AL157394.1 | <0.001 | 0.471 | <0.001 |
| AL157770.1 | 0.002 | 0.612 | 0.011 |
| AL157786.1 | 0.006 | 0.319 | 0.023 |
| AL157935.2 | 0.002 | -0.440 | 0.009 |
| AL158071.1 | <0.001 | -0.734 | <0.001 |
| AL158071.3 | 0.002 | 0.594 | 0.010 |
| AL158071.4 | 0.009 | 0.509 | 0.033 |
| AL158071.5 | 0.008 | 0.313 | 0.029 |
| AL158151.1 | 0.003 | 0.821 | 0.013 |
| AL158166.2 | 0.007 | 0.524 | 0.027 |
| AL158207.1 | <0.001 | -0.907 | <0.001 |
| AL158801.2 | 0.004 | -0.407 | 0.019 |
| AL159169.2 | <0.001 | -0.511 | 0.001 |
| AL159169.3 | <0.001 | -0.874 | <0.001 |
| AL160191.3 | 0.004 | 0.859 | 0.016 |
| AL160313.1 | <0.001 | -0.890 | <0.001 |
| AL160313.2 | <0.001 | -0.449 | 0.003 |
| AL160408.1 | <0.001 | -0.629 | <0.001 |
| AL160408.3 | <0.001 | -1.324 | <0.001 |
| AL160412.1 | <0.001 | -0.885 | 0.002 |
| AL161636.1 | <0.001 | -1.589 | <0.001 |
| AL161645.1 | <0.001 | -0.820 | <0.001 |
| AL161908.1 | 0.011 | 0.695 | 0.038 |
| AL161909.1 | 0.003 | 0.411 | 0.013 |
| AL162171.1 | 0.010 | -0.270 | 0.035 |
| AL162231.1 | <0.001 | 0.448 | 0.002 |
| AL162231.4 | <0.001 | 0.460 | 0.004 |
| AL162376.1 | <0.001 | -1.518 | <0.001 |
| AL162424.1 | <0.001 | -0.559 | <0.001 |
| AL162431.1 | <0.001 | 0.279 | 0.004 |
| AL162457.2 | 0.003 | 0.893 | 0.015 |
| AL163636.3 | 0.002 | 0.477 | 0.012 |
| AL353150.1 | 0.001 | 0.432 | 0.008 |
| AL353719.1 | 0.014 | -0.264 | 0.045 |
| AL353746.1 | 0.004 | 0.967 | 0.017 |
| AL353768.1 | <0.001 | -0.990 | <0.001 |
| AL353997.2 | <0.001 | -1.482 | <0.001 |
| AL354696.2 | <0.001 | 0.430 | <0.001 |
| AL354751.1 | <0.001 | 0.931 | 0.003 |
| AL354754.1 | 0.004 | -1.093 | 0.016 |
| AL354811.1 | <0.001 | 0.841 | <0.001 |
| AL354813.1 | <0.001 | 0.604 | <0.001 |
| AL354893.2 | 0.011 | 0.429 | 0.038 |
| AL354919.2 | 0.002 | 0.477 | 0.008 |
| AL354977.1 | 0.002 | 0.644 | 0.011 |
| AL355102.1 | 0.005 | -0.450 | 0.020 |
| AL355102.2 | 0.009 | -0.326 | 0.034 |
| AL355102.4 | 0.013 | -0.369 | 0.044 |
| AL355297.3 | <0.001 | 0.428 | 0.005 |
| AL355303.1 | 0.006 | -0.639 | 0.025 |
| AL355312.4 | <0.001 | -0.835 | <0.001 |
| AL355355.2 | <0.001 | 0.409 | <0.001 |
| AL355490.3 | 0.001 | 0.632 | 0.008 |
| AL355581.1 | <0.001 | 0.488 | <0.001 |
| AL355596.1 | 0.004 | 1.444 | 0.018 |
| AL355864.1 | 0.002 | 0.396 | 0.011 |
| AL355922.1 | 0.009 | 0.496 | 0.032 |
| AL355987.3 | 0.001 | 0.502 | 0.007 |
| AL356056.2 | <0.001 | 0.505 | 0.004 |
| AL356124.1 | 0.002 | 0.460 | 0.012 |
| AL356215.1 | <0.001 | -0.509 | <0.001 |
| AL356234.2 | <0.001 | -0.875 | <0.001 |
| AL356235.1 | <0.001 | -0.302 | 0.003 |
| AL356273.6 | <0.001 | 0.400 | 0.001 |
| AL356292.1 | <0.001 | -0.694 | 0.005 |
| AL356417.2 | 0.001 | 0.641 | 0.006 |
| AL356585.1 | 0.005 | 0.542 | 0.020 |
| AL356599.1 | <0.001 | 0.495 | <0.001 |
| AL356750.1 | <0.001 | 0.605 | 0.003 |
| AL356867.1 | <0.001 | -1.832 | <0.001 |
| AL357033.1 | <0.001 | -1.718 | <0.001 |
| AL357033.3 | <0.001 | -0.323 | 0.002 |
| AL357033.4 | <0.001 | -0.291 | <0.001 |
| AL357054.4 | 0.001 | 0.433 | 0.006 |
| AL357143.1 | 0.001 | -0.976 | 0.007 |
| AL357153.1 | <0.001 | -0.995 | 0.002 |
| AL357673.2 | 0.003 | 0.955 | 0.014 |
| AL357992.1 | <0.001 | 0.547 | <0.001 |
| AL358332.1 | 0.001 | -0.460 | 0.006 |
| AL358473.1 | 0.013 | 0.531 | 0.043 |
| AL358852.1 | <0.001 | -0.362 | <0.001 |
| AL359183.1 | <0.001 | 0.346 | 0.002 |
| AL359220.1 | <0.001 | -0.363 | <0.001 |
| AL359263.1 | <0.001 | 0.562 | 0.002 |
| AL359504.2 | <0.001 | 0.658 | <0.001 |
| AL359532.1 | <0.001 | 0.387 | <0.001 |
| AL359692.1 | <0.001 | -0.957 | <0.001 |
| AL359704.1 | 0.009 | 0.411 | 0.033 |
| AL359979.1 | <0.001 | -1.840 | <0.001 |
| AL360181.3 | <0.001 | -0.498 | <0.001 |
| AL360181.4 | <0.001 | -0.771 | 0.002 |
| AL360270.1 | <0.001 | -0.325 | 0.003 |
| AL360270.2 | 0.003 | -0.353 | 0.013 |
| AL360270.3 | 0.009 | -0.269 | 0.034 |
| AL365203.2 | <0.001 | 0.390 | <0.001 |
| AL365226.1 | <0.001 | -1.201 | <0.001 |
| AL365475.1 | <0.001 | 0.582 | 0.004 |
| AL390718.1 | <0.001 | -1.675 | <0.001 |
| AL390955.2 | <0.001 | 0.394 | <0.001 |
| AL391069.2 | <0.001 | 0.399 | 0.001 |
| AL391094.1 | 0.005 | -0.474 | 0.021 |
| AL391244.1 | 0.009 | -0.275 | 0.031 |
| AL391422.1 | 0.015 | 0.283 | 0.049 |
| AL391704.1 | <0.001 | 1.315 | 0.004 |
| AL391832.3 | <0.001 | 0.474 | 0.005 |
| AL391903.1 | 0.011 | 0.575 | 0.038 |
| AL442125.1 | 0.003 | 0.344 | 0.013 |
| AL442125.2 | 0.001 | 0.394 | 0.008 |
| AL445187.1 | 0.006 | -0.328 | 0.025 |
| AL445307.1 | 0.002 | 0.830 | 0.008 |
| AL445423.1 | 0.003 | 0.955 | 0.013 |
| AL445437.1 | 0.006 | -0.709 | 0.024 |
| AL445471.1 | <0.001 | 0.690 | <0.001 |
| AL445531.1 | <0.001 | -1.203 | <0.001 |
| AL449106.1 | <0.001 | -0.548 | 0.005 |
| AL450326.2 | 0.014 | 0.945 | 0.047 |
| AL450992.1 | 0.005 | 0.528 | 0.021 |
| AL451007.1 | 0.009 | 0.302 | 0.034 |
| AL451042.1 | <0.001 | -0.662 | <0.001 |
| AL451042.2 | <0.001 | -0.513 | <0.001 |
| AL451064.1 | 0.010 | 0.486 | 0.036 |
| AL451064.2 | 0.005 | 0.574 | 0.021 |
| AL451074.6 | 0.005 | 0.437 | 0.021 |
| AL451139.1 | <0.001 | -0.662 | <0.001 |
| AL500527.1 | 0.003 | 0.591 | 0.013 |
| AL512274.1 | <0.001 | -0.587 | <0.001 |
| AL512306.3 | 0.005 | 0.694 | 0.019 |
| AL512310.8 | 0.006 | 0.622 | 0.025 |
| AL512326.5 | <0.001 | -0.375 | <0.001 |
| AL512329.2 | 0.004 | 0.428 | 0.017 |
| AL512363.1 | <0.001 | -0.480 | <0.001 |
| AL512625.2 | <0.001 | -0.529 | 0.004 |
| AL512631.1 | <0.001 | -1.174 | <0.001 |
| AL512785.2 | 0.007 | -0.464 | 0.027 |
| AL513122.2 | <0.001 | -0.936 | <0.001 |
| AL513165.2 | 0.006 | 0.398 | 0.022 |
| AL513304.1 | 0.003 | -0.746 | 0.014 |
| AL513318.2 | 0.002 | 0.366 | 0.009 |
| AL513327.1 | <0.001 | 0.350 | 0.002 |
| AL513365.1 | 0.005 | 0.292 | 0.022 |
| AL513477.1 | 0.003 | 0.411 | 0.013 |
| AL513485.1 | <0.001 | -0.987 | <0.001 |
| AL583722.1 | 0.014 | -0.470 | 0.046 |
| AL583824.1 | 0.003 | -0.479 | 0.012 |
| AL589745.1 | <0.001 | 1.164 | <0.001 |
| AL589765.4 | 0.001 | 0.351 | 0.006 |
| AL589765.5 | 0.003 | 0.527 | 0.013 |
| AL589986.2 | <0.001 | -1.211 | <0.001 |
| AL589993.1 | 0.007 | 0.458 | 0.026 |
| AL590004.3 | 0.001 | 1.541 | 0.006 |
| AL590006.1 | 0.007 | 0.427 | 0.025 |
| AL590399.4 | 0.001 | 0.775 | 0.006 |
| AL590438.1 | <0.001 | 0.564 | 0.002 |
| AL590560.2 | <0.001 | -1.281 | <0.001 |
| AL590617.1 | 0.004 | -0.509 | 0.018 |
| AL590652.1 | <0.001 | -0.540 | <0.001 |
| AL590666.3 | <0.001 | -0.815 | 0.003 |
| AL590762.1 | 0.014 | 0.320 | 0.047 |
| AL590764.1 | <0.001 | 0.436 | 0.003 |
| AL591135.1 | <0.001 | 0.376 | <0.001 |
| AL591501.1 | <0.001 | -0.965 | <0.001 |
| AL592293.2 | 0.012 | 0.544 | 0.040 |
| AL592429.1 | 0.002 | 0.552 | 0.009 |
| AL592494.2 | <0.001 | 0.811 | 0.003 |
| AL596244.1 | <0.001 | 0.741 | <0.001 |
| AL596275.1 | 0.013 | 0.312 | 0.043 |
| AL603766.1 | 0.003 | 0.628 | 0.013 |
| AL606469.1 | 0.002 | 0.479 | 0.011 |
| AL606490.2 | 0.011 | 0.563 | 0.037 |
| AL606491.1 | <0.001 | 0.426 | <0.001 |
| AL606534.1 | 0.001 | 0.469 | 0.006 |
| AL606807.1 | <0.001 | 1.128 | 0.002 |
| AL627171.2 | <0.001 | -0.576 | 0.001 |
| AL627309.5 | 0.012 | -0.337 | 0.040 |
| AL627309.6 | 0.003 | -0.263 | 0.015 |
| AL627309.7 | 0.003 | -0.270 | 0.013 |
| AL627389.1 | 0.009 | 0.309 | 0.034 |
| AL645608.3 | <0.001 | -0.803 | 0.002 |
| AL645608.8 | 0.009 | 0.418 | 0.033 |
| AL645933.1 | <0.001 | 0.541 | <0.001 |
| AL645939.2 | 0.001 | 0.440 | 0.006 |
| AL645939.3 | 0.008 | 0.562 | 0.029 |
| AL645939.4 | <0.001 | 0.830 | <0.001 |
| AL662907.2 | <0.001 | -0.346 | 0.002 |
| AL671277.2 | <0.001 | 0.307 | 0.003 |
| AL672207.1 | <0.001 | -0.450 | 0.001 |
| AL683807.1 | <0.001 | 0.522 | 0.002 |
| AL683842.1 | <0.001 | 0.681 | <0.001 |
| AL691403.2 | 0.005 | -0.304 | 0.022 |
| AL691432.2 | <0.001 | -0.286 | <0.001 |
| AL691482.4 | <0.001 | -0.679 | <0.001 |
| AL731566.3 | <0.001 | 0.771 | 0.003 |
| AL731684.1 | <0.001 | -1.492 | <0.001 |
| AL731733.1 | <0.001 | 0.784 | <0.001 |
| AL732414.1 | 0.003 | -0.341 | 0.013 |
| AL844908.1 | 0.008 | 0.264 | 0.031 |
| AL845472.2 | 0.002 | -0.533 | 0.010 |
| AL928654.1 | 0.012 | 0.282 | 0.040 |
| AL929236.1 | <0.001 | 0.581 | <0.001 |
| ALDH1B1 | <0.001 | 0.326 | <0.001 |
| ALDH3A1 | <0.001 | -0.591 | <0.001 |
| ALDH3B2 | <0.001 | -0.352 | <0.001 |
| ALDH5A1 | <0.001 | -0.331 | <0.001 |
| ALG1L12P | 0.015 | 0.438 | 0.048 |
| ALG1L3P | 0.015 | -0.760 | 0.048 |
| ALMS1P1 | <0.001 | 0.465 | 0.004 |
| ALOX12 | <0.001 | -0.687 | <0.001 |
| ALOX12B | <0.001 | -1.277 | <0.001 |
| ALOX15B | 0.003 | -0.335 | 0.012 |
| ALOX15P1 | 0.007 | -0.309 | 0.027 |
| ALOXE3 | <0.001 | -1.015 | <0.001 |
| ALPK2 | <0.001 | 0.825 | <0.001 |
| ALX4 | 0.005 | 0.828 | 0.020 |
| AMIGO2 | <0.001 | 0.298 | <0.001 |
| AMN | <0.001 | -0.661 | <0.001 |
| AMPH | <0.001 | 0.767 | <0.001 |
| AMTN | <0.001 | 0.459 | 0.003 |
| AMZ1 | 0.015 | 0.506 | 0.050 |
| ANG | <0.001 | 0.287 | <0.001 |
| ANGPT1 | <0.001 | 0.485 | <0.001 |
| ANGPTL1 | 0.010 | 0.468 | 0.035 |
| ANGPTL7 | 0.006 | 0.763 | 0.023 |
| ANK2 | 0.002 | 0.423 | 0.011 |
| ANK3 | <0.001 | -0.330 | <0.001 |
| ANKFN1 | 0.005 | -0.732 | 0.022 |
| ANKK1 | <0.001 | -0.505 | <0.001 |
| ANKRD10-IT1 | <0.001 | 0.269 | <0.001 |
| ANKRD18CP | 0.001 | 0.821 | 0.007 |
| ANKRD22 | <0.001 | -0.386 | <0.001 |
| ANKRD23 | 0.015 | 0.386 | 0.050 |
| ANKRD31 | <0.001 | -1.256 | <0.001 |
| ANKRD33B | <0.001 | 0.551 | <0.001 |
| ANKRD34A | <0.001 | 0.366 | <0.001 |
| ANKRD35 | <0.001 | -0.837 | <0.001 |
| ANKRD44 | <0.001 | 0.310 | 0.003 |
| ANKRD55 | 0.009 | 0.409 | 0.033 |
| ANKRD61 | <0.001 | 0.296 | 0.002 |
| ANKRD62 | 0.007 | -0.743 | 0.025 |
| ANKRD7 | <0.001 | 0.921 | <0.001 |
| ANO4 | <0.001 | 1.129 | <0.001 |
| ANO5 | 0.005 | 0.701 | 0.020 |
| ANO7L1 | <0.001 | -0.374 | <0.001 |
| ANTXR2 | <0.001 | 0.446 | <0.001 |
| ANTXRLP1 | 0.015 | 0.560 | 0.048 |
| ANXA13 | <0.001 | 0.873 | 0.001 |
| ANXA6 | <0.001 | 0.287 | <0.001 |
| ANXA9 | <0.001 | -0.724 | <0.001 |
| AOC3 | <0.001 | 0.281 | 0.004 |
| AOC4P | 0.014 | 0.587 | 0.046 |
| AP000221.1 | 0.015 | -0.827 | 0.048 |
| AP000229.1 | <0.001 | 0.785 | <0.001 |
| AP000265.1 | 0.003 | 0.510 | 0.014 |
| AP000295.10 | 0.010 | 0.290 | 0.035 |
| AP000317.2 | <0.001 | 0.875 | <0.001 |
| AP000344.1 | 0.002 | 1.282 | 0.011 |
| AP000345.2 | 0.015 | 0.483 | 0.049 |
| AP000679.1 | <0.001 | -0.957 | <0.001 |
| AP000688.1 | <0.001 | -0.451 | 0.002 |
| AP000695.1 | <0.001 | 0.566 | <0.001 |
| AP000695.2 | <0.001 | 0.580 | <0.001 |
| AP000695.3 | <0.001 | 0.711 | <0.001 |
| AP000704.2 | 0.003 | 0.418 | 0.014 |
| AP000769.1 | <0.001 | -0.337 | <0.001 |
| AP000783.1 | <0.001 | 0.558 | <0.001 |
| AP000802.1 | 0.006 | 0.925 | 0.023 |
| AP000873.3 | <0.001 | 0.618 | 0.003 |
| AP000880.1 | 0.010 | -0.459 | 0.035 |
| AP000892.4 | <0.001 | 0.511 | <0.001 |
| AP000894.4 | <0.001 | -0.347 | 0.005 |
| AP000925.1 | <0.001 | -0.751 | 0.005 |
| AP000941.1 | <0.001 | 0.896 | <0.001 |
| AP000943.1 | 0.001 | -0.633 | 0.006 |
| AP001010.1 | 0.010 | 0.485 | 0.037 |
| AP001062.1 | 0.011 | 0.278 | 0.039 |
| AP001065.3 | 0.002 | 0.426 | 0.009 |
| AP001094.2 | 0.001 | 0.630 | 0.007 |
| AP001148.1 | <0.001 | 0.634 | <0.001 |
| AP001189.1 | 0.004 | 0.395 | 0.018 |
| AP001189.3 | <0.001 | 0.450 | 0.002 |
| AP001207.3 | <0.001 | -0.518 | <0.001 |
| AP001269.1 | 0.003 | -0.616 | 0.015 |
| AP001360.1 | 0.002 | -1.098 | 0.011 |
| AP001434.1 | 0.002 | 0.406 | 0.009 |
| AP001471.1 | 0.001 | 0.494 | 0.006 |
| AP001528.1 | <0.001 | 0.641 | <0.001 |
| AP001528.2 | <0.001 | 0.514 | <0.001 |
| AP001533.1 | 0.012 | 1.036 | 0.040 |
| AP001542.3 | <0.001 | -0.333 | <0.001 |
| AP001922.6 | 0.003 | 0.423 | 0.013 |
| AP001962.1 | <0.001 | -0.872 | <0.001 |
| AP001994.1 | 0.007 | 1.591 | 0.026 |
| AP002008.1 | <0.001 | -0.790 | 0.002 |
| AP002449.1 | 0.002 | -0.353 | 0.011 |
| AP002490.1 | 0.008 | 0.334 | 0.029 |
| AP002498.1 | <0.001 | -0.933 | 0.002 |
| AP002505.2 | 0.014 | 0.466 | 0.046 |
| AP002761.2 | 0.003 | 0.650 | 0.014 |
| AP002800.1 | <0.001 | -0.829 | <0.001 |
| AP002812.3 | 0.007 | 0.487 | 0.026 |
| AP002812.5 | 0.007 | 0.412 | 0.025 |
| AP002852.1 | 0.003 | 0.656 | 0.015 |
| AP002989.1 | <0.001 | 0.698 | 0.003 |
| AP003031.3 | 0.004 | -0.553 | 0.016 |
| AP003059.1 | <0.001 | 0.861 | 0.004 |
| AP003068.2 | <0.001 | -0.421 | <0.001 |
| AP003068.4 | <0.001 | -1.447 | <0.001 |
| AP003108.5 | 0.001 | 0.707 | 0.006 |
| AP003119.2 | <0.001 | -0.371 | <0.001 |
| AP003170.1 | 0.014 | 0.364 | 0.046 |
| AP003500.1 | <0.001 | -1.271 | <0.001 |
| AP003555.1 | <0.001 | 0.801 | <0.001 |
| AP003555.2 | 0.002 | 0.972 | 0.010 |
| AP003680.1 | 0.013 | -0.376 | 0.043 |
| AP003721.1 | <0.001 | -0.563 | <0.001 |
| AP003721.2 | 0.004 | -0.491 | 0.015 |
| AP004245.1 | 0.002 | 0.448 | 0.010 |
| AP004609.3 | 0.001 | 0.877 | 0.007 |
| AP004609.5 | 0.014 | -0.270 | 0.045 |
| AP005019.1 | 0.003 | 0.476 | 0.015 |
| AP005131.1 | 0.010 | 0.617 | 0.036 |
| AP005131.2 | 0.008 | 0.434 | 0.031 |
| AP005136.3 | 0.014 | -0.266 | 0.047 |
| AP005329.2 | 0.002 | -0.282 | 0.011 |
| AP005432.2 | <0.001 | -0.506 | <0.001 |
| AP005482.2 | 0.001 | -0.553 | 0.006 |
| AP006621.4 | <0.001 | -0.473 | 0.004 |
| AP006623.1 | <0.001 | 0.427 | 0.004 |
| AP3B2 | <0.001 | 0.708 | 0.002 |
| APBB1 | <0.001 | 0.355 | <0.001 |
| APBB1IP | <0.001 | 0.385 | <0.001 |
| APCDD1L | <0.001 | 0.511 | <0.001 |
| APELA | 0.005 | -0.498 | 0.020 |
| APLN | <0.001 | 0.313 | <0.001 |
| APLP1 | 0.002 | 0.371 | 0.009 |
| APOBEC2 | 0.009 | 0.554 | 0.033 |
| APOBEC3A | <0.001 | -0.429 | <0.001 |
| APOBEC3F | <0.001 | 0.318 | <0.001 |
| APOBEC3G | <0.001 | 0.376 | <0.001 |
| APOBEC3H | 0.008 | 0.300 | 0.031 |
| APOC2 | 0.011 | 0.457 | 0.037 |
| AQP5 | 0.002 | -0.642 | 0.010 |
| AR | 0.005 | 0.629 | 0.019 |
| ARC | 0.010 | -0.394 | 0.035 |
| ARF4-AS1 | 0.005 | 0.300 | 0.021 |
| ARG1 | <0.001 | -2.283 | <0.001 |
| ARHGAP15 | <0.001 | 0.385 | 0.001 |
| ARHGAP22 | <0.001 | 0.493 | <0.001 |
| ARHGAP25 | <0.001 | 0.309 | <0.001 |
| ARHGAP27 | <0.001 | -0.329 | <0.001 |
| ARHGAP27P1-BPTFP1-KPNA2P3 | <0.001 | -0.312 | <0.001 |
| ARHGAP27P2 | <0.001 | -0.703 | 0.004 |
| ARHGAP29 | <0.001 | 0.609 | <0.001 |
| ARHGAP31 | <0.001 | 0.341 | <0.001 |
| ARHGAP32 | <0.001 | -0.301 | <0.001 |
| ARHGAP33 | <0.001 | 0.280 | 0.006 |
| ARHGAP36 | 0.007 | 0.902 | 0.027 |
| ARHGAP40 | <0.001 | -0.724 | <0.001 |
| ARHGAP42 | <0.001 | 0.293 | 0.001 |
| ARHGAP6 | <0.001 | 0.446 | <0.001 |
| ARHGAP9 | <0.001 | 0.296 | <0.001 |
| ARHGEF10 | <0.001 | 0.337 | <0.001 |
| ARHGEF17 | <0.001 | 0.272 | <0.001 |
| ARHGEF26 | <0.001 | -0.544 | <0.001 |
| ARHGEF26-AS1 | 0.005 | -0.866 | 0.019 |
| ARHGEF35-AS1 | 0.001 | -0.538 | 0.006 |
| ARHGEF40 | <0.001 | 0.405 | <0.001 |
| ARHGEF6 | <0.001 | 0.396 | <0.001 |
| ARID3C | 0.012 | 0.352 | 0.040 |
| ARIH2P1 | 0.013 | 0.296 | 0.042 |
| ARL11 | <0.001 | -0.297 | <0.001 |
| ARL13A | 0.002 | 0.330 | 0.010 |
| ARMC9 | <0.001 | 0.402 | <0.001 |
| ARMH1 | <0.001 | 0.529 | 0.002 |
| ARMH4 | 0.005 | 0.280 | 0.021 |
| ARNTL2-AS1 | 0.001 | -0.570 | 0.006 |
| ARPC3P5 | 0.004 | -0.544 | 0.016 |
| ARPP21 | 0.005 | 0.816 | 0.020 |
| ARSB | <0.001 | 0.345 | <0.001 |
| ARSH | <0.001 | -0.757 | 0.005 |
| ARSI | <0.001 | 0.356 | <0.001 |
| ARSK | <0.001 | 0.294 | <0.001 |
| ART3 | <0.001 | 1.060 | <0.001 |
| ART4 | 0.003 | 0.949 | 0.014 |
| ASB10 | 0.010 | 0.970 | 0.036 |
| ASB14 | 0.003 | 0.286 | 0.012 |
| ASB18 | 0.015 | 0.797 | 0.048 |
| ASB2 | <0.001 | 0.362 | <0.001 |
| ASB4 | 0.009 | 0.848 | 0.034 |
| ASB5 | 0.006 | 0.654 | 0.024 |
| ASGR1 | 0.007 | 0.319 | 0.026 |
| ASPG | <0.001 | -1.124 | <0.001 |
| ASPHD1 | <0.001 | 0.399 | <0.001 |
| ASPRV1 | <0.001 | -1.205 | <0.001 |
| ASTL | <0.001 | -0.845 | <0.001 |
| ASXL3 | <0.001 | 0.861 | 0.003 |
| ATE1-AS1 | <0.001 | 0.745 | <0.001 |
| ATG9B | <0.001 | -1.060 | <0.001 |
| ATOH8 | 0.002 | 0.529 | 0.008 |
| ATP10A | <0.001 | 0.497 | <0.001 |
| ATP10B | <0.001 | -1.095 | <0.001 |
| ATP12A | <0.001 | -0.735 | <0.001 |
| ATP13A4 | <0.001 | -1.494 | <0.001 |
| ATP13A5 | <0.001 | -1.995 | <0.001 |
| ATP1A1-AS1 | <0.001 | 0.297 | <0.001 |
| ATP1A2 | 0.008 | 0.582 | 0.031 |
| ATP1A4 | 0.002 | 0.745 | 0.010 |
| ATP1B4 | 0.003 | 0.799 | 0.014 |
| ATP2C2 | <0.001 | -0.324 | 0.002 |
| ATP5MFP4 | 0.002 | 0.597 | 0.010 |
| ATP5PBP1 | <0.001 | -0.697 | 0.004 |
| ATP5PBP5 | <0.001 | 0.542 | <0.001 |
| ATP6AP1L | <0.001 | 0.605 | <0.001 |
| ATP6V0A4 | <0.001 | -0.583 | 0.002 |
| ATP6V0E2-AS1 | 0.002 | 0.489 | 0.011 |
| ATP6V1C2 | <0.001 | -0.985 | <0.001 |
| ATP6V1G2 | 0.006 | 0.399 | 0.025 |
| ATP8A1 | <0.001 | 0.449 | <0.001 |
| ATP8A2 | <0.001 | 1.108 | <0.001 |
| ATP8B4 | <0.001 | 0.481 | <0.001 |
| AXL | <0.001 | 0.378 | <0.001 |
| AZGP1 | <0.001 | -0.686 | <0.001 |
| AZGP1P1 | <0.001 | -1.195 | 0.002 |
| AZIN1-AS1 | <0.001 | 0.638 | <0.001 |
| AZIN2 | <0.001 | 0.677 | <0.001 |
| B3GAT3P1 | 0.004 | 0.477 | 0.018 |
| B3GNT4 | <0.001 | -0.275 | 0.005 |
| B3GNT6 | 0.002 | -1.039 | 0.009 |
| B3GNT8 | <0.001 | -0.266 | <0.001 |
| B4GALNT3 | <0.001 | -0.422 | <0.001 |
| B4GALT6 | <0.001 | 0.444 | <0.001 |
| BAALC-AS1 | 0.004 | 0.274 | 0.016 |
| BACH2 | <0.001 | 0.612 | <0.001 |
| BAIAP3 | 0.006 | -0.288 | 0.023 |
| BASP1 | <0.001 | 0.318 | <0.001 |
| BASP1-AS1 | <0.001 | 1.160 | <0.001 |
| BATF3 | <0.001 | 0.525 | <0.001 |
| BBOX1 | <0.001 | -0.369 | 0.003 |
| BBS12 | <0.001 | 0.406 | <0.001 |
| BCAP31P1 | <0.001 | -0.843 | <0.001 |
| BCAP31P2 | 0.004 | -0.914 | 0.017 |
| BCAR3 | <0.001 | 0.342 | <0.001 |
| BCAR3-AS1 | <0.001 | 0.710 | 0.005 |
| BCAS1 | <0.001 | -0.731 | <0.001 |
| BCL2L10 | <0.001 | -0.563 | <0.001 |
| BCL2L14 | <0.001 | 0.551 | 0.005 |
| BCO1 | <0.001 | -1.081 | <0.001 |
| BCO2 | <0.001 | -0.820 | <0.001 |
| BCRP8 | <0.001 | -0.889 | <0.001 |
| BDH1 | <0.001 | -0.437 | <0.001 |
| BDNF | <0.001 | -0.587 | 0.006 |
| BEGAIN | 0.001 | 0.564 | 0.007 |
| BEND3P1 | 0.008 | 0.432 | 0.029 |
| BEND3P3 | 0.002 | 0.312 | 0.010 |
| BEND5 | 0.011 | 0.431 | 0.037 |
| BEND6 | <0.001 | 0.385 | <0.001 |
| BEST3 | 0.004 | 0.812 | 0.017 |
| BHLHE40-AS1 | <0.001 | 0.342 | 0.004 |
| BHLHE41 | <0.001 | 0.439 | <0.001 |
| BHMT2 | <0.001 | 0.415 | 0.004 |
| BICC1 | <0.001 | 0.382 | <0.001 |
| BICDL1 | <0.001 | -0.271 | <0.001 |
| BICDL2 | <0.001 | -0.651 | <0.001 |
| BIN1 | <0.001 | 0.298 | <0.001 |
| BIRC7 | 0.003 | 0.681 | 0.015 |
| BLACE | 0.003 | -1.411 | 0.015 |
| BLZF2P | <0.001 | 0.613 | 0.005 |
| BMERB1 | <0.001 | 0.361 | <0.001 |
| BMP5 | 0.007 | 1.121 | 0.028 |
| BMP8A | <0.001 | 0.562 | <0.001 |
| BMPER | <0.001 | 1.264 | <0.001 |
| BNC2 | <0.001 | 0.726 | <0.001 |
| BNC2-AS1 | <0.001 | 1.059 | <0.001 |
| BNIPL | <0.001 | -0.725 | <0.001 |
| BOK-AS1 | <0.001 | -2.039 | <0.001 |
| BPI | <0.001 | 0.827 | 0.003 |
| BPIFB1 | 0.011 | -0.705 | 0.039 |
| BPIFB4 | 0.003 | 1.307 | 0.014 |
| BPIFC | <0.001 | -1.298 | <0.001 |
| BRD9P2 | 0.004 | -0.627 | 0.016 |
| BRSK1 | 0.002 | 0.354 | 0.008 |
| BSN | 0.003 | 0.738 | 0.012 |
| BSPRY | <0.001 | -0.575 | <0.001 |
| BST1 | <0.001 | 0.314 | 0.001 |
| BTBD16 | <0.001 | -1.319 | <0.001 |
| BTBD6P1 | 0.002 | 0.715 | 0.011 |
| BTBD8 | 0.002 | 0.322 | 0.010 |
| BTC | 0.006 | 0.403 | 0.023 |
| BTF3P13 | 0.002 | 0.508 | 0.011 |
| BTF3P7 | <0.001 | -0.750 | <0.001 |
| BTG1P1 | 0.012 | 0.530 | 0.042 |
| BTLA | 0.001 | 0.496 | 0.007 |
| BTN1A1 | 0.005 | 0.773 | 0.020 |
| BTN2A3P | <0.001 | 0.333 | <0.001 |
| BUB1B-PAK6 | 0.003 | -0.479 | 0.012 |
| BVES | <0.001 | 0.424 | <0.001 |
| BVES-AS1 | <0.001 | 0.616 | 0.003 |
| BX119917.1 | <0.001 | 0.652 | <0.001 |
| BX284668.2 | 0.002 | -0.926 | 0.011 |
| BX293535.1 | 0.002 | -0.438 | 0.009 |
| BX322562.1 | <0.001 | 0.363 | <0.001 |
| BX322650.1 | 0.002 | 0.641 | 0.009 |
| BX324167.1 | <0.001 | -1.280 | <0.001 |
| BX324167.2 | <0.001 | -1.102 | <0.001 |
| BX679664.1 | 0.004 | 0.356 | 0.018 |
| C10orf105 | <0.001 | -1.548 | 0.003 |
| C10orf142 | <0.001 | 1.077 | <0.001 |
| C10orf25 | <0.001 | 0.502 | <0.001 |
| C10orf71 | 0.006 | 0.781 | 0.023 |
| C10orf91 | <0.001 | -0.672 | <0.001 |
| C10orf95 | <0.001 | -0.500 | <0.001 |
| C10orf99 | <0.001 | -0.494 | <0.001 |
| C11orf16 | 0.003 | -0.923 | 0.014 |
| C11orf21 | 0.007 | 0.413 | 0.028 |
| C11orf44 | <0.001 | 0.700 | 0.002 |
| C11orf52 | 0.005 | -0.482 | 0.020 |
| C11orf96 | <0.001 | 0.270 | <0.001 |
| C12orf56 | <0.001 | -0.421 | <0.001 |
| C12orf80 | <0.001 | -1.109 | <0.001 |
| C14orf132 | <0.001 | 0.320 | <0.001 |
| C15orf56 | 0.010 | -0.403 | 0.035 |
| C15orf62 | <0.001 | -0.892 | <0.001 |
| C16orf54 | <0.001 | 0.367 | 0.001 |
| C17orf47 | <0.001 | 0.672 | 0.002 |
| C17orf49 | <0.001 | 0.270 | <0.001 |
| C17orf50 | <0.001 | 0.947 | <0.001 |
| C19orf38 | 0.001 | 0.278 | 0.007 |
| C1DP5 | 0.011 | 0.551 | 0.039 |
| C1orf105 | 0.002 | 0.987 | 0.010 |
| C1orf116 | <0.001 | -0.273 | <0.001 |
| C1orf127 | <0.001 | 0.676 | 0.002 |
| C1orf137 | <0.001 | -1.271 | <0.001 |
| C1orf162 | <0.001 | 0.311 | <0.001 |
| C1orf210 | <0.001 | -0.266 | <0.001 |
| C1orf226 | <0.001 | -0.369 | <0.001 |
| C1orf68 | <0.001 | -0.698 | 0.002 |
| C1QL1 | <0.001 | 0.491 | <0.001 |
| C1QL4 | 0.002 | 0.694 | 0.011 |
| C1QTNF12 | 0.004 | -0.376 | 0.017 |
| C1QTNF2 | <0.001 | 0.553 | <0.001 |
| C1QTNF3 | <0.001 | 0.629 | <0.001 |
| C1QTNF6 | <0.001 | 0.289 | <0.001 |
| C1QTNF7 | 0.003 | 0.544 | 0.014 |
| C1QTNF7-AS1 | <0.001 | -0.624 | 0.005 |
| C2-AS1 | 0.002 | -0.898 | 0.009 |
| C21orf62-AS1 | <0.001 | 0.429 | 0.004 |
| C2CD4D | 0.005 | 0.324 | 0.021 |
| C2orf27A | <0.001 | 0.292 | <0.001 |
| C3AR1 | <0.001 | 0.344 | <0.001 |
| C3orf18 | <0.001 | 0.564 | <0.001 |
| C3orf35 | 0.015 | 0.267 | 0.048 |
| C3orf80 | <0.001 | 0.559 | <0.001 |
| C4A | <0.001 | 0.442 | 0.005 |
| C4orf54 | 0.003 | 0.873 | 0.013 |
| C5orf34 | <0.001 | 0.333 | <0.001 |
| C5orf66 | <0.001 | -0.442 | <0.001 |
| C5orf66-AS1 | <0.001 | -0.590 | <0.001 |
| C6orf132 | <0.001 | -0.320 | <0.001 |
| C6orf15 | <0.001 | -0.878 | <0.001 |
| C6orf223 | 0.011 | -0.413 | 0.039 |
| C7 | 0.002 | 0.804 | 0.009 |
| C7orf57 | <0.001 | -0.943 | <0.001 |
| C7orf61 | 0.010 | 0.295 | 0.037 |
| C7orf65 | 0.002 | 1.047 | 0.011 |
| C8orf37-AS1 | 0.013 | 0.407 | 0.044 |
| C8orf48 | 0.001 | 0.506 | 0.006 |
| C8orf74 | <0.001 | -2.351 | <0.001 |
| C8orf88 | <0.001 | 0.629 | <0.001 |
| C9 | 0.002 | 0.597 | 0.009 |
| C9orf131 | 0.006 | -0.892 | 0.022 |
| C9orf139 | 0.004 | 0.422 | 0.018 |
| C9orf170 | <0.001 | 1.238 | <0.001 |
| C9orf47 | <0.001 | 0.741 | <0.001 |
| CA3 | 0.014 | 0.494 | 0.045 |
| CA6 | 0.012 | -0.984 | 0.041 |
| CABP4 | <0.001 | 0.913 | <0.001 |
| CACNA1A | 0.007 | 0.385 | 0.026 |
| CACNA1C | <0.001 | 0.354 | 0.003 |
| CACNA1G | 0.005 | 0.546 | 0.019 |
| CACNA1H | 0.002 | 0.338 | 0.010 |
| CACNA1S | 0.009 | 0.689 | 0.033 |
| CACNA2D2 | 0.015 | 0.334 | 0.049 |
| CACNA2D3 | <0.001 | -0.381 | 0.002 |
| CACNA2D4 | <0.001 | 0.324 | 0.004 |
| CACNB1 | <0.001 | 0.399 | <0.001 |
| CACNB2 | <0.001 | 0.455 | 0.002 |
| CACNG1 | 0.002 | 0.661 | 0.011 |
| CACNG6 | 0.010 | 0.609 | 0.035 |
| CACTIN-AS1 | 0.004 | 0.269 | 0.016 |
| CADM1 | 0.003 | 0.302 | 0.013 |
| CADM3 | <0.001 | 0.473 | 0.004 |
| CADM4 | <0.001 | -0.296 | <0.001 |
| CALB1 | 0.004 | -0.653 | 0.019 |
| CALB2 | <0.001 | -0.409 | <0.001 |
| CALHM1 | <0.001 | -1.850 | 0.001 |
| CALHM5 | <0.001 | 0.466 | <0.001 |
| CALHM6 | <0.001 | 0.367 | <0.001 |
| CALML5 | <0.001 | -0.497 | <0.001 |
| CAMK1 | <0.001 | 0.289 | <0.001 |
| CAMK1G | <0.001 | 0.538 | <0.001 |
| CAMK2A | <0.001 | 0.703 | <0.001 |
| CAMK2B | 0.014 | 0.474 | 0.047 |
| CAMK2N1 | <0.001 | 0.312 | <0.001 |
| CAMK4 | <0.001 | 0.597 | <0.001 |
| CAMSAP3 | <0.001 | -0.371 | <0.001 |
| CAP2 | <0.001 | 0.267 | 0.001 |
| CAPN14 | <0.001 | -0.848 | <0.001 |
| CAPN6 | <0.001 | 1.045 | 0.001 |
| CAPNS1P1 | <0.001 | -1.030 | <0.001 |
| CAPNS2 | <0.001 | -0.377 | <0.001 |
| CARD14 | <0.001 | -0.536 | <0.001 |
| CARD17 | <0.001 | -0.378 | 0.001 |
| CARD18 | <0.001 | -0.891 | <0.001 |
| CARMIL2 | 0.009 | 0.302 | 0.033 |
| CARMIL3 | <0.001 | -0.778 | <0.001 |
| CARMN | <0.001 | 0.609 | <0.001 |
| CARNS1 | <0.001 | 0.450 | 0.004 |
| CASC18 | <0.001 | 1.000 | <0.001 |
| CASC19 | <0.001 | -0.601 | 0.005 |
| CASKIN1 | <0.001 | -0.638 | 0.002 |
| CASP17P | <0.001 | 0.426 | <0.001 |
| CASP1P2 | <0.001 | -0.385 | 0.004 |
| CASQ2 | 0.004 | 0.514 | 0.017 |
| CASS4 | 0.003 | 0.329 | 0.015 |
| CASTOR1 | <0.001 | -0.265 | 0.001 |
| CATIP-AS1 | 0.012 | 0.385 | 0.040 |
| CATSPER1 | 0.010 | 0.346 | 0.036 |
| CAV3 | 0.009 | 0.642 | 0.032 |
| CAVIN3 | <0.001 | 0.292 | <0.001 |
| CAVIN4 | <0.001 | 0.582 | 0.003 |
| CBR3-AS1 | <0.001 | -0.348 | <0.001 |
| CCAT1 | <0.001 | -0.648 | 0.002 |
| CCBE1 | <0.001 | 0.899 | <0.001 |
| CCDC102A | <0.001 | 0.309 | <0.001 |
| CCDC102B | <0.001 | 0.418 | <0.001 |
| CCDC110 | <0.001 | 0.519 | 0.004 |
| CCDC113 | <0.001 | 0.442 | <0.001 |
| CCDC120 | <0.001 | -0.301 | <0.001 |
| CCDC122 | <0.001 | 0.353 | <0.001 |
| CCDC141 | <0.001 | 0.775 | 0.001 |
| CCDC144A | 0.009 | 0.630 | 0.034 |
| CCDC149 | <0.001 | 0.266 | <0.001 |
| CCDC150P1 | 0.009 | -0.323 | 0.031 |
| CCDC158 | <0.001 | 1.009 | <0.001 |
| CCDC177 | 0.013 | -0.617 | 0.045 |
| CCDC180 | 0.010 | 0.518 | 0.036 |
| CCDC187 | 0.001 | -0.889 | 0.007 |
| CCDC188 | <0.001 | 0.456 | <0.001 |
| CCDC190 | <0.001 | -0.887 | <0.001 |
| CCDC200 | <0.001 | 0.409 | <0.001 |
| CCDC28B | <0.001 | 0.303 | <0.001 |
| CCDC36 | <0.001 | 0.502 | 0.006 |
| CCDC40 | 0.010 | 0.272 | 0.036 |
| CCDC42 | <0.001 | 0.826 | 0.002 |
| CCDC54 | <0.001 | 0.673 | 0.003 |
| CCDC68 | 0.003 | 0.420 | 0.015 |
| CCDC7 | <0.001 | 0.365 | <0.001 |
| CCDC8 | 0.001 | 0.303 | 0.006 |
| CCDC88A | <0.001 | 0.292 | <0.001 |
| CCDC88B | <0.001 | -0.316 | <0.001 |
| CCIN | <0.001 | 0.974 | <0.001 |
| CCK | 0.002 | 1.262 | 0.009 |
| CCKAR | 0.002 | 0.834 | 0.009 |
| CCL13 | <0.001 | 0.352 | <0.001 |
| CCL24 | 0.002 | -0.285 | 0.011 |
| CCL26 | 0.007 | -0.369 | 0.028 |
| CCL3L3 | 0.007 | 0.275 | 0.027 |
| CCL4 | <0.001 | 0.304 | <0.001 |
| CCL4L2 | <0.001 | 0.340 | <0.001 |
| CCL7 | 0.004 | 0.470 | 0.018 |
| CCL8 | 0.010 | 0.269 | 0.035 |
| CCNJL | <0.001 | 0.483 | <0.001 |
| CCR1 | <0.001 | 0.332 | <0.001 |
| CCR2 | 0.005 | 0.396 | 0.020 |
| CCR3 | 0.015 | 0.499 | 0.050 |
| CCR5 | <0.001 | 0.357 | 0.001 |
| CCR5AS | 0.002 | 0.335 | 0.008 |
| CCR6 | 0.002 | 0.556 | 0.010 |
| CCR8 | <0.001 | 0.423 | 0.001 |
| CCR9 | <0.001 | 0.826 | <0.001 |
| CCRL2 | <0.001 | 0.328 | <0.001 |
| CCT7P1 | <0.001 | -1.117 | <0.001 |
| CD163 | <0.001 | 0.278 | 0.002 |
| CD164L2 | <0.001 | -0.432 | <0.001 |
| CD177 | <0.001 | -0.939 | <0.001 |
| CD177P1 | <0.001 | -1.526 | <0.001 |
| CD180 | 0.001 | 0.365 | 0.007 |
| CD1A | 0.002 | 0.309 | 0.008 |
| CD1D | <0.001 | 0.590 | <0.001 |
| CD1E | 0.007 | 0.339 | 0.026 |
| CD200R1 | 0.015 | 0.293 | 0.048 |
| CD207 | 0.011 | 0.264 | 0.039 |
| CD209 | 0.006 | 0.270 | 0.025 |
| CD226 | 0.002 | 0.397 | 0.011 |
| CD24P4 | 0.013 | -0.336 | 0.044 |
| CD28 | <0.001 | 0.462 | <0.001 |
| CD300A | <0.001 | 0.347 | <0.001 |
| CD300C | <0.001 | 0.394 | <0.001 |
| CD300LF | <0.001 | 0.442 | <0.001 |
| CD300LG | 0.012 | 0.673 | 0.041 |
| CD33 | 0.001 | 0.372 | 0.006 |
| CD36 | 0.002 | -0.388 | 0.010 |
| CD37 | <0.001 | 0.290 | <0.001 |
| CD3G | 0.002 | 0.340 | 0.009 |
| CD40LG | 0.009 | 0.439 | 0.034 |
| CD48 | 0.001 | 0.274 | 0.007 |
| CD5L | 0.003 | 1.265 | 0.013 |
| CD69 | <0.001 | 0.352 | 0.001 |
| CD70 | <0.001 | 0.541 | <0.001 |
| CD72 | <0.001 | 0.595 | <0.001 |
| CD80 | <0.001 | 0.682 | <0.001 |
| CD84 | <0.001 | 0.373 | 0.003 |
| CD86 | <0.001 | 0.314 | <0.001 |
| CD8A | 0.005 | 0.281 | 0.020 |
| CD8B | 0.004 | 0.370 | 0.016 |
| CD8B2 | 0.004 | 1.135 | 0.016 |
| CD96 | 0.005 | 0.266 | 0.022 |
| CDA | <0.001 | -0.278 | <0.001 |
| CDC20P1 | <0.001 | 0.442 | <0.001 |
| CDC42EP3 | <0.001 | 0.299 | <0.001 |
| CDH11 | <0.001 | 0.328 | <0.001 |
| CDH15 | <0.001 | 0.785 | <0.001 |
| CDH16 | <0.001 | -1.017 | <0.001 |
| CDH2 | <0.001 | 1.019 | <0.001 |
| CDH20 | <0.001 | 0.688 | 0.002 |
| CDH22 | <0.001 | 1.122 | 0.001 |
| CDH23 | <0.001 | 0.560 | 0.003 |
| CDH26 | <0.001 | -0.915 | <0.001 |
| CDK14 | <0.001 | 0.370 | <0.001 |
| CDK15 | 0.002 | 0.444 | 0.010 |
| CDK18 | <0.001 | 0.357 | <0.001 |
| CDK20 | <0.001 | 0.531 | <0.001 |
| CDK5R1 | <0.001 | -0.271 | <0.001 |
| CDK5R2 | <0.001 | 1.587 | <0.001 |
| CDKL3 | <0.001 | 0.361 | <0.001 |
| CDON | 0.001 | 0.353 | 0.007 |
| CDRT1 | 0.013 | -0.324 | 0.044 |
| CDSN | <0.001 | -1.290 | <0.001 |
| CDX1 | <0.001 | 0.522 | 0.004 |
| CDYL2 | <0.001 | 0.314 | <0.001 |
| CEACAM1 | <0.001 | -0.353 | <0.001 |
| CEACAM19 | <0.001 | -0.380 | <0.001 |
| CEACAM4 | 0.014 | 0.296 | 0.046 |
| CEACAM5 | <0.001 | -0.753 | <0.001 |
| CEACAM6 | <0.001 | -0.562 | <0.001 |
| CEACAM7 | <0.001 | -1.165 | <0.001 |
| CEACAMP10 | 0.005 | 0.638 | 0.022 |
| CEBPA | <0.001 | -0.316 | <0.001 |
| CEBPE | <0.001 | -0.836 | 0.004 |
| CELF2 | <0.001 | 0.269 | 0.003 |
| CELF4 | 0.003 | 1.395 | 0.012 |
| CEP112 | <0.001 | 0.385 | <0.001 |
| CEP126 | <0.001 | 0.649 | 0.001 |
| CEP170 | <0.001 | 0.321 | <0.001 |
| CEP170P1 | 0.004 | 0.393 | 0.017 |
| CEP250-AS1 | 0.010 | 0.362 | 0.035 |
| CEP41 | <0.001 | 0.293 | <0.001 |
| CEP57L1 | <0.001 | 0.286 | <0.001 |
| CEP85L | <0.001 | 0.440 | <0.001 |
| CERKL | <0.001 | 0.451 | <0.001 |
| CEROX1 | 0.010 | 0.558 | 0.036 |
| CERS3 | <0.001 | -0.503 | <0.001 |
| CERS3-AS1 | <0.001 | -0.567 | <0.001 |
| CES1P2 | <0.001 | -1.208 | 0.001 |
| CES3 | <0.001 | -0.865 | <0.001 |
| CETN4P | <0.001 | 0.546 | <0.001 |
| CETP | <0.001 | 0.329 | 0.003 |
| CFAP300 | <0.001 | 0.616 | 0.002 |
| CFAP46 | 0.008 | -0.881 | 0.030 |
| CFAP53 | <0.001 | -0.399 | 0.002 |
| CFAP54 | 0.004 | 0.382 | 0.019 |
| CFAP58 | 0.003 | 0.499 | 0.015 |
| CFAP58-DT | 0.002 | -0.346 | 0.008 |
| CFAP61 | 0.015 | 0.468 | 0.049 |
| CGB5 | 0.007 | 1.045 | 0.027 |
| CGB7 | <0.001 | 0.545 | <0.001 |
| CGB8 | <0.001 | 1.230 | <0.001 |
| CGN | <0.001 | -0.493 | <0.001 |
| CHAC1 | <0.001 | -0.375 | <0.001 |
| CHAD | 0.009 | 0.506 | 0.032 |
| CHCHD2P4 | <0.001 | -1.191 | <0.001 |
| CHGB | <0.001 | 0.681 | 0.003 |
| CHN2 | <0.001 | 0.429 | <0.001 |
| CHODL | <0.001 | -0.468 | <0.001 |
| CHP2 | <0.001 | -0.593 | 0.002 |
| chr22-38_28785274-29006793.1 | <0.001 | -0.583 | <0.001 |
| CHRD | <0.001 | 0.424 | <0.001 |
| CHRDL1 | 0.010 | 0.479 | 0.036 |
| CHRFAM7A | <0.001 | 0.691 | 0.005 |
| CHRM4 | <0.001 | 0.713 | 0.002 |
| CHRM5 | 0.002 | -0.949 | 0.011 |
| CHRNA1 | 0.001 | 0.581 | 0.007 |
| CHRNA10 | 0.006 | 0.289 | 0.023 |
| CHRNA6 | <0.001 | 1.059 | 0.002 |
| CHRNA7 | 0.010 | 0.556 | 0.035 |
| CHRND | 0.004 | 0.688 | 0.018 |
| CHRNG | 0.002 | 0.714 | 0.011 |
| CHST11 | <0.001 | 0.264 | <0.001 |
| CHST13 | <0.001 | 0.615 | <0.001 |
| CHSY3 | <0.001 | 0.411 | <0.001 |
| CHURC1-FNTB | <0.001 | 0.499 | 0.003 |
| CIDEA | <0.001 | -1.052 | <0.001 |
| CIITA | 0.002 | 0.298 | 0.011 |
| CKLF-CMTM1 | <0.001 | 0.310 | 0.001 |
| CKMT1A | <0.001 | -0.288 | <0.001 |
| CKMT1B | <0.001 | -0.283 | <0.001 |
| CKS1BP1 | <0.001 | -0.290 | <0.001 |
| CKS1BP7 | 0.005 | 0.363 | 0.021 |
| CLCA3P | 0.001 | -0.669 | 0.006 |
| CLCA4 | <0.001 | -0.654 | <0.001 |
| CLCA4-AS1 | 0.001 | -0.700 | 0.006 |
| CLCN4 | <0.001 | 0.587 | <0.001 |
| CLDN10 | 0.004 | 0.695 | 0.018 |
| CLDN11 | 0.001 | 0.495 | 0.007 |
| CLDN17 | <0.001 | -1.838 | <0.001 |
| CLDN19 | 0.006 | -0.965 | 0.024 |
| CLDN34 | 0.007 | -0.431 | 0.025 |
| CLDN4 | <0.001 | -0.315 | <0.001 |
| CLDN8 | 0.003 | -0.660 | 0.014 |
| CLEC17A | 0.004 | 0.644 | 0.018 |
| CLEC1A | 0.006 | 0.344 | 0.024 |
| CLEC2D | <0.001 | 0.375 | <0.001 |
| CLEC4A | <0.001 | 0.360 | <0.001 |
| CLEC4F | <0.001 | 0.912 | 0.003 |
| CLEC9A | 0.009 | 0.391 | 0.033 |
| CLECL1 | <0.001 | 0.543 | <0.001 |
| CLIC3 | <0.001 | -0.472 | <0.001 |
| CLIC5 | <0.001 | 0.621 | 0.001 |
| CLIP3 | <0.001 | 0.332 | <0.001 |
| CLMP | <0.001 | 0.347 | <0.001 |
| CLNK | 0.010 | 0.365 | 0.036 |
| CLSTN2 | <0.001 | 0.547 | 0.002 |
| CLUL1 | 0.002 | 1.018 | 0.012 |
| CMKLR1 | <0.001 | 0.416 | <0.001 |
| CMTM1 | <0.001 | 0.270 | 0.003 |
| CMTM5 | <0.001 | -1.069 | 0.004 |
| CMTM7 | <0.001 | 0.321 | <0.001 |
| CMYA5 | 0.009 | 0.477 | 0.033 |
| CNFN | <0.001 | -0.532 | <0.001 |
| CNGA1 | 0.004 | -0.289 | 0.019 |
| CNIH3 | <0.001 | 0.502 | <0.001 |
| CNIH3-AS2 | <0.001 | 0.827 | <0.001 |
| CNKSR3 | <0.001 | -0.362 | <0.001 |
| CNN1 | 0.001 | 0.284 | 0.007 |
| CNRIP1 | <0.001 | 0.305 | <0.001 |
| CNTN1 | <0.001 | -0.382 | <0.001 |
| CNTN2 | 0.005 | 1.940 | 0.021 |
| CNTN4 | <0.001 | 0.641 | 0.004 |
| CNTNAP3 | <0.001 | 0.521 | <0.001 |
| CNTNAP3P2 | 0.009 | 0.335 | 0.032 |
| COL10A1 | <0.001 | 0.404 | <0.001 |
| COL11A1 | <0.001 | 0.415 | 0.004 |
| COL11A2 | 0.002 | 0.543 | 0.009 |
| COL13A1 | <0.001 | 0.309 | <0.001 |
| COL19A1 | <0.001 | 2.013 | <0.001 |
| COL22A1 | <0.001 | 0.674 | <0.001 |
| COL23A1 | <0.001 | 0.514 | <0.001 |
| COL24A1 | <0.001 | 0.455 | 0.002 |
| COL25A1 | <0.001 | 0.967 | 0.005 |
| COL26A1 | 0.007 | 0.690 | 0.027 |
| COL4A2-AS1 | 0.004 | 0.549 | 0.018 |
| COL4A3 | <0.001 | 0.821 | <0.001 |
| COL4A4 | <0.001 | 0.770 | 0.001 |
| COL5A1 | <0.001 | 0.275 | <0.001 |
| COL5A1-AS1 | <0.001 | 1.035 | <0.001 |
| COL8A1 | <0.001 | 0.314 | 0.002 |
| COLEC11 | 0.008 | -0.475 | 0.030 |
| COLEC12 | <0.001 | 0.371 | <0.001 |
| COLQ | <0.001 | 0.684 | <0.001 |
| COPS8P2 | 0.004 | 0.514 | 0.017 |
| CORIN | 0.008 | 0.438 | 0.031 |
| CORO2B | <0.001 | 0.819 | <0.001 |
| CORO6 | 0.006 | 0.292 | 0.025 |
| COX6A2 | 0.012 | 0.498 | 0.042 |
| CPAMD8 | 0.009 | 0.371 | 0.033 |
| CPEB1-AS1 | 0.004 | 0.981 | 0.016 |
| CPEB2-DT | <0.001 | -0.678 | 0.003 |
| CPEB3 | <0.001 | -0.294 | <0.001 |
| CPNE9 | 0.003 | 0.659 | 0.015 |
| CPQ | <0.001 | 0.449 | <0.001 |
| CPXM1 | <0.001 | 0.284 | <0.001 |
| CR1L | 0.001 | 1.011 | 0.007 |
| CRB3 | <0.001 | -0.273 | <0.001 |
| CRCT1 | <0.001 | -0.692 | <0.001 |
| CREB3L1 | <0.001 | 0.384 | <0.001 |
| CREB5 | 0.008 | 0.264 | 0.030 |
| CRISP3 | <0.001 | -0.910 | 0.004 |
| CRNN | <0.001 | -1.153 | <0.001 |
| CRTAC1 | <0.001 | 0.681 | 0.001 |
| CRTAM | 0.009 | 0.387 | 0.031 |
| CRYBB1 | <0.001 | 0.420 | 0.001 |
| CRYBG2 | <0.001 | -0.372 | <0.001 |
| CRYGN | <0.001 | 0.670 | 0.005 |
| CRYM | <0.001 | -0.900 | <0.001 |
| CRYM-AS1 | <0.001 | -0.295 | 0.005 |
| CSDC2 | 0.005 | 0.509 | 0.020 |
| CSF1 | <0.001 | 0.331 | <0.001 |
| CSF1R | <0.001 | 0.294 | <0.001 |
| CSF2 | <0.001 | 0.457 | 0.004 |
| CSF2RBP1 | 0.003 | -0.854 | 0.013 |
| CSMD2 | <0.001 | 0.583 | 0.001 |
| CSMD3 | 0.014 | 0.876 | 0.045 |
| CSPG4 | <0.001 | 0.269 | <0.001 |
| CSPG4P13 | <0.001 | 0.722 | 0.002 |
| CST2 | <0.001 | 0.729 | 0.002 |
| CSTA | <0.001 | -0.373 | <0.001 |
| CT69 | <0.001 | -0.483 | <0.001 |
| CT75 | 0.002 | -0.525 | 0.009 |
| CTB-119C2.1 | <0.001 | 0.417 | 0.004 |
| CTC-205M6.5 | <0.001 | 0.366 | <0.001 |
| CTC-251I16.1 | <0.001 | 0.407 | <0.001 |
| CTC-786C10.1 | <0.001 | 0.405 | <0.001 |
| CTD-2331H12.5 | 0.002 | -0.436 | 0.009 |
| CTD-2554C21.3 | <0.001 | -0.783 | 0.003 |
| CTLA4 | <0.001 | 0.364 | <0.001 |
| CTNNA3 | 0.013 | 0.866 | 0.043 |
| CTRC | <0.001 | 0.765 | 0.001 |
| CTSE | 0.006 | 1.028 | 0.024 |
| CU633967.1 | 0.004 | 0.568 | 0.017 |
| CU634019.6 | <0.001 | 0.865 | <0.001 |
| CUBN | <0.001 | 0.499 | <0.001 |
| CUBNP2 | <0.001 | -1.138 | <0.001 |
| CWH43 | <0.001 | -0.516 | 0.002 |
| CX3CR1 | <0.001 | 0.583 | <0.001 |
| CXCL11 | <0.001 | 0.421 | 0.001 |
| CXCL17 | <0.001 | -0.577 | <0.001 |
| CXCL3 | <0.001 | 0.457 | <0.001 |
| CXCR2 | <0.001 | -0.801 | <0.001 |
| CXCR2P1 | <0.001 | 0.783 | <0.001 |
| CXCR3 | <0.001 | 0.376 | 0.004 |
| CXCR5 | 0.005 | 0.945 | 0.019 |
| CXorf21 | <0.001 | 0.412 | <0.001 |
| CXorf65 | 0.006 | 0.448 | 0.023 |
| CYB5A | <0.001 | -0.273 | <0.001 |
| CYB5R2 | <0.001 | -0.339 | <0.001 |
| CYCSP10 | 0.011 | 0.339 | 0.037 |
| CYCSP6 | <0.001 | -0.433 | 0.005 |
| CYFIP2 | <0.001 | 0.314 | 0.004 |
| CYP1A1 | 0.002 | -1.283 | 0.011 |
| CYP1B1 | <0.001 | 0.437 | <0.001 |
| CYP1B1-AS1 | <0.001 | 0.759 | 0.005 |
| CYP26A1 | <0.001 | -0.934 | <0.001 |
| CYP27A1 | <0.001 | 0.325 | 0.001 |
| CYP2AB1P | <0.001 | -2.060 | <0.001 |
| CYP2C18 | <0.001 | -1.266 | <0.001 |
| CYP2C19 | <0.001 | -1.477 | <0.001 |
| CYP2C9 | <0.001 | -1.666 | <0.001 |
| CYP2E1 | 0.005 | -0.574 | 0.019 |
| CYP2U1 | <0.001 | 0.308 | <0.001 |
| CYP2W1 | <0.001 | -0.896 | <0.001 |
| CYP3A5 | <0.001 | -0.679 | <0.001 |
| CYP46A1 | <0.001 | 0.635 | <0.001 |
| CYP4B1 | 0.006 | -0.500 | 0.023 |
| CYP4F11 | <0.001 | -0.471 | <0.001 |
| CYP4F12 | 0.013 | -0.449 | 0.043 |
| CYP4F2 | <0.001 | -1.734 | <0.001 |
| CYP4F22 | <0.001 | -1.384 | <0.001 |
| CYP4F3 | <0.001 | -0.625 | 0.002 |
| CYP4V2 | <0.001 | 0.339 | <0.001 |
| CYP8B1 | <0.001 | -0.712 | 0.005 |
| CYS1 | <0.001 | 0.422 | <0.001 |
| CYSLTR1 | <0.001 | 0.426 | 0.002 |
| CYSLTR2 | 0.002 | 0.402 | 0.008 |
| CYSRT1 | <0.001 | -0.781 | <0.001 |
| CYTH4 | <0.001 | 0.327 | <0.001 |
| CYTOR | <0.001 | 0.364 | <0.001 |
| DAAM2 | <0.001 | 0.308 | 0.003 |
| DAAM2-AS1 | 0.001 | 0.336 | 0.006 |
| DAB2 | <0.001 | 0.393 | <0.001 |
| DACT1 | <0.001 | 0.615 | <0.001 |
| DACT3 | <0.001 | 0.449 | <0.001 |
| DAPL1 | <0.001 | -0.489 | <0.001 |
| DAZL | <0.001 | 1.078 | 0.002 |
| DBH | 0.010 | 0.486 | 0.035 |
| DBNDD1 | <0.001 | -0.302 | <0.001 |
| DCBLD2 | <0.001 | 0.282 | <0.001 |
| DCHS1 | <0.001 | 0.342 | <0.001 |
| DCHS2 | <0.001 | 1.038 | 0.004 |
| DCLK1 | <0.001 | 0.614 | <0.001 |
| DCLK2 | <0.001 | 0.364 | 0.002 |
| DCLK3 | <0.001 | 0.546 | <0.001 |
| DCP1B | <0.001 | 0.303 | <0.001 |
| DCTN1-AS1 | <0.001 | -0.478 | 0.002 |
| DCUN1D3 | <0.001 | -0.282 | <0.001 |
| DDIT4L | 0.003 | 0.539 | 0.015 |
| DDX55P1 | 0.001 | 0.654 | 0.007 |
| DEFB1 | <0.001 | -0.395 | <0.001 |
| DEFB131E | 0.002 | 0.779 | 0.008 |
| DEFB4A | <0.001 | -0.989 | <0.001 |
| DEGS2 | <0.001 | -0.815 | <0.001 |
| DENND2A | <0.001 | 0.488 | <0.001 |
| DENND2C | <0.001 | -0.436 | <0.001 |
| DENND3 | <0.001 | 0.318 | <0.001 |
| DENND5B | <0.001 | 0.419 | <0.001 |
| DEPP1 | <0.001 | 0.309 | <0.001 |
| DES | 0.014 | 0.374 | 0.045 |
| DET1 | <0.001 | 0.355 | <0.001 |
| DGCR5 | <0.001 | 0.736 | <0.001 |
| DGKZP1 | <0.001 | 0.307 | <0.001 |
| DHH | 0.004 | 0.443 | 0.017 |
| DHRS4L1 | 0.004 | -0.386 | 0.018 |
| DHRS9 | <0.001 | -0.740 | <0.001 |
| DIO1 | 0.008 | -0.658 | 0.030 |
| DIO3 | <0.001 | 0.710 | <0.001 |
| DIO3OS | 0.005 | 0.516 | 0.020 |
| DIRAS1 | <0.001 | 0.589 | <0.001 |
| DIRAS3 | 0.015 | 0.342 | 0.048 |
| DISC1FP1 | 0.001 | -1.166 | 0.007 |
| DISP1 | <0.001 | 0.435 | <0.001 |
| DISP2 | <0.001 | 0.717 | <0.001 |
| DISP3 | 0.002 | 0.772 | 0.010 |
| DKK1 | 0.005 | 0.281 | 0.020 |
| DKK2 | 0.009 | 0.325 | 0.033 |
| DKK4 | <0.001 | -1.167 | <0.001 |
| DKKL1 | <0.001 | 0.608 | 0.002 |
| DLC1 | <0.001 | 0.341 | <0.001 |
| DLEU2L | <0.001 | 0.555 | <0.001 |
| DLEU7-AS1 | <0.001 | 0.578 | <0.001 |
| DLG4 | <0.001 | 0.324 | <0.001 |
| DLK1 | 0.010 | 1.614 | 0.036 |
| DLX3 | <0.001 | -0.343 | <0.001 |
| DLX5 | <0.001 | -0.266 | <0.001 |
| DLX6-AS1 | 0.003 | -0.475 | 0.014 |
| DMBX1 | <0.001 | 0.930 | 0.002 |
| DMD | <0.001 | 0.529 | <0.001 |
| DMGDH | 0.008 | 0.280 | 0.029 |
| DMKN | <0.001 | -0.457 | <0.001 |
| DNAAF4 | <0.001 | 0.575 | <0.001 |
| DNAH1 | <0.001 | 0.302 | 0.005 |
| DNAH10OS | 0.002 | 0.405 | 0.009 |
| DNAH14 | 0.005 | 0.423 | 0.021 |
| DNAH3 | <0.001 | -0.579 | <0.001 |
| DNAH5 | <0.001 | 0.644 | 0.001 |
| DNAJB13 | 0.002 | -0.853 | 0.010 |
| DNAJB3 | <0.001 | -1.187 | <0.001 |
| DNAJC12 | <0.001 | 0.507 | <0.001 |
| DNAJC18 | <0.001 | 0.386 | <0.001 |
| DNAJC19P5 | <0.001 | -0.559 | <0.001 |
| DNAJC5B | 0.004 | 0.585 | 0.016 |
| DNAL1 | <0.001 | 0.263 | <0.001 |
| DNASE1L2 | <0.001 | -0.705 | <0.001 |
| DNASE1L3 | 0.001 | -0.338 | 0.006 |
| DND1P1 | <0.001 | -0.367 | 0.003 |
| DNM1P35 | <0.001 | 0.613 | <0.001 |
| DNM1P46 | <0.001 | 0.717 | 0.001 |
| DNM1P47 | 0.012 | 0.632 | 0.041 |
| DNM3 | <0.001 | 0.532 | <0.001 |
| DNM3OS | <0.001 | 0.527 | 0.005 |
| DNMBP-AS1 | <0.001 | -0.561 | 0.004 |
| DOCK10 | <0.001 | 0.361 | <0.001 |
| DOCK2 | <0.001 | 0.393 | <0.001 |
| DOCK3 | 0.008 | -0.440 | 0.031 |
| DOCK4 | <0.001 | 0.279 | <0.001 |
| DOCK9-DT | <0.001 | -0.398 | <0.001 |
| DOK1 | <0.001 | 0.314 | <0.001 |
| DOK5 | <0.001 | 0.693 | <0.001 |
| DOK6 | <0.001 | 0.740 | <0.001 |
| DOK7 | 0.002 | 0.454 | 0.011 |
| DPEP2NB | <0.001 | -1.855 | <0.001 |
| DPF1 | 0.005 | -0.290 | 0.020 |
| DPF3 | <0.001 | 0.658 | <0.001 |
| DPH6-DT | <0.001 | 0.893 | <0.001 |
| DPP4 | 0.009 | -0.270 | 0.033 |
| DPP4-DT | 0.005 | -0.981 | 0.020 |
| DPRXP4 | <0.001 | -0.404 | <0.001 |
| DPY19L1P2 | <0.001 | 0.428 | <0.001 |
| DPY19L2P2 | 0.011 | 0.410 | 0.039 |
| DRAXIN | <0.001 | 0.807 | <0.001 |
| DRD1 | 0.003 | 1.277 | 0.014 |
| DRD2 | 0.003 | 0.928 | 0.014 |
| DRP2 | <0.001 | 0.901 | <0.001 |
| DSC1 | <0.001 | -1.138 | <0.001 |
| DSC2 | <0.001 | -0.272 | <0.001 |
| DSCAM | 0.004 | 0.652 | 0.018 |
| DSEL | <0.001 | 0.432 | 0.002 |
| DSG1 | <0.001 | -0.671 | <0.001 |
| DSG1-AS1 | <0.001 | -0.827 | <0.001 |
| DSG4 | 0.005 | -1.049 | 0.019 |
| DTNA | 0.001 | 0.495 | 0.008 |
| DUBR | <0.001 | 0.349 | <0.001 |
| DUOX2 | <0.001 | -0.583 | <0.001 |
| DUOXA1 | <0.001 | -0.290 | <0.001 |
| DUOXA2 | <0.001 | -0.872 | <0.001 |
| DUSP15 | 0.004 | 0.710 | 0.018 |
| DUSP26 | 0.003 | 0.598 | 0.014 |
| DUSP27 | 0.005 | 0.690 | 0.022 |
| DUSP4 | <0.001 | 0.505 | <0.001 |
| DUTP1 | 0.001 | -0.525 | 0.007 |
| DYNAP | <0.001 | -1.598 | <0.001 |
| DYNLL1P2 | 0.007 | -0.400 | 0.028 |
| DYRK3 | <0.001 | 0.327 | <0.001 |
| DYSF | <0.001 | 0.316 | <0.001 |
| EBF1 | <0.001 | 0.404 | <0.001 |
| EBF2 | <0.001 | 0.534 | 0.002 |
| EBF3 | 0.001 | 0.384 | 0.006 |
| ECEL1 | 0.013 | 0.530 | 0.043 |
| ECM2 | <0.001 | 0.299 | 0.002 |
| ECRG4 | <0.001 | 1.172 | <0.001 |
| EDA2R | <0.001 | 0.758 | <0.001 |
| EDNRA | <0.001 | 0.310 | <0.001 |
| EEF1A1P16 | 0.004 | 0.298 | 0.017 |
| EEF1A1P17 | 0.015 | 0.347 | 0.049 |
| EEF1A1P22 | 0.002 | 0.292 | 0.008 |
| EEF1A1P25 | 0.004 | 0.284 | 0.017 |
| EEF1A1P29 | 0.003 | 0.326 | 0.014 |
| EEF1DP3 | 0.009 | -0.411 | 0.031 |
| EFCAB10 | <0.001 | -0.601 | 0.004 |
| EFCAB13 | 0.002 | 0.378 | 0.008 |
| EFCAB2 | <0.001 | 0.371 | <0.001 |
| EFCAB5 | 0.007 | 0.341 | 0.026 |
| EFEMP2 | <0.001 | 0.282 | <0.001 |
| EFHD1 | <0.001 | 0.426 | <0.001 |
| EFNB3 | 0.004 | 0.318 | 0.015 |
| EFR3B | <0.001 | -0.597 | <0.001 |
| EGFEM1P | 0.007 | 1.070 | 0.028 |
| EGR4 | <0.001 | -0.723 | 0.003 |
| EHD4-AS1 | <0.001 | -0.440 | 0.005 |
| EHF | <0.001 | -0.296 | <0.001 |
| EID2B | <0.001 | 0.438 | <0.001 |
| EIF2S2P3 | 0.006 | 0.290 | 0.024 |
| EIF4A2P2 | 0.012 | 0.297 | 0.041 |
| ELAVL4 | <0.001 | 0.718 | <0.001 |
| ELF3 | <0.001 | -0.337 | <0.001 |
| ELF3-AS1 | <0.001 | -0.462 | <0.001 |
| ELF5 | <0.001 | -1.714 | <0.001 |
| ELFN2 | <0.001 | 1.052 | <0.001 |
| ELMO1 | <0.001 | 0.277 | <0.001 |
| ELOVL2 | 0.001 | 0.528 | 0.007 |
| ELOVL4 | <0.001 | -0.395 | <0.001 |
| ELOVL7 | <0.001 | -0.414 | <0.001 |
| EMB | <0.001 | 0.284 | <0.001 |
| EMBP1 | <0.001 | 0.528 | 0.003 |
| EMID1 | <0.001 | 0.573 | <0.001 |
| EML4-AS1 | <0.001 | 0.788 | <0.001 |
| EML5 | 0.002 | -0.624 | 0.009 |
| EML6 | <0.001 | -0.416 | 0.005 |
| EMX2 | <0.001 | 0.683 | <0.001 |
| EMX2OS | <0.001 | 0.678 | 0.001 |
| ENC1 | <0.001 | 0.418 | <0.001 |
| ENDOU | <0.001 | -1.305 | <0.001 |
| ENO3 | 0.002 | 0.412 | 0.010 |
| ENO4 | 0.008 | 0.445 | 0.030 |
| ENOX1 | <0.001 | 0.840 | <0.001 |
| ENPEP | <0.001 | 0.331 | <0.001 |
| ENPP1 | <0.001 | 0.539 | <0.001 |
| ENPP2 | <0.001 | 0.375 | <0.001 |
| ENPP4 | <0.001 | 0.416 | 0.003 |
| ENPP6 | 0.009 | 1.012 | 0.031 |
| ENPP7 | 0.010 | -0.865 | 0.035 |
| EP300-AS1 | <0.001 | -0.472 | <0.001 |
| EPB41L1 | <0.001 | -0.318 | <0.001 |
| EPB41L4B | <0.001 | -0.376 | <0.001 |
| EPDR1 | <0.001 | 0.368 | <0.001 |
| EPGN | <0.001 | -0.637 | <0.001 |
| EPHA1-AS1 | <0.001 | -0.539 | 0.003 |
| EPHA3 | <0.001 | 0.656 | <0.001 |
| EPHB2 | <0.001 | 0.338 | <0.001 |
| EPHB6 | <0.001 | -0.415 | <0.001 |
| EPHX3 | <0.001 | -0.671 | <0.001 |
| EPHX4 | 0.005 | 0.408 | 0.021 |
| EPN3 | <0.001 | -0.312 | <0.001 |
| EPO | <0.001 | -0.533 | 0.004 |
| EPOR | <0.001 | 0.332 | <0.001 |
| EPPK1 | <0.001 | -0.316 | <0.001 |
| EPS15-AS1 | 0.001 | -0.458 | 0.006 |
| EPS8L1 | <0.001 | -0.447 | <0.001 |
| EQTN | <0.001 | 0.615 | 0.001 |
| ERAP2 | 0.002 | 0.279 | 0.011 |
| ERBB3 | <0.001 | -0.280 | <0.001 |
| ERFE | 0.005 | 0.306 | 0.019 |
| ERFL | <0.001 | 0.518 | <0.001 |
| ERICD | <0.001 | 0.446 | <0.001 |
| ERICH2 | <0.001 | 0.736 | <0.001 |
| ERMN | <0.001 | 0.548 | 0.004 |
| ERO1B | <0.001 | 0.286 | 0.004 |
| ERVE-1 | 0.002 | -0.401 | 0.011 |
| ERVK9-11 | <0.001 | 0.456 | 0.005 |
| ESM1 | <0.001 | 0.284 | <0.001 |
| ESPNL | 0.003 | -0.577 | 0.014 |
| ESPNP | 0.002 | 1.055 | 0.008 |
| ESR1 | <0.001 | 0.668 | 0.002 |
| ESRG | 0.001 | -1.059 | 0.007 |
| ESRP2 | <0.001 | -0.321 | <0.001 |
| ESRRB | 0.002 | 1.138 | 0.008 |
| ESYT3 | <0.001 | -0.794 | <0.001 |
| ETV7-AS1 | 0.002 | 0.432 | 0.010 |
| EVA1A | <0.001 | 0.676 | <0.001 |
| EVI2A | <0.001 | 0.304 | <0.001 |
| EVPL | <0.001 | -0.336 | <0.001 |
| EVPLL | <0.001 | -1.310 | <0.001 |
| EXPH5 | <0.001 | -0.503 | <0.001 |
| EXTL1 | 0.009 | 0.778 | 0.031 |
| EYA1 | <0.001 | 1.007 | <0.001 |
| F13A1 | 0.001 | 0.275 | 0.006 |
| F2 | 0.007 | -1.211 | 0.027 |
| F5 | <0.001 | 0.523 | <0.001 |
| F7 | 0.004 | 0.941 | 0.017 |
| F8 | <0.001 | 0.320 | <0.001 |
| FA2H | <0.001 | -0.544 | <0.001 |
| FABP6-AS1 | 0.010 | -0.559 | 0.035 |
| FADS3 | <0.001 | 0.375 | <0.001 |
| FAHD2CP | <0.001 | 0.434 | <0.001 |
| FALEC | <0.001 | -1.096 | <0.001 |
| FAM110B | <0.001 | 0.459 | <0.001 |
| FAM131B | <0.001 | 0.515 | 0.003 |
| FAM135A | <0.001 | -0.394 | <0.001 |
| FAM13C | 0.004 | 0.383 | 0.017 |
| FAM151A | 0.009 | -0.332 | 0.033 |
| FAM156A | <0.001 | 0.374 | 0.004 |
| FAM157B | <0.001 | -0.678 | <0.001 |
| FAM157C | <0.001 | -0.480 | 0.004 |
| FAM160A1 | <0.001 | -0.288 | <0.001 |
| FAM160A1-DT | <0.001 | -0.286 | 0.001 |
| FAM161A | <0.001 | 0.274 | 0.004 |
| FAM166C | 0.010 | 0.571 | 0.035 |
| FAM171B | <0.001 | 0.387 | <0.001 |
| FAM180B | 0.002 | 0.790 | 0.011 |
| FAM184A | 0.008 | 0.540 | 0.030 |
| FAM184B | 0.003 | -0.641 | 0.015 |
| FAM189A1 | 0.003 | 0.625 | 0.014 |
| FAM198B-AS1 | <0.001 | 0.599 | <0.001 |
| FAM205C | 0.012 | 0.669 | 0.041 |
| FAM222A | <0.001 | -0.355 | 0.001 |
| FAM225A | <0.001 | 0.840 | <0.001 |
| FAM225B | <0.001 | 1.018 | <0.001 |
| FAM227A | <0.001 | 0.621 | <0.001 |
| FAM228B | <0.001 | 0.328 | <0.001 |
| FAM229B | <0.001 | 0.421 | <0.001 |
| FAM25A | <0.001 | -1.029 | <0.001 |
| FAM25BP | <0.001 | -1.360 | <0.001 |
| FAM25C | <0.001 | -1.146 | <0.001 |
| FAM25G | <0.001 | -1.512 | <0.001 |
| FAM27E3 | 0.008 | 0.453 | 0.029 |
| FAM3B | 0.006 | -0.500 | 0.022 |
| FAM3D | <0.001 | -1.028 | <0.001 |
| FAM53A | 0.012 | 0.273 | 0.042 |
| FAM66A | 0.005 | 0.658 | 0.021 |
| FAM66C | 0.010 | 0.402 | 0.036 |
| FAM71F1 | 0.013 | -0.895 | 0.043 |
| FAM78A | <0.001 | 0.332 | <0.001 |
| FAM78B | <0.001 | 0.624 | <0.001 |
| FAM78B-AS1 | 0.008 | 0.462 | 0.030 |
| FAM83C | <0.001 | -0.686 | <0.001 |
| FAM83C-AS1 | <0.001 | -0.835 | <0.001 |
| FAM83E | <0.001 | -0.429 | <0.001 |
| FAM86B1 | 0.002 | 0.458 | 0.008 |
| FAM87A | <0.001 | 0.517 | 0.002 |
| FAM96AP2 | <0.001 | -0.623 | <0.001 |
| FAP | <0.001 | 0.443 | <0.001 |
| FAR2 | <0.001 | 0.286 | 0.002 |
| FAS | <0.001 | 0.297 | <0.001 |
| FASLG | 0.002 | 0.465 | 0.008 |
| FAT3 | <0.001 | 1.288 | 0.003 |
| FAXC | 0.008 | -0.411 | 0.028 |
| FBLN7 | <0.001 | 0.758 | <0.001 |
| FBN1 | <0.001 | 0.290 | <0.001 |
| FBXL2 | <0.001 | 0.286 | <0.001 |
| FBXL22 | <0.001 | -0.439 | <0.001 |
| FBXL7 | <0.001 | 0.424 | <0.001 |
| FBXO17 | <0.001 | 0.306 | <0.001 |
| FBXO32 | <0.001 | 0.293 | <0.001 |
| FBXO43 | <0.001 | 0.632 | <0.001 |
| FCER2 | 0.015 | 0.849 | 0.048 |
| FCGR1A | <0.001 | 0.448 | <0.001 |
| FCGR1B | 0.001 | 0.503 | 0.006 |
| FCGR1CP | 0.001 | 0.521 | 0.006 |
| FCGR2C | 0.004 | 0.311 | 0.017 |
| FCGR3A | <0.001 | 0.273 | <0.001 |
| FCGR3B | 0.010 | -0.446 | 0.035 |
| FCMR | <0.001 | 0.364 | <0.001 |
| FCRL3 | 0.002 | 0.562 | 0.010 |
| FERMT2 | <0.001 | 0.333 | <0.001 |
| FETUB | <0.001 | -1.318 | <0.001 |
| FEZF1 | 0.015 | -0.673 | 0.049 |
| FEZF1-AS1 | 0.003 | -0.640 | 0.012 |
| FFAR1 | 0.007 | -1.919 | 0.028 |
| FFAR2 | <0.001 | -0.841 | <0.001 |
| FGD5 | <0.001 | 0.325 | <0.001 |
| FGF1 | 0.002 | 0.330 | 0.012 |
| FGF11 | 0.006 | -0.276 | 0.024 |
| FGF18 | <0.001 | 0.590 | 0.002 |
| FGF2 | 0.006 | 0.368 | 0.023 |
| FGF22 | <0.001 | -0.615 | 0.002 |
| FGF5 | 0.002 | 1.297 | 0.009 |
| FGFBP2 | <0.001 | -0.873 | 0.003 |
| FGFBP3 | <0.001 | -0.440 | 0.001 |
| FGFR1 | <0.001 | 0.290 | <0.001 |
| FGL2 | <0.001 | 0.267 | 0.002 |
| FHIT | <0.001 | 0.416 | 0.002 |
| FHOD3 | <0.001 | 0.619 | <0.001 |
| FIBIN | 0.002 | 0.303 | 0.011 |
| FIGN | <0.001 | 0.748 | <0.001 |
| FKBP7 | <0.001 | 0.359 | <0.001 |
| FKBP9P1 | <0.001 | 0.368 | 0.004 |
| FKSG51 | <0.001 | -0.548 | <0.001 |
| FKSG52 | 0.002 | -0.367 | 0.011 |
| FKSG54 | <0.001 | -0.577 | 0.004 |
| FKSG56 | 0.003 | -0.519 | 0.014 |
| FKSG61 | 0.001 | -0.496 | 0.007 |
| FKSG62 | <0.001 | -0.556 | <0.001 |
| FKSG66 | 0.001 | -0.786 | 0.007 |
| FKSG68 | 0.001 | -0.641 | 0.007 |
| FLG | <0.001 | -1.573 | <0.001 |
| FLG-AS1 | 0.003 | -0.580 | 0.013 |
| FLG2 | <0.001 | -2.717 | <0.001 |
| FLI1 | <0.001 | 0.354 | <0.001 |
| FLRT2 | <0.001 | 0.464 | <0.001 |
| FLT3LG | <0.001 | 0.266 | <0.001 |
| FLT4 | <0.001 | 0.409 | <0.001 |
| FLVCR2 | <0.001 | -0.421 | <0.001 |
| FMN1 | 0.002 | 0.279 | 0.010 |
| FMNL1 | <0.001 | 0.266 | <0.001 |
| FMNL3 | <0.001 | 0.305 | <0.001 |
| FN1 | <0.001 | 0.306 | <0.001 |
| FNDC1-IT1 | 0.009 | 0.630 | 0.032 |
| FOLR2 | <0.001 | 0.316 | <0.001 |
| FOXA2 | 0.001 | 0.909 | 0.006 |
| FOXB1 | <0.001 | 1.322 | 0.004 |
| FOXC2 | <0.001 | 0.896 | <0.001 |
| FOXC2-AS1 | <0.001 | 1.444 | <0.001 |
| FOXCUT | 0.009 | 0.404 | 0.032 |
| FOXE1 | <0.001 | -0.383 | <0.001 |
| FOXH1 | <0.001 | -1.183 | 0.005 |
| FOXI1 | 0.012 | 1.100 | 0.041 |
| FOXJ1 | 0.007 | -0.630 | 0.027 |
| FOXL1 | <0.001 | 0.930 | <0.001 |
| FOXN1 | <0.001 | -0.494 | <0.001 |
| FOXP2 | <0.001 | -0.587 | <0.001 |
| FOXP3 | <0.001 | 0.305 | <0.001 |
| FOXS1 | <0.001 | 0.512 | <0.001 |
| FP565260.3 | 0.008 | 0.389 | 0.029 |
| FPR3 | <0.001 | 0.266 | <0.001 |
| FRAS1 | 0.009 | -0.389 | 0.031 |
| FRMD3 | <0.001 | 0.411 | 0.002 |
| FRMD5 | <0.001 | 0.720 | <0.001 |
| FRMPD1 | 0.014 | -0.625 | 0.047 |
| FRMPD4 | <0.001 | 1.200 | 0.003 |
| FRS3 | <0.001 | 0.287 | <0.001 |
| FRZB | 0.001 | 0.361 | 0.007 |
| FSCN3 | 0.008 | 0.572 | 0.029 |
| FSD1 | <0.001 | 0.618 | 0.002 |
| FSD1L | <0.001 | 0.539 | <0.001 |
| FSIP1 | <0.001 | 0.557 | <0.001 |
| FSTL3 | <0.001 | 0.295 | <0.001 |
| FTCDNL1 | 0.006 | -0.311 | 0.024 |
| FTH1P10 | <0.001 | 0.400 | 0.003 |
| FTH1P12 | <0.001 | 0.358 | 0.002 |
| FTH1P15 | 0.002 | 0.281 | 0.008 |
| FTH1P2 | <0.001 | 0.273 | 0.001 |
| FTH1P22 | 0.005 | 0.393 | 0.020 |
| FTH1P4 | 0.001 | 0.352 | 0.007 |
| FTH1P5 | 0.002 | 0.273 | 0.011 |
| FTLP10 | <0.001 | -1.658 | <0.001 |
| FTLP14 | <0.001 | -0.297 | <0.001 |
| FTLP15 | 0.004 | 0.789 | 0.016 |
| FTLP2 | <0.001 | 0.325 | <0.001 |
| FUT1 | <0.001 | -0.295 | <0.001 |
| FUT2 | <0.001 | -0.470 | <0.001 |
| FUT3 | <0.001 | -0.635 | <0.001 |
| FUT4 | <0.001 | 0.278 | <0.001 |
| FUT6 | <0.001 | -1.169 | <0.001 |
| FUT7 | <0.001 | 0.426 | 0.004 |
| FUT8 | <0.001 | 0.333 | <0.001 |
| FUT8-AS1 | 0.002 | 0.361 | 0.011 |
| FXYD6 | <0.001 | 0.457 | 0.002 |
| FYB1 | <0.001 | 0.388 | <0.001 |
| FZD2 | <0.001 | 0.332 | <0.001 |
| FZD4 | <0.001 | 0.320 | <0.001 |
| FZD8 | <0.001 | 0.344 | <0.001 |
| GAB3 | <0.001 | 0.426 | <0.001 |
| GABRD | <0.001 | 0.515 | 0.001 |
| GABRR2 | 0.015 | 0.310 | 0.048 |
| GADD45G | <0.001 | 0.316 | <0.001 |
| GALNT12 | <0.001 | -0.309 | <0.001 |
| GALNT16 | <0.001 | 0.711 | 0.005 |
| GALNT17 | <0.001 | 0.897 | <0.001 |
| GALR2 | <0.001 | -0.665 | 0.005 |
| GAN | <0.001 | -0.421 | <0.001 |
| GAP43 | <0.001 | 0.521 | 0.004 |
| GAPDHP14 | 0.008 | -0.572 | 0.031 |
| GAPDHP2 | <0.001 | 0.337 | 0.005 |
| GAPDHP22 | 0.001 | 0.561 | 0.008 |
| GAPDHP32 | <0.001 | 0.890 | 0.002 |
| GAPDHP33 | 0.001 | 0.805 | 0.007 |
| GAPDHP40 | 0.004 | 0.298 | 0.015 |
| GAPDHP44 | 0.010 | 0.332 | 0.034 |
| GAPDHP60 | 0.014 | 0.277 | 0.047 |
| GAPDHP63 | <0.001 | 0.316 | 0.005 |
| GAPDHP67 | <0.001 | 0.683 | 0.002 |
| GAPDHP71 | 0.001 | 0.437 | 0.006 |
| GAPDHP72 | 0.007 | 0.291 | 0.027 |
| GAPDHP73 | 0.008 | 0.391 | 0.028 |
| GAPT | 0.007 | 0.398 | 0.025 |
| GAS1RR | 0.002 | 0.492 | 0.008 |
| GAS2 | 0.015 | 0.755 | 0.049 |
| GAS2L1P2 | 0.003 | 0.633 | 0.015 |
| GASK1B | <0.001 | 0.291 | <0.001 |
| GAST | <0.001 | -0.648 | <0.001 |
| GATA3 | <0.001 | 0.411 | <0.001 |
| GATA6 | <0.001 | 0.618 | <0.001 |
| GATA6-AS1 | 0.004 | 0.866 | 0.016 |
| GBP6 | <0.001 | -0.406 | <0.001 |
| GCC2-AS1 | <0.001 | 0.452 | <0.001 |
| GCKR | 0.006 | -0.559 | 0.023 |
| GCNT1 | <0.001 | 0.296 | <0.001 |
| GCNT2 | 0.009 | -0.359 | 0.032 |
| GCNT3 | <0.001 | -0.810 | <0.001 |
| GCNT4 | <0.001 | -0.303 | 0.003 |
| GCOM1 | <0.001 | -1.584 | <0.001 |
| GCOM2 | <0.001 | -1.201 | <0.001 |
| GDA | <0.001 | -0.847 | <0.001 |
| GDF10 | 0.002 | 0.681 | 0.009 |
| GDF3 | <0.001 | 0.503 | 0.004 |
| GDF6 | <0.001 | 1.043 | <0.001 |
| GDPD2 | <0.001 | -0.564 | <0.001 |
| GDPD3 | <0.001 | -0.875 | <0.001 |
| GDPD5 | <0.001 | 0.684 | <0.001 |
| GEM | <0.001 | 0.343 | <0.001 |
| GFI1 | <0.001 | 0.393 | <0.001 |
| GFI1B | 0.006 | 0.933 | 0.023 |
| GFOD2 | <0.001 | -0.300 | <0.001 |
| GFPT2 | <0.001 | 0.343 | <0.001 |
| GGT6 | <0.001 | -0.583 | <0.001 |
| GGT8P | 0.013 | -0.631 | 0.044 |
| GGTA1P | <0.001 | 0.330 | 0.004 |
| GGTLC4P | 0.003 | 0.586 | 0.012 |
| GHRHR | 0.013 | 0.988 | 0.043 |
| GHRL | 0.003 | 0.417 | 0.013 |
| GHRLOS | 0.004 | 0.290 | 0.015 |
| GIHCG | <0.001 | 0.485 | <0.001 |
| GIMAP1 | <0.001 | 0.359 | <0.001 |
| GIMAP5 | <0.001 | 0.500 | <0.001 |
| GIMAP8 | <0.001 | 0.282 | <0.001 |
| GIPC3 | <0.001 | 0.389 | <0.001 |
| GIPR | 0.014 | -0.307 | 0.047 |
| GJC1 | <0.001 | 0.265 | 0.003 |
| GK | <0.001 | -0.308 | <0.001 |
| GLI2 | 0.001 | -0.400 | 0.006 |
| GLI3 | <0.001 | 0.297 | <0.001 |
| GLIPR1L2 | 0.002 | 0.380 | 0.010 |
| GLIS1 | <0.001 | 0.565 | <0.001 |
| GLIS2 | <0.001 | 0.282 | <0.001 |
| GLIS3 | <0.001 | 0.455 | <0.001 |
| GLRB | <0.001 | 0.604 | <0.001 |
| GLT1D1 | 0.006 | 0.443 | 0.025 |
| GLT8D2 | <0.001 | 0.335 | <0.001 |
| GLTPD2 | <0.001 | -0.537 | <0.001 |
| GLUD1P2 | <0.001 | 0.521 | 0.002 |
| GLYATL1P1 | 0.004 | 0.723 | 0.016 |
| GMPR | <0.001 | 0.421 | <0.001 |
| GNAZ | 0.004 | 0.404 | 0.018 |
| GNB4 | <0.001 | 0.265 | <0.001 |
| GNG2 | <0.001 | 0.472 | <0.001 |
| GNGT2 | <0.001 | 0.431 | <0.001 |
| GNLY | <0.001 | 0.385 | <0.001 |
| GNRHR | <0.001 | -1.247 | <0.001 |
| GOLGA6L22 | 0.005 | -1.410 | 0.022 |
| GOLGA6L9 | 0.014 | -0.275 | 0.046 |
| GOLGA8H | <0.001 | 0.532 | <0.001 |
| GOLGA8K | 0.007 | 0.478 | 0.028 |
| GOLGA8R | <0.001 | 0.582 | <0.001 |
| GORAB-AS1 | <0.001 | 0.633 | <0.001 |
| GP5 | <0.001 | 0.604 | 0.005 |
| GPA33 | <0.001 | 0.632 | 0.004 |
| GPAT3 | <0.001 | -0.371 | <0.001 |
| GPBAR1 | <0.001 | 0.460 | <0.001 |
| GPC4 | <0.001 | 0.536 | <0.001 |
| GPC6 | <0.001 | 0.611 | <0.001 |
| GPLD1 | <0.001 | -1.173 | <0.001 |
| GPR1 | <0.001 | -0.386 | <0.001 |
| GPR137C | 0.012 | 0.370 | 0.042 |
| GPR141 | <0.001 | 0.845 | <0.001 |
| GPR143 | 0.001 | -0.408 | 0.007 |
| GPR15 | 0.003 | -0.509 | 0.012 |
| GPR160 | <0.001 | -0.441 | <0.001 |
| GPR162 | <0.001 | 0.374 | <0.001 |
| GPR173 | <0.001 | 0.725 | <0.001 |
| GPR174 | 0.003 | 0.483 | 0.015 |
| GPR18 | 0.002 | 0.498 | 0.009 |
| GPR182 | <0.001 | 0.569 | <0.001 |
| GPR31 | 0.005 | 0.673 | 0.021 |
| GPR34 | <0.001 | 0.409 | <0.001 |
| GPR35 | 0.002 | 0.329 | 0.008 |
| GPR37L1 | <0.001 | -0.604 | 0.002 |
| GPR39 | 0.008 | 0.272 | 0.029 |
| GPR65 | <0.001 | 0.431 | <0.001 |
| GPR78 | <0.001 | -0.995 | <0.001 |
| GPR82 | <0.001 | 0.610 | <0.001 |
| GPR84 | <0.001 | 0.389 | <0.001 |
| GPRACR | 0.007 | 0.622 | 0.025 |
| GPRASP1 | 0.002 | 0.277 | 0.010 |
| GPRC5C | <0.001 | 0.278 | <0.001 |
| GPRC5D | <0.001 | -0.761 | <0.001 |
| GPRIN3 | <0.001 | 0.388 | <0.001 |
| GPT2 | <0.001 | -0.306 | <0.001 |
| GPX2 | <0.001 | -0.408 | <0.001 |
| GPX7 | <0.001 | 0.332 | <0.001 |
| GRAMD1B | <0.001 | 0.533 | <0.001 |
| GRAMD1C | <0.001 | -0.399 | <0.001 |
| GRAP | 0.001 | 0.278 | 0.007 |
| GRAP2 | 0.005 | 0.331 | 0.021 |
| GRB10 | <0.001 | 0.303 | <0.001 |
| GREB1L | <0.001 | 1.036 | <0.001 |
| GRHL1 | <0.001 | -0.595 | <0.001 |
| GRHL3 | <0.001 | -0.586 | <0.001 |
| GRHL3-AS1 | <0.001 | -0.552 | <0.001 |
| GRIA3 | 0.002 | 0.551 | 0.009 |
| GRID1 | <0.001 | 0.373 | 0.005 |
| GRIN2A | <0.001 | 1.414 | 0.004 |
| GRIN3A | 0.006 | 0.340 | 0.024 |
| GRIP1 | <0.001 | 0.326 | <0.001 |
| GRIP2 | 0.007 | 0.338 | 0.027 |
| GRK7 | 0.004 | -0.761 | 0.018 |
| GRM2 | <0.001 | 0.338 | 0.004 |
| GRM4 | <0.001 | 1.253 | <0.001 |
| GRP | <0.001 | 0.676 | <0.001 |
| GRPEL2-AS1 | <0.001 | -0.592 | <0.001 |
| GSC | <0.001 | 0.482 | 0.002 |
| GSDMA | <0.001 | -0.857 | <0.001 |
| GSEC | <0.001 | 0.322 | <0.001 |
| GSTA1 | <0.001 | -0.784 | <0.001 |
| GSTA3 | <0.001 | -2.166 | <0.001 |
| GSTA4 | <0.001 | -0.393 | <0.001 |
| GSTA9P | <0.001 | -1.452 | <0.001 |
| GSTM2 | 0.001 | -0.466 | 0.006 |
| GSTM3 | 0.002 | -0.297 | 0.008 |
| GTF2F2P1 | 0.004 | 0.408 | 0.016 |
| GTF2H2 | <0.001 | 0.349 | 0.004 |
| GTF2H2B | 0.003 | 0.377 | 0.014 |
| GTF2H2C | <0.001 | 0.282 | <0.001 |
| GTF2IP7 | <0.001 | -1.064 | <0.001 |
| GTF2IP9 | 0.010 | 0.326 | 0.035 |
| GTSE1-DT | 0.008 | -0.267 | 0.030 |
| GUCA1B | 0.007 | 0.267 | 0.025 |
| GUCY1A1 | <0.001 | 0.301 | <0.001 |
| GUCY1A2 | <0.001 | 0.418 | <0.001 |
| GUCY1B1 | <0.001 | 0.294 | <0.001 |
| GUCY2C | <0.001 | -0.536 | 0.001 |
| GVINP1 | 0.005 | 0.394 | 0.021 |
| GVQW1 | <0.001 | 0.280 | <0.001 |
| GVQW2 | 0.006 | 0.344 | 0.023 |
| GXYLT2 | <0.001 | 0.523 | <0.001 |
| GYPC | <0.001 | 0.299 | <0.001 |
| GYPE | 0.003 | 0.493 | 0.013 |
| GYS2 | 0.013 | -1.401 | 0.043 |
| GZMH | 0.015 | 0.263 | 0.049 |
| GZMK | 0.005 | 0.351 | 0.020 |
| GZMM | 0.007 | 0.306 | 0.025 |
| H1-9 | <0.001 | 1.167 | <0.001 |
| H2AC12 | 0.011 | 0.366 | 0.039 |
| H2BC6 | 0.011 | -0.337 | 0.037 |
| H2BC7 | <0.001 | -0.419 | 0.005 |
| H2BC8 | <0.001 | -0.291 | <0.001 |
| H2BP1 | 0.004 | 0.574 | 0.018 |
| H2BU1 | 0.010 | -0.331 | 0.035 |
| H2BW3P | 0.014 | 0.621 | 0.047 |
| H3C1 | 0.001 | -0.446 | 0.006 |
| H3C10 | <0.001 | -0.266 | 0.001 |
| H3C6 | <0.001 | -0.384 | <0.001 |
| H4-16 | 0.002 | -0.304 | 0.010 |
| H4C10P | 0.009 | -0.519 | 0.033 |
| HAGLR | 0.010 | 0.280 | 0.035 |
| HAL | <0.001 | -2.289 | <0.001 |
| HAND2 | <0.001 | 0.671 | 0.003 |
| HAND2-AS1 | 0.013 | 0.992 | 0.043 |
| HAP1 | 0.002 | -0.365 | 0.008 |
| HAS2 | <0.001 | 0.389 | <0.001 |
| HAS2-AS1 | <0.001 | 0.649 | <0.001 |
| HAS3 | <0.001 | -0.285 | <0.001 |
| HAVCR2 | <0.001 | 0.353 | <0.001 |
| HBE1 | 0.001 | -1.346 | 0.006 |
| HBG2 | 0.008 | -1.145 | 0.031 |
| HCAR1 | 0.005 | -1.019 | 0.021 |
| HCG17 | 0.003 | 0.436 | 0.013 |
| HCG22 | <0.001 | -1.195 | 0.002 |
| HCG9 | 0.016 | 0.496 | 0.050 |
| HDAC9 | <0.001 | 0.437 | <0.001 |
| HDX | 0.004 | 0.356 | 0.016 |
| HECW1 | <0.001 | 0.689 | <0.001 |
| HECW2-AS1 | <0.001 | 0.535 | <0.001 |
| HELB | <0.001 | 0.273 | <0.001 |
| HEMGN | 0.011 | 0.684 | 0.038 |
| HEPH | 0.001 | 0.278 | 0.006 |
| HERC2P10 | 0.013 | 0.388 | 0.045 |
| HES5 | <0.001 | -1.016 | <0.001 |
| HESX1 | <0.001 | 0.435 | <0.001 |
| HEXA-AS1 | 0.014 | -0.283 | 0.047 |
| HEYL | <0.001 | 0.340 | <0.001 |
| HFM1 | 0.002 | 1.170 | 0.009 |
| HGF | 0.003 | 0.587 | 0.013 |
| HHATL | 0.011 | 0.704 | 0.038 |
| HHIP-AS1 | 0.004 | 0.625 | 0.016 |
| HHIPL1 | <0.001 | 0.594 | <0.001 |
| HHLA2 | <0.001 | -1.411 | 0.004 |
| HIC1 | <0.001 | 0.402 | <0.001 |
| HIPK4 | <0.001 | 0.561 | 0.001 |
| HIVEP3 | <0.001 | 0.424 | <0.001 |
| HK2P1 | <0.001 | -0.643 | <0.001 |
| HK3 | 0.009 | 0.271 | 0.031 |
| HKDC1 | <0.001 | -0.936 | <0.001 |
| HLA-DOA | <0.001 | 0.270 | 0.005 |
| HLA-DPA3 | 0.005 | 0.482 | 0.021 |
| HLA-DPB2 | 0.002 | 0.484 | 0.009 |
| HLA-DQA2 | <0.001 | 0.291 | <0.001 |
| HLA-DQB1-AS1 | 0.004 | 0.322 | 0.018 |
| HLA-DRB9 | 0.003 | 0.404 | 0.012 |
| HLA-F-AS1 | <0.001 | 0.477 | <0.001 |
| HLA-L | <0.001 | 0.334 | 0.001 |
| HLA-V | <0.001 | 0.479 | 0.001 |
| HLA-W | <0.001 | 0.565 | <0.001 |
| HLF | <0.001 | -0.538 | <0.001 |
| HLX | <0.001 | 0.388 | <0.001 |
| HLX-AS1 | <0.001 | 0.849 | 0.003 |
| HMCN1 | <0.001 | 0.436 | <0.001 |
| HMGA1P3 | <0.001 | -0.464 | <0.001 |
| HMGA2-AS1 | 0.006 | 0.375 | 0.022 |
| HMGB1P1 | <0.001 | 0.361 | <0.001 |
| HMGB1P11 | 0.014 | 0.449 | 0.046 |
| HMGB1P20 | <0.001 | 0.371 | 0.002 |
| HMGB1P3 | 0.014 | 0.343 | 0.045 |
| HMGB1P37 | <0.001 | 0.429 | 0.002 |
| HMGB1P49 | 0.007 | 0.554 | 0.028 |
| HMGB1P8 | <0.001 | 0.341 | 0.001 |
| HMGB3P22 | 0.004 | 0.535 | 0.016 |
| HMGCLL1 | 0.005 | 1.082 | 0.020 |
| HMGN1P13 | 0.005 | 0.563 | 0.022 |
| HMGN1P7 | <0.001 | -0.959 | <0.001 |
| HMGN2P10 | 0.013 | 0.481 | 0.042 |
| HMGN2P18 | 0.015 | 0.472 | 0.050 |
| HMGN2P19 | 0.008 | 0.442 | 0.029 |
| HMGN2P23 | <0.001 | -1.013 | <0.001 |
| HMGN2P27 | 0.009 | 0.404 | 0.034 |
| HMGN2P31 | 0.007 | 0.397 | 0.027 |
| HMGN2P4 | <0.001 | 0.300 | 0.001 |
| HMGN2P6 | <0.001 | 0.396 | 0.001 |
| HNMT | <0.001 | 0.340 | <0.001 |
| HNRNPA1P21 | <0.001 | 0.284 | <0.001 |
| HNRNPA1P25 | 0.008 | 0.425 | 0.029 |
| HNRNPA1P34 | 0.013 | 0.417 | 0.043 |
| HNRNPA1P50 | 0.009 | 0.315 | 0.033 |
| HNRNPA3P7 | 0.007 | 0.528 | 0.026 |
| HNRNPDP1 | 0.014 | 0.361 | 0.047 |
| HNRNPLP1 | 0.006 | -0.437 | 0.025 |
| HOGA1 | <0.001 | -0.680 | <0.001 |
| HOPX | <0.001 | -0.694 | <0.001 |
| HOTAIR | <0.001 | 0.829 | <0.001 |
| HOXA11 | <0.001 | 0.509 | 0.004 |
| HOXA11-AS | 0.012 | 0.578 | 0.041 |
| HOXB1 | <0.001 | -1.282 | <0.001 |
| HOXB13 | 0.001 | 0.747 | 0.008 |
| HOXB9 | <0.001 | 0.593 | 0.004 |
| HOXC-AS1 | 0.010 | 0.342 | 0.036 |
| HOXC-AS2 | 0.001 | 0.351 | 0.007 |
| HOXC-AS3 | 0.008 | 0.803 | 0.031 |
| HOXC10 | <0.001 | 0.367 | 0.005 |
| HOXC11 | <0.001 | 0.561 | <0.001 |
| HOXC4 | <0.001 | 0.508 | <0.001 |
| HOXC5 | <0.001 | 0.641 | 0.001 |
| HOXC6 | <0.001 | 0.447 | <0.001 |
| HOXC8 | 0.009 | 0.321 | 0.031 |
| HOXC9 | <0.001 | 0.406 | <0.001 |
| HPCAL4 | 0.004 | 0.611 | 0.016 |
| HPSE | <0.001 | -0.330 | <0.001 |
| HRC | 0.004 | 0.576 | 0.016 |
| HRCT1 | <0.001 | 0.532 | 0.003 |
| HRH2 | <0.001 | 0.627 | <0.001 |
| HRH3 | <0.001 | -1.185 | 0.002 |
| HRNR | <0.001 | -1.880 | <0.001 |
| HS3ST3B1 | <0.001 | 0.326 | <0.001 |
| HS3ST6 | <0.001 | -0.922 | <0.001 |
| HSD11B1-AS1 | <0.001 | 0.469 | 0.005 |
| HSD11B2 | <0.001 | -0.313 | <0.001 |
| HSD17B14 | <0.001 | 0.345 | <0.001 |
| HSD3BP5 | <0.001 | 0.638 | <0.001 |
| HSF2BP | <0.001 | 0.388 | <0.001 |
| HSF5 | 0.014 | 1.041 | 0.046 |
| HSP90B3P | <0.001 | 0.446 | 0.004 |
| HSPA12B | <0.001 | 0.263 | <0.001 |
| HSPA4L | <0.001 | -0.331 | <0.001 |
| HSPA7 | 0.002 | 0.310 | 0.008 |
| HSPA8P15 | 0.015 | 0.310 | 0.047 |
| HSPA8P4 | 0.012 | 0.318 | 0.041 |
| HSPB7 | 0.009 | 0.473 | 0.033 |
| HSPD1P6 | <0.001 | 0.403 | <0.001 |
| HTR1B | 0.002 | 0.461 | 0.011 |
| HTR2A | <0.001 | 0.726 | 0.001 |
| HTR3A | <0.001 | -1.377 | <0.001 |
| HTR3B | <0.001 | -2.196 | <0.001 |
| HTR6 | <0.001 | 0.973 | <0.001 |
| HUNK | 0.008 | 0.351 | 0.031 |
| HVCN1 | <0.001 | 0.493 | <0.001 |
| HYAL1 | <0.001 | -0.353 | <0.001 |
| HYAL4 | 0.001 | -0.784 | 0.006 |
| IATPR | 0.001 | 0.757 | 0.006 |
| IBSP | <0.001 | 0.547 | 0.002 |
| ICAM5 | <0.001 | 0.468 | <0.001 |
| ICOS | <0.001 | 0.420 | <0.001 |
| IFFO1 | <0.001 | 0.322 | <0.001 |
| IFI30 | <0.001 | 0.343 | <0.001 |
| IFIT1B | 0.002 | 0.650 | 0.010 |
| IFIT1P1 | 0.014 | 0.417 | 0.045 |
| IFIT2 | <0.001 | 0.271 | <0.001 |
| IFITM4P | 0.011 | 0.421 | 0.037 |
| IFITM9P | <0.001 | 0.495 | <0.001 |
| IFNA1 | 0.003 | 0.769 | 0.012 |
| IFNA20P | <0.001 | 0.408 | 0.002 |
| IFNG | 0.012 | 0.420 | 0.040 |
| IFNWP2 | 0.005 | 0.626 | 0.020 |
| IGDCC3 | 0.008 | 0.933 | 0.030 |
| IGDCC4 | <0.001 | 0.415 | 0.002 |
| IGFBP7-AS1 | <0.001 | 1.021 | 0.003 |
| IGFL1 | 0.003 | -0.271 | 0.012 |
| IGFL1P1 | 0.003 | -0.392 | 0.012 |
| IGFL2-AS1 | 0.003 | -0.387 | 0.013 |
| IGHV1-67 | 0.011 | -0.454 | 0.038 |
| IGHV1OR15-9 | 0.006 | -0.489 | 0.023 |
| IGHV3-36 | 0.013 | -0.706 | 0.044 |
| IGHV3-63 | 0.014 | -0.433 | 0.047 |
| IGHV3OR16-16 | 0.011 | -0.730 | 0.037 |
| IGHV7-27 | 0.014 | -0.538 | 0.045 |
| IGKV1D-17 | 0.004 | -0.672 | 0.017 |
| IGKV1D-27 | 0.010 | -0.622 | 0.037 |
| IGKV1D-42 | 0.010 | -0.532 | 0.034 |
| IGKV1OR2-6 | 0.015 | -0.458 | 0.049 |
| IGLV2-18 | 0.014 | -0.361 | 0.047 |
| IGLV8-61 | 0.015 | -0.304 | 0.049 |
| IGSF21 | <0.001 | 0.609 | <0.001 |
| IGSF22 | <0.001 | -0.575 | <0.001 |
| IGSF6 | <0.001 | 0.374 | <0.001 |
| IKZF1 | <0.001 | 0.359 | 0.002 |
| IKZF2 | <0.001 | -0.335 | <0.001 |
| IL10 | 0.004 | 0.285 | 0.019 |
| IL11 | <0.001 | 0.315 | 0.003 |
| IL11RA | <0.001 | 0.414 | <0.001 |
| IL12B | 0.009 | 0.449 | 0.031 |
| IL12RB1 | <0.001 | 0.444 | <0.001 |
| IL15 | <0.001 | 0.480 | <0.001 |
| IL16 | <0.001 | 0.396 | <0.001 |
| IL17C | <0.001 | -1.535 | <0.001 |
| IL17D | 0.002 | 0.452 | 0.010 |
| IL17REL | 0.005 | 1.250 | 0.021 |
| IL18BP | <0.001 | 0.326 | <0.001 |
| IL18R1 | <0.001 | 0.370 | <0.001 |
| IL19 | <0.001 | -1.039 | 0.004 |
| IL1F10 | <0.001 | -1.959 | <0.001 |
| IL1RN | <0.001 | -0.345 | <0.001 |
| IL2 | 0.013 | 0.588 | 0.044 |
| IL20 | 0.003 | 0.488 | 0.014 |
| IL20RA | <0.001 | -0.306 | <0.001 |
| IL20RB-AS1 | 0.011 | -0.320 | 0.039 |
| IL21R | <0.001 | 0.519 | <0.001 |
| IL21R-AS1 | 0.002 | 0.621 | 0.008 |
| IL22RA1 | <0.001 | -0.328 | <0.001 |
| IL22RA2 | <0.001 | 0.802 | <0.001 |
| IL27 | <0.001 | 0.569 | 0.003 |
| IL2RA | <0.001 | 0.365 | <0.001 |
| IL36A | <0.001 | -1.379 | <0.001 |
| IL36B | <0.001 | -1.621 | <0.001 |
| IL36G | <0.001 | -0.591 | <0.001 |
| IL36RN | <0.001 | -0.910 | <0.001 |
| IL37 | <0.001 | -1.181 | <0.001 |
| IL4 | <0.001 | 0.899 | <0.001 |
| IL4I1 | <0.001 | 0.342 | <0.001 |
| IL6-AS1 | <0.001 | 0.718 | 0.003 |
| IL7R | <0.001 | 0.306 | <0.001 |
| ILDR1 | <0.001 | -0.297 | 0.004 |
| ILDR2 | 0.008 | 0.658 | 0.031 |
| IMPDH1P5 | <0.001 | -0.362 | 0.003 |
| IMPG1 | 0.006 | 0.393 | 0.024 |
| INHA | 0.015 | 0.396 | 0.047 |
| INHBA | <0.001 | 0.309 | <0.001 |
| INHBA-AS1 | <0.001 | 0.719 | <0.001 |
| INO80-AS1 | 0.010 | -0.410 | 0.035 |
| INO80B-WBP1 | 0.015 | -0.352 | 0.047 |
| INPP5J | <0.001 | -0.337 | 0.004 |
| INSL3 | 0.006 | 0.371 | 0.023 |
| INSM1 | 0.014 | 1.185 | 0.045 |
| INSRR | <0.001 | 0.865 | <0.001 |
| INSYN1-AS1 | 0.010 | -1.082 | 0.034 |
| INSYN2A | 0.002 | 0.696 | 0.010 |
| IP6K3 | 0.006 | 0.531 | 0.023 |
| IPCEF1 | <0.001 | 0.443 | 0.004 |
| IPO7P2 | 0.004 | 0.430 | 0.018 |
| IPPK | <0.001 | -0.307 | <0.001 |
| IQCH | 0.002 | 0.299 | 0.011 |
| IQGAP2 | <0.001 | 0.456 | <0.001 |
| IQSEC3 | 0.003 | 0.584 | 0.015 |
| IRAK1BP1 | <0.001 | 0.340 | 0.002 |
| IRAK3 | <0.001 | 0.337 | 0.002 |
| IRF1-AS1 | <0.001 | 0.470 | <0.001 |
| IRF8 | <0.001 | 0.324 | <0.001 |
| IRGM | 0.007 | 0.628 | 0.028 |
| ISM1-AS1 | <0.001 | 0.757 | 0.002 |
| ITGA1 | <0.001 | 0.359 | <0.001 |
| ITGA10 | <0.001 | 0.430 | <0.001 |
| ITGA11 | <0.001 | 0.415 | 0.002 |
| ITGA4 | <0.001 | 0.460 | <0.001 |
| ITGA5 | <0.001 | 0.293 | <0.001 |
| ITGA7 | <0.001 | 0.341 | <0.001 |
| ITGA9 | 0.001 | 0.271 | 0.006 |
| ITGAL | 0.002 | 0.274 | 0.009 |
| ITGB1-DT | <0.001 | 0.791 | <0.001 |
| ITGB1BP2 | 0.008 | 0.356 | 0.030 |
| ITGB2-AS1 | 0.005 | 0.361 | 0.022 |
| ITGB3 | <0.001 | 0.690 | <0.001 |
| ITGBL1 | 0.009 | 0.374 | 0.032 |
| ITIH4 | <0.001 | 0.568 | 0.003 |
| ITK | 0.002 | 0.374 | 0.011 |
| ITPR1-DT | 0.001 | 0.448 | 0.006 |
| ITPRIP-AS1 | <0.001 | -0.533 | <0.001 |
| IVL | <0.001 | -0.505 | <0.001 |
| IZUMO1 | <0.001 | -0.949 | <0.001 |
| JAKMIP1 | <0.001 | 0.491 | 0.004 |
| JARID2-AS1 | <0.001 | -0.394 | 0.005 |
| JAZF1 | <0.001 | 0.416 | <0.001 |
| JAZF1-AS1 | <0.001 | 0.685 | <0.001 |
| JCAD | <0.001 | 0.290 | <0.001 |
| JMJD1C-AS1 | 0.002 | 0.273 | 0.010 |
| JMJD7 | <0.001 | -0.592 | <0.001 |
| JMJD7-PLA2G4B | <0.001 | -0.357 | 0.001 |
| JPH2 | <0.001 | 0.673 | <0.001 |
| KANK4 | <0.001 | 0.923 | <0.001 |
| KANTR | 0.003 | 0.539 | 0.014 |
| KATNAL1 | <0.001 | 0.447 | <0.001 |
| KAZALD1 | <0.001 | -0.336 | <0.001 |
| KAZN | <0.001 | -0.275 | <0.001 |
| KB-1458E12.1 | 0.009 | 0.478 | 0.032 |
| KB-1507C5.4 | <0.001 | 0.706 | <0.001 |
| KBTBD11 | 0.007 | 0.314 | 0.026 |
| KBTBD12 | 0.013 | 0.758 | 0.042 |
| KCNA2 | 0.008 | 1.195 | 0.028 |
| KCNA5 | 0.007 | 0.545 | 0.027 |
| KCNA7 | 0.003 | 0.709 | 0.015 |
| KCNB1 | 0.012 | 0.860 | 0.041 |
| KCNB2 | <0.001 | 1.712 | <0.001 |
| KCND1 | <0.001 | 0.447 | <0.001 |
| KCND2 | 0.001 | 0.455 | 0.007 |
| KCND3 | 0.007 | 0.268 | 0.027 |
| KCNE4 | <0.001 | 0.491 | <0.001 |
| KCNE5 | 0.008 | 0.396 | 0.029 |
| KCNF1 | <0.001 | 0.841 | <0.001 |
| KCNG1 | <0.001 | 0.565 | 0.001 |
| KCNG2 | <0.001 | 0.449 | 0.004 |
| KCNH1 | 0.012 | -0.698 | 0.041 |
| KCNH1-IT1 | 0.003 | -0.834 | 0.014 |
| KCNH3 | 0.002 | 0.513 | 0.009 |
| KCNIP1 | 0.010 | 0.927 | 0.035 |
| KCNIP3 | <0.001 | 0.613 | <0.001 |
| KCNJ1 | 0.006 | 0.517 | 0.023 |
| KCNJ18 | <0.001 | -1.743 | <0.001 |
| KCNJ5 | <0.001 | 0.742 | <0.001 |
| KCNJ6 | <0.001 | 1.110 | <0.001 |
| KCNJ8 | <0.001 | 0.305 | <0.001 |
| KCNK17 | <0.001 | 0.569 | 0.003 |
| KCNK3 | 0.002 | 0.970 | 0.011 |
| KCNK7 | <0.001 | -0.790 | <0.001 |
| KCNMA1 | 0.005 | 0.318 | 0.022 |
| KCNMB1 | <0.001 | 0.545 | <0.001 |
| KCNMB4 | <0.001 | 0.728 | <0.001 |
| KCNN2 | <0.001 | 0.732 | <0.001 |
| KCNN3 | 0.003 | 0.307 | 0.014 |
| KCNN4 | <0.001 | 0.269 | <0.001 |
| KCNQ3 | 0.004 | 0.724 | 0.018 |
| KCNT1 | 0.005 | 0.817 | 0.019 |
| KCNT2 | <0.001 | 0.872 | <0.001 |
| KCP | 0.001 | -0.352 | 0.006 |
| KCTD4 | <0.001 | -1.888 | <0.001 |
| KCTD8 | 0.007 | 1.028 | 0.027 |
| KCTD9P4 | <0.001 | -0.643 | 0.002 |
| KERA | 0.015 | 0.835 | 0.049 |
| KHDC1L | <0.001 | -0.753 | <0.001 |
| KHDRBS2 | 0.004 | 0.776 | 0.018 |
| KIAA0319 | 0.002 | -0.866 | 0.011 |
| KIAA0408 | <0.001 | 1.432 | 0.001 |
| KIAA1107 | <0.001 | 0.274 | 0.005 |
| KIAA1257 | <0.001 | -0.954 | 0.003 |
| KIAA1549L | <0.001 | 0.502 | <0.001 |
| KIAA1614 | <0.001 | 0.461 | <0.001 |
| KIAA1755 | <0.001 | 0.847 | <0.001 |
| KIF12 | <0.001 | 0.938 | <0.001 |
| KIF21B | <0.001 | 0.534 | <0.001 |
| KIF25 | 0.001 | 1.237 | 0.006 |
| KIF26B | <0.001 | 0.285 | 0.001 |
| KIF4B | 0.015 | 0.298 | 0.049 |
| KIF7 | <0.001 | 0.274 | <0.001 |
| KIF9-AS1 | <0.001 | 0.530 | <0.001 |
| KIR2DL3 | 0.012 | 0.755 | 0.041 |
| KIR2DS4 | 0.008 | 0.896 | 0.029 |
| KIRREL2 | 0.013 | 0.705 | 0.043 |
| KIRREL3 | 0.003 | 0.824 | 0.015 |
| KIZ-AS1 | 0.007 | 0.508 | 0.027 |
| KLB | 0.004 | -0.456 | 0.018 |
| KLF17 | 0.005 | 0.823 | 0.022 |
| KLF4 | <0.001 | -0.277 | <0.001 |
| KLF8 | <0.001 | -0.510 | <0.001 |
| KLHDC1 | <0.001 | 0.415 | <0.001 |
| KLHL29 | 0.001 | 0.296 | 0.007 |
| KLHL2P1 | <0.001 | -0.446 | 0.002 |
| KLHL30 | 0.002 | 0.578 | 0.010 |
| KLHL30-AS1 | 0.003 | 0.872 | 0.012 |
| KLHL33 | 0.001 | 1.044 | 0.007 |
| KLHL35 | 0.009 | 0.498 | 0.032 |
| KLHL38 | <0.001 | 0.935 | 0.003 |
| KLHL4 | <0.001 | 0.983 | <0.001 |
| KLHL41 | 0.015 | 0.492 | 0.048 |
| KLHL6 | 0.001 | 0.284 | 0.007 |
| KLK1 | <0.001 | -0.424 | 0.001 |
| KLK10 | <0.001 | -0.588 | <0.001 |
| KLK11 | <0.001 | -0.718 | <0.001 |
| KLK12 | <0.001 | -1.371 | <0.001 |
| KLK13 | <0.001 | -1.002 | <0.001 |
| KLK14 | <0.001 | -1.186 | <0.001 |
| KLK5 | <0.001 | -0.451 | <0.001 |
| KLK6 | <0.001 | -0.728 | <0.001 |
| KLK7 | <0.001 | -0.581 | <0.001 |
| KLK8 | <0.001 | -0.529 | <0.001 |
| KLK9 | <0.001 | -0.944 | <0.001 |
| KLRD1 | 0.002 | 0.458 | 0.011 |
| KLRG1 | <0.001 | 0.495 | 0.001 |
| KLRG2 | <0.001 | -0.451 | <0.001 |
| KMO | <0.001 | 0.595 | <0.001 |
| KPNA7 | <0.001 | -0.598 | <0.001 |
| KPRP | <0.001 | -1.374 | <0.001 |
| KRT1 | <0.001 | -0.547 | <0.001 |
| KRT10 | <0.001 | -0.276 | <0.001 |
| KRT13 | <0.001 | -0.534 | <0.001 |
| KRT16P1 | <0.001 | -0.740 | <0.001 |
| KRT16P2 | <0.001 | -0.442 | 0.001 |
| KRT16P3 | <0.001 | -0.681 | <0.001 |
| KRT17P1 | 0.002 | -0.356 | 0.010 |
| KRT17P2 | 0.002 | -0.383 | 0.010 |
| KRT17P3 | 0.005 | -0.324 | 0.019 |
| KRT17P5 | <0.001 | -0.921 | <0.001 |
| KRT17P6 | 0.001 | -0.340 | 0.008 |
| KRT18P22 | <0.001 | 0.836 | <0.001 |
| KRT18P29 | 0.002 | -0.713 | 0.010 |
| KRT18P3 | 0.005 | 0.640 | 0.020 |
| KRT18P34 | 0.001 | -0.349 | 0.006 |
| KRT18P59 | <0.001 | -0.860 | <0.001 |
| KRT2 | <0.001 | -1.111 | <0.001 |
| KRT23 | <0.001 | -0.668 | <0.001 |
| KRT24 | <0.001 | -1.138 | <0.001 |
| KRT27 | 0.015 | -0.929 | 0.050 |
| KRT3 | <0.001 | -1.585 | <0.001 |
| KRT33A | <0.001 | -0.916 | <0.001 |
| KRT33B | <0.001 | -1.247 | <0.001 |
| KRT37 | <0.001 | -1.207 | <0.001 |
| KRT4 | <0.001 | -0.776 | <0.001 |
| KRT42P | <0.001 | -0.917 | <0.001 |
| KRT6C | <0.001 | -0.304 | <0.001 |
| KRT75 | 0.008 | -0.264 | 0.030 |
| KRT76 | 0.002 | -1.131 | 0.011 |
| KRT77 | 0.001 | -0.941 | 0.006 |
| KRT78 | <0.001 | -1.260 | <0.001 |
| KRT79 | <0.001 | -1.204 | <0.001 |
| KRT80 | <0.001 | -0.520 | <0.001 |
| KRT82 | <0.001 | -1.066 | <0.001 |
| KRT87P | 0.002 | 0.877 | 0.008 |
| KRT89P | <0.001 | -0.834 | 0.004 |
| KRT8P1 | <0.001 | -0.783 | 0.003 |
| KRT8P10 | <0.001 | -0.817 | 0.003 |
| KRT8P13 | 0.015 | -0.333 | 0.049 |
| KRT8P18 | 0.003 | -0.875 | 0.014 |
| KRT8P19 | <0.001 | -1.070 | 0.003 |
| KRT8P21 | <0.001 | -1.201 | <0.001 |
| KRT8P22 | <0.001 | -0.913 | 0.004 |
| KRT8P26 | <0.001 | -1.128 | <0.001 |
| KRT8P31 | 0.007 | -0.610 | 0.026 |
| KRT8P38 | <0.001 | -0.979 | 0.001 |
| KRT8P4 | <0.001 | -1.297 | 0.002 |
| KRT8P40 | 0.003 | -1.709 | 0.013 |
| KRT8P41 | 0.001 | -0.720 | 0.006 |
| KRT8P42 | 0.003 | -0.483 | 0.013 |
| KRT8P43 | <0.001 | -0.948 | 0.003 |
| KRT8P49 | 0.011 | -0.503 | 0.037 |
| KRT8P7 | 0.004 | -0.476 | 0.017 |
| KRT8P9 | <0.001 | -0.646 | 0.001 |
| KRT9 | <0.001 | -0.588 | <0.001 |
| KRTAP5-AS1 | 0.014 | -0.456 | 0.045 |
| KRTDAP | <0.001 | -0.637 | <0.001 |
| KY | 0.010 | 0.624 | 0.035 |
| L1CAM | <0.001 | 0.760 | <0.001 |
| L3MBTL1 | 0.005 | 0.318 | 0.020 |
| LAG3 | <0.001 | 0.374 | <0.001 |
| LAIR1 | <0.001 | 0.396 | <0.001 |
| LAIR2 | 0.012 | 0.290 | 0.041 |
| LAMA2 | 0.002 | 0.299 | 0.008 |
| LAMA4-AS1 | <0.001 | 0.559 | 0.002 |
| LAMC1-AS1 | <0.001 | 0.414 | <0.001 |
| LAMP5 | <0.001 | 0.377 | <0.001 |
| LAMTOR5-AS1 | 0.001 | 0.326 | 0.006 |
| LANCL1-AS1 | 0.005 | 0.544 | 0.020 |
| LANCL3 | <0.001 | -0.745 | <0.001 |
| LARP6 | <0.001 | 0.463 | <0.001 |
| LBX1 | 0.004 | 0.912 | 0.015 |
| LBX1-AS1 | 0.014 | 0.743 | 0.046 |
| LCA5 | <0.001 | 0.425 | <0.001 |
| LCA5L | <0.001 | 0.356 | 0.002 |
| LCE1A | <0.001 | -1.651 | <0.001 |
| LCE1B | <0.001 | -1.742 | <0.001 |
| LCE1C | <0.001 | -0.721 | <0.001 |
| LCE1E | <0.001 | -1.379 | <0.001 |
| LCE1F | <0.001 | -1.126 | <0.001 |
| LCE2A | <0.001 | -2.001 | <0.001 |
| LCE2B | <0.001 | -2.357 | <0.001 |
| LCE2C | <0.001 | -2.262 | <0.001 |
| LCE2D | <0.001 | -2.386 | <0.001 |
| LCE3A | <0.001 | -0.779 | <0.001 |
| LCE3D | <0.001 | -0.564 | <0.001 |
| LCE3E | <0.001 | -0.808 | <0.001 |
| LCE5A | 0.002 | -0.884 | 0.008 |
| LCE6A | <0.001 | -2.359 | <0.001 |
| LCN1 | 0.009 | 0.946 | 0.034 |
| LCN2 | <0.001 | -0.456 | <0.001 |
| LCP2 | <0.001 | 0.330 | <0.001 |
| LCT | 0.002 | -1.197 | 0.008 |
| LCT-AS1 | <0.001 | 0.686 | <0.001 |
| LCTL | 0.002 | 0.393 | 0.011 |
| LDB3 | 0.014 | 0.548 | 0.046 |
| LDHD | <0.001 | -0.498 | <0.001 |
| LDLRAD1 | <0.001 | -1.139 | 0.002 |
| LDLRAD2 | <0.001 | 0.312 | <0.001 |
| LDLRAD4 | <0.001 | 0.459 | <0.001 |
| LEF1-AS1 | <0.001 | 0.618 | <0.001 |
| LEXM | <0.001 | -1.474 | <0.001 |
| LGALS4 | <0.001 | -1.195 | 0.003 |
| LGALS7 | <0.001 | -0.410 | 0.003 |
| LGALSL | <0.001 | -0.391 | <0.001 |
| LGALSL-DT | <0.001 | -0.564 | <0.001 |
| LGI2 | <0.001 | 0.846 | <0.001 |
| LGI3 | <0.001 | -1.217 | <0.001 |
| LGMNP1 | 0.003 | 0.299 | 0.013 |
| LGR5 | <0.001 | 0.804 | <0.001 |
| LHB | <0.001 | 0.559 | <0.001 |
| LHX1 | 0.005 | 0.501 | 0.021 |
| LHX1-DT | 0.004 | 0.511 | 0.016 |
| LHX8 | 0.010 | 0.370 | 0.036 |
| LIF | <0.001 | 0.324 | 0.004 |
| LIFR | <0.001 | 0.697 | <0.001 |
| LIFR-AS1 | <0.001 | 0.639 | 0.002 |
| LILRA4 | 0.006 | 0.455 | 0.023 |
| LILRA6 | 0.001 | 0.367 | 0.006 |
| LILRB1 | <0.001 | 0.344 | 0.003 |
| LILRB2 | <0.001 | 0.283 | 0.003 |
| LILRB3 | 0.002 | 0.282 | 0.010 |
| LILRB4 | 0.006 | 0.264 | 0.025 |
| LILRB5 | 0.001 | 0.390 | 0.006 |
| LIM2 | <0.001 | -2.550 | <0.001 |
| LIMD1-AS1 | 0.001 | 0.474 | 0.007 |
| LIMS1-AS1 | <0.001 | 0.441 | 0.004 |
| LIN7A | 0.005 | 0.395 | 0.019 |
| LINC00032 | <0.001 | 0.654 | 0.004 |
| LINC00111 | <0.001 | -1.042 | 0.005 |
| LINC00114 | <0.001 | -0.920 | 0.002 |
| LINC00161 | <0.001 | -1.016 | <0.001 |
| LINC00202-1 | 0.006 | -0.681 | 0.023 |
| LINC00235 | 0.005 | -0.275 | 0.020 |
| LINC00237 | 0.005 | 1.731 | 0.020 |
| LINC00242 | <0.001 | 0.591 | <0.001 |
| LINC00243 | 0.010 | -0.378 | 0.037 |
| LINC00271 | 0.001 | 0.358 | 0.007 |
| LINC00302 | <0.001 | -1.452 | <0.001 |
| LINC00310 | 0.001 | 0.396 | 0.007 |
| LINC00313 | <0.001 | 0.644 | 0.004 |
| LINC00315 | <0.001 | 0.428 | 0.004 |
| LINC00323 | 0.002 | 0.695 | 0.008 |
| LINC00327 | 0.002 | 0.698 | 0.010 |
| LINC00330 | 0.001 | -1.224 | 0.006 |
| LINC00398 | <0.001 | 0.722 | <0.001 |
| LINC00426 | 0.001 | 0.461 | 0.006 |
| LINC00460 | <0.001 | 0.815 | <0.001 |
| LINC00462 | <0.001 | -0.951 | <0.001 |
| LINC00466 | 0.004 | 0.791 | 0.017 |
| LINC00471 | 0.001 | 0.361 | 0.006 |
| LINC00472 | 0.007 | 0.673 | 0.026 |
| LINC00482 | 0.006 | -0.460 | 0.024 |
| LINC00494 | 0.007 | 0.936 | 0.025 |
| LINC00520 | 0.002 | -0.462 | 0.008 |
| LINC00524 | 0.008 | 0.819 | 0.029 |
| LINC00544 | 0.003 | 0.688 | 0.014 |
| LINC00556 | <0.001 | -1.634 | <0.001 |
| LINC00565 | 0.007 | 0.601 | 0.026 |
| LINC00574 | <0.001 | 0.794 | <0.001 |
| LINC00589 | 0.011 | 0.725 | 0.038 |
| LINC00592 | <0.001 | -0.874 | <0.001 |
| LINC00595 | 0.003 | 0.571 | 0.012 |
| LINC00598 | <0.001 | 0.414 | <0.001 |
| LINC00601 | <0.001 | 1.343 | <0.001 |
| LINC00607 | <0.001 | 1.022 | 0.001 |
| LINC00614 | 0.003 | -1.166 | 0.013 |
| LINC00622 | <0.001 | 0.719 | <0.001 |
| LINC00623 | <0.001 | 0.408 | <0.001 |
| LINC00628 | <0.001 | -0.933 | 0.001 |
| LINC00629 | 0.001 | 0.607 | 0.007 |
| LINC00632 | <0.001 | 0.877 | 0.001 |
| LINC00637 | 0.013 | -0.536 | 0.042 |
| LINC00640 | <0.001 | -0.467 | <0.001 |
| LINC00652 | <0.001 | 0.667 | 0.005 |
| LINC00654 | <0.001 | 0.689 | <0.001 |
| LINC00659 | 0.001 | -0.737 | 0.007 |
| LINC00663 | <0.001 | 0.341 | 0.002 |
| LINC00693 | 0.003 | 0.960 | 0.013 |
| LINC00702 | <0.001 | 0.907 | <0.001 |
| LINC00840 | 0.015 | 0.449 | 0.049 |
| LINC00861 | 0.009 | 0.496 | 0.033 |
| LINC00862 | 0.004 | 1.107 | 0.018 |
| LINC00869 | <0.001 | 0.405 | <0.001 |
| LINC00871 | <0.001 | -2.051 | <0.001 |
| LINC00877 | 0.004 | 0.534 | 0.016 |
| LINC00880 | 0.005 | 0.476 | 0.020 |
| LINC00881 | 0.003 | 0.807 | 0.012 |
| LINC00882 | <0.001 | 0.680 | <0.001 |
| LINC00888 | <0.001 | 0.429 | <0.001 |
| LINC00892 | 0.008 | 0.472 | 0.029 |
| LINC00926 | 0.002 | 0.480 | 0.008 |
| LINC00943 | <0.001 | 0.818 | <0.001 |
| LINC00944 | <0.001 | 0.495 | <0.001 |
| LINC00950 | <0.001 | 0.726 | 0.003 |
| LINC00954 | 0.008 | 0.542 | 0.028 |
| LINC00964 | 0.002 | -0.445 | 0.011 |
| LINC00973 | <0.001 | 1.593 | <0.001 |
| LINC00974 | <0.001 | -2.301 | 0.005 |
| LINC00996 | <0.001 | 0.552 | <0.001 |
| LINC01006 | 0.002 | 0.327 | 0.010 |
| LINC01010 | <0.001 | -0.769 | <0.001 |
| LINC01013 | <0.001 | 0.712 | 0.004 |
| LINC01048 | 0.002 | 1.135 | 0.009 |
| LINC01050 | <0.001 | 0.902 | <0.001 |
| LINC01058 | <0.001 | 0.542 | 0.005 |
| LINC01060 | 0.006 | 0.516 | 0.025 |
| LINC01088 | 0.005 | -0.895 | 0.019 |
| LINC01116 | <0.001 | 0.519 | <0.001 |
| LINC01117 | <0.001 | 0.507 | <0.001 |
| LINC01118 | <0.001 | 0.576 | 0.003 |
| LINC01119 | <0.001 | 0.681 | <0.001 |
| LINC01121 | <0.001 | 0.851 | 0.002 |
| LINC01122 | <0.001 | 1.283 | <0.001 |
| LINC01133 | <0.001 | -0.653 | <0.001 |
| LINC01136 | 0.010 | -0.395 | 0.035 |
| LINC01138 | <0.001 | 0.312 | <0.001 |
| LINC01142 | <0.001 | 0.773 | 0.005 |
| LINC01145 | <0.001 | 0.353 | <0.001 |
| LINC01146 | 0.003 | 0.416 | 0.015 |
| LINC01150 | <0.001 | 0.645 | <0.001 |
| LINC01151 | 0.002 | 1.039 | 0.010 |
| LINC01152 | <0.001 | 1.390 | <0.001 |
| LINC01169 | 0.003 | -1.049 | 0.013 |
| LINC01179 | <0.001 | 1.491 | <0.001 |
| LINC01181 | 0.003 | -0.821 | 0.013 |
| LINC01204 | 0.001 | 1.017 | 0.007 |
| LINC01214 | <0.001 | -1.349 | <0.001 |
| LINC01224 | 0.005 | -1.072 | 0.021 |
| LINC01235 | 0.010 | 0.308 | 0.036 |
| LINC01237 | 0.002 | 0.435 | 0.011 |
| LINC01251 | <0.001 | -1.465 | <0.001 |
| LINC01252 | <0.001 | 0.713 | 0.002 |
| LINC01265 | <0.001 | 0.980 | 0.001 |
| LINC01269 | <0.001 | -1.340 | <0.001 |
| LINC01277 | 0.001 | 0.428 | 0.007 |
| LINC01281 | 0.010 | 0.409 | 0.035 |
| LINC01301 | <0.001 | 0.750 | <0.001 |
| LINC01315 | 0.012 | 0.382 | 0.041 |
| LINC01317 | 0.005 | -1.316 | 0.019 |
| LINC01336 | 0.014 | -0.305 | 0.047 |
| LINC01341 | 0.008 | -0.648 | 0.029 |
| LINC01343 | <0.001 | -0.893 | <0.001 |
| LINC01344 | 0.004 | -0.865 | 0.018 |
| LINC01348 | <0.001 | -0.790 | <0.001 |
| LINC01352 | <0.001 | 0.681 | 0.002 |
| LINC01354 | <0.001 | -0.895 | 0.001 |
| LINC01356 | 0.002 | 0.539 | 0.008 |
| LINC01357 | 0.003 | 0.480 | 0.014 |
| LINC01358 | <0.001 | 0.709 | <0.001 |
| LINC01359 | 0.006 | 0.476 | 0.024 |
| LINC01361 | 0.013 | 0.481 | 0.043 |
| LINC01366 | <0.001 | 0.730 | <0.001 |
| LINC01395 | <0.001 | -1.175 | <0.001 |
| LINC01422 | <0.001 | 0.579 | 0.002 |
| LINC01429 | <0.001 | 0.928 | <0.001 |
| LINC01449 | <0.001 | 0.748 | 0.002 |
| LINC01479 | 0.002 | 0.800 | 0.010 |
| LINC01483 | <0.001 | 0.713 | 0.003 |
| LINC01527 | <0.001 | -1.366 | <0.001 |
| LINC01546 | 0.001 | 0.516 | 0.007 |
| LINC01550 | 0.006 | 0.391 | 0.024 |
| LINC01558 | 0.013 | 0.353 | 0.044 |
| LINC01559 | <0.001 | -0.730 | <0.001 |
| LINC01561 | <0.001 | 0.744 | 0.001 |
| LINC01564 | <0.001 | -0.618 | <0.001 |
| LINC01583 | 0.011 | 0.911 | 0.037 |
| LINC01587 | 0.010 | -0.555 | 0.035 |
| LINC01588 | <0.001 | -0.324 | 0.002 |
| LINC01600 | <0.001 | 0.780 | <0.001 |
| LINC01607 | <0.001 | 0.642 | 0.001 |
| LINC01614 | <0.001 | 0.648 | <0.001 |
| LINC01615 | <0.001 | 0.382 | <0.001 |
| LINC01630 | 0.002 | 0.799 | 0.011 |
| LINC01638 | <0.001 | 0.890 | 0.003 |
| LINC01649 | 0.012 | 0.557 | 0.041 |
| LINC01655 | <0.001 | 0.750 | <0.001 |
| LINC01679 | <0.001 | 0.401 | <0.001 |
| LINC01684 | <0.001 | 0.808 | <0.001 |
| LINC01686 | <0.001 | 0.467 | <0.001 |
| LINC01694 | <0.001 | -0.909 | 0.002 |
| LINC01695 | 0.014 | -1.089 | 0.047 |
| LINC01697 | <0.001 | -1.074 | 0.002 |
| LINC01702 | <0.001 | -0.922 | <0.001 |
| LINC01704 | <0.001 | 0.906 | <0.001 |
| LINC01711 | <0.001 | 0.885 | <0.001 |
| LINC01715 | <0.001 | 0.335 | 0.002 |
| LINC01730 | 0.008 | 0.372 | 0.029 |
| LINC01732 | <0.001 | -0.775 | 0.004 |
| LINC01747 | <0.001 | 1.105 | 0.001 |
| LINC01748 | 0.010 | -0.334 | 0.035 |
| LINC01752 | <0.001 | -0.620 | <0.001 |
| LINC01770 | 0.013 | -0.274 | 0.043 |
| LINC01772 | <0.001 | 0.349 | <0.001 |
| LINC01775 | <0.001 | 0.567 | <0.001 |
| LINC01783 | 0.010 | -0.876 | 0.035 |
| LINC01792 | <0.001 | -1.035 | <0.001 |
| LINC01802 | 0.015 | 0.476 | 0.050 |
| LINC01805 | <0.001 | -1.218 | <0.001 |
| LINC01828 | 0.010 | 1.014 | 0.035 |
| LINC01857 | 0.006 | 0.477 | 0.022 |
| LINC01886 | 0.007 | -1.007 | 0.026 |
| LINC01891 | 0.015 | 0.623 | 0.049 |
| LINC01894 | 0.001 | 1.422 | 0.006 |
| LINC01915 | <0.001 | 0.753 | 0.005 |
| LINC01929 | 0.002 | 0.608 | 0.009 |
| LINC01932 | <0.001 | -0.587 | <0.001 |
| LINC01933 | 0.002 | 0.810 | 0.008 |
| LINC01934 | 0.004 | 0.610 | 0.016 |
| LINC01936 | <0.001 | 0.954 | 0.003 |
| LINC01943 | <0.001 | 0.563 | <0.001 |
| LINC01948 | <0.001 | -0.461 | 0.002 |
| LINC01963 | <0.001 | 0.270 | <0.001 |
| LINC01964 | <0.001 | -2.120 | <0.001 |
| LINC01977 | 0.011 | 0.546 | 0.038 |
| LINC01983 | <0.001 | -1.518 | <0.001 |
| LINC01990 | <0.001 | 0.741 | 0.004 |
| LINC01994 | <0.001 | -1.397 | <0.001 |
| LINC01995 | <0.001 | -1.752 | <0.001 |
| LINC01998 | <0.001 | 0.919 | 0.002 |
| LINC02004 | 0.013 | -0.480 | 0.044 |
| LINC02005 | 0.009 | -0.575 | 0.033 |
| LINC02006 | 0.001 | -0.693 | 0.007 |
| LINC02012 | <0.001 | -0.876 | <0.001 |
| LINC02014 | <0.001 | -0.680 | <0.001 |
| LINC02031 | <0.001 | -1.051 | <0.001 |
| LINC02033 | 0.006 | 0.353 | 0.022 |
| LINC02038 | 0.004 | -1.052 | 0.016 |
| LINC02044 | <0.001 | 0.706 | 0.004 |
| LINC02080 | 0.007 | 0.401 | 0.027 |
| LINC02084 | 0.004 | 0.380 | 0.016 |
| LINC02099 | <0.001 | 0.472 | <0.001 |
| LINC02100 | <0.001 | 0.387 | 0.002 |
| LINC02104 | <0.001 | 0.978 | <0.001 |
| LINC02126 | 0.002 | 0.755 | 0.008 |
| LINC02132 | <0.001 | 0.800 | 0.005 |
| LINC02137 | <0.001 | -0.976 | <0.001 |
| LINC02158 | <0.001 | -1.355 | <0.001 |
| LINC02178 | 0.002 | -0.367 | 0.008 |
| LINC02185 | <0.001 | -0.902 | 0.002 |
| LINC02202 | <0.001 | 0.523 | <0.001 |
| LINC02210 | <0.001 | -0.263 | <0.001 |
| LINC02211 | 0.005 | 0.895 | 0.021 |
| LINC02234 | <0.001 | 0.877 | 0.003 |
| LINC02243 | <0.001 | -0.848 | <0.001 |
| LINC02244 | <0.001 | -1.080 | <0.001 |
| LINC02257 | <0.001 | 0.831 | <0.001 |
| LINC02265 | 0.005 | 0.437 | 0.021 |
| LINC02280 | 0.006 | -0.923 | 0.023 |
| LINC02285 | <0.001 | 0.497 | 0.002 |
| LINC02308 | 0.014 | -0.714 | 0.047 |
| LINC02310 | <0.001 | -1.159 | <0.001 |
| LINC02323 | <0.001 | -0.696 | <0.001 |
| LINC02328 | 0.004 | 0.323 | 0.018 |
| LINC02345 | 0.003 | 0.559 | 0.014 |
| LINC02381 | <0.001 | 0.528 | <0.001 |
| LINC02392 | 0.010 | 0.993 | 0.036 |
| LINC02397 | 0.003 | 0.730 | 0.012 |
| LINC02408 | <0.001 | 0.517 | 0.002 |
| LINC02416 | <0.001 | 0.413 | 0.001 |
| LINC02421 | 0.002 | 0.596 | 0.009 |
| LINC02428 | 0.010 | -0.394 | 0.035 |
| LINC02437 | <0.001 | -1.686 | <0.001 |
| LINC02448 | <0.001 | -1.346 | <0.001 |
| LINC02454 | 0.002 | 0.434 | 0.011 |
| LINC02458 | 0.006 | 0.731 | 0.024 |
| LINC02487 | <0.001 | -1.527 | <0.001 |
| LINC02528 | <0.001 | 0.803 | 0.002 |
| LINC02538 | <0.001 | -1.261 | 0.001 |
| LINC02544 | <0.001 | 0.488 | 0.005 |
| LINC02550 | 0.012 | 0.464 | 0.042 |
| LINC02551 | <0.001 | 0.781 | <0.001 |
| LINC02560 | <0.001 | -0.891 | <0.001 |
| LINC02568 | 0.008 | -0.668 | 0.030 |
| LINC02570 | <0.001 | -1.176 | <0.001 |
| LINC02577 | <0.001 | 0.542 | <0.001 |
| LINC02580 | <0.001 | -1.085 | <0.001 |
| LINC02587 | 0.015 | 0.617 | 0.049 |
| LINC02595 | <0.001 | -0.436 | <0.001 |
| LINC02598 | <0.001 | -0.758 | 0.005 |
| LINC02599 | <0.001 | 0.740 | 0.002 |
| LINC02603 | 0.012 | 0.385 | 0.040 |
| LINC02607 | 0.003 | 0.711 | 0.012 |
| LINC02608 | <0.001 | -1.068 | <0.001 |
| LINC02609 | <0.001 | 0.455 | <0.001 |
| LINC02611 | <0.001 | 0.524 | <0.001 |
| LINC02617 | <0.001 | -1.356 | <0.001 |
| LINC02621 | <0.001 | -1.514 | <0.001 |
| LINC02637 | 0.003 | 0.357 | 0.013 |
| LINC02649 | 0.012 | 0.354 | 0.040 |
| LINC02656 | <0.001 | 0.606 | <0.001 |
| LINC02701 | 0.010 | 0.509 | 0.035 |
| LINC02709 | 0.003 | -0.280 | 0.013 |
| LINC02725 | 0.002 | 1.650 | 0.009 |
| LINC02763 | 0.006 | -0.808 | 0.025 |
| LINC02765 | 0.005 | -0.727 | 0.020 |
| LINC02777 | <0.001 | 0.494 | <0.001 |
| LINC02785 | <0.001 | 0.627 | 0.003 |
| LINC02798 | <0.001 | 1.102 | <0.001 |
| LINC02800 | <0.001 | -0.857 | <0.001 |
| LINC02804 | 0.001 | 0.599 | 0.007 |
| LINC02816 | 0.003 | 0.489 | 0.015 |
| LINC02817 | 0.004 | 0.714 | 0.018 |
| LINC02820 | 0.007 | -0.418 | 0.026 |
| LINC02846 | <0.001 | -0.358 | 0.003 |
| LINC02847 | <0.001 | 0.790 | 0.003 |
| LINC02863 | <0.001 | 0.739 | <0.001 |
| LINGO1 | 0.002 | 0.357 | 0.008 |
| LINGO2 | 0.004 | 0.704 | 0.015 |
| LINGO3 | 0.003 | 0.593 | 0.012 |
| LIPE-AS1 | <0.001 | -0.380 | 0.003 |
| LIPH | <0.001 | -0.796 | <0.001 |
| LIPK | <0.001 | -0.902 | <0.001 |
| LIPM | <0.001 | -0.838 | <0.001 |
| LIPN | <0.001 | -1.843 | <0.001 |
| LIVAR | <0.001 | -0.450 | 0.002 |
| LIX1 | 0.014 | 1.596 | 0.046 |
| LIX1-AS1 | <0.001 | 0.554 | 0.006 |
| LIX1L | <0.001 | 0.352 | <0.001 |
| LIX1L-AS1 | <0.001 | 0.451 | <0.001 |
| LLGL2 | <0.001 | -0.323 | <0.001 |
| LLPH-DT | 0.004 | 0.335 | 0.016 |
| LMO7 | <0.001 | -0.299 | <0.001 |
| LMOD1 | <0.001 | 0.348 | 0.002 |
| LMOD3 | 0.006 | 0.654 | 0.024 |
| LMTK3 | <0.001 | -0.359 | <0.001 |
| LMX1B | <0.001 | 0.663 | <0.001 |
| LNCOC1 | <0.001 | 0.509 | 0.001 |
| LNCOG | <0.001 | 1.050 | <0.001 |
| LNCSRLR | <0.001 | 0.722 | <0.001 |
| LNX1 | <0.001 | -0.365 | <0.001 |
| LOR | <0.001 | -2.002 | <0.001 |
| LOXL2 | <0.001 | 0.328 | <0.001 |
| LOXL3 | <0.001 | 0.301 | <0.001 |
| LOXL4 | 0.002 | 0.386 | 0.008 |
| LPAL2 | <0.001 | 0.748 | <0.001 |
| LPAR4 | <0.001 | 0.457 | 0.002 |
| LRCH2 | <0.001 | 0.673 | <0.001 |
| LRG1 | <0.001 | -0.341 | <0.001 |
| LRGUK | 0.006 | 0.434 | 0.023 |
| LRIG2-DT | <0.001 | 0.439 | <0.001 |
| LRP4-AS1 | <0.001 | 0.444 | 0.005 |
| LRRC10B | 0.015 | 0.522 | 0.049 |
| LRRC14B | <0.001 | 0.848 | 0.005 |
| LRRC17 | <0.001 | 0.461 | <0.001 |
| LRRC2 | 0.004 | 0.754 | 0.017 |
| LRRC25 | <0.001 | 0.327 | <0.001 |
| LRRC27 | <0.001 | 0.339 | <0.001 |
| LRRC29 | <0.001 | 0.296 | <0.001 |
| LRRC36 | 0.001 | 0.487 | 0.006 |
| LRRC37A4P | 0.003 | -0.407 | 0.014 |
| LRRC37A5P | <0.001 | 0.567 | 0.002 |
| LRRC37A7P | 0.008 | 0.664 | 0.029 |
| LRRC39 | 0.002 | 0.520 | 0.008 |
| LRRC4 | <0.001 | -0.799 | <0.001 |
| LRRC4C | <0.001 | 0.914 | 0.001 |
| LRRC6 | <0.001 | 0.517 | <0.001 |
| LRRC63 | <0.001 | 0.715 | <0.001 |
| LRRC66 | <0.001 | 0.716 | <0.001 |
| LRRC75A | <0.001 | 0.517 | <0.001 |
| LRRC8B | <0.001 | -0.287 | <0.001 |
| LRRC8E | <0.001 | -0.478 | <0.001 |
| LRRN1 | 0.003 | 0.465 | 0.013 |
| LRRN2 | <0.001 | 0.736 | <0.001 |
| LSAMP | <0.001 | 0.509 | 0.004 |
| LSMEM2 | 0.015 | 0.541 | 0.048 |
| LST1 | <0.001 | 0.300 | <0.001 |
| LTA | <0.001 | 0.504 | <0.001 |
| LTB | <0.001 | 0.279 | <0.001 |
| LTB4R | <0.001 | -0.274 | <0.001 |
| LTB4R2 | <0.001 | -0.377 | <0.001 |
| LY6G6C | <0.001 | -0.970 | <0.001 |
| LY6G6E | 0.004 | -1.078 | 0.018 |
| LY6H | 0.002 | 0.897 | 0.012 |
| LY75-CD302 | 0.005 | 0.510 | 0.022 |
| LY86 | <0.001 | 0.392 | <0.001 |
| LYL1 | <0.001 | 0.307 | <0.001 |
| LYNX1 | <0.001 | -0.620 | <0.001 |
| LYPD2 | <0.001 | -1.223 | <0.001 |
| LYPD5 | <0.001 | -0.537 | <0.001 |
| LYRM9 | 0.001 | 0.313 | 0.007 |
| LZTS1 | <0.001 | 0.467 | <0.001 |
| MAB21L3 | <0.001 | -0.761 | <0.001 |
| MAB21L4 | <0.001 | -0.515 | <0.001 |
| MACC1 | <0.001 | -0.626 | <0.001 |
| MACORIS | 0.003 | 0.436 | 0.013 |
| MAFTRR | 0.006 | -0.367 | 0.022 |
| MAGEA11 | 0.014 | -0.549 | 0.047 |
| MAGEL2 | <0.001 | 0.704 | 0.003 |
| MAGI1-IT1 | 0.008 | -0.576 | 0.031 |
| MAGI2-AS3 | <0.001 | 0.489 | <0.001 |
| MAGIX | <0.001 | -0.294 | 0.005 |
| MAGOH2P | 0.004 | 0.339 | 0.019 |
| MAL | <0.001 | -0.702 | <0.001 |
| MAL2-AS1 | <0.001 | -0.494 | 0.004 |
| MALL | <0.001 | -0.529 | <0.001 |
| MAMDC2 | <0.001 | 0.526 | <0.001 |
| MAMDC2-AS1 | 0.002 | 0.490 | 0.010 |
| MAMDC4 | 0.015 | 0.291 | 0.050 |
| MAN1C1 | <0.001 | 0.346 | 0.001 |
| MANSC1 | <0.001 | -0.306 | <0.001 |
| MAP10 | 0.003 | 0.395 | 0.013 |
| MAP1A | <0.001 | 0.339 | 0.002 |
| MAP1B | <0.001 | 0.314 | <0.001 |
| MAP3K14-AS1 | <0.001 | 0.286 | 0.003 |
| MAP3K2-DT | <0.001 | 0.410 | <0.001 |
| MAP3K7CL | <0.001 | 0.342 | <0.001 |
| MAP3K8 | <0.001 | -0.297 | <0.001 |
| MAP3K9 | <0.001 | -0.391 | <0.001 |
| MAPK10 | <0.001 | -0.504 | <0.001 |
| MAPK13 | <0.001 | -0.269 | <0.001 |
| MAPK4 | <0.001 | 1.354 | <0.001 |
| MAPK6-DT | <0.001 | -0.594 | <0.001 |
| MARCHF1 | <0.001 | 0.308 | <0.001 |
| MARCHF10 | 0.007 | 0.506 | 0.028 |
| MARCHF3 | <0.001 | -0.330 | <0.001 |
| MARCHF4 | <0.001 | 1.337 | <0.001 |
| MASP1 | <0.001 | -0.675 | 0.002 |
| MAST4-AS1 | <0.001 | -0.587 | <0.001 |
| MATN1 | <0.001 | 0.622 | <0.001 |
| MATN1-AS1 | <0.001 | 0.417 | <0.001 |
| MATN3 | <0.001 | 0.624 | <0.001 |
| MBL1P | 0.002 | 0.569 | 0.009 |
| MCF2 | <0.001 | 1.474 | 0.001 |
| MCOLN2 | <0.001 | 0.450 | <0.001 |
| MCOLN3 | 0.003 | 0.401 | 0.012 |
| MCUR1P1 | 0.003 | -0.557 | 0.015 |
| MDS2 | 0.003 | 0.560 | 0.015 |
| ME1 | <0.001 | -0.352 | <0.001 |
| ME3 | <0.001 | 0.479 | <0.001 |
| MECOM | 0.001 | 0.317 | 0.007 |
| MECOM-AS1 | <0.001 | -0.693 | <0.001 |
| MEF2C | <0.001 | 0.427 | <0.001 |
| MEF2C-AS1 | <0.001 | 0.635 | 0.003 |
| MEFV | <0.001 | -0.506 | <0.001 |
| MEG3 | <0.001 | 0.604 | <0.001 |
| MEG8 | 0.002 | 0.968 | 0.008 |
| MEG9 | <0.001 | 0.994 | <0.001 |
| MEIS3 | <0.001 | 0.289 | 0.005 |
| MEIS3P1 | <0.001 | 0.375 | 0.003 |
| MEIS3P2 | 0.002 | 0.516 | 0.010 |
| MEOX2 | <0.001 | 0.444 | 0.005 |
| MEP1A | <0.001 | 0.726 | <0.001 |
| MESTP1 | 0.003 | 0.531 | 0.014 |
| MESTP3 | 0.010 | 0.569 | 0.035 |
| METTL11B | <0.001 | 0.710 | <0.001 |
| METTL7B | <0.001 | 0.618 | <0.001 |
| MEX3A | <0.001 | 0.330 | 0.003 |
| MEX3B | <0.001 | 0.286 | 0.004 |
| MFAP1P1 | <0.001 | 0.819 | 0.005 |
| MFF-DT | <0.001 | 0.304 | <0.001 |
| MFRP | <0.001 | 0.556 | 0.003 |
| MFSD2B | <0.001 | -0.374 | <0.001 |
| MFSD6L | 0.009 | -0.531 | 0.032 |
| MGAT4EP | <0.001 | -0.661 | 0.005 |
| MGAT5B | <0.001 | 0.845 | <0.001 |
| MIA | 0.012 | 0.683 | 0.041 |
| MICAL2 | <0.001 | 0.338 | <0.001 |
| MICB | <0.001 | 0.318 | <0.001 |
| MICE | <0.001 | 0.469 | <0.001 |
| MICOS10-NBL1 | 0.005 | 0.569 | 0.021 |
| MILR1 | <0.001 | 0.423 | <0.001 |
| MINAR1 | <0.001 | 0.537 | <0.001 |
| MIOX | <0.001 | -0.972 | 0.002 |
| MIR1-1HG-AS1 | 0.001 | 0.910 | 0.006 |
| MIR100HG | <0.001 | 0.581 | <0.001 |
| MIR155HG | 0.002 | 0.356 | 0.010 |
| MIR203A | <0.001 | -1.165 | <0.001 |
| MIR210 | 0.002 | -0.384 | 0.009 |
| MIR2117HG | 0.007 | -0.363 | 0.026 |
| MIR3150BHG | <0.001 | -0.791 | <0.001 |
| MIR3681HG | 0.002 | 1.248 | 0.009 |
| MIR421 | 0.014 | 0.422 | 0.046 |
| MIR4326 | 0.014 | 0.412 | 0.046 |
| MIR4435-2HG | <0.001 | 0.283 | <0.001 |
| MIR4453HG | <0.001 | -0.267 | 0.003 |
| MIR4482 | 0.006 | -0.318 | 0.025 |
| MIR4520-1 | <0.001 | -0.450 | 0.001 |
| MIR4645 | 0.007 | 0.400 | 0.026 |
| MIR4697HG | 0.002 | 0.578 | 0.010 |
| MIR497HG | <0.001 | 0.413 | <0.001 |
| MIR503HG | <0.001 | 0.677 | <0.001 |
| MIR5188 | <0.001 | -1.044 | <0.001 |
| MIR554 | <0.001 | -0.817 | <0.001 |
| MIR5689HG | 0.013 | -0.526 | 0.043 |
| MIR6772 | 0.015 | -0.339 | 0.050 |
| MIR6867 | <0.001 | -1.021 | <0.001 |
| MIR708 | 0.007 | -0.490 | 0.026 |
| MIR744 | 0.011 | -0.417 | 0.039 |
| MIR8058 | 0.007 | 0.497 | 0.026 |
| MIR936 | 0.009 | 0.495 | 0.032 |
| MISP3 | 0.002 | -0.286 | 0.010 |
| MITF | <0.001 | 0.414 | <0.001 |
| MKRN2OS | <0.001 | -0.362 | 0.005 |
| MKRN4P | <0.001 | -1.090 | 0.001 |
| MLIP-IT1 | <0.001 | -0.954 | <0.001 |
| MME | <0.001 | 0.442 | 0.001 |
| MMP13 | <0.001 | 0.397 | 0.001 |
| MMP16 | <0.001 | 0.683 | <0.001 |
| MMP19 | <0.001 | 0.288 | <0.001 |
| MMP24 | <0.001 | 0.570 | <0.001 |
| MMP28 | <0.001 | 0.464 | <0.001 |
| MMP7 | <0.001 | 0.305 | <0.001 |
| MNDA | <0.001 | 0.293 | <0.001 |
| MOGAT2 | 0.001 | 1.041 | 0.007 |
| MORN3 | <0.001 | -0.623 | 0.002 |
| MORN4 | <0.001 | 0.483 | <0.001 |
| MPDZ | <0.001 | 0.443 | <0.001 |
| MPIG6B | <0.001 | -0.962 | <0.001 |
| MPP1 | <0.001 | 0.331 | <0.001 |
| MPP2 | <0.001 | 0.498 | 0.002 |
| MPP4 | 0.009 | -0.662 | 0.032 |
| MPP7 | <0.001 | -0.311 | <0.001 |
| MPZL3 | <0.001 | -0.441 | <0.001 |
| MRAP | 0.007 | 0.650 | 0.027 |
| MRAP2 | 0.002 | -0.422 | 0.009 |
| MRAS | <0.001 | 0.542 | <0.001 |
| MREG | <0.001 | -0.302 | <0.001 |
| MRLN | <0.001 | -1.204 | <0.001 |
| MRO | <0.001 | 0.479 | 0.005 |
| MROH2A | 0.007 | -0.697 | 0.028 |
| MRPL40P1 | 0.012 | 0.556 | 0.041 |
| MRPS17P1 | 0.002 | 0.773 | 0.009 |
| MRPS30-DT | 0.007 | 0.376 | 0.028 |
| MRVI1-AS1 | <0.001 | -0.502 | 0.003 |
| MS4A14 | <0.001 | 0.439 | <0.001 |
| MS4A4A | <0.001 | 0.347 | <0.001 |
| MS4A4E | 0.001 | 0.355 | 0.007 |
| MS4A6A | <0.001 | 0.317 | <0.001 |
| MSANTD3-TMEFF1 | <0.001 | 0.553 | <0.001 |
| MSC | <0.001 | 0.353 | <0.001 |
| MSC-AS1 | <0.001 | 0.501 | <0.001 |
| MSGN1 | 0.001 | -1.090 | 0.006 |
| MSLN | 0.003 | -0.416 | 0.014 |
| MSMB | <0.001 | -0.556 | 0.004 |
| MSNP1 | <0.001 | 0.478 | <0.001 |
| MSR1 | <0.001 | 0.339 | 0.002 |
| MSRB3 | <0.001 | 0.444 | <0.001 |
| MSS51 | <0.001 | 0.313 | <0.001 |
| MSTN | 0.007 | 0.627 | 0.027 |
| MSX1 | <0.001 | 0.292 | 0.003 |
| MT1A | 0.004 | 0.489 | 0.019 |
| MT1DP | <0.001 | 0.508 | <0.001 |
| MT1F | <0.001 | 0.394 | <0.001 |
| MT1L | <0.001 | 0.352 | <0.001 |
| MT1M | 0.003 | 0.302 | 0.015 |
| MT2P1 | <0.001 | 0.413 | <0.001 |
| MTMR7 | 0.004 | 0.325 | 0.016 |
| MTND4P20 | <0.001 | -1.490 | <0.001 |
| MTTP | 0.003 | 0.668 | 0.014 |
| MUC12 | <0.001 | 0.670 | <0.001 |
| MUC15 | <0.001 | -0.972 | <0.001 |
| MUC20 | <0.001 | -0.653 | <0.001 |
| MUC20P1 | <0.001 | -0.615 | 0.002 |
| MUC21 | <0.001 | -1.189 | <0.001 |
| MUC22 | 0.002 | -1.643 | 0.010 |
| MUC4 | <0.001 | -0.614 | 0.004 |
| MUCL3 | 0.007 | -1.419 | 0.026 |
| MUSK | 0.002 | 0.668 | 0.010 |
| MXD1 | <0.001 | -0.284 | <0.001 |
| MXD3 | <0.001 | 0.305 | <0.001 |
| MXRA5 | <0.001 | 0.319 | <0.001 |
| MXRA5Y | 0.003 | 0.615 | 0.013 |
| MYBL1 | <0.001 | 0.566 | <0.001 |
| MYBPC1 | <0.001 | 0.759 | 0.002 |
| MYBPC2 | 0.002 | 0.592 | 0.010 |
| MYBPH | 0.008 | 0.487 | 0.031 |
| MYCL | <0.001 | -0.334 | <0.001 |
| MYF5 | 0.009 | 0.754 | 0.033 |
| MYF6 | 0.010 | 0.572 | 0.034 |
| MYH14 | <0.001 | -0.298 | <0.001 |
| MYH3 | 0.002 | 0.457 | 0.010 |
| MYH8 | 0.015 | 0.665 | 0.048 |
| MYL3 | 0.008 | 0.647 | 0.029 |
| MYL4 | 0.005 | 0.630 | 0.019 |
| MYLK2 | 0.001 | 0.695 | 0.006 |
| MYLK4 | 0.002 | -0.403 | 0.008 |
| MYMK | 0.015 | 0.628 | 0.048 |
| MYO1F | <0.001 | 0.279 | <0.001 |
| MYO1H | <0.001 | -1.497 | <0.001 |
| MYO5B | <0.001 | -0.359 | <0.001 |
| MYO5C | 0.006 | -0.302 | 0.022 |
| MYO7B | 0.014 | 0.564 | 0.047 |
| MYOCD | 0.011 | 0.714 | 0.037 |
| MYOG | 0.005 | 0.572 | 0.021 |
| MYOM1 | 0.011 | 0.516 | 0.037 |
| MYOM2 | 0.006 | 0.476 | 0.023 |
| MYOT | 0.007 | 0.546 | 0.026 |
| MYPN | 0.005 | 0.709 | 0.021 |
| MYZAP | <0.001 | -0.691 | <0.001 |
| N4BP2L1 | <0.001 | 0.301 | 0.002 |
| NAALADL1 | <0.001 | 0.317 | 0.002 |
| NADK2-AS1 | 0.003 | 0.452 | 0.014 |
| NAIP | <0.001 | 0.547 | 0.003 |
| NAIPP1 | <0.001 | 0.529 | 0.001 |
| NAIPP2 | <0.001 | 0.444 | 0.002 |
| NAIPP3 | <0.001 | 0.563 | 0.004 |
| NAIPP4 | 0.008 | 0.360 | 0.029 |
| NALCN | 0.001 | 0.954 | 0.008 |
| NANOS1 | 0.001 | 0.310 | 0.006 |
| NAP1L1P3 | 0.002 | 0.284 | 0.008 |
| NAP1L3 | <0.001 | 0.640 | <0.001 |
| NAT14 | <0.001 | 0.280 | <0.001 |
| NAV2 | <0.001 | 0.269 | <0.001 |
| NAV2-AS2 | 0.003 | 0.628 | 0.014 |
| NBPF13P | 0.004 | 0.717 | 0.016 |
| NBPF15 | <0.001 | 0.314 | <0.001 |
| NBPF20 | <0.001 | 0.272 | 0.001 |
| NBPF8 | <0.001 | 0.357 | <0.001 |
| NBPF9 | <0.001 | 0.404 | <0.001 |
| NCAM1 | <0.001 | 0.676 | 0.001 |
| NCAM2 | 0.010 | 0.718 | 0.035 |
| NCCRP1 | <0.001 | -0.618 | <0.001 |
| NCKAP1L | <0.001 | 0.332 | <0.001 |
| NCR3 | 0.005 | 0.430 | 0.019 |
| NDRG2 | <0.001 | -0.268 | <0.001 |
| NDRG4 | <0.001 | -0.499 | <0.001 |
| NDST3 | 0.011 | 0.803 | 0.039 |
| NEBL | <0.001 | -0.437 | <0.001 |
| NECAB1 | <0.001 | 0.775 | <0.001 |
| NECTIN1-AS1 | <0.001 | -0.783 | 0.002 |
| NECTIN3 | <0.001 | 0.465 | <0.001 |
| NEDD9 | <0.001 | 0.299 | <0.001 |
| NEK6 | <0.001 | 0.295 | <0.001 |
| NEU2 | <0.001 | -2.037 | <0.001 |
| NEUROD2 | 0.007 | -1.198 | 0.027 |
| NEXN | <0.001 | 0.415 | <0.001 |
| NFAM1 | <0.001 | 0.316 | 0.003 |
| NFATC2 | <0.001 | 0.307 | <0.001 |
| NFIA-AS2 | <0.001 | 1.012 | <0.001 |
| NGF | 0.007 | 0.339 | 0.026 |
| NHSL2 | <0.001 | 0.595 | <0.001 |
| NID2 | <0.001 | 0.355 | <0.001 |
| NIPAL1 | <0.001 | -0.340 | <0.001 |
| NIPSNAP3B | 0.014 | 0.302 | 0.046 |
| NKAIN1 | 0.002 | -0.446 | 0.008 |
| NKAPL | <0.001 | 0.759 | <0.001 |
| NKILA | <0.001 | 0.672 | <0.001 |
| NKPD1 | <0.001 | -0.847 | <0.001 |
| NKX3-2 | <0.001 | 0.711 | <0.001 |
| NKX6-1 | 0.004 | 0.483 | 0.016 |
| NLRC3 | 0.002 | 0.302 | 0.010 |
| NLRC4 | <0.001 | 0.327 | <0.001 |
| NLRP10 | <0.001 | -1.115 | <0.001 |
| NLRP3 | <0.001 | 0.463 | <0.001 |
| NLRX1 | <0.001 | -0.382 | <0.001 |
| NME7 | <0.001 | 0.296 | <0.001 |
| NME8 | <0.001 | 0.650 | 0.001 |
| NMNAT2 | 0.001 | 0.350 | 0.008 |
| NMRAL2P | <0.001 | -0.786 | <0.001 |
| NMU | <0.001 | -0.350 | <0.001 |
| NMUR1 | 0.002 | 0.849 | 0.009 |
| NNMT | <0.001 | 0.271 | <0.001 |
| NOD2 | <0.001 | -0.382 | <0.001 |
| NOG | <0.001 | 1.116 | <0.001 |
| NOL4L | <0.001 | 0.385 | <0.001 |
| NOS1 | 0.002 | -0.542 | 0.008 |
| NOS1AP | <0.001 | -0.596 | <0.001 |
| NOVA2 | <0.001 | 0.314 | <0.001 |
| NOX4 | <0.001 | 0.373 | <0.001 |
| NPAS1 | <0.001 | -0.420 | <0.001 |
| NPAS3 | <0.001 | 0.859 | <0.001 |
| NPBWR1 | 0.015 | 0.403 | 0.048 |
| NPEPPSP1 | <0.001 | -0.363 | 0.004 |
| NPHP1 | <0.001 | 0.369 | 0.001 |
| NPHP3-ACAD11 | 0.014 | 0.359 | 0.046 |
| NPHS1 | 0.006 | 1.211 | 0.022 |
| NPM1P18 | 0.008 | 0.416 | 0.030 |
| NPM1P19 | 0.002 | 0.391 | 0.011 |
| NPM1P22 | 0.001 | 0.547 | 0.006 |
| NPM1P24 | 0.015 | 0.341 | 0.048 |
| NPM1P34 | 0.013 | 0.398 | 0.045 |
| NPM1P38 | 0.008 | 0.490 | 0.028 |
| NPR2 | <0.001 | 0.344 | 0.003 |
| NPTX2 | 0.012 | 0.322 | 0.041 |
| NPTXR | 0.004 | 0.345 | 0.017 |
| NPY1R | 0.003 | 0.671 | 0.014 |
| NR2F2-AS1 | <0.001 | 0.721 | <0.001 |
| NR5A1 | <0.001 | -1.662 | <0.001 |
| NR5A2 | 0.009 | 0.368 | 0.031 |
| NRADDP | 0.004 | -0.384 | 0.016 |
| NREP | <0.001 | 0.296 | <0.001 |
| NRG2 | 0.005 | 0.641 | 0.019 |
| NRIP2 | <0.001 | 0.269 | 0.003 |
| NRIR | 0.006 | 0.438 | 0.024 |
| NRP1 | <0.001 | 0.275 | <0.001 |
| NRROS | <0.001 | 0.349 | <0.001 |
| NSG1 | <0.001 | -0.329 | <0.001 |
| NT5C1A | 0.003 | 1.189 | 0.012 |
| NT5C1B | <0.001 | -0.552 | <0.001 |
| NT5E | <0.001 | 0.356 | <0.001 |
| NT5M | <0.001 | 0.480 | <0.001 |
| NTM | 0.001 | 0.282 | 0.007 |
| NTN4 | <0.001 | 0.371 | <0.001 |
| NTNG1 | <0.001 | 1.824 | 0.001 |
| NTNG2 | <0.001 | 0.480 | <0.001 |
| NTRK1 | 0.002 | 0.545 | 0.009 |
| NTS | <0.001 | -1.000 | <0.001 |
| NUAK1 | <0.001 | 0.429 | <0.001 |
| NUDT6 | 0.001 | 0.274 | 0.006 |
| NUP210P3 | 0.015 | 0.529 | 0.049 |
| NUTM1 | 0.004 | -0.945 | 0.018 |
| NUTM2B | 0.004 | 0.371 | 0.017 |
| NUTM2D | 0.001 | 0.319 | 0.007 |
| NWD2 | <0.001 | -0.916 | 0.001 |
| NXPH2 | <0.001 | 1.437 | 0.004 |
| NXPH3 | <0.001 | 0.705 | <0.001 |
| NYAP1 | 0.003 | -0.566 | 0.014 |
| OACYLP | 0.005 | 0.407 | 0.020 |
| OAZ3 | <0.001 | 0.322 | <0.001 |
| OBP2A | <0.001 | 0.978 | <0.001 |
| OCLN | <0.001 | -0.632 | <0.001 |
| ODCP | 0.003 | -0.295 | 0.012 |
| ODF3L1 | <0.001 | 0.406 | <0.001 |
| ODF4 | <0.001 | -1.410 | 0.001 |
| OGN | <0.001 | 0.645 | 0.005 |
| OIT3 | 0.010 | 0.425 | 0.034 |
| OLAH | 0.001 | -0.877 | 0.006 |
| OLFML1 | <0.001 | 0.346 | <0.001 |
| OLFML2B | <0.001 | 0.283 | <0.001 |
| OLFML3 | <0.001 | 0.325 | <0.001 |
| OLR1 | 0.001 | 0.348 | 0.008 |
| OMP | <0.001 | -0.965 | <0.001 |
| ONECUT1 | 0.015 | -0.767 | 0.048 |
| ONECUT3 | 0.010 | -0.925 | 0.035 |
| OPRK1 | 0.004 | 1.769 | 0.016 |
| OPRL1 | <0.001 | 0.409 | <0.001 |
| OR2A1-AS1 | 0.007 | -0.299 | 0.027 |
| OR2I1P | <0.001 | 0.411 | 0.001 |
| OR51E1 | <0.001 | 0.349 | 0.005 |
| OR52K3P | 0.011 | 0.406 | 0.039 |
| OR52N4 | 0.004 | 0.672 | 0.017 |
| OR5BA1P | 0.015 | -0.343 | 0.049 |
| OR7A5 | 0.006 | -0.958 | 0.025 |
| OR7C1 | 0.015 | -0.905 | 0.048 |
| OR7E102P | 0.009 | 0.684 | 0.032 |
| OR7E108P | <0.001 | -1.134 | <0.001 |
| OR7E14P | <0.001 | -0.517 | <0.001 |
| OR7E62P | <0.001 | -0.680 | <0.001 |
| OR7E91P | <0.001 | -0.831 | <0.001 |
| OR7M1P | 0.008 | -0.514 | 0.030 |
| ORAI2 | <0.001 | 0.307 | <0.001 |
| OSER1-DT | <0.001 | 0.271 | 0.001 |
| OSGIN1 | <0.001 | -0.342 | 0.001 |
| OTOA | 0.002 | 0.532 | 0.011 |
| OTOAP1 | 0.002 | 0.625 | 0.009 |
| OTOP2 | <0.001 | -1.364 | <0.001 |
| OTOP3 | <0.001 | -1.688 | <0.001 |
| OTUB2 | <0.001 | -0.270 | 0.002 |
| OTX1 | <0.001 | -0.431 | <0.001 |
| OVOL1 | <0.001 | -0.552 | <0.001 |
| OVOL1-AS1 | <0.001 | -1.051 | <0.001 |
| OXCT1-AS1 | 0.004 | 0.390 | 0.016 |
| OXER1 | 0.002 | 0.449 | 0.008 |
| OXTR | <0.001 | 0.713 | <0.001 |
| P2RX5 | <0.001 | 0.557 | <0.001 |
| P2RX6 | <0.001 | 0.734 | <0.001 |
| P2RY1 | <0.001 | -0.458 | <0.001 |
| P2RY10 | 0.001 | 0.397 | 0.006 |
| P2RY12 | <0.001 | 0.687 | <0.001 |
| P2RY13 | <0.001 | 0.394 | 0.003 |
| P2RY14 | 0.010 | 0.298 | 0.036 |
| P2RY8 | 0.008 | 0.272 | 0.030 |
| P3H1 | <0.001 | 0.306 | <0.001 |
| P4HA2-AS1 | <0.001 | 1.063 | <0.001 |
| P4HA3 | <0.001 | 0.567 | <0.001 |
| PABPC4L | 0.008 | 0.395 | 0.029 |
| PABPC5 | <0.001 | 0.728 | <0.001 |
| PACSIN1 | <0.001 | 0.587 | 0.003 |
| PADI1 | 0.001 | -0.483 | 0.008 |
| PADI2 | <0.001 | 0.518 | <0.001 |
| PADI3 | 0.001 | -0.363 | 0.007 |
| PAG1 | <0.001 | 0.324 | <0.001 |
| PAK3 | <0.001 | 1.148 | 0.002 |
| PAK5 | 0.006 | -1.044 | 0.023 |
| PALM2 | <0.001 | 0.642 | 0.001 |
| PALM2AKAP2 | <0.001 | 0.753 | <0.001 |
| PANK1 | <0.001 | -0.270 | 0.001 |
| PAPPA | 0.001 | 0.332 | 0.006 |
| PAPSS2 | <0.001 | 0.355 | <0.001 |
| PAQR5 | 0.002 | -0.276 | 0.010 |
| PAQR8 | <0.001 | 0.319 | <0.001 |
| PARD3-AS1 | <0.001 | -0.527 | 0.001 |
| PARD3B | <0.001 | 0.567 | <0.001 |
| PARD6B | <0.001 | -0.316 | <0.001 |
| PARM1 | 0.003 | 0.297 | 0.014 |
| PARP15 | 0.005 | 0.370 | 0.021 |
| PARP1P1 | 0.008 | 0.484 | 0.031 |
| PARP4P2 | 0.009 | 0.351 | 0.033 |
| PARP8 | <0.001 | 0.266 | <0.001 |
| PARVG | <0.001 | 0.362 | <0.001 |
| PATL2 | 0.010 | 0.296 | 0.035 |
| PAX5 | 0.003 | 0.468 | 0.013 |
| PAX9 | <0.001 | -0.333 | <0.001 |
| PAXIP1-AS1 | <0.001 | -0.310 | <0.001 |
| PCARE | <0.001 | -0.995 | <0.001 |
| PCAT1 | <0.001 | -0.527 | <0.001 |
| PCAT7 | 0.014 | 0.295 | 0.045 |
| PCBP3 | <0.001 | 0.603 | <0.001 |
| PCDH11X | 0.015 | 1.563 | 0.049 |
| PCDH12 | <0.001 | 0.314 | <0.001 |
| PCDH18 | <0.001 | 0.408 | <0.001 |
| PCDH8 | 0.009 | 1.941 | 0.032 |
| PCDHAC1 | <0.001 | 1.260 | <0.001 |
| PCDHAC2 | <0.001 | 0.933 | 0.002 |
| PCDHGA1 | <0.001 | -0.515 | 0.002 |
| PCDHGA12 | <0.001 | 0.495 | 0.003 |
| PCED1B | <0.001 | 0.329 | <0.001 |
| PCED1B-AS1 | 0.003 | 0.283 | 0.012 |
| PCOLCE-AS1 | <0.001 | 0.745 | 0.005 |
| PCOLCE2 | <0.001 | 0.554 | 0.003 |
| PCP4L1 | <0.001 | -0.600 | <0.001 |
| PCSK6 | <0.001 | -0.373 | <0.001 |
| PCSK9 | <0.001 | -0.307 | <0.001 |
| PDCD1 | <0.001 | 0.384 | 0.002 |
| PDCD1LG2 | <0.001 | 0.400 | <0.001 |
| PDE10A | <0.001 | 0.451 | 0.004 |
| PDE1A | <0.001 | 0.501 | <0.001 |
| PDE1B | <0.001 | 0.486 | <0.001 |
| PDE1C | <0.001 | 0.685 | 0.003 |
| PDE2A | <0.001 | 0.356 | <0.001 |
| PDE3A | <0.001 | 0.384 | <0.001 |
| PDE4B | <0.001 | 0.420 | <0.001 |
| PDE4C | <0.001 | -0.757 | 0.005 |
| PDE4DIP | <0.001 | 0.445 | <0.001 |
| PDE7A | <0.001 | 0.346 | <0.001 |
| PDE8B | <0.001 | 0.280 | <0.001 |
| PDGFC | <0.001 | 0.410 | <0.001 |
| PDGFD | 0.006 | 0.307 | 0.023 |
| PDGFRL | 0.001 | 0.297 | 0.007 |
| PDIA2 | 0.003 | -0.898 | 0.015 |
| PDLIM3 | <0.001 | 0.494 | <0.001 |
| PEAK3 | 0.011 | 0.287 | 0.038 |
| PEAR1 | <0.001 | 0.269 | 0.002 |
| PEBP4 | 0.011 | 0.785 | 0.039 |
| PEF1-AS1 | 0.010 | 0.344 | 0.035 |
| PERM1 | <0.001 | -0.346 | <0.001 |
| PES1P2 | 0.013 | 1.541 | 0.044 |
| PFN1P1 | <0.001 | 0.278 | 0.003 |
| PFN1P2 | <0.001 | 0.488 | <0.001 |
| PFN1P6 | <0.001 | 0.610 | <0.001 |
| PGA5 | 0.011 | 0.807 | 0.038 |
| PGAM1P5 | 0.008 | 0.341 | 0.029 |
| PGAP4 | <0.001 | -0.349 | <0.001 |
| PGBD5 | 0.002 | -0.279 | 0.010 |
| PGK1P2 | 0.007 | 0.294 | 0.027 |
| PGLYRP2 | <0.001 | -1.407 | <0.001 |
| PGLYRP3 | <0.001 | -0.611 | <0.001 |
| PGLYRP4 | <0.001 | -0.548 | <0.001 |
| PGM2L1 | <0.001 | 0.393 | <0.001 |
| PGM5 | 0.005 | 0.414 | 0.019 |
| PHACTR1 | 0.002 | 0.284 | 0.008 |
| PHBP3 | 0.003 | 0.423 | 0.014 |
| PHC1 | <0.001 | 0.372 | <0.001 |
| PHC1P1 | <0.001 | 0.554 | <0.001 |
| PHEX | 0.006 | -0.313 | 0.024 |
| PHF21B | 0.004 | 0.897 | 0.017 |
| PHKG1 | 0.002 | 0.482 | 0.011 |
| PHLDB1 | <0.001 | 0.496 | <0.001 |
| PHLDB3 | <0.001 | -0.373 | <0.001 |
| PHYHD1 | <0.001 | -0.589 | <0.001 |
| PHYHIP | <0.001 | -0.596 | 0.003 |
| PI16 | 0.004 | 0.599 | 0.016 |
| PI3 | <0.001 | -0.424 | <0.001 |
| PICART1 | <0.001 | 0.550 | <0.001 |
| PIEZO2 | <0.001 | 0.538 | 0.001 |
| PIGFP1 | 0.015 | -0.328 | 0.048 |
| PIK3CD-AS1 | 0.006 | 1.110 | 0.024 |
| PIK3CG | 0.005 | 0.342 | 0.021 |
| PIK3R5 | <0.001 | 0.287 | 0.002 |
| PIK3R6 | <0.001 | 0.453 | <0.001 |
| PINLYP | 0.001 | -0.346 | 0.006 |
| PIP4P2 | <0.001 | 0.428 | <0.001 |
| PITX2 | <0.001 | -0.381 | 0.003 |
| PITX3 | <0.001 | 0.458 | 0.004 |
| PIWIL2 | 0.012 | -0.611 | 0.041 |
| PIWIL4 | 0.001 | 0.431 | 0.007 |
| PKDCC | <0.001 | 0.640 | <0.001 |
| PKIA | <0.001 | 0.394 | 0.002 |
| PKIG | <0.001 | 0.288 | <0.001 |
| PKMP3 | <0.001 | 0.510 | <0.001 |
| PKN2-AS1 | <0.001 | 0.551 | 0.004 |
| PKP4-AS1 | <0.001 | -0.814 | <0.001 |
| PLA1A | <0.001 | 0.457 | 0.002 |
| PLA2G2C | 0.007 | 0.680 | 0.026 |
| PLA2G2F | 0.001 | -0.593 | 0.006 |
| PLA2G3 | <0.001 | -1.052 | <0.001 |
| PLA2G4A | <0.001 | -0.363 | <0.001 |
| PLA2G4B | <0.001 | -0.973 | <0.001 |
| PLA2G4D | <0.001 | -1.463 | <0.001 |
| PLA2G4E | <0.001 | -0.862 | <0.001 |
| PLA2G4E-AS1 | <0.001 | -1.464 | <0.001 |
| PLA2G4F | <0.001 | -0.785 | <0.001 |
| PLAAT2 | 0.014 | -0.335 | 0.045 |
| PLAC9 | 0.001 | 0.281 | 0.007 |
| PLAC9P1 | 0.004 | 0.855 | 0.016 |
| PLAG1 | 0.009 | -0.305 | 0.034 |
| PLB1 | <0.001 | -0.322 | 0.005 |
| PLBD1 | <0.001 | -0.354 | <0.001 |
| PLBD1-AS1 | <0.001 | -0.601 | <0.001 |
| PLCB4 | 0.006 | 0.459 | 0.025 |
| PLCD1 | <0.001 | -0.319 | <0.001 |
| PLCE1-AS1 | <0.001 | 1.045 | 0.003 |
| PLCL1 | <0.001 | 0.564 | <0.001 |
| PLCL2 | <0.001 | 0.441 | <0.001 |
| PLCXD1 | <0.001 | -0.293 | <0.001 |
| PLCXD2 | 0.002 | -0.492 | 0.009 |
| PLCXD3 | <0.001 | 1.094 | <0.001 |
| PLD4 | <0.001 | 0.468 | 0.001 |
| PLEKHA3P1 | <0.001 | 0.467 | 0.003 |
| PLEKHA7 | <0.001 | -0.373 | <0.001 |
| PLEKHA8P1 | <0.001 | 0.321 | <0.001 |
| PLEKHG4B | 0.004 | 0.338 | 0.018 |
| PLEKHM1P1 | <0.001 | -0.272 | <0.001 |
| PLEKHN1 | <0.001 | -0.298 | <0.001 |
| PLEKHO1 | <0.001 | 0.277 | <0.001 |
| PLGLB2 | 0.012 | 0.437 | 0.042 |
| PLIN5 | 0.008 | -0.436 | 0.028 |
| PLLP | 0.002 | -0.266 | 0.011 |
| PLN | <0.001 | 0.635 | 0.002 |
| PLOD3 | <0.001 | 0.295 | <0.001 |
| PLPP4 | <0.001 | 0.507 | <0.001 |
| PLPP7 | 0.003 | 0.336 | 0.013 |
| PLS1 | <0.001 | -0.285 | 0.003 |
| PLXDC1 | <0.001 | 0.302 | <0.001 |
| PLXNC1 | <0.001 | 0.345 | <0.001 |
| PMEL | <0.001 | -0.345 | 0.005 |
| PMEPA1 | <0.001 | 0.288 | <0.001 |
| PNMA2 | <0.001 | 0.672 | 0.001 |
| PNMA8A | <0.001 | 0.650 | 0.001 |
| PNPLA1 | <0.001 | -1.537 | <0.001 |
| PNPLA3 | 0.005 | -0.345 | 0.021 |
| PODN | 0.003 | 0.303 | 0.014 |
| POF1B | <0.001 | -0.671 | <0.001 |
| POLN | 0.002 | 0.415 | 0.011 |
| POLR2F | 0.013 | 0.929 | 0.045 |
| POLR2J3 | <0.001 | -0.708 | <0.001 |
| POM121L9P | <0.001 | 0.683 | <0.001 |
| POPDC2 | <0.001 | 0.432 | <0.001 |
| POPDC3 | <0.001 | 0.372 | 0.001 |
| POSTN | <0.001 | 0.264 | <0.001 |
| POU2F2 | <0.001 | 0.442 | <0.001 |
| POU2F3 | <0.001 | -0.730 | <0.001 |
| POU3F2 | 0.007 | 1.460 | 0.026 |
| POU4F3 | <0.001 | 0.510 | 0.003 |
| POU5F1P3 | 0.012 | 0.343 | 0.040 |
| POU6F1 | <0.001 | 0.393 | <0.001 |
| PPARGC1A | 0.003 | 0.538 | 0.012 |
| PPDPFL | 0.009 | 1.345 | 0.033 |
| PPEF1 | <0.001 | 0.789 | <0.001 |
| PPFIA2 | 0.010 | 0.439 | 0.037 |
| PPFIA4 | <0.001 | 0.480 | <0.001 |
| PPFIBP2 | <0.001 | -0.277 | <0.001 |
| PPIAP28 | <0.001 | -1.027 | <0.001 |
| PPIAP40 | 0.003 | -0.289 | 0.012 |
| PPIAP45 | <0.001 | 0.364 | <0.001 |
| PPIAP59 | 0.003 | -0.624 | 0.013 |
| PPIEL | <0.001 | 0.496 | <0.001 |
| PPIL6 | <0.001 | 0.463 | 0.001 |
| PPL | <0.001 | -0.377 | <0.001 |
| PPM1E | 0.006 | 0.853 | 0.023 |
| PPM1K | <0.001 | 0.309 | <0.001 |
| PPM1K-DT | <0.001 | -0.809 | <0.001 |
| PPP1R14D | 0.005 | -0.666 | 0.020 |
| PPP1R1C | 0.002 | -0.446 | 0.008 |
| PPP1R2P1 | 0.011 | 0.529 | 0.037 |
| PPP1R32 | <0.001 | 0.329 | 0.004 |
| PPP1R36 | <0.001 | 0.485 | <0.001 |
| PPP1R3A | 0.004 | 1.099 | 0.016 |
| PPP1R3D | <0.001 | -0.292 | <0.001 |
| PPP1R3F | <0.001 | 0.294 | <0.001 |
| PPP1R3G | 0.003 | -0.332 | 0.015 |
| PPP2R2C | <0.001 | -0.281 | <0.001 |
| PPP4R4 | <0.001 | 0.514 | <0.001 |
| PPP5D1 | <0.001 | 0.399 | 0.003 |
| PRAP1 | <0.001 | -0.859 | <0.001 |
| PRDM16-DT | 0.004 | 0.765 | 0.019 |
| PRDM6 | 0.002 | 0.433 | 0.010 |
| PRDM8 | <0.001 | 0.692 | <0.001 |
| PRDX2P4 | <0.001 | 0.629 | 0.002 |
| PRELID2P1 | 0.002 | -0.595 | 0.011 |
| PRELP | 0.008 | 0.286 | 0.031 |
| PRG4 | 0.006 | 0.735 | 0.023 |
| PRICKLE2-DT | <0.001 | 0.945 | 0.004 |
| PRICKLE4 | 0.002 | -0.472 | 0.008 |
| PRKAA2 | 0.003 | 0.512 | 0.012 |
| PRKACB | <0.001 | 0.284 | <0.001 |
| PRKAG2-AS1 | 0.003 | 0.424 | 0.015 |
| PRKAG3 | 0.001 | 0.769 | 0.007 |
| PRKAR2B | 0.004 | 0.301 | 0.016 |
| PRKCA | <0.001 | 0.349 | <0.001 |
| PRKCB | <0.001 | 0.425 | <0.001 |
| PRKCG | 0.005 | 1.070 | 0.020 |
| PRKD1 | <0.001 | 0.401 | 0.002 |
| PRKN | <0.001 | 0.532 | 0.002 |
| PRLR | <0.001 | 0.875 | <0.001 |
| PROC | <0.001 | 0.627 | <0.001 |
| PRODH | 0.008 | -0.289 | 0.029 |
| PROS2P | <0.001 | -0.756 | 0.005 |
| PROX1 | 0.001 | 0.503 | 0.007 |
| PRR16 | <0.001 | 0.599 | <0.001 |
| PRR26 | 0.009 | 1.132 | 0.031 |
| PRR32 | 0.003 | 0.940 | 0.013 |
| PRR5-ARHGAP8 | 0.014 | -0.438 | 0.047 |
| PRR9 | <0.001 | -1.094 | <0.001 |
| PRRT2 | 0.004 | 0.393 | 0.018 |
| PRRX1 | <0.001 | 0.297 | <0.001 |
| PRSS1 | 0.014 | 1.574 | 0.046 |
| PRSS2 | <0.001 | 1.434 | 0.005 |
| PRSS22 | <0.001 | -0.362 | <0.001 |
| PRSS23 | <0.001 | 0.292 | <0.001 |
| PRSS27 | <0.001 | -1.095 | <0.001 |
| PRSS3 | <0.001 | -1.052 | <0.001 |
| PRSS51 | 0.002 | 1.067 | 0.010 |
| PRSS56 | 0.009 | 1.635 | 0.033 |
| PRTG | 0.003 | 0.504 | 0.013 |
| PRUNE2 | <0.001 | 0.773 | <0.001 |
| PRX | <0.001 | 0.278 | <0.001 |
| PSAPL1 | <0.001 | -0.723 | 0.005 |
| PSCA | <0.001 | -0.665 | <0.001 |
| PSD | 0.001 | 0.300 | 0.007 |
| PSD2 | 0.006 | 0.672 | 0.024 |
| PSG5 | 0.002 | 1.430 | 0.009 |
| PSMD10P1 | 0.003 | 0.405 | 0.015 |
| PSMG3-AS1 | <0.001 | 0.452 | <0.001 |
| PSORS1C2 | <0.001 | -1.222 | <0.001 |
| PSORS1C3 | 0.012 | 0.490 | 0.040 |
| PSPC1P1 | <0.001 | -0.574 | <0.001 |
| PTCHD4 | <0.001 | 0.691 | 0.004 |
| PTCRA | 0.004 | 0.463 | 0.018 |
| PTCSC2 | <0.001 | -0.718 | <0.001 |
| PTGDR | <0.001 | 0.535 | <0.001 |
| PTGER1 | 0.003 | -0.698 | 0.014 |
| PTGER2 | <0.001 | 0.545 | <0.001 |
| PTGER4 | <0.001 | 0.339 | <0.001 |
| PTGIR | <0.001 | 0.400 | <0.001 |
| PTGIS | 0.011 | 0.403 | 0.039 |
| PTH1R | <0.001 | 0.720 | <0.001 |
| PTH2R | 0.012 | -0.534 | 0.042 |
| PTK6 | <0.001 | -0.302 | <0.001 |
| PTP4A2P2 | <0.001 | 0.387 | 0.001 |
| PTPRG-AS1 | 0.014 | 0.464 | 0.046 |
| PTPRH | <0.001 | -0.394 | 0.002 |
| PTPRM | <0.001 | 0.334 | <0.001 |
| PTPRN | 0.001 | 0.774 | 0.006 |
| PTPRO | <0.001 | 0.752 | <0.001 |
| PTPRR | <0.001 | 1.072 | <0.001 |
| PTPRZ1 | <0.001 | -0.264 | <0.001 |
| PTRH1 | 0.007 | 0.361 | 0.026 |
| PTTG2 | 0.011 | 0.446 | 0.037 |
| PTX3 | <0.001 | 0.774 | <0.001 |
| PTX4 | <0.001 | -1.578 | <0.001 |
| PUDPP2 | <0.001 | -1.161 | <0.001 |
| PXDN | <0.001 | 0.266 | <0.001 |
| PXT1 | 0.001 | 0.471 | 0.006 |
| PYHIN1 | 0.003 | 0.380 | 0.014 |
| PYROXD2 | 0.002 | 0.277 | 0.008 |
| PYY2 | 0.013 | -0.269 | 0.044 |
| QRFPR | 0.014 | -0.626 | 0.046 |
| RAB11FIP1 | <0.001 | -0.464 | <0.001 |
| RAB25 | <0.001 | -0.264 | <0.001 |
| RAB33A | <0.001 | 0.484 | <0.001 |
| RAB39A | <0.001 | 0.571 | 0.002 |
| RAB3B | 0.003 | -0.660 | 0.013 |
| RAB3IL1 | <0.001 | 0.372 | <0.001 |
| RAB42 | <0.001 | 0.507 | <0.001 |
| RAB44 | <0.001 | -1.343 | <0.001 |
| RAB9B | 0.007 | 0.470 | 0.028 |
| RAET1E | <0.001 | -0.811 | <0.001 |
| RAET1E-AS1 | <0.001 | -0.832 | <0.001 |
| RAET1K | 0.004 | 0.448 | 0.018 |
| RAET1L | <0.001 | -0.499 | <0.001 |
| RAI14 | <0.001 | 0.317 | <0.001 |
| RAMP1 | <0.001 | 0.449 | <0.001 |
| RANP4 | 0.006 | 0.366 | 0.023 |
| RAP1AP | 0.007 | 0.411 | 0.026 |
| RAP2C-AS1 | <0.001 | 0.327 | <0.001 |
| RAP2CP1 | 0.015 | -0.802 | 0.049 |
| RAPGEFL1 | <0.001 | -0.409 | <0.001 |
| RARB | <0.001 | 0.383 | 0.005 |
| RASA4CP | <0.001 | -0.467 | <0.001 |
| RASAL1 | <0.001 | -0.601 | <0.001 |
| RASD2 | <0.001 | 0.745 | <0.001 |
| RASGRF1 | <0.001 | 1.041 | <0.001 |
| RASGRF2 | <0.001 | 0.428 | <0.001 |
| RASGRF2-AS1 | 0.001 | 0.625 | 0.006 |
| RASGRP1 | <0.001 | -0.413 | <0.001 |
| RASGRP2 | 0.001 | 0.352 | 0.008 |
| RASGRP3 | <0.001 | 0.351 | <0.001 |
| RASGRP4 | 0.003 | 0.274 | 0.012 |
| RASL10B | <0.001 | 0.563 | 0.001 |
| RASL11A | 0.002 | -0.298 | 0.010 |
| RASL11B | <0.001 | 0.330 | 0.004 |
| RASL12 | <0.001 | 0.350 | <0.001 |
| RASSF4 | <0.001 | 0.382 | <0.001 |
| RASSF6 | 0.001 | 0.304 | 0.006 |
| RASSF8 | <0.001 | 0.345 | <0.001 |
| RASSF8-AS1 | <0.001 | 0.630 | <0.001 |
| RASSF9 | 0.012 | -0.271 | 0.040 |
| RBBP8NL | <0.001 | -0.409 | <0.001 |
| RBFADN | 0.001 | 0.521 | 0.006 |
| RBFOX1 | 0.005 | 0.866 | 0.020 |
| RBM15-AS1 | <0.001 | 0.334 | <0.001 |
| RBM22P2 | <0.001 | 0.968 | <0.001 |
| RBM24 | 0.003 | 0.557 | 0.013 |
| RBMS3 | <0.001 | 0.545 | <0.001 |
| RBMS3-AS3 | <0.001 | 0.740 | <0.001 |
| RBP3 | <0.001 | 1.393 | <0.001 |
| RBPMS-AS1 | <0.001 | -0.648 | <0.001 |
| RBPMS2 | <0.001 | 0.452 | <0.001 |
| RCAN2 | <0.001 | 0.354 | <0.001 |
| RCSD1 | <0.001 | 0.310 | <0.001 |
| RCVRN | 0.002 | 0.610 | 0.011 |
| RDH12 | <0.001 | -1.330 | <0.001 |
| RDH13 | <0.001 | -0.274 | <0.001 |
| RDH5 | <0.001 | 0.439 | 0.002 |
| RDM1P5 | 0.001 | 0.454 | 0.007 |
| RECK | <0.001 | 0.406 | <0.001 |
| REEP1 | <0.001 | -0.638 | <0.001 |
| REEP2 | 0.005 | 0.394 | 0.020 |
| RELCH | <0.001 | -0.271 | <0.001 |
| REM1 | <0.001 | 0.642 | <0.001 |
| REN | <0.001 | -1.187 | <0.001 |
| REP15 | <0.001 | 0.296 | 0.005 |
| REPS2 | 0.001 | 0.300 | 0.006 |
| RERGL | 0.006 | 0.799 | 0.022 |
| RET | <0.001 | 0.579 | <0.001 |
| RETN | <0.001 | 0.701 | 0.003 |
| RETREG1 | <0.001 | 0.420 | <0.001 |
| RFESD | <0.001 | 0.267 | <0.001 |
| RFLNA | <0.001 | 0.727 | <0.001 |
| RFPL4AP6 | 0.007 | -0.691 | 0.025 |
| RFX3 | <0.001 | 0.294 | 0.001 |
| RFX3-AS1 | <0.001 | 0.601 | <0.001 |
| RFX8 | 0.001 | 0.451 | 0.007 |
| RGL4 | 0.004 | 0.320 | 0.016 |
| RGMA | <0.001 | -0.289 | 0.005 |
| RGS18 | <0.001 | 0.489 | <0.001 |
| RGS4 | <0.001 | 0.361 | 0.003 |
| RGS9 | <0.001 | 0.484 | 0.005 |
| RHBG | <0.001 | -1.337 | <0.001 |
| RHCG | <0.001 | -0.423 | <0.001 |
| RHD | 0.012 | 0.295 | 0.041 |
| RHEBL1 | <0.001 | 0.306 | <0.001 |
| RHOBTB1 | <0.001 | 0.357 | <0.001 |
| RHOBTB2 | <0.001 | 0.415 | <0.001 |
| RHOF | 0.008 | -0.295 | 0.029 |
| RHOH | 0.005 | 0.278 | 0.019 |
| RHOXF1-AS1 | 0.002 | -0.919 | 0.011 |
| RIC3 | 0.004 | 0.619 | 0.017 |
| RIMS2 | 0.001 | 0.487 | 0.007 |
| RIPOR3 | <0.001 | 0.386 | 0.004 |
| RIPPLY1 | 0.009 | 0.519 | 0.032 |
| RN7SKP116 | 0.002 | -0.423 | 0.009 |
| RN7SKP150 | 0.008 | 0.487 | 0.031 |
| RN7SKP16 | <0.001 | 0.450 | <0.001 |
| RN7SKP296 | 0.002 | 0.419 | 0.008 |
| RN7SL12P | 0.007 | -0.565 | 0.026 |
| RN7SL130P | 0.005 | 0.309 | 0.021 |
| RN7SL181P | <0.001 | -0.347 | 0.001 |
| RN7SL405P | 0.003 | 0.532 | 0.012 |
| RN7SL441P | <0.001 | -1.584 | <0.001 |
| RN7SL689P | <0.001 | 0.279 | <0.001 |
| RN7SL738P | <0.001 | -0.282 | 0.004 |
| RN7SL832P | 0.015 | -0.264 | 0.048 |
| RNASE2 | <0.001 | 0.512 | <0.001 |
| RNASE3 | 0.002 | 0.532 | 0.012 |
| RNASE4 | <0.001 | 0.376 | 0.001 |
| RNASE7 | <0.001 | -0.466 | <0.001 |
| RNASEH2B-AS1 | <0.001 | 0.425 | <0.001 |
| RND1 | <0.001 | -0.349 | 0.003 |
| RNF103-CHMP3 | <0.001 | -0.582 | <0.001 |
| RNF126P1 | <0.001 | -0.921 | 0.004 |
| RNF150 | <0.001 | 0.821 | <0.001 |
| RNF157 | <0.001 | 0.572 | 0.001 |
| RNF180 | 0.013 | 0.301 | 0.044 |
| RNF183 | 0.004 | -0.761 | 0.016 |
| RNF208 | <0.001 | -0.553 | <0.001 |
| RNF212B | <0.001 | 0.564 | 0.004 |
| RNF222 | <0.001 | -1.481 | <0.001 |
| RNF223 | <0.001 | -0.682 | <0.001 |
| RNF224 | <0.001 | -1.133 | <0.001 |
| RNF225 | <0.001 | -1.273 | <0.001 |
| RNF39 | <0.001 | -0.555 | <0.001 |
| RNU1-138P | 0.005 | -0.465 | 0.019 |
| RNU1-153P | <0.001 | 0.540 | 0.002 |
| RNU2-27P | <0.001 | -0.288 | <0.001 |
| RNU6-1161P | <0.001 | -0.354 | 0.002 |
| RNU6-140P | <0.001 | -0.314 | <0.001 |
| RNU6-167P | <0.001 | -1.220 | <0.001 |
| RNU6-181P | 0.010 | -0.444 | 0.037 |
| RNU6-26P | <0.001 | 0.771 | <0.001 |
| RNU6-403P | <0.001 | 0.712 | <0.001 |
| RNU6-510P | 0.002 | 0.303 | 0.008 |
| RNU6-611P | 0.003 | 0.418 | 0.012 |
| RNU7-40P | 0.007 | 0.357 | 0.028 |
| RNVU1-25 | <0.001 | 0.834 | <0.001 |
| RNVU1-3 | <0.001 | 0.764 | <0.001 |
| RNY3P16 | 0.006 | 0.286 | 0.023 |
| ROR1 | <0.001 | 0.630 | <0.001 |
| ROR1-AS1 | 0.002 | 0.885 | 0.010 |
| ROR2 | <0.001 | 0.343 | <0.001 |
| RORA | <0.001 | -0.300 | <0.001 |
| ROS1 | 0.003 | 0.762 | 0.014 |
| RP1 | 0.008 | 0.657 | 0.029 |
| RP1-168P16.2 | 0.012 | -0.347 | 0.040 |
| RP1-309I22.2 | <0.001 | 0.774 | <0.001 |
| RP11-1102P16.1 | 0.002 | 1.138 | 0.011 |
| RP11-141C7.4 | <0.001 | -0.553 | 0.003 |
| RP11-15N24.4 | 0.014 | 0.268 | 0.047 |
| RP11-180P8.1 | 0.003 | 0.463 | 0.013 |
| RP11-180P8.3 | <0.001 | 0.708 | 0.001 |
| RP11-1M18.1 | 0.002 | -0.547 | 0.010 |
| RP11-264E20.1 | 0.001 | 0.520 | 0.006 |
| RP11-264E20.2 | 0.009 | 0.434 | 0.033 |
| RP11-265N7.1 | <0.001 | 0.558 | 0.005 |
| RP11-275I4.2 | <0.001 | -0.781 | <0.001 |
| RP11-285F16.1 | 0.001 | 0.716 | 0.006 |
| RP11-291I6.2 | <0.001 | 0.664 | 0.003 |
| RP11-292E2.4 | 0.011 | 0.534 | 0.039 |
| RP11-314C16.1 | 0.002 | 0.377 | 0.010 |
| RP11-333I13.1 | <0.001 | 0.401 | 0.003 |
| RP11-337C18.9 | 0.003 | 0.264 | 0.012 |
| RP11-345J18.2 | <0.001 | 0.631 | <0.001 |
| RP11-380L11.4 | 0.009 | 0.330 | 0.033 |
| RP11-389O22.1 | <0.001 | 0.458 | 0.003 |
| RP11-394O4.5 | <0.001 | 0.425 | 0.001 |
| RP11-408E5.5 | <0.001 | -0.924 | <0.001 |
| RP11-467J12.4 | 0.006 | -0.289 | 0.022 |
| RP11-488P3.1 | <0.001 | 0.771 | <0.001 |
| RP11-53M11.3 | <0.001 | 0.582 | 0.005 |
| RP11-541P9.3 | 0.002 | 0.517 | 0.010 |
| RP11-554A11.9 | 0.007 | 0.499 | 0.028 |
| RP11-556E13.1 | <0.001 | 0.514 | 0.001 |
| RP11-61L19.3 | <0.001 | -0.324 | <0.001 |
| RP11-680F20.6 | 0.004 | -0.922 | 0.018 |
| RP11-69E11.4 | <0.001 | 0.505 | <0.001 |
| RP11-701H24.7 | 0.005 | 0.384 | 0.021 |
| RP11-723O4.6 | <0.001 | -1.260 | <0.001 |
| RP11-803D5.1 | <0.001 | 0.541 | <0.001 |
| RP11-86H7.7 | <0.001 | 0.636 | <0.001 |
| RP11-972P1.7 | 0.002 | 0.322 | 0.009 |
| RP13-476E20.1 | <0.001 | -0.457 | 0.001 |
| RP3-331H24.7 | <0.001 | 0.700 | 0.004 |
| RP3-355L5.5 | <0.001 | 0.321 | 0.002 |
| RP4-575N6.5 | <0.001 | 0.533 | 0.005 |
| RP4-614O4.12 | <0.001 | 0.522 | 0.003 |
| RP4-737E23.7 | 0.002 | -0.661 | 0.009 |
| RP5-857K21.1 | <0.001 | -0.924 | <0.001 |
| RP5-886K2.1 | 0.011 | 0.433 | 0.037 |
| RPE65 | <0.001 | -1.076 | <0.001 |
| RPH3AL | 0.003 | -0.315 | 0.015 |
| RPL10P1 | 0.011 | 0.300 | 0.038 |
| RPL10P12 | 0.003 | 0.363 | 0.015 |
| RPL10P13 | 0.008 | -0.295 | 0.031 |
| RPL10P7 | <0.001 | 0.553 | 0.003 |
| RPL11P3 | 0.001 | 0.332 | 0.007 |
| RPL12P10 | 0.013 | 0.301 | 0.045 |
| RPL12P12 | 0.003 | -0.384 | 0.012 |
| RPL12P27 | 0.006 | 0.459 | 0.025 |
| RPL12P6 | 0.014 | 0.312 | 0.046 |
| RPL13AP2 | 0.006 | 0.394 | 0.024 |
| RPL13AP23 | 0.009 | 0.377 | 0.032 |
| RPL13AP3 | 0.009 | 0.299 | 0.031 |
| RPL17P50 | <0.001 | 0.383 | <0.001 |
| RPL21P120 | 0.006 | 0.294 | 0.024 |
| RPL21P13 | <0.001 | -1.174 | 0.001 |
| RPL22P12 | 0.009 | 0.398 | 0.034 |
| RPL23AP10 | 0.009 | -0.609 | 0.033 |
| RPL23AP18 | 0.009 | 0.274 | 0.033 |
| RPL23AP49 | 0.014 | 0.401 | 0.046 |
| RPL23AP87 | <0.001 | -0.580 | 0.005 |
| RPL23AP88 | 0.004 | 0.458 | 0.017 |
| RPL23AP97 | <0.001 | 0.452 | <0.001 |
| RPL24P7 | 0.011 | 0.450 | 0.037 |
| RPL29P19 | <0.001 | 0.466 | <0.001 |
| RPL32P1 | <0.001 | 0.614 | 0.003 |
| RPL35AP28 | 0.005 | -0.357 | 0.020 |
| RPL37P23 | 0.008 | 0.263 | 0.030 |
| RPL37P3 | 0.007 | -0.507 | 0.027 |
| RPL3P10 | 0.002 | 0.567 | 0.011 |
| RPL4P1 | <0.001 | 0.596 | <0.001 |
| RPL7AP14 | <0.001 | 0.625 | <0.001 |
| RPL7AP58 | 0.012 | 0.466 | 0.040 |
| RPL7AP64 | <0.001 | -0.589 | <0.001 |
| RPL7AP8 | 0.011 | 0.414 | 0.038 |
| RPL7L1P3 | <0.001 | -0.601 | 0.002 |
| RPL7L1P8 | <0.001 | -1.201 | <0.001 |
| RPL7P16 | 0.005 | -0.615 | 0.019 |
| RPL9P18 | 0.014 | 0.303 | 0.047 |
| RPL9P2 | 0.011 | 0.338 | 0.038 |
| RPLP0P2 | <0.001 | 0.366 | <0.001 |
| RPS10P5 | <0.001 | -0.282 | 0.002 |
| RPS11P7 | <0.001 | 0.666 | 0.005 |
| RPS15AP38 | 0.012 | 0.305 | 0.040 |
| RPS16P9 | 0.003 | -0.909 | 0.013 |
| RPS27P29 | 0.011 | 0.438 | 0.038 |
| RPS29P17 | 0.006 | 0.373 | 0.023 |
| RPS2P44 | 0.008 | -0.459 | 0.030 |
| RPS4XP1 | 0.010 | 0.276 | 0.035 |
| RPSAP11 | 0.009 | 0.339 | 0.031 |
| RPSAP18 | 0.002 | 0.325 | 0.008 |
| RPSAP4 | 0.010 | 0.299 | 0.035 |
| RPSAP45 | 0.008 | 0.374 | 0.031 |
| RPSAP46 | 0.007 | 0.363 | 0.027 |
| RPSAP47 | 0.008 | 0.381 | 0.029 |
| RPSAP52 | <0.001 | 0.683 | <0.001 |
| RPTN | <0.001 | -1.663 | <0.001 |
| RRN3P2 | <0.001 | 0.395 | 0.001 |
| RSPO3 | <0.001 | 0.630 | <0.001 |
| RTEL1 | <0.001 | 0.272 | <0.001 |
| RTEL1-TNFRSF6B | <0.001 | 0.339 | <0.001 |
| RTL3 | <0.001 | 0.573 | 0.004 |
| RTL5 | <0.001 | 0.425 | 0.003 |
| RTL9 | 0.003 | 0.528 | 0.013 |
| RTN4RL1 | <0.001 | 0.355 | 0.004 |
| RTP3 | <0.001 | 0.744 | 0.005 |
| RTTN | <0.001 | 0.321 | <0.001 |
| RUBCNL | <0.001 | 0.607 | <0.001 |
| RUNX1T1 | <0.001 | 0.491 | 0.004 |
| RUNX2 | <0.001 | 0.469 | <0.001 |
| RXFP1 | 0.004 | 1.247 | 0.018 |
| RXFP3 | 0.002 | 0.711 | 0.009 |
| RXRG | <0.001 | 0.945 | 0.003 |
| RYR3 | 0.004 | 0.588 | 0.015 |
| S100A1 | 0.003 | 0.392 | 0.013 |
| S100A12 | <0.001 | -0.689 | <0.001 |
| S100A15A | <0.001 | -1.479 | <0.001 |
| S100A5 | 0.001 | -0.459 | 0.006 |
| S100A7 | <0.001 | -0.305 | <0.001 |
| S100A7A | <0.001 | -0.642 | <0.001 |
| S100P | <0.001 | -0.487 | <0.001 |
| S100Z | 0.011 | 0.397 | 0.038 |
| S1PR3 | <0.001 | 0.587 | <0.001 |
| SACS | <0.001 | 0.274 | <0.001 |
| SALL1 | 0.003 | 0.589 | 0.014 |
| SALL4 | <0.001 | 0.432 | 0.004 |
| SAMD11 | 0.004 | 0.466 | 0.017 |
| SAMD12 | <0.001 | -0.447 | <0.001 |
| SAMD12-AS1 | <0.001 | -0.792 | <0.001 |
| SAMD13 | <0.001 | 0.548 | <0.001 |
| SAMD14 | <0.001 | 0.573 | <0.001 |
| SAMD3 | <0.001 | 0.676 | <0.001 |
| SAMD4A | <0.001 | 0.274 | <0.001 |
| SAP30-DT | 0.001 | 0.273 | 0.007 |
| SARDH | <0.001 | 0.576 | <0.001 |
| SARM1 | 0.003 | 0.334 | 0.013 |
| SASH1 | <0.001 | -0.278 | <0.001 |
| SBSN | <0.001 | -0.529 | <0.001 |
| SCAPER | <0.001 | -0.285 | <0.001 |
| SCARF2 | <0.001 | 0.353 | <0.001 |
| SCAT1 | <0.001 | 0.540 | <0.001 |
| SCEL | <0.001 | -0.782 | <0.001 |
| SCG2 | <0.001 | 0.448 | 0.005 |
| SCG5 | <0.001 | 0.612 | <0.001 |
| SCHIP1 | <0.001 | 0.449 | <0.001 |
| SCIMP | <0.001 | 0.461 | 0.003 |
| SCML4 | 0.014 | 0.459 | 0.046 |
| SCN11A | 0.003 | 0.862 | 0.014 |
| SCN1B | <0.001 | 0.386 | 0.001 |
| SCN4A | 0.009 | 0.738 | 0.033 |
| SCN5A | <0.001 | 0.839 | <0.001 |
| SCN9A | 0.003 | -0.702 | 0.015 |
| SCNN1A | <0.001 | -0.587 | <0.001 |
| SCNN1B | <0.001 | -0.530 | <0.001 |
| SCNN1D | <0.001 | -0.747 | <0.001 |
| SCNN1G | 0.003 | -0.353 | 0.015 |
| SCT | <0.001 | 0.539 | <0.001 |
| SCUBE1 | <0.001 | 0.558 | 0.003 |
| SDAD1P2 | 0.005 | -0.701 | 0.022 |
| SDC2 | <0.001 | 0.362 | <0.001 |
| SDC3 | <0.001 | 0.294 | <0.001 |
| SDCBP2 | <0.001 | -0.405 | <0.001 |
| SDCBP2-AS1 | <0.001 | 0.284 | <0.001 |
| SDHDP6 | <0.001 | 0.729 | <0.001 |
| SDK2 | <0.001 | 0.340 | <0.001 |
| SDR16C5 | <0.001 | -0.319 | <0.001 |
| SDR42E1 | <0.001 | -0.269 | <0.001 |
| SDR9C7 | <0.001 | -0.982 | <0.001 |
| SDS | <0.001 | 0.314 | 0.002 |
| SDSL | <0.001 | 0.439 | <0.001 |
| SEC14L6 | 0.008 | 0.604 | 0.029 |
| SEC22B4P | 0.002 | 0.513 | 0.011 |
| SEC31B | 0.002 | 0.338 | 0.011 |
| SEMA3A | <0.001 | 0.538 | <0.001 |
| SEMA5A | 0.004 | 0.266 | 0.016 |
| SEMA5B | <0.001 | 0.609 | <0.001 |
| SEMA6A | <0.001 | 0.441 | <0.001 |
| SEMA6A-AS1 | 0.002 | 0.511 | 0.008 |
| SENCR | <0.001 | 0.606 | <0.001 |
| SEPHS1P6 | 0.016 | 0.566 | 0.050 |
| SEPTIN4 | <0.001 | 0.459 | <0.001 |
| SEPTIN4-AS1 | <0.001 | 0.588 | <0.001 |
| SEPTIN7-AS1 | <0.001 | 0.381 | <0.001 |
| SEPTIN9-DT | <0.001 | -0.523 | <0.001 |
| SERAC1 | <0.001 | -0.311 | <0.001 |
| SERP2 | <0.001 | 0.527 | 0.003 |
| SERPINA1 | <0.001 | 0.271 | <0.001 |
| SERPINA10 | 0.007 | 0.586 | 0.025 |
| SERPINA12 | 0.001 | -1.212 | 0.006 |
| SERPINA5 | <0.001 | 1.044 | <0.001 |
| SERPINB11 | <0.001 | -1.318 | <0.001 |
| SERPINB12 | <0.001 | -1.080 | <0.001 |
| SERPINB13 | <0.001 | -0.424 | <0.001 |
| SERPINB2 | <0.001 | -0.507 | <0.001 |
| SERPINB3 | <0.001 | -0.425 | <0.001 |
| SERPINB4 | <0.001 | -0.369 | <0.001 |
| SERPINB7 | <0.001 | -0.436 | <0.001 |
| SEZ6L2 | <0.001 | 0.374 | <0.001 |
| SFRP1 | <0.001 | 0.459 | <0.001 |
| SFRP5 | 0.002 | 1.084 | 0.011 |
| SFTA2 | <0.001 | -1.827 | <0.001 |
| SGCA | 0.009 | 0.481 | 0.034 |
| SGCD | <0.001 | 0.634 | <0.001 |
| SGIP1 | <0.001 | 0.367 | 0.004 |
| SGPP2 | <0.001 | -0.358 | <0.001 |
| SH2B3 | <0.001 | 0.271 | <0.001 |
| SH2D1A | <0.001 | 0.419 | 0.003 |
| SH2D1B | 0.009 | -0.540 | 0.034 |
| SH2D4B | 0.001 | 0.543 | 0.007 |
| SH3BGR | <0.001 | 0.350 | <0.001 |
| SH3BGRL2 | <0.001 | -0.359 | <0.001 |
| SH3D21 | <0.001 | -0.279 | <0.001 |
| SH3PXD2A-AS1 | <0.001 | -0.482 | <0.001 |
| SH3RF3 | <0.001 | 0.352 | <0.001 |
| SH3RF3-AS1 | <0.001 | 0.402 | <0.001 |
| SH3TC2 | 0.002 | 0.394 | 0.010 |
| SHANK1 | <0.001 | 0.524 | 0.003 |
| SHANK2 | <0.001 | 0.519 | 0.002 |
| SHBG | 0.006 | 0.348 | 0.025 |
| SHC2 | 0.003 | 0.264 | 0.014 |
| SHC3 | 0.002 | 0.407 | 0.010 |
| SHE | <0.001 | 0.417 | 0.003 |
| SHISA2 | <0.001 | 0.654 | <0.001 |
| SHISA3 | <0.001 | 1.860 | <0.001 |
| SHISA4 | <0.001 | 0.273 | <0.001 |
| SHISAL1 | <0.001 | 0.449 | <0.001 |
| SHISAL2A | <0.001 | 0.580 | 0.002 |
| SHLD2P1 | <0.001 | 0.555 | 0.002 |
| SHLD2P3 | <0.001 | 0.403 | 0.004 |
| SHROOM4 | <0.001 | 0.412 | <0.001 |
| SIAH2-AS1 | 0.002 | -0.345 | 0.009 |
| SIGLEC1 | <0.001 | 0.426 | <0.001 |
| SIGLEC10 | <0.001 | 0.392 | <0.001 |
| SIGLEC12 | 0.003 | 0.429 | 0.013 |
| SIGLEC14 | <0.001 | 0.413 | 0.002 |
| SIGLEC5 | 0.003 | 0.392 | 0.015 |
| SIGLEC7 | <0.001 | 0.470 | <0.001 |
| SIGLEC8 | <0.001 | 0.660 | 0.003 |
| SIGLEC9 | <0.001 | 0.345 | <0.001 |
| SIK1 | 0.002 | 0.535 | 0.009 |
| SIM1 | <0.001 | 2.440 | <0.001 |
| SIRPD | 0.002 | 0.620 | 0.008 |
| SIRPG | <0.001 | 0.410 | 0.001 |
| SKAP1 | 0.004 | 0.314 | 0.017 |
| SKINT1L | 0.004 | 0.927 | 0.016 |
| SLA2 | <0.001 | 0.358 | 0.002 |
| SLAMF1 | 0.007 | 0.299 | 0.026 |
| SLAMF8 | <0.001 | 0.302 | <0.001 |
| SLAMF9 | 0.005 | -0.399 | 0.019 |
| SLC10A1 | <0.001 | -0.659 | <0.001 |
| SLC10A6 | <0.001 | -0.578 | <0.001 |
| SLC12A5-AS1 | 0.004 | 0.497 | 0.017 |
| SLC12A6 | <0.001 | -0.314 | <0.001 |
| SLC13A2 | 0.008 | -0.841 | 0.031 |
| SLC13A3 | <0.001 | 0.414 | 0.005 |
| SLC13A4 | <0.001 | -0.987 | <0.001 |
| SLC14A1 | 0.007 | 0.860 | 0.026 |
| SLC14A2 | 0.003 | 0.678 | 0.013 |
| SLC15A1 | <0.001 | -0.969 | <0.001 |
| SLC15A2 | <0.001 | -0.294 | 0.004 |
| SLC16A12 | <0.001 | 1.041 | 0.004 |
| SLC16A14 | <0.001 | -0.379 | 0.001 |
| SLC16A2 | <0.001 | 0.323 | <0.001 |
| SLC16A4 | 0.008 | 0.284 | 0.031 |
| SLC16A6 | <0.001 | -0.576 | <0.001 |
| SLC16A7 | 0.008 | -0.321 | 0.029 |
| SLC16A9 | <0.001 | -0.753 | <0.001 |
| SLC17A7 | 0.010 | 0.595 | 0.034 |
| SLC1A7 | 0.014 | 0.542 | 0.046 |
| SLC22A16 | 0.006 | 0.862 | 0.024 |
| SLC22A20P | <0.001 | -0.458 | 0.001 |
| SLC22A3 | <0.001 | 0.453 | <0.001 |
| SLC22A4 | <0.001 | 0.514 | <0.001 |
| SLC23A1 | <0.001 | -0.883 | <0.001 |
| SLC24A2 | <0.001 | 0.504 | 0.004 |
| SLC24A4 | <0.001 | 0.487 | 0.004 |
| SLC25A25 | <0.001 | -0.274 | <0.001 |
| SLC25A27 | 0.003 | 0.334 | 0.015 |
| SLC25A34-AS1 | 0.003 | 0.617 | 0.015 |
| SLC25A48 | 0.005 | -0.647 | 0.021 |
| SLC26A10 | 0.009 | 0.463 | 0.031 |
| SLC26A9 | <0.001 | -1.008 | <0.001 |
| SLC27A1 | <0.001 | 0.282 | <0.001 |
| SLC27A5 | 0.002 | -0.373 | 0.008 |
| SLC27A6 | 0.013 | 0.700 | 0.045 |
| SLC2A1-AS1 | <0.001 | -0.332 | 0.004 |
| SLC2A3 | <0.001 | 0.279 | <0.001 |
| SLC2A3P4 | 0.008 | 0.396 | 0.028 |
| SLC2A6 | <0.001 | 0.352 | <0.001 |
| SLC34A1 | <0.001 | -0.531 | 0.001 |
| SLC34A3 | <0.001 | -1.158 | <0.001 |
| SLC35E2A | 0.001 | 0.266 | 0.006 |
| SLC35F3 | 0.007 | 0.320 | 0.026 |
| SLC35G1 | 0.008 | -0.335 | 0.030 |
| SLC38A8 | <0.001 | -1.510 | <0.001 |
| SLC39A13 | <0.001 | 0.274 | <0.001 |
| SLC39A2 | <0.001 | -0.633 | <0.001 |
| SLC39A5 | 0.009 | 0.483 | 0.033 |
| SLC41A2 | <0.001 | 0.374 | <0.001 |
| SLC43A1 | <0.001 | 0.301 | 0.001 |
| SLC44A3-AS1 | <0.001 | 0.429 | <0.001 |
| SLC44A4 | 0.008 | -0.447 | 0.030 |
| SLC45A1 | <0.001 | 0.673 | <0.001 |
| SLC45A2 | <0.001 | 0.894 | <0.001 |
| SLC46A2 | <0.001 | -0.979 | <0.001 |
| SLC46A3 | <0.001 | 0.264 | <0.001 |
| SLC4A11 | <0.001 | -0.399 | <0.001 |
| SLC4A3 | <0.001 | 0.281 | <0.001 |
| SLC51B | 0.005 | 0.418 | 0.022 |
| SLC52A3 | <0.001 | -0.348 | <0.001 |
| SLC5A1 | <0.001 | -0.879 | <0.001 |
| SLC5A10 | <0.001 | -1.102 | <0.001 |
| SLC5A11 | 0.001 | -1.101 | 0.006 |
| SLC5A5 | 0.015 | -0.569 | 0.050 |
| SLC5A9 | 0.011 | 0.478 | 0.038 |
| SLC6A12 | <0.001 | 0.559 | 0.002 |
| SLC6A14 | <0.001 | -0.533 | <0.001 |
| SLC6A16 | <0.001 | 0.558 | <0.001 |
| SLC6A17 | 0.003 | 0.640 | 0.012 |
| SLC6A4 | <0.001 | -1.203 | <0.001 |
| SLC7A10 | 0.003 | 1.685 | 0.013 |
| SLC7A11 | <0.001 | -0.446 | <0.001 |
| SLC7A7 | <0.001 | 0.358 | <0.001 |
| SLC7A9 | <0.001 | 1.015 | 0.002 |
| SLC8A1 | <0.001 | 0.537 | <0.001 |
| SLC8A3 | <0.001 | 0.949 | 0.002 |
| SLC9A3 | 0.007 | -0.372 | 0.025 |
| SLC9A4 | <0.001 | -1.360 | 0.002 |
| SLC9A5 | 0.002 | 0.305 | 0.008 |
| SLC9B1 | 0.001 | -0.372 | 0.006 |
| SLCO1B3 | 0.005 | -0.461 | 0.019 |
| SLCO2B1 | <0.001 | 0.346 | <0.001 |
| SLCO4A1 | <0.001 | -0.265 | 0.001 |
| SLCO4A1-AS1 | <0.001 | -0.528 | 0.005 |
| SLCO5A1 | <0.001 | 0.653 | 0.003 |
| SLFN12L | <0.001 | 0.370 | 0.002 |
| SLIT3 | <0.001 | 0.318 | 0.004 |
| SLITRK6 | <0.001 | 0.472 | <0.001 |
| SLURP1 | <0.001 | -0.948 | <0.001 |
| SMAD5-AS1 | <0.001 | -0.617 | 0.003 |
| SMAGP | <0.001 | -0.269 | <0.001 |
| SMARCD3 | <0.001 | 0.299 | <0.001 |
| SMARCE1P6 | <0.001 | -0.554 | 0.003 |
| SMCP | <0.001 | -2.040 | <0.001 |
| SMIM1 | 0.007 | 0.299 | 0.026 |
| SMIM10L2B | 0.003 | -0.317 | 0.014 |
| SMIM17 | <0.001 | 0.785 | <0.001 |
| SMIM2 | 0.004 | 0.556 | 0.018 |
| SMIM2-AS1 | <0.001 | 0.584 | <0.001 |
| SMIM35 | <0.001 | -1.218 | <0.001 |
| SMIM5 | <0.001 | -1.188 | <0.001 |
| SMOC1 | <0.001 | 0.825 | <0.001 |
| SMPD3 | <0.001 | -0.850 | <0.001 |
| SMTNL2 | 0.008 | 0.641 | 0.030 |
| SMYD1 | 0.005 | 0.742 | 0.021 |
| SNAI1 | <0.001 | 0.271 | <0.001 |
| SNAI3-AS1 | 0.004 | 0.292 | 0.016 |
| SNAP25 | 0.012 | 0.526 | 0.041 |
| SND1-IT1 | 0.009 | -0.564 | 0.033 |
| SNED1 | <0.001 | 0.397 | 0.002 |
| SNHG14 | 0.007 | 0.325 | 0.028 |
| SNHG24 | 0.006 | 1.051 | 0.024 |
| SNHG28 | <0.001 | -0.563 | <0.001 |
| SNORA14A | 0.012 | -0.581 | 0.040 |
| SNORA70F | 0.003 | -0.555 | 0.014 |
| SNORA80B | <0.001 | -0.825 | <0.001 |
| SNPH | <0.001 | 0.382 | <0.001 |
| SNRK-AS1 | <0.001 | 0.739 | 0.003 |
| SNRPCP2 | <0.001 | 0.574 | 0.005 |
| SNTB1 | <0.001 | 0.409 | <0.001 |
| SNTG2-AS1 | 0.014 | 0.740 | 0.046 |
| SNX20 | <0.001 | 0.309 | 0.004 |
| SNX31 | 0.006 | -0.529 | 0.024 |
| SOBP | <0.001 | 0.492 | <0.001 |
| SOCAR | <0.001 | 0.460 | <0.001 |
| SOGA3 | <0.001 | 0.689 | <0.001 |
| SORBS1 | 0.004 | 0.291 | 0.018 |
| SORCS1 | <0.001 | 1.043 | 0.002 |
| SORCS2 | <0.001 | 0.346 | 0.001 |
| SOWAHB | <0.001 | -0.691 | <0.001 |
| SOX11 | 0.002 | 0.621 | 0.010 |
| SOX2 | <0.001 | -0.352 | <0.001 |
| SOX21 | <0.001 | -0.711 | <0.001 |
| SOX21-AS1 | <0.001 | -0.627 | <0.001 |
| SOX8 | 0.011 | 0.731 | 0.039 |
| SOX9-AS1 | <0.001 | 0.996 | <0.001 |
| SP5 | 0.002 | 0.752 | 0.009 |
| SPA17P1 | 0.012 | 0.524 | 0.040 |
| SPAAR | <0.001 | 0.330 | 0.002 |
| SPACA4 | <0.001 | -0.840 | <0.001 |
| SPACA6 | <0.001 | 0.373 | <0.001 |
| SPACA6P-AS | <0.001 | 0.765 | <0.001 |
| SPAG17 | <0.001 | -0.879 | <0.001 |
| SPAG8 | 0.003 | 0.435 | 0.015 |
| SPATA13 | <0.001 | 0.296 | <0.001 |
| SPATA17 | <0.001 | 0.585 | <0.001 |
| SPATA20P1 | <0.001 | 0.421 | 0.003 |
| SPATA32 | 0.009 | 0.320 | 0.032 |
| SPATA41 | <0.001 | -0.375 | <0.001 |
| SPATA45 | <0.001 | 0.652 | 0.005 |
| SPATA9 | <0.001 | 0.428 | <0.001 |
| SPDYC | <0.001 | -1.476 | <0.001 |
| SPECC1 | <0.001 | 0.312 | <0.001 |
| SPIB | <0.001 | 1.158 | <0.001 |
| SPIC | 0.005 | 1.278 | 0.019 |
| SPINK5 | <0.001 | -0.561 | <0.001 |
| SPINK7 | <0.001 | -1.319 | <0.001 |
| SPN | <0.001 | 0.344 | 0.002 |
| SPNS2 | <0.001 | -0.597 | <0.001 |
| SPOCD1 | <0.001 | 0.481 | <0.001 |
| SPOCK1 | <0.001 | 0.571 | <0.001 |
| SPOCK3 | <0.001 | 0.904 | 0.005 |
| SPON1-AS1 | <0.001 | 0.668 | <0.001 |
| SPRR1A | <0.001 | -0.539 | <0.001 |
| SPRR1B | <0.001 | -0.393 | <0.001 |
| SPRR2A | <0.001 | -0.503 | <0.001 |
| SPRR2B | <0.001 | -1.025 | <0.001 |
| SPRR2C | <0.001 | -0.906 | <0.001 |
| SPRR2D | <0.001 | -0.541 | <0.001 |
| SPRR2E | <0.001 | -0.601 | <0.001 |
| SPRR2F | <0.001 | -1.046 | <0.001 |
| SPRR2G | <0.001 | -0.636 | <0.001 |
| SPRR3 | <0.001 | -0.846 | <0.001 |
| SPRR4 | <0.001 | -1.253 | <0.001 |
| SPRY4-AS1 | <0.001 | 0.706 | <0.001 |
| SPTBN2 | <0.001 | -0.277 | <0.001 |
| SPTLC1P4 | <0.001 | -1.026 | 0.001 |
| SPTSSB | <0.001 | -0.997 | <0.001 |
| SRGAP2B | <0.001 | 0.293 | <0.001 |
| SRMS | <0.001 | -0.608 | <0.001 |
| SRP14-AS1 | <0.001 | 0.371 | <0.001 |
| SRPX | <0.001 | 0.382 | <0.001 |
| SRRM3 | <0.001 | -0.295 | 0.002 |
| SRSF12 | 0.007 | 0.439 | 0.027 |
| SSC5D | <0.001 | 0.356 | 0.003 |
| SSPN | <0.001 | 0.505 | <0.001 |
| SSR4P1 | <0.001 | 0.270 | 0.002 |
| SST | <0.001 | -1.212 | <0.001 |
| SSTR2 | <0.001 | 1.028 | <0.001 |
| SSTR3 | <0.001 | 0.750 | <0.001 |
| SSTR5 | 0.011 | -0.578 | 0.039 |
| SSUH2 | 0.001 | 0.449 | 0.006 |
| ST13P20 | <0.001 | -1.161 | <0.001 |
| ST20-MTHFS | 0.005 | 0.361 | 0.020 |
| ST3GAL2 | <0.001 | 0.401 | <0.001 |
| ST3GAL5 | <0.001 | 0.439 | <0.001 |
| ST3GAL5-AS1 | <0.001 | 0.611 | <0.001 |
| ST3GAL6-AS1 | 0.002 | 0.342 | 0.010 |
| ST6GAL2 | 0.011 | 0.523 | 0.037 |
| ST6GALNAC1 | <0.001 | -0.696 | <0.001 |
| ST6GALNAC3 | <0.001 | 0.387 | 0.001 |
| ST6GALNAC5 | 0.003 | 0.355 | 0.014 |
| ST8SIA1 | <0.001 | 0.505 | <0.001 |
| ST8SIA5 | 0.005 | 0.657 | 0.019 |
| ST8SIA6 | 0.009 | -0.431 | 0.034 |
| STAC | <0.001 | 0.546 | 0.002 |
| STAC3 | <0.001 | 0.408 | 0.002 |
| STAMBPL1 | <0.001 | 0.265 | <0.001 |
| STARD13 | <0.001 | 0.448 | <0.001 |
| STARD13-AS | 0.001 | 0.578 | 0.006 |
| STARD3NL | <0.001 | 0.294 | <0.001 |
| STARD4-AS1 | <0.001 | 0.582 | <0.001 |
| STARD8 | <0.001 | 0.351 | <0.001 |
| STARD9 | <0.001 | 0.498 | <0.001 |
| STAT4 | <0.001 | 0.359 | <0.001 |
| STBD1 | <0.001 | 0.269 | 0.003 |
| STC1 | <0.001 | 0.292 | <0.001 |
| STEAP1B | 0.010 | -0.329 | 0.035 |
| STEAP4 | <0.001 | -0.432 | <0.001 |
| STK24-AS1 | 0.001 | -0.349 | 0.006 |
| STK33 | 0.002 | 0.720 | 0.011 |
| STK4-AS1 | <0.001 | 0.307 | 0.001 |
| STMN2 | <0.001 | 0.987 | 0.003 |
| STON1-GTF2A1L | 0.007 | 0.463 | 0.025 |
| STPG3 | 0.010 | -0.563 | 0.036 |
| STRA6LP | 0.004 | 0.546 | 0.016 |
| STUM | 0.001 | 0.736 | 0.007 |
| STX17-AS1 | <0.001 | -0.527 | <0.001 |
| STX19 | <0.001 | -0.770 | <0.001 |
| STX1A | <0.001 | 0.279 | <0.001 |
| STX2 | <0.001 | 0.346 | <0.001 |
| SUCLA2-AS1 | <0.001 | -0.415 | <0.001 |
| SUCLG2-AS1 | 0.005 | 0.300 | 0.022 |
| SUCNR1 | <0.001 | 0.624 | <0.001 |
| SUGT1P1 | <0.001 | 0.351 | 0.003 |
| SUGT1P4-STRA6LP | 0.002 | 0.480 | 0.009 |
| SULT1B1 | <0.001 | -0.945 | <0.001 |
| SULT1C4 | 0.009 | 0.429 | 0.033 |
| SULT2B1 | <0.001 | -0.700 | <0.001 |
| SUMO2P17 | 0.002 | 0.284 | 0.009 |
| SUPT3H | <0.001 | 0.286 | <0.001 |
| SUSD2 | <0.001 | 0.422 | <0.001 |
| SUSD3 | <0.001 | 0.310 | <0.001 |
| SUSD4 | 0.001 | -0.276 | 0.008 |
| SUSD5 | <0.001 | 0.910 | <0.001 |
| SV2A | 0.004 | 0.330 | 0.017 |
| SV2B | <0.001 | 1.232 | <0.001 |
| SYDE1 | <0.001 | 0.277 | <0.001 |
| SYN1 | 0.005 | 0.351 | 0.019 |
| SYNC | <0.001 | 0.346 | 0.001 |
| SYNDIG1 | <0.001 | 0.582 | <0.001 |
| SYNDIG1L | <0.001 | 1.048 | 0.003 |
| SYNE1 | <0.001 | 0.555 | <0.001 |
| SYNE3 | <0.001 | 0.435 | <0.001 |
| SYNPO2 | <0.001 | 0.501 | <0.001 |
| SYPL2 | 0.002 | 0.698 | 0.009 |
| SYS1-DBNDD2 | <0.001 | 0.417 | <0.001 |
| SYT11 | <0.001 | 0.337 | <0.001 |
| SYT14P1 | <0.001 | -1.750 | <0.001 |
| SYT16 | 0.006 | 0.806 | 0.025 |
| SYT6 | 0.004 | 0.987 | 0.016 |
| SYT7 | <0.001 | 0.317 | <0.001 |
| SYT8 | 0.009 | -0.377 | 0.032 |
| SYTL2 | 0.004 | 0.275 | 0.018 |
| SYTL5 | <0.001 | -0.963 | <0.001 |
| TAFA2 | 0.002 | 0.374 | 0.010 |
| TAFA3 | <0.001 | 0.791 | <0.001 |
| TAFA5 | <0.001 | 0.486 | <0.001 |
| TAL2 | <0.001 | 1.043 | <0.001 |
| TAS1R1 | 0.001 | -0.950 | 0.007 |
| TAS1R3 | <0.001 | -0.690 | <0.001 |
| TAS2R10 | <0.001 | 0.968 | <0.001 |
| TAS2R60 | <0.001 | -0.893 | <0.001 |
| TATDN1P1 | <0.001 | 0.465 | 0.004 |
| TBC1D10C | 0.002 | 0.307 | 0.008 |
| TBC1D30 | 0.007 | -0.283 | 0.027 |
| TBC1D3B | 0.014 | -0.438 | 0.045 |
| TBCAP3 | 0.007 | -0.414 | 0.027 |
| TBL1X | <0.001 | -0.329 | <0.001 |
| TBX15 | <0.001 | 0.534 | <0.001 |
| TBX19 | <0.001 | 0.296 | <0.001 |
| TBX21 | 0.002 | 0.400 | 0.011 |
| TBX6 | <0.001 | -0.570 | <0.001 |
| TBXA2R | <0.001 | 0.578 | <0.001 |
| TCAF1P1 | 0.004 | 0.269 | 0.017 |
| TCEAL7 | <0.001 | 0.556 | <0.001 |
| TCF7 | <0.001 | 0.541 | <0.001 |
| TCHH | <0.001 | -0.766 | 0.002 |
| TCHHL1 | 0.013 | -0.674 | 0.043 |
| TCHP | <0.001 | -0.274 | <0.001 |
| TCL6 | 0.011 | 0.857 | 0.039 |
| TCN1 | <0.001 | -0.447 | 0.003 |
| TCP10L | 0.003 | 0.453 | 0.015 |
| TCP11L2 | <0.001 | -0.279 | <0.001 |
| TCP1P3 | 0.015 | 0.362 | 0.048 |
| TCTN1 | <0.001 | 0.295 | <0.001 |
| TDRD10 | 0.003 | 0.862 | 0.013 |
| TDRD6 | <0.001 | 0.460 | 0.001 |
| TDRKH | <0.001 | 0.456 | <0.001 |
| TDRKH-AS1 | 0.002 | 0.433 | 0.011 |
| TEC | <0.001 | -0.529 | <0.001 |
| TEDDM1 | 0.011 | -0.566 | 0.037 |
| TEKT3 | 0.002 | 0.524 | 0.009 |
| TENM3 | <0.001 | 0.489 | <0.001 |
| TENM3-AS1 | <0.001 | 0.488 | 0.003 |
| TENT5B | <0.001 | -0.264 | <0.001 |
| TERT | <0.001 | 0.701 | <0.001 |
| TESC | <0.001 | 0.545 | <0.001 |
| TESMIN | 0.009 | 0.309 | 0.034 |
| TESPA1 | <0.001 | 0.510 | <0.001 |
| TEX101 | <0.001 | -0.940 | <0.001 |
| TEX14 | 0.001 | 0.418 | 0.008 |
| TEX21P | <0.001 | 0.582 | <0.001 |
| TEX29 | 0.010 | 0.371 | 0.035 |
| TEX45 | 0.015 | 0.534 | 0.048 |
| TEX48 | 0.012 | 0.561 | 0.042 |
| TEX9 | 0.005 | 0.270 | 0.019 |
| TF | 0.005 | -0.603 | 0.020 |
| TFAP2E | <0.001 | -0.556 | <0.001 |
| TFEC | <0.001 | 0.474 | <0.001 |
| TFPI | <0.001 | 0.364 | <0.001 |
| TFPI2 | 0.005 | 0.494 | 0.022 |
| TFR2 | 0.013 | -0.332 | 0.043 |
| TGFB1I1 | <0.001 | 0.289 | <0.001 |
| TGFB2 | <0.001 | 0.540 | <0.001 |
| TGFB2-AS1 | <0.001 | 0.606 | <0.001 |
| TGFB3-AS1 | 0.003 | 0.621 | 0.012 |
| TGFBR3 | <0.001 | -0.376 | <0.001 |
| TGIF2-RAB5IF | <0.001 | 0.452 | 0.001 |
| TGM1 | <0.001 | -0.667 | <0.001 |
| TGM2 | <0.001 | 0.290 | <0.001 |
| TGM3 | <0.001 | -1.407 | <0.001 |
| TGM5 | <0.001 | -0.938 | <0.001 |
| TGM6 | <0.001 | -1.776 | <0.001 |
| TGM7 | <0.001 | -1.713 | <0.001 |
| THAP12P1 | 0.003 | 0.719 | 0.012 |
| THAP12P3 | 0.003 | 1.114 | 0.015 |
| THBS1 | <0.001 | 0.268 | <0.001 |
| THBS4 | <0.001 | 0.547 | <0.001 |
| THEM5 | <0.001 | -0.456 | <0.001 |
| THEMIS | <0.001 | 0.556 | 0.003 |
| THPO | 0.001 | 0.580 | 0.006 |
| TIAM1 | <0.001 | -0.341 | <0.001 |
| TIAM2 | <0.001 | 0.373 | <0.001 |
| TICAM2 | <0.001 | 0.451 | <0.001 |
| TIFAB | 0.006 | 0.440 | 0.025 |
| TIGIT | <0.001 | 0.323 | 0.004 |
| TIMD4 | 0.012 | 0.628 | 0.041 |
| TIMM8AP1 | 0.003 | -0.575 | 0.013 |
| TIMP3 | <0.001 | 0.577 | <0.001 |
| TINAGL1 | <0.001 | 0.312 | <0.001 |
| TINCR | <0.001 | -0.309 | <0.001 |
| TLCD3B | 0.003 | 0.847 | 0.013 |
| TLCD5 | <0.001 | 0.381 | <0.001 |
| TLE2 | 0.003 | 0.278 | 0.015 |
| TLE6 | <0.001 | 0.649 | <0.001 |
| TLL1 | <0.001 | 0.727 | <0.001 |
| TLR1 | <0.001 | 0.292 | 0.002 |
| TLR4 | <0.001 | 0.370 | <0.001 |
| TLR6 | <0.001 | 0.339 | <0.001 |
| TLR7 | <0.001 | 0.566 | <0.001 |
| TLR8 | 0.012 | 0.324 | 0.042 |
| TLR9 | 0.003 | 0.521 | 0.015 |
| TM4SF1-AS1 | 0.007 | -0.404 | 0.027 |
| TM6SF1 | <0.001 | 0.404 | <0.001 |
| TM6SF2 | <0.001 | 0.604 | <0.001 |
| TM7SF2 | <0.001 | -0.669 | <0.001 |
| TMC1 | 0.006 | -0.428 | 0.024 |
| TMC3-AS1 | <0.001 | 0.460 | <0.001 |
| TMC5 | <0.001 | -0.646 | 0.002 |
| TMED7-TICAM2 | <0.001 | 0.310 | 0.005 |
| TMEFF1 | 0.012 | 0.443 | 0.040 |
| TMEM106A | <0.001 | 0.308 | <0.001 |
| TMEM108 | <0.001 | 0.671 | 0.005 |
| TMEM119 | <0.001 | 0.327 | <0.001 |
| TMEM121 | <0.001 | 0.354 | 0.005 |
| TMEM121B | 0.003 | 0.370 | 0.013 |
| TMEM125 | <0.001 | -0.587 | <0.001 |
| TMEM130 | 0.002 | 0.574 | 0.011 |
| TMEM133 | <0.001 | 0.316 | <0.001 |
| TMEM150B | 0.003 | 0.308 | 0.014 |
| TMEM151A | 0.008 | 0.775 | 0.029 |
| TMEM154 | <0.001 | -0.315 | <0.001 |
| TMEM156 | 0.004 | 0.395 | 0.018 |
| TMEM169 | <0.001 | 0.576 | <0.001 |
| TMEM176A | <0.001 | 0.313 | <0.001 |
| TMEM178B | 0.003 | 0.661 | 0.015 |
| TMEM184A | <0.001 | -0.507 | <0.001 |
| TMEM198 | <0.001 | 0.319 | <0.001 |
| TMEM200A | <0.001 | 0.912 | <0.001 |
| TMEM200C | 0.003 | 0.787 | 0.012 |
| TMEM202-AS1 | <0.001 | -0.383 | <0.001 |
| TMEM215 | 0.003 | 1.033 | 0.013 |
| TMEM231 | <0.001 | 0.376 | <0.001 |
| TMEM233 | <0.001 | 0.667 | <0.001 |
| TMEM238L | <0.001 | -1.045 | <0.001 |
| TMEM240 | <0.001 | 0.387 | 0.005 |
| TMEM244 | <0.001 | 0.816 | <0.001 |
| TMEM246-AS1 | 0.006 | -0.646 | 0.023 |
| TMEM255A | 0.014 | 0.340 | 0.045 |
| TMEM255B | <0.001 | 0.345 | <0.001 |
| TMEM26 | <0.001 | 0.959 | <0.001 |
| TMEM26-AS1 | <0.001 | 0.886 | <0.001 |
| TMEM272 | 0.002 | 0.707 | 0.008 |
| TMEM273 | <0.001 | 0.281 | 0.002 |
| TMEM31 | <0.001 | 0.653 | 0.005 |
| TMEM40 | <0.001 | -0.361 | <0.001 |
| TMEM45B | <0.001 | -0.918 | <0.001 |
| TMEM47 | <0.001 | 0.283 | <0.001 |
| TMEM63C | <0.001 | -0.562 | <0.001 |
| TMEM72-AS1 | <0.001 | 0.797 | <0.001 |
| TMEM74 | 0.003 | 0.547 | 0.015 |
| TMEM79 | <0.001 | -0.288 | <0.001 |
| TMEM86A | <0.001 | -0.272 | 0.002 |
| TMEM88B | <0.001 | -0.766 | 0.001 |
| TMEM8B | <0.001 | 0.465 | <0.001 |
| TMEM92 | <0.001 | 0.410 | <0.001 |
| TMEM97P1 | <0.001 | -0.780 | 0.002 |
| TMIGD3 | <0.001 | 0.387 | <0.001 |
| TMOD1 | <0.001 | 0.718 | <0.001 |
| TMOD2 | <0.001 | 0.275 | <0.001 |
| TMPRSS11A | <0.001 | -1.139 | <0.001 |
| TMPRSS11B | <0.001 | -1.283 | <0.001 |
| TMPRSS11BNL | <0.001 | -1.621 | <0.001 |
| TMPRSS11D | <0.001 | -0.583 | <0.001 |
| TMPRSS11E | <0.001 | -0.840 | <0.001 |
| TMPRSS11F | <0.001 | -1.283 | <0.001 |
| TMPRSS11GP | <0.001 | -1.574 | <0.001 |
| TMPRSS13 | <0.001 | -0.440 | <0.001 |
| TMPRSS2 | 0.002 | -0.466 | 0.010 |
| TMPRSS3 | <0.001 | 1.101 | <0.001 |
| TMPRSS4 | <0.001 | -0.424 | <0.001 |
| TMSB10P1 | <0.001 | 0.348 | <0.001 |
| TMSB15B | <0.001 | 0.656 | <0.001 |
| TMSB4XP1 | <0.001 | 0.387 | <0.001 |
| TMSB4XP2 | 0.002 | 0.391 | 0.009 |
| TNFAIP6 | <0.001 | 0.333 | <0.001 |
| TNFAIP8L2 | <0.001 | 0.336 | <0.001 |
| TNFRSF14-AS1 | 0.015 | 0.296 | 0.048 |
| TNFRSF9 | <0.001 | 0.508 | <0.001 |
| TNFSF11 | <0.001 | 0.733 | <0.001 |
| TNFSF12 | <0.001 | 0.290 | <0.001 |
| TNFSF12-TNFSF13 | <0.001 | 0.555 | 0.003 |
| TNFSF13B | <0.001 | 0.420 | <0.001 |
| TNFSF15 | 0.014 | 0.315 | 0.045 |
| TNFSF18 | <0.001 | -0.736 | <0.001 |
| TNFSF4 | <0.001 | 0.531 | <0.001 |
| TNFSF8 | 0.008 | 0.323 | 0.030 |
| TNIK | <0.001 | 0.528 | <0.001 |
| TNS3 | <0.001 | 0.365 | <0.001 |
| TOMM20P2 | <0.001 | 0.519 | 0.003 |
| TOX | <0.001 | 0.668 | <0.001 |
| TOX2 | <0.001 | 0.351 | <0.001 |
| TP53INP2 | <0.001 | -0.324 | <0.001 |
| TPBGL | <0.001 | 0.476 | 0.002 |
| TPH1 | 0.013 | 0.406 | 0.043 |
| TPM1 | <0.001 | 0.320 | <0.001 |
| TPM1-AS | <0.001 | 0.928 | <0.001 |
| TPPP | <0.001 | -0.329 | <0.001 |
| TPRG1 | <0.001 | -0.780 | <0.001 |
| TPRG1-AS1 | 0.002 | -0.352 | 0.008 |
| TPRG1-AS2 | <0.001 | -1.504 | <0.001 |
| TPRXL | <0.001 | -0.361 | <0.001 |
| TPT1P5 | <0.001 | 0.523 | <0.001 |
| TRABD2A | <0.001 | 0.621 | <0.001 |
| TRABD2B | <0.001 | 0.624 | <0.001 |
| TRAF1 | <0.001 | 0.356 | <0.001 |
| TRAF3IP2-AS1 | <0.001 | 0.287 | 0.001 |
| TRAF3IP3 | 0.001 | 0.284 | 0.006 |
| TRAF5 | <0.001 | 0.376 | <0.001 |
| TRAT1 | 0.001 | 0.558 | 0.006 |
| TRAV1-2 | 0.003 | 0.532 | 0.013 |
| TRAV12-1 | <0.001 | 0.594 | 0.001 |
| TRAV12-2 | 0.004 | 0.395 | 0.017 |
| TRAV12-3 | <0.001 | 0.663 | <0.001 |
| TRAV13-1 | 0.002 | 0.417 | 0.009 |
| TRAV13-2 | 0.003 | 0.449 | 0.013 |
| TRAV14DV4 | <0.001 | 0.579 | 0.005 |
| TRAV16 | 0.006 | 0.404 | 0.024 |
| TRAV17 | <0.001 | 0.513 | 0.004 |
| TRAV18 | 0.005 | 0.592 | 0.019 |
| TRAV19 | 0.002 | 0.459 | 0.010 |
| TRAV2 | 0.002 | 0.455 | 0.010 |
| TRAV20 | 0.012 | 0.452 | 0.041 |
| TRAV21 | 0.005 | 0.398 | 0.020 |
| TRAV22 | 0.011 | 0.444 | 0.037 |
| TRAV23DV6 | 0.003 | 0.538 | 0.014 |
| TRAV25 | <0.001 | 0.778 | <0.001 |
| TRAV26-1 | <0.001 | 0.489 | 0.002 |
| TRAV26-2 | 0.005 | 0.535 | 0.019 |
| TRAV27 | <0.001 | 0.599 | 0.003 |
| TRAV29DV5 | 0.002 | 0.483 | 0.008 |
| TRAV3 | 0.005 | 0.446 | 0.019 |
| TRAV30 | 0.009 | 0.541 | 0.032 |
| TRAV34 | 0.003 | 0.650 | 0.015 |
| TRAV35 | 0.002 | 0.572 | 0.009 |
| TRAV36DV7 | <0.001 | 0.575 | 0.004 |
| TRAV38-1 | 0.010 | 0.553 | 0.035 |
| TRAV39 | <0.001 | 0.539 | 0.004 |
| TRAV4 | 0.001 | 0.480 | 0.007 |
| TRAV41 | 0.005 | 0.507 | 0.022 |
| TRAV5 | 0.002 | 0.519 | 0.009 |
| TRAV6 | <0.001 | 0.702 | 0.001 |
| TRAV8-1 | <0.001 | 0.793 | <0.001 |
| TRAV8-2 | <0.001 | 0.518 | 0.003 |
| TRAV8-3 | 0.002 | 0.429 | 0.009 |
| TRAV8-4 | 0.002 | 0.444 | 0.010 |
| TRAV8-6 | <0.001 | 0.472 | 0.004 |
| TRAV9-2 | 0.001 | 0.461 | 0.006 |
| TRBC1 | 0.004 | 0.411 | 0.016 |
| TRBJ2-1 | <0.001 | 0.571 | <0.001 |
| TRBJ2-2P | 0.007 | 0.486 | 0.027 |
| TRBJ2-3 | 0.002 | 0.461 | 0.010 |
| TRBJ2-7 | <0.001 | 0.432 | 0.005 |
| TRBV10-3 | 0.004 | 0.464 | 0.016 |
| TRBV11-1 | <0.001 | 0.993 | <0.001 |
| TRBV11-3 | 0.015 | 0.603 | 0.049 |
| TRBV12-4 | 0.004 | 0.481 | 0.017 |
| TRBV13 | 0.002 | 0.746 | 0.009 |
| TRBV15 | <0.001 | 0.800 | <0.001 |
| TRBV18 | 0.008 | 0.381 | 0.030 |
| TRBV19 | 0.001 | 0.446 | 0.007 |
| TRBV2 | <0.001 | 0.539 | 0.003 |
| TRBV20-1 | 0.003 | 0.312 | 0.012 |
| TRBV21-1 | 0.002 | 0.724 | 0.009 |
| TRBV24-1 | <0.001 | 0.726 | <0.001 |
| TRBV27 | 0.014 | 0.502 | 0.047 |
| TRBV29-1 | 0.003 | 0.431 | 0.013 |
| TRBV3-1 | <0.001 | 0.526 | 0.003 |
| TRBV30 | 0.002 | 0.515 | 0.011 |
| TRBV4-1 | 0.003 | 0.552 | 0.013 |
| TRBV4-2 | <0.001 | 0.665 | 0.001 |
| TRBV5-1 | 0.003 | 0.398 | 0.015 |
| TRBV5-4 | <0.001 | 0.532 | 0.003 |
| TRBV5-5 | 0.008 | 0.549 | 0.029 |
| TRBV5-6 | 0.009 | 0.425 | 0.032 |
| TRBV6-1 | 0.001 | 0.481 | 0.008 |
| TRBV6-5 | <0.001 | 0.558 | 0.001 |
| TRBV7-3 | 0.001 | 0.497 | 0.006 |
| TRBV7-4 | 0.006 | 0.645 | 0.025 |
| TRBV7-6 | 0.008 | 0.512 | 0.031 |
| TRBV7-9 | 0.005 | 0.347 | 0.021 |
| TRBV9 | 0.003 | 0.445 | 0.013 |
| TRDMT1 | <0.001 | 0.316 | <0.001 |
| TRDN | 0.010 | 0.593 | 0.035 |
| TREM1 | 0.003 | 0.305 | 0.015 |
| TREML2 | 0.009 | 0.491 | 0.032 |
| TREX2 | <0.001 | -1.152 | <0.001 |
| TRGC1 | 0.002 | 0.485 | 0.011 |
| TRGC2 | 0.003 | 0.440 | 0.014 |
| TRGV10 | 0.003 | 0.443 | 0.014 |
| TRGV2 | 0.006 | 0.565 | 0.022 |
| TRGV3 | 0.004 | 0.480 | 0.016 |
| TRGV9 | 0.005 | 0.600 | 0.020 |
| TRHDE | 0.004 | 0.903 | 0.016 |
| TRIL | 0.001 | 0.405 | 0.006 |
| TRIM31-AS1 | <0.001 | -0.727 | <0.001 |
| TRIM36 | <0.001 | 0.516 | <0.001 |
| TRIM46 | <0.001 | 0.419 | <0.001 |
| TRIM55 | 0.001 | 0.714 | 0.008 |
| TRIM7 | <0.001 | -0.409 | <0.001 |
| TRIML1 | <0.001 | -1.317 | <0.001 |
| TRIML2 | 0.007 | 0.786 | 0.028 |
| TRMT9B | 0.014 | 0.478 | 0.047 |
| TRO | 0.001 | 0.365 | 0.006 |
| TRPC1 | <0.001 | 0.421 | <0.001 |
| TRPC2 | 0.014 | 0.438 | 0.045 |
| TRPC3 | 0.004 | 0.631 | 0.016 |
| TRPM6 | <0.001 | -0.602 | <0.001 |
| TRPV3 | <0.001 | -0.638 | <0.001 |
| TSGA10IP | <0.001 | -1.506 | <0.001 |
| TSHZ3 | <0.001 | 0.363 | <0.001 |
| TSPAN2 | <0.001 | 0.752 | <0.001 |
| TSPAN4 | <0.001 | 0.326 | <0.001 |
| TSPEAR | 0.010 | 0.888 | 0.037 |
| TSPOAP1-AS1 | 0.002 | 0.407 | 0.009 |
| TSPYL5 | 0.010 | 0.342 | 0.034 |
| TSSC2 | <0.001 | -0.923 | 0.004 |
| TTC21A | <0.001 | 0.319 | 0.002 |
| TTC22 | <0.001 | -0.381 | <0.001 |
| TTC23L | <0.001 | 0.526 | 0.005 |
| TTC25 | 0.007 | 0.373 | 0.026 |
| TTC39A | <0.001 | -0.932 | <0.001 |
| TTC39A-AS1 | <0.001 | -1.126 | <0.001 |
| TTC39B | <0.001 | -0.343 | <0.001 |
| TTC4P1 | 0.006 | 0.783 | 0.022 |
| TTC9 | <0.001 | -0.588 | <0.001 |
| TTLL11 | <0.001 | 0.277 | <0.001 |
| TTLL11-IT1 | 0.005 | 0.461 | 0.020 |
| TTLL7 | <0.001 | 0.553 | <0.001 |
| TTN | 0.008 | 0.660 | 0.031 |
| TTTY15 | 0.003 | 0.480 | 0.013 |
| TTYH1 | 0.008 | -0.510 | 0.029 |
| TUB | 0.001 | 0.354 | 0.006 |
| TUBA3FP | <0.001 | 0.497 | 0.002 |
| TUBA8 | <0.001 | 0.501 | 0.004 |
| TUBAP2 | 0.001 | 0.265 | 0.006 |
| TUBB2BP1 | <0.001 | -0.640 | 0.003 |
| TUBB7P | 0.013 | -0.992 | 0.044 |
| TUBB8B | 0.012 | -0.355 | 0.040 |
| TUBB8P7 | <0.001 | -1.672 | <0.001 |
| TUBB8P8 | 0.003 | -0.522 | 0.013 |
| TUBBP2 | <0.001 | 0.390 | 0.003 |
| TULP1 | 0.002 | -0.585 | 0.008 |
| TULP2 | 0.013 | -0.477 | 0.043 |
| TVP23A | 0.008 | 0.273 | 0.029 |
| TVP23C-CDRT4 | 0.003 | 0.307 | 0.013 |
| TWIST1 | <0.001 | 0.277 | <0.001 |
| TXLNB | 0.014 | 0.411 | 0.047 |
| TXNDC2 | 0.014 | 0.312 | 0.045 |
| TYRO3 | <0.001 | -0.284 | <0.001 |
| UBASH3A | <0.001 | 0.478 | <0.001 |
| UBD | <0.001 | 0.375 | 0.002 |
| UBE2QL1 | <0.001 | 0.675 | <0.001 |
| UBE2R2-AS1 | <0.001 | -0.658 | <0.001 |
| UBQLNL | 0.012 | 0.378 | 0.041 |
| UBXN10 | 0.001 | 0.522 | 0.007 |
| UBXN10-AS1 | <0.001 | 0.852 | <0.001 |
| UCP3 | 0.003 | 0.488 | 0.013 |
| UGT1A1 | 0.009 | -0.607 | 0.033 |
| UGT1A6 | <0.001 | -0.584 | 0.004 |
| UGT1A7 | <0.001 | -1.310 | <0.001 |
| UGT1A8 | 0.001 | -1.417 | 0.006 |
| UNC45B | 0.002 | 0.722 | 0.010 |
| UNC5A | <0.001 | 0.606 | 0.001 |
| UNC5B-AS1 | <0.001 | -0.538 | <0.001 |
| UNC5C | <0.001 | 0.947 | <0.001 |
| UNC93A | <0.001 | -1.075 | <0.001 |
| UPK1A | <0.001 | -1.512 | <0.001 |
| UPK1B | <0.001 | -0.939 | <0.001 |
| UPK2 | <0.001 | -0.603 | 0.001 |
| UPK3B | <0.001 | -0.384 | 0.004 |
| UPK3BL1 | 0.012 | -0.646 | 0.041 |
| UQCRHP2 | 0.006 | -0.715 | 0.023 |
| URAHP | 0.003 | -0.341 | 0.015 |
| USH1G | <0.001 | -0.666 | <0.001 |
| USP12-AS2 | <0.001 | 0.695 | 0.003 |
| USP2 | <0.001 | -0.399 | <0.001 |
| USP50 | <0.001 | 0.593 | 0.004 |
| USP51 | <0.001 | 0.377 | 0.002 |
| USP54 | <0.001 | -0.270 | <0.001 |
| USP6NL | <0.001 | -0.310 | <0.001 |
| UTS2B | 0.007 | 0.410 | 0.028 |
| UTS2R | 0.005 | 0.659 | 0.019 |
| VASH2 | <0.001 | 0.736 | <0.001 |
| VAT1L | 0.002 | 0.653 | 0.011 |
| VCAM1 | <0.001 | 0.444 | <0.001 |
| VCAN | <0.001 | 0.294 | <0.001 |
| VCAN-AS1 | 0.003 | 0.686 | 0.012 |
| VEGFC | <0.001 | 0.421 | <0.001 |
| VENTX | 0.003 | 0.384 | 0.014 |
| VEPH1 | <0.001 | 0.835 | <0.001 |
| VGLL1 | <0.001 | -0.923 | <0.001 |
| VGLL2 | 0.005 | 0.781 | 0.021 |
| VGLL3 | <0.001 | 0.405 | 0.002 |
| VIM-AS1 | <0.001 | 0.728 | <0.001 |
| VN1R108P | <0.001 | 0.550 | <0.001 |
| VN1R20P | 0.004 | 0.533 | 0.018 |
| VN1R80P | 0.008 | -0.473 | 0.028 |
| VNN1 | <0.001 | -0.543 | <0.001 |
| VNN3 | <0.001 | -0.874 | <0.001 |
| VSIG10L | <0.001 | -0.798 | <0.001 |
| VSIG2 | <0.001 | -0.609 | 0.004 |
| VSIG4 | <0.001 | 0.297 | 0.003 |
| VSIG8 | <0.001 | -1.640 | <0.001 |
| VSTM1 | 0.006 | 0.633 | 0.025 |
| VSTM2L | <0.001 | 0.572 | <0.001 |
| VSTM4 | <0.001 | 0.477 | <0.001 |
| VWA8-AS1 | <0.001 | -0.551 | 0.003 |
| VWC2 | 0.006 | 1.474 | 0.024 |
| WDFY4 | <0.001 | 0.408 | 0.005 |
| WDR19 | <0.001 | 0.291 | <0.001 |
| WDR63 | 0.015 | 0.402 | 0.050 |
| WDR64 | <0.001 | 0.536 | 0.005 |
| WDR78 | <0.001 | 0.478 | 0.001 |
| WDR86 | <0.001 | 0.482 | <0.001 |
| WFDC12 | <0.001 | -1.176 | <0.001 |
| WFDC21P | <0.001 | -0.499 | <0.001 |
| WFDC5 | <0.001 | -0.611 | <0.001 |
| WHAMMP3 | 0.001 | 0.329 | 0.006 |
| WHRN | <0.001 | 0.287 | <0.001 |
| WIPF1 | <0.001 | 0.296 | <0.001 |
| WNT2B | <0.001 | -0.692 | <0.001 |
| WNT4 | <0.001 | -0.279 | <0.001 |
| WNT5B | <0.001 | 0.568 | <0.001 |
| WNT6 | <0.001 | 0.712 | <0.001 |
| WNT7A | 0.007 | 0.289 | 0.026 |
| WSCD1 | <0.001 | 0.922 | <0.001 |
| WTAPP1 | 0.002 | 0.523 | 0.010 |
| WTIP | <0.001 | 0.426 | <0.001 |
| WWP1P1 | 0.010 | 0.411 | 0.036 |
| XCR1 | 0.004 | -0.394 | 0.019 |
| XIAPP3 | 0.004 | -0.850 | 0.016 |
| XIRP1 | 0.002 | 0.556 | 0.012 |
| XK | <0.001 | -0.689 | <0.001 |
| XKR4 | 0.002 | 1.948 | 0.009 |
| XKRX | <0.001 | -0.760 | <0.001 |
| XRCC6P1 | 0.007 | -0.586 | 0.027 |
| YIPF7 | 0.009 | 0.828 | 0.032 |
| YOD1 | <0.001 | -0.322 | <0.001 |
| YPEL1 | <0.001 | 0.648 | <0.001 |
| YWHAEP1 | 0.006 | -0.783 | 0.024 |
| Z69720.2 | 0.006 | -0.456 | 0.022 |
| Z73417.1 | <0.001 | -0.607 | 0.001 |
| Z73420.1 | 0.001 | -0.456 | 0.006 |
| Z82188.2 | 0.006 | 0.497 | 0.022 |
| Z82243.1 | <0.001 | -0.385 | <0.001 |
| Z85994.1 | <0.001 | 1.000 | 0.001 |
| Z92544.2 | 0.003 | -0.265 | 0.013 |
| Z93930.3 | 0.002 | -0.285 | 0.012 |
| Z94721.1 | 0.001 | 0.370 | 0.007 |
| Z97200.1 | <0.001 | 0.709 | 0.005 |
| Z97653.2 | <0.001 | -0.678 | <0.001 |
| Z97832.2 | 0.003 | 0.402 | 0.015 |
| Z97989.1 | <0.001 | 0.410 | 0.003 |
| Z98885.2 | 0.001 | -0.687 | 0.006 |
| Z99289.1 | <0.001 | 0.735 | <0.001 |
| Z99289.2 | 0.003 | 0.580 | 0.015 |
| Z99916.1 | 0.007 | -0.543 | 0.028 |
| ZAN | <0.001 | -0.847 | <0.001 |
| ZBED3-AS1 | <0.001 | 0.598 | 0.003 |
| ZBTB10 | <0.001 | 0.531 | <0.001 |
| ZBTB46 | <0.001 | 0.349 | <0.001 |
| ZBTB7C | <0.001 | -0.560 | <0.001 |
| ZBTB8B | 0.013 | 1.280 | 0.044 |
| ZC2HC1A | <0.001 | 0.266 | <0.001 |
| ZC3H12D | <0.001 | 0.370 | 0.005 |
| ZC4H2 | 0.004 | 0.333 | 0.016 |
| ZCCHC18 | <0.001 | 0.719 | <0.001 |
| ZCWPW2 | <0.001 | 0.393 | <0.001 |
| ZDHHC11B | <0.001 | -0.612 | <0.001 |
| ZDHHC20-IT1 | 0.014 | 0.345 | 0.047 |
| ZDHHC20P1 | <0.001 | 0.648 | <0.001 |
| ZDHHC21 | <0.001 | -0.452 | <0.001 |
| ZDHHC4P1 | 0.013 | 0.718 | 0.045 |
| ZDHHC8P1 | 0.004 | 0.593 | 0.016 |
| ZEB1 | <0.001 | 0.362 | <0.001 |
| ZEB1-AS1 | <0.001 | 0.293 | <0.001 |
| ZEB2 | <0.001 | 0.423 | <0.001 |
| ZEB2-AS1 | <0.001 | 0.505 | 0.004 |
| ZFHX2 | 0.010 | 0.454 | 0.037 |
| ZFP2 | <0.001 | 0.415 | 0.002 |
| ZFP92 | 0.009 | 0.274 | 0.032 |
| ZFPM2 | <0.001 | 0.549 | <0.001 |
| ZKSCAN7 | <0.001 | 0.334 | 0.003 |
| ZMIZ1-AS1 | <0.001 | 0.453 | 0.003 |
| ZNF154 | 0.002 | 0.305 | 0.011 |
| ZNF185 | <0.001 | -0.270 | <0.001 |
| ZNF236-DT | <0.001 | 0.366 | <0.001 |
| ZNF239 | <0.001 | 0.415 | 0.002 |
| ZNF251 | <0.001 | 0.264 | <0.001 |
| ZNF382 | <0.001 | 0.451 | <0.001 |
| ZNF385B | 0.010 | 0.517 | 0.035 |
| ZNF385D | 0.004 | 0.402 | 0.017 |
| ZNF423 | <0.001 | 0.411 | 0.001 |
| ZNF426 | <0.001 | -0.302 | <0.001 |
| ZNF431 | <0.001 | -0.280 | <0.001 |
| ZNF438 | <0.001 | 0.276 | <0.001 |
| ZNF469 | <0.001 | 0.427 | <0.001 |
| ZNF474 | 0.009 | 0.434 | 0.032 |
| ZNF488 | <0.001 | 0.386 | <0.001 |
| ZNF491 | 0.009 | 0.314 | 0.033 |
| ZNF521 | <0.001 | 0.374 | 0.001 |
| ZNF602P | <0.001 | 0.659 | <0.001 |
| ZNF608 | <0.001 | 0.454 | <0.001 |
| ZNF618 | <0.001 | 0.313 | <0.001 |
| ZNF630 | 0.001 | 0.276 | 0.006 |
| ZNF660 | <0.001 | 0.492 | <0.001 |
| ZNF675 | <0.001 | -0.301 | 0.004 |
| ZNF681 | 0.008 | -0.376 | 0.030 |
| ZNF683 | 0.008 | 0.386 | 0.031 |
| ZNF697 | <0.001 | 0.272 | <0.001 |
| ZNF704 | <0.001 | 0.535 | <0.001 |
| ZNF713 | <0.001 | 0.379 | 0.005 |
| ZNF732 | 0.013 | -0.669 | 0.044 |
| ZNF750 | <0.001 | -0.433 | <0.001 |
| ZNF804A | <0.001 | 0.900 | <0.001 |
| ZNF807P | 0.010 | 0.360 | 0.037 |
| ZNF812P | <0.001 | -0.682 | <0.001 |
| ZNF826P | 0.003 | 0.596 | 0.015 |
| ZNF835 | 0.003 | 0.377 | 0.013 |
| ZNF853 | 0.001 | 0.367 | 0.007 |
| ZNF878 | 0.009 | -0.438 | 0.033 |
| ZRANB2-AS2 | 0.007 | 0.335 | 0.026 |
| ZSCAN12P1 | 0.001 | -0.276 | 0.008 |
| ZSCAN2 | <0.001 | 0.375 | <0.001 |
| ZSCAN31 | <0.001 | -0.308 | <0.001 |
