# Supplementary material for: Predictive and mechanistic insights of GLTP on survival in patients with head and neck squamous cell carcinoma
Source: PeerJ. 2026 Aug 5;14:e21611. doi: 10.7717/peerj.21611 (PMC13452426; doi:10.7717/peerj.21611)
Supplement: Supplementary file 3 [file peerj-14-21611-s007.docx]

**Table S3. The pathways of DEGs identified through GSEA in the TCGA-HNSCC cohort.**

| Description | enrichmentScore | NES | pvalue | FDR | setSize |
| --- | --- | --- | --- | --- | --- |
| Calcium signaling pathway  cGMP-PKG signaling pathway  cAMP signaling pathway  Chemokine signaling pathway  PI3K-Akt signaling pathway | 0.493; 0.536; 0.471; 0.467; 0.405 | 1.700; 1.775; 1.598; 1.572; 1.431 | < 0.001  < 0.001  < 0.001  < 0.001  < 0.001 | < 0.001  < 0.001  0.002  0.004  0.011 | 240; 160; 208; 187; 328 |

DEG, differentially expressed genes; TCGA, the Cancer Genome Atlas; HNSCC, head and neck squamous cell carcinoma; NES, normalized enrichment score; FDR, false discovery rate.
