# Supplementary material for: Predictive and mechanistic insights of GLTP on survival in patients with head and neck squamous cell carcinoma
Source: PeerJ. 2026 Aug 5;14:e21611. doi: 10.7717/peerj.21611 (PMC13452426; doi:10.7717/peerj.21611)
Supplement: Supplementary file 4 [file peerj-14-21611-s008.docx]

**Table S4. Clinicopathological characteristics of the patients in the TCGA HNSCC cohort and the GSE41613 cohort.**

|  | TCGA-HNSCC cohort (340) | GSE41613 cohort (97) |
| --- | --- | --- |
| Age (years) |  |  |
| ≤60 | 167 | N/A |
| >60 | 173 | N/A |
| Sex |  |  |
| Male | 256 | 31 |
| Female | 84 | 66 |
| Grade |  |  |
| I/II | 245 | 41 |
| III/IV | 95 | 56 |
| Stage |  |  |
| I/II | 64 | N/A |
| III/IV | 276 | N/A |
| T |  |  |
| T_1_/T_2_ | 115 | N/A |
| T_3_/T_4_ | 225 | N/A |
| M |  |  |
| M_1_ | 336 | N/A |
| M_2_ | 4 | N/A |
| N |  |  |
| N_0_/N_1_ | 171 | N/A |
| N_2_/N_3_ | 119 | N/A |
| fustat |  |  |
| Alive | 217 | 46 |
| Dead | 160 | 51 |
